# Soft Yet Tough Tanglemers Through Rapid Photopolymerization for DLP 3D Printing

**DOI:** 10.1002/adma.74661

**Published:** 2026-08-19

**Authors:** Yutong Liu, Tiffany N. Jeng, Elizabeth A. Recker, Ellen J. Forehand, Gabriel E. Sanoja, Zachariah A. Page

**Affiliations:** ^1^ Department of Chemistry The University of Texas at Austin Austin Texas USA; ^2^ McKetta Department of Chemical Engineering The University of Texas at Austin Austin Texas USA

**Keywords:** 3D printing, digital light processing, entanglement, photopolymerization, toughness

## Abstract

Additive manufacturing of compliant materials that simultaneously combine softness, elasticity, and toughness remains a central challenge. Here, we report a single‐resin photopolymerization strategy to produce densely entangled polymer networks (“tanglemers”) via digital light processing (DLP) 3D printing using industrially abundant monomers. Comparisons between controlled and uncontrolled radical polymerizations reveal that the uncontrolled, free‐radical approach at low initiator loadings and light intensities balances DLP‐relevant gelation timescale with high molecular weight between crosslinks. The resulting elastomers and hydrogels exhibit a rare combination of properties, including low modulus (*E*_T_ <1 MPa for elastomers and <100 kPa for hydrogels), high extensibility (*ε*_f_ > 400%), low hysteresis (∼10%), and high toughness (*U*_t_ > 1 MJ m^−^^3^, *G*_c_ ≈ 1 kJ m^−^^2^ for elastomers; *U*_t_ ≈ 1 MJ m^−^^3^, *G*_c_ ≈ 3 kJ m^−^^2^ for hydrogels). These materials translate from casting to DLP 3D printing with feature sizes down to ∼100 µm, surpassing commercial resins in softness and elasticity, while maintaining competitive toughness and aging resistance after initial accelerated weathering. This work establishes simple, single‐resin formulations as a route to DLP‐printable, defect‐tolerant tanglemers with potential relevance to soft robotic, wearable, and biomedical technologies.

## Graphical abstract

Soft yet tough tanglemers are produced through low‐initiator free radical photopolymerization and translated to DLP 3D printing. Systematic control over light intensity, initiator loading, and crosslink density reveals processing windows that balance printability, elasticity, toughness, and feature fidelity.

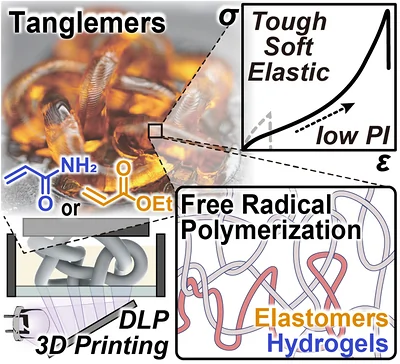

## 1 Introduction

Additive manufacturing (3D printing) has emerged as a powerful platform for fabricating functional materials with precisely controlled geometries and programmable properties. Among printable materials, soft elastomers and hydrogels have attracted particular interest in human‐facing applications, ranging from soft robotics [1, 2, 3] to biomedical devices [4, 5]. However, achieving materials that simultaneously combine softness, elasticity, strength, and toughness remains a central and unresolved challenge in 3D printing [6, 7].

Exemplifying the demand for soft yet mechanically robust materials are the extensive efforts by the 3D printing community in recent years [8, 9, 10, 11, 12, 13, 14, 15]. Notable examples include the implementation of bottlebrush polymers in extrusion [11] and digital light processing (DLP) 3D printing [12, 13] to produce elastomers with tensile modulus (*E*_T_) below 100 kPa (Figure 1A‐i). This approach leverages densely grafted sidechains to suppress entanglements; however, despite achieving exceptional softness, the tensile strength (*σ*_m_ ≤ 0.15 MPa) and stretchability (fracture strain, *ε*_f_ ≤ 25%) remain limited. An alternative strategy prioritized strength and modulus of toughness (*U*_t_, defined here as the area under the stress‐stretch curve or strain energy density of a defect‐free specimen up to failure) through dense hydrogen bonding and dynamic urea bond exchange enabled by thermal post‐curing [16] (Figure 1A‐ii). In these custom materials, heat treatment results in *σ*_m_ values up to ≈ 90 MPa and *U*_t_ up to ≈ 300 MJ m^−3^, albeit at the expense of increased stiffness (*E*_T_ ≥ 26 MPa) and hysteresis. Notably, fracture toughness (*G*_c_), which quantifies resistance to crack propagation and thus sensitivity to defects, was not reported for these systems and remains rarely measured in 3D‐printed materials, despite the ubiquity of defects in additively manufactured parts.

**FIGURE 1:**
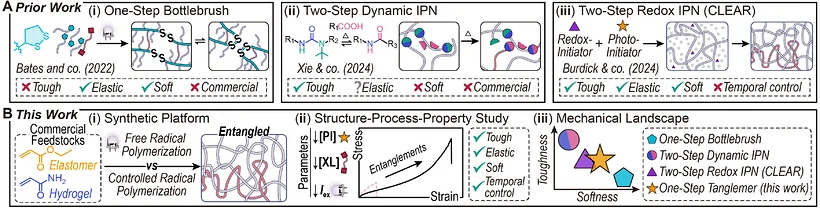
Design strategies and mechanical tradeoffs in 3D printing soft and tough polymer networks. (A) Prior approaches to DLP 3D printing of soft and/or tough materials. (i) One‐step soft bottlebrush polymer networks [12, 13], (ii) two‐step tough dynamic interpenetrating polymer networks [14], and (iii) two‐step soft and tough CLEAR printing combining light and dark (redox) polymerizations [21]. (B) This work: (i) free and controlled radical polymerizations to form entangled elastomers and hydrogels, (ii) tuning of structure–process–property relationships via formulation and light conditions, and (iii) access to a mechanical landscape combining softness, elasticity, and toughness, enabling one‐step DLP 3D printing.

To decouple toughness (*U*_t_ and *G*_c_) from stiffness (*E*), polymer networks have recently been designed with a high density of entanglements relative to permanent crosslinks, giving rise to materials termed “tanglemers” [6, 17, 18, 19]. Tanglemers require the formation of high molecular weight polymer chains between crosslinks, as well as low‐to‐moderate entanglement molecular weight (*M*_e_), defined as the average molecular weight between entanglements. To date, hydrogel‐ and elastomer‐based tanglemers have primarily been prepared by casting or molding using very low initiator and crosslinker concentrations relative to (neat) monomers or functionalized polymers [18, 20, 21, 22, 23, 24]. Recently, this concept was extended to additive manufacturing through continuous curing after light exposure aided by redox initiation (CLEAR), wherein light defines the printed geometry while a redox‐initiated dark polymerization drives continued monomer conversion and entanglement formation after exposure [25] (Figure 1A‐iii). This approach enables entangled hydrogels with *E*_T_ ≈ 50–290 kPa, *U*_t_ ≈ 0.15–1.7 MJ m^−3^, and excellent elasticity under cyclic loading. However, the secondary redox process compromises temporal control, limiting resin pot life (hours or less). Thus, a simple, one‐step strategy to rapidly 3D print materials that simultaneously combine softness, strength, elasticity, and toughness remains elusive.

Here, we report a simple formulation and process to rapidly produce mechanically robust tanglemers, including elastomers as the primary focus and hydrogels as a complementary platform, and demonstrate their direct translation from casting to DLP 3D printing (Figure 1B). The resins are composed primarily of industrially abundant monomers—neat ethyl acrylate (EA) for elastomers and aqueous acrylamide (AAm) for hydrogels—combined with inexpensive acrylate crosslinkers and acylphosphine oxide photoinitiators at sub‐mol% loadings. Initial comparisons between controlled and uncontrolled (“free”) radical polymerizations establish that the latter provides an optimal balance of curing speed, low light intensity, and ultrahigh polymer molecular weight between crosslinks, enabling tanglemers with exceptional mechanical performance. These resins exhibit excellent spatiotemporal control, achieving printed features down to ∼100 µm. Within selected processing windows, the resulting materials display a rare combination of softness (*E_T_* < 1 MPa; < 30 Shore A), high extensibility (ε_*f*_ > 400%; low hysteresis loss ≈ 10%), strength (σ_*m*_ ≈ 2 MPa for elastomers and ≈ 0.5 MPa for hydrogels), and toughness (*U_t_* > 1 MJ m^−^^3^ and, for elastomers, *G_c_* ≈ 1 kJ m^−^^2^), while also exhibiting substantially improved stability under accelerated weathering relative to benchmark commercial DLP elastomer resins.

## 2 Results and Discussion

### 2.1 Linear Poly(EA): Controlled Versus Uncontrolled Photopolymerization

EA was chosen as the monomer for elastomer synthesis because poly(EA) combines a low glass transition temperature (*T*_g_ ≈ −20°C) with a relatively low entanglement molecular weight (*M*_e_ ≈ 12 kg mol^−^^1^) [26], making it well suited for forming tanglemers. Real‐time Fourier transform infrared (FTIR) spectroscopy in transmission mode was used to characterize photopolymerization kinetics by monitoring the decrease in the combined C═C─H and C═C stretch (∼4450–4550 cm^−^^1^), with validation from the C═C─H overtone (∼6100–6250 cm^−^^1^) [27, 28] (Figures S1–S3). Polymerizations were conducted between glass slides separated by 100‐µm‐thick spacers, irradiating with 365 or 405 nm LEDs at varying intensities and exposure times. Light exposure was initiated 10 s after the start of each FTIR measurement. Gel permeation chromatography (GPC) was used to determine the molecular weight and dispersity of the resulting poly(EA). Together, FTIR and GPC provide a basis to assess polymerization kinetics and chain growth across controlled and uncontrolled radical polymerization conditions. These uncrosslinked EA polymerizations served as model systems to determine how polymerization mechanism, initiator loading, and light intensity influence chain growth under DLP‐relevant conditions.

Initial studies focused on controlled radical polymerizations via photoiniferter polymerization of neat EA resins using a series of chain transfer agents (CTAs) (Table S1, Figures S4–S18), followed by uncontrolled radical polymerizations using a Type I photoinitiator commonly employed in DLP resins, phenylbis(2,4,6‐trimethylbenzoyl)phosphine oxide (BAPO) (Figure 2A). To accelerate photopolymerization and mitigate oxygen inhibition, triphenylphosphine (PPh_3_, 0.02–1 wt%) was added to the EA resins [29]. At 0.1 wt%, the inhibition time during uncontrolled radical polymerization was reduced from ∼29 s to ∼3 s under 365 nm irradiation (5 mW cm^−^^2^, 0.01 mol% BAPO), which was sufficient for printing and therefore used in all subsequent experiments (Figures S19–S23, Tables S2–S8).

**FIGURE 2:**
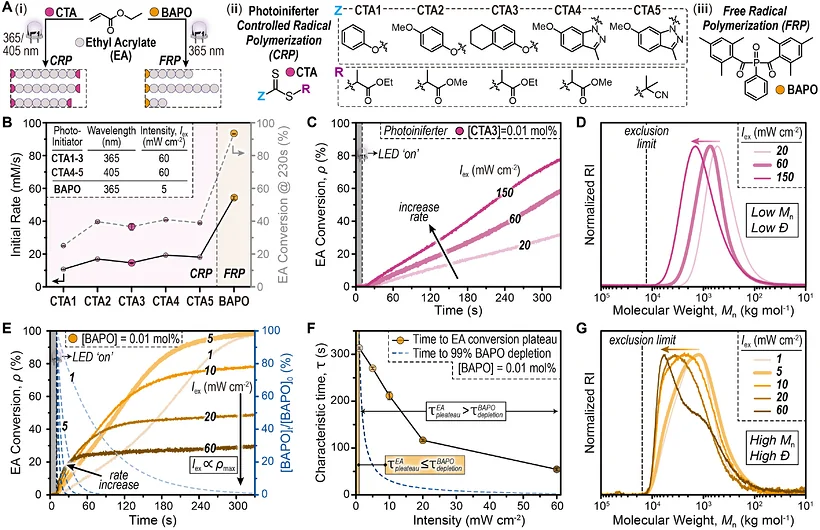
Kinetics of ethyl acrylate conversion via photoiniferter and free radical polymerization. (A) Chemical structures of resin components. (i) Monomer and schematic illustrating chain length distributions expected from controlled versus uncontrolled polymerization. (ii) Xanthate and dithiocarbamate chain‐transfer agents (CTAs) examined. (iii) Type I radical photoinitiator. All samples contain [PPh_3_] = 0.1 wt% to minimize oxygen inhibition. (B) Comparison of photopolymerization mechanisms in terms of initial rate and monomer conversion after 230 s of irradiation. Data are presented as mean values (*n* ≥ 3) ± SD. (C) Real‐time photoiniferter polymerization kinetics monitored with FTIR spectroscopy. Samples (100 µm thick) were irradiated with a 365 nm LED at varying light intensities, with illumination initiated 10 s after the measurement began. (D) Corresponding GPC traces of polymers obtained after 330 s of irradiation. (E) Real‐time free radical photopolymerization kinetics monitored using FTIR spectroscopy (solid orange lines) overlaid with calculated BAPO depletion (dashed blue lines). Samples (100 µm thick) were irradiated with a 365 nm LED at varying light intensities, with illumination initiated 10 s after the measurement began. (F) Time to ethyl acrylate conversion plateau ($\tau_{plateau}^{EA}$, defined at 95% conversion) and calculated BAPO depletion time ($\tau_{depletion}^{BAPO}$, 99% depletion) as functions of light intensity. Data are presented as mean values (*n* ≥ 3) ± SD. (G) Corresponding GPC traces of polymers obtained after 330 s of irradiation at varying light intensities.

For the controlled radical polymerizations, photoiniferter offered a compelling starting point because of its precedent for synthesizing ultrahigh molecular weight polymers, motivating its selection to form tanglemers [30, 31, 32]. Although most implementations rely on commercial xanthate or trithiocarbonate CTAs and require hours of carefully dosed irradiation to reach high molecular weight, recent examples of tailored aryl‐xanthates and dithiocarbamates achieve >80% monomer conversion within minutes [33, 34, 35, 36, 37]. Inspired by these advances, we synthesized four literature‐reported CTAs and one literature‐inspired analogue [33, 34, 35, 36, 37] and evaluated their performance in the photoiniferter polymerization of EA (Figure 2A). At 0.01 mol% CTA loading—corresponding to a target degree of polymerization ≈ 10 000—EA resins were irradiated for 230 s with 365 or 405 nm LEDs (60 mW cm^−^^2^) to maximize overlap with the π→π* transition and thus reactivity [38] (Figure 2B). Increasing the CTA loading to 0.1 mol% (targeting lower molecular weight) did not significantly affect the apparent polymerization rate (Figures S24 and S25, Tables S9–S12).

Across all CTAs (0.01 mol% loading), initial polymerization rates were comparable (∼11–19 mM s^−^^1^), while inhibition times varied modestly (∼8–20 s). On the basis of these similar kinetic profiles and its synthetic accessibility, tetrahydronaphthalenyl xanthate (CTA 3) was selected for further study. For this system, increasing the light intensity from 20 to 150 mW cm^−^^2^ led to a corresponding increase in polymerization rate (≈9–23 mM s^−^^1^) and final monomer conversion after 330 s of irradiation (≈30–78%) (Figure 2C). GPC analysis revealed narrow, Gaussian molecular weight distributions (*Đ* ≈ 1.3) and a linear increase in molecular weight with EA conversion, consistent with the controlled nature of the photoiniferter process (Figure 2D). However, these photoiniferter‐mediated polymerizations required relatively high light intensities and provided limited monomer conversion, motivating the comparison with uncontrolled radical photopolymerization (Figures S26 and S27, Tables S13 and S14).

Turning to uncontrolled radical polymerizations with BAPO resulted in rapid polymerization of EA under low light intensity. At 0.01 mol% BAPO, increasing the 365 nm LED intensity from 1 to 60 mW cm^−^^2^ resulted in a > 5‐fold increase in the initial polymerization rate (≈25–142 mM s^−^^1^) and a corresponding decrease in inhibition time (≈10–0.4 s) (Figure 2E, Tables S15–S17). In contrast, over the same intensity range, the final monomer conversion after 320 s decreased from >98% to 30%, reflecting an early conversion plateau under high radical flux (Figure S28). Two additional 365 nm‐active Type I photoinitiators, 2,2‐dimethoxy‐2‐phenylacetophenone (DMPA) and 2‐hydroxy‐2‐methylpropiophenone (Irgacure 1173), were evaluated at 0.1 and 0.01 mol% and exhibited similar trends, albeit with slower rates than BAPO (Figures S29 and S30).

This premature conversion plateau is attributed to increased bimolecular termination and rapid initiator depletion. Consistent with this interpretation, calculations based on light intensity, absorption cross‐section, and a reported internal quantum yield (*Φ* = 0.6) [39, 40] indicated near‐quantitative BAPO consumption prior to the conversion plateau (Figure 2E,F). Further supporting this mechanism, GPC analysis revealed bimodal molecular weight distributions in line with termination by combination, with ultrahigh number‐average molecular weights (*M_n_* ≈ 700–1100 kg mol^−^^1^) and broad dispersities (*Đ* ≈ 2–3) (Figure 2G, Table S16). Moreover, varying BAPO loading shifted the relative timing of initiator depletion and the conversion plateau: at higher loading (0.1 mol%), depletion preceded the plateau only at light intensities above ∼5 mW cm^−^^2^ and reduced the molecular weight, whereas at lower loading (0.001 mol%), depletion occurred well before the plateau even at <5 mW cm^−^^2^, limiting maximum conversion and promoting bimodal distributions (Figures S31–S35, Tables S18–21). Taken together, these results show that BAPO enables substantially faster polymerization than photoiniferter systems while achieving comparable molecular weights, making it well suited for rapid DLP 3D printing of tanglemers.

### 2.2 Poly(EA) Elastomer Networks: Synthesis and Mechanical Properties

To identify DLP‐relevant conditions that provide soft, tough poly(EA) elastomers, the effects of light intensity, photoinitiator concentration, and diethylene glycol diacrylate (DEGDA) crosslinker (XL) concentration on gelation, sol:gel fraction, and mechanical performance were systematically examined. Resins were mixed, injected between glass slides separated by a 1/32″ thick silicone rubber spacer (∼800 µm), and irradiated with 365 nm LED light (Figures 3A and S36). After irradiation, the films were removed from the mold, weighed, and then dried under vacuum at 40°C for 24 h to remove residual monomer, and determine sol:gel fractions prior to punching into ASTM D‐1708 specimens for mechanical testing. Elastomers prepared by photoiniferter polymerization required substantially higher light intensities and longer exposure times to form gels, whereas BAPO‐mediated free‐radical polymerization produced gels rapidly under conditions more compatible with DLP 3D printing. For the free‐radical‐derived networks, extensive (solvent) washing followed by drying resulted in sol:gel fractions and mechanical performance comparable to those obtained after vacuum drying alone, confirming that vacuum drying was sufficient to remove residual monomer (Figure S37, Tables S22 and S23). Under tension, elastomers prepared by photoiniferter polymerization were only modestly softer and more extensible than BAPO‐derived networks (Figures S38 and S39), consistent with previous studies examining how controlled polymerization affects gelation and mechanical properties of polymer networks [41, 42, 43]. Because uncontrolled radical polymerization enabled faster gelation under DLP‐relevant conditions while maintaining mechanical performance comparable to photoiniferter‐derived networks, subsequent studies focused on BAPO‐initiated free radical formulations.

**FIGURE 3:**
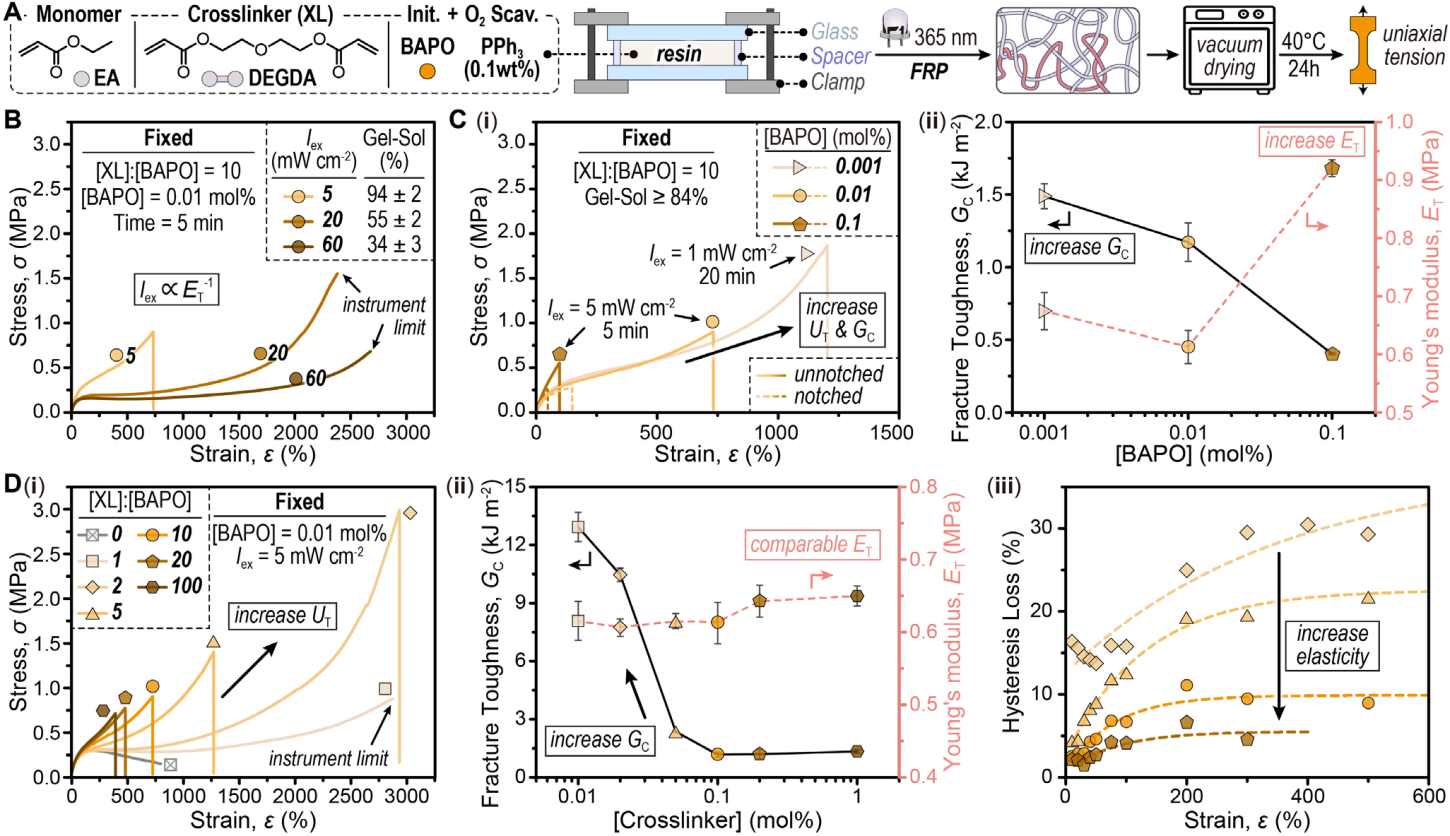
Mechanical properties of poly(EA) tanglemers. (A) Resin formulation, casting setup, and post‐processing for elastomer synthesis. (B) Uniaxial tensile stress–strain curves for elastomers prepared at varying light intensities (365 nm, 5 min irradiation). (C) Effect of initiator concentration. (i) Stress–strain curves for representative conditions with high gel fractions. (ii) Summary of fracture toughness (*G*_c_) and Young's modulus (*E*_T_). Data are presented as mean values (*n* ≥ 3) ± SD. (D) Effect of crosslinker concentration (365 nm, 5 mW cm^−^^2^, 5 min irradiation). (i) Stress–strain curves. (ii) Summary of *G_c_* and *E*_T_. Data are presented as mean values (*n* ≥ 3) ± SD. (iii) Hysteresis from stepwise cyclic tensile testing, with dashed lines representing exponential fits included as guides to the eye. All tensile tests were conducted at an initial strain rate of 0.02 s^−^^1^.

The influence of light intensity was first examined to determine how radical flux balances gelation rate, monomer conversion, gel fraction, and mechanical performance in poly(EA) elastomers prepared with 0.01 mol% BAPO and a [XL]:[BAPO] ratio of 10. Mechanical properties were measured using fully dried specimens, for which increasing the light intensity from 5 to 60 mW cm^−^^2^ produced a modest decrease in tensile modulus (*E*_T_ ≈ 600 to 500 kPa), but a pronounced increase in strain softening and elongation at break (ε_*f*_ >2000%) (Figure 3B, Table S24). This behavior is attributed to a reduction in gel fraction, from 94% to 34%, with increasing light intensity, consistent with the lower monomer conversion measured by FTIR. After drying, this lower gel fraction likely corresponds to a network architecture closer to the percolation threshold: sufficiently connected to sustain load, but enriched in elastically ineffective strands, dangling chains, loops, and trapped entanglements that may delocalize stress. Small‐amplitude oscillatory shear (SAOS) time‐sweep photorheology during gelation further supports this interpretation. At a fixed frequency of 10 rad s^−^^1^ and strain amplitude of 1%, increasing light intensity decreased the gelation time, defined by the crossover of the storage modulus (*G*′) and loss modulus (*G*′′), from 43 to 22 s, while reducing the plateau *G*′ from 18.7 to 0.6 kPa. Altogether, these observations identify conditions well suited for DLP 3D printing, where low light intensities maximize monomer conversion (Figure S40) while still enabling rapid gelation and the formation of soft, extensible, and tough networks.

The influence of BAPO concentration was examined while holding [XL]:BAPO = 10, using specimens prepared under conditions that produced gel fractions ≥ 85% to isolate the role of initiator loading (Figure 3C). Notably, achieving high gel fractions required different irradiation parameters: resins with 0.1 and 0.01 mol% BAPO were cured at 5 mW cm^−^^2^ for 5 min, whereas resins containing 0.001 mol% BAPO required a lower irradiation intensity and longer exposure times (1 mW cm^−^^2^, 20 min) (Figures S41 and S42, Table S25–S27). Under these conditions, decreasing the BAPO and crosslinker concentrations markedly increased elongation at break (ε_*f*_ ≈ 100% to 1300%), tensile strength (σ_*m*_ ≈ 0.6 to 2 MPa), and modulus of toughness (*U_t_* ≈ 0.3 to 12 MJ m^−^^3^), while maintaining *E*_T_ ≤ 1 MPa (Figure 3C‐i). These mechanical properties are consistent with a network architecture closer to the percolation threshold, one that is sufficiently connected to sustain load, but less densely crosslinked and therefore better able to redistribute stress through elastically active strands, topological defects, and physically trapped entanglements.

Single‐edge notch tests supported this interpretation, revealing an increase in fracture toughness (*G_c_*) from 0.4 to 1.5 kJ m^−^^2^ as the BAPO concentration decreased, alongside a modest reduction in *E*_T_, from ∼1 MPa (0.1 mol%) to ∼0.6–0.7 MPa (0.01–0.001 mol%) (Figure 3C‐ii). Additional experiments at fixed BAPO loading further confirmed this balance between gelation time, network percolation, and entanglement‐mediated toughening (Figures S42–S44, Tables S26–S29). Although 0.001 mol% BAPO produced the highest toughness, its long exposure time is less compatible with practical layer‐by‐layer printing. Thus, 0.01 mol% BAPO irradiated at 5 mW cm^−^^2^ was selected as the preferred DLP‐relevant condition, balancing gelation timescale, high gel fraction, sub‐MPa stiffness, and high toughness. Overall, these results demonstrate that this photopolymerization process decouples stiffness from toughness in poly(EA) elastomers, while also highlighting the processing tradeoff between printing speed and entanglement‐mediated mechanical performance.

The role of covalent crosslink density in stabilizing entangled poly(EA) networks was next examined by varying the [XL]:[BAPO] ratio from 1 to 100 under the optimized initiator and irradiation conditions (0.01 mol% BAPO, 5 mW cm^−^^2^), along with a control sample without crosslinker (Figure 3D, Table S30). Based on feed stoichiometry, the preferred formulation containing 0.1 mol% DEGDA (XL) is expected to contain multiple entanglements per covalent network strand, as the nominal crosslinker spacing of ∼50 kg mol^−^^1^ is approximately five times the reported entanglement molecular weight of poly(EA) (∼12 kg mol^−^^1^) [26]. Mechanically, such networks are expected to fall between a conventional chemically crosslinked rubber, where increasing crosslinker concentration would increase *E*_T_, and an uncrosslinked entangled melt, where chains ultimately relax by disentanglement at long times. The present materials exhibit features of both limits. SAOS rheology showed a low‐frequency rubbery plateau with no terminal *G*′/*G*″ crossover within the accessible frequency window (Figure S45), indicating solid‐like behavior stabilized by covalent crosslinks. However, *E*_T_ remained nearly independent of crosslinker concentration (∼600–650 kPa), and using *M*_app_ ≈ 3*ρRT*/*E*_T_, where *ρ* is the polymer density (1.1 g cm^−^^3^), *R* is the universal gas constant, *T* is the absolute temperature, and *E*_T_ is the tensile modulus, gave an apparent molecular weight between elastically active constraints of *M*_app_ ≈ 12.7 kg mol^−^^1^, close to the reported *M*_e_ of poly(EA). Together, these results suggest that stiffness is governed primarily by physically trapped entanglements and other topological constraints, while sparse covalent crosslinks prevent terminal flow.

Having established that stiffness is relatively insensitive to crosslinker loading, we next examined how covalent crosslink density affects dissipative mechanical behavior, fracture resistance, and elastic recovery. As the crosslinker content decreased, the materials displayed greater strain softening and energy dissipation, with *U*_T_ reaching a maximum at [XL]:[BAPO] = 2 (Figure 3D‐i). Fracture testing revealed a similar dependence on network connectivity, with *G*_c_ increasing from 1.2 to 12.9 kJ m^−^^2^ as [XL]:[BAPO] decreased from 10 to 1 before plateauing (Figure 3D‐ii), consistent with enhanced energy dissipation in less densely crosslinked, entanglement‐rich networks. Stepwise cyclic tensile testing with increasing maximum strain revealed the complementary effect of crosslinker content on elastic recovery, with hysteresis at large deformations (> 400%) decreasing from > 30% for [XL]:[BAPO] = 2 to < 10% for [XL]:[BAPO] ≥ 10 (Figures 3D‐iii and S46–S48). Thus, reducing crosslink density enhances dissipation and fracture resistance, whereas increasing crosslink density improves elastic recovery, identifying [XL]:[BAPO] = 10 as a balanced formulation that combines softness, elasticity, strength, and toughness.

### 2.3 Poly(AAm) Hydrogels: Photopolymerization and Mechanical Properties

To extend this approach to hydrogels, AAm‐based resins were formulated and evaluated under analogous conditions. Resins comprised AAm as the monomer, selected for its high propagation rate constant *k*_p_ (*k*_p_ = 3.93 × 10^4^ L mol^−1^ s^−1^ at [AAm] = 2.8 M and 20°C) [44] and the low reported entanglement molecular weight of poly(AAm) (*M*_e_ ≈ 9 kg mol^−^^1^, neat; *M*_e_ ≈ 35 kg mol^−1^, water content = 33.6 wt%) [45], features expected to promote rapid chain growth and entanglement formation during gelation. The resins also contained *N, N′*‐methylenebisacrylamide as the crosslinker, and either lithium phenyl‐2, 4, 6‐trimethylbenzoylphosphinate (LAP) as a water‐soluble photoinitiator for free radical polymerization (Figure 4A) or methyl 2‐[(ethoxycarbonothioyl)thio]propanoate (XAN) for photoiniferter polymerization (Figure S49). For these formulations, a high monomer‐to‐water mass ratio of 2:1 was selected to promote entanglement formation. As observed for elastomers, LAP‐initiated polymerization (0.001 mol% relative to AAm) achieved comparable monomer conversion and mechanical properties under milder conditions (365 nm LED, 2 mW cm^−^^2^, 10 min) than those required for photoiniferter polymerization (365 nm LED, 100 mW cm^−^^2^, 10 min) (Figures S50 and S51, Table S31). These results suggest that the mechanical enhancements are more closely associated with conditions that favor high conversion and entanglement formation, rather than the specific radical polymerization mechanism.

**FIGURE 4:**
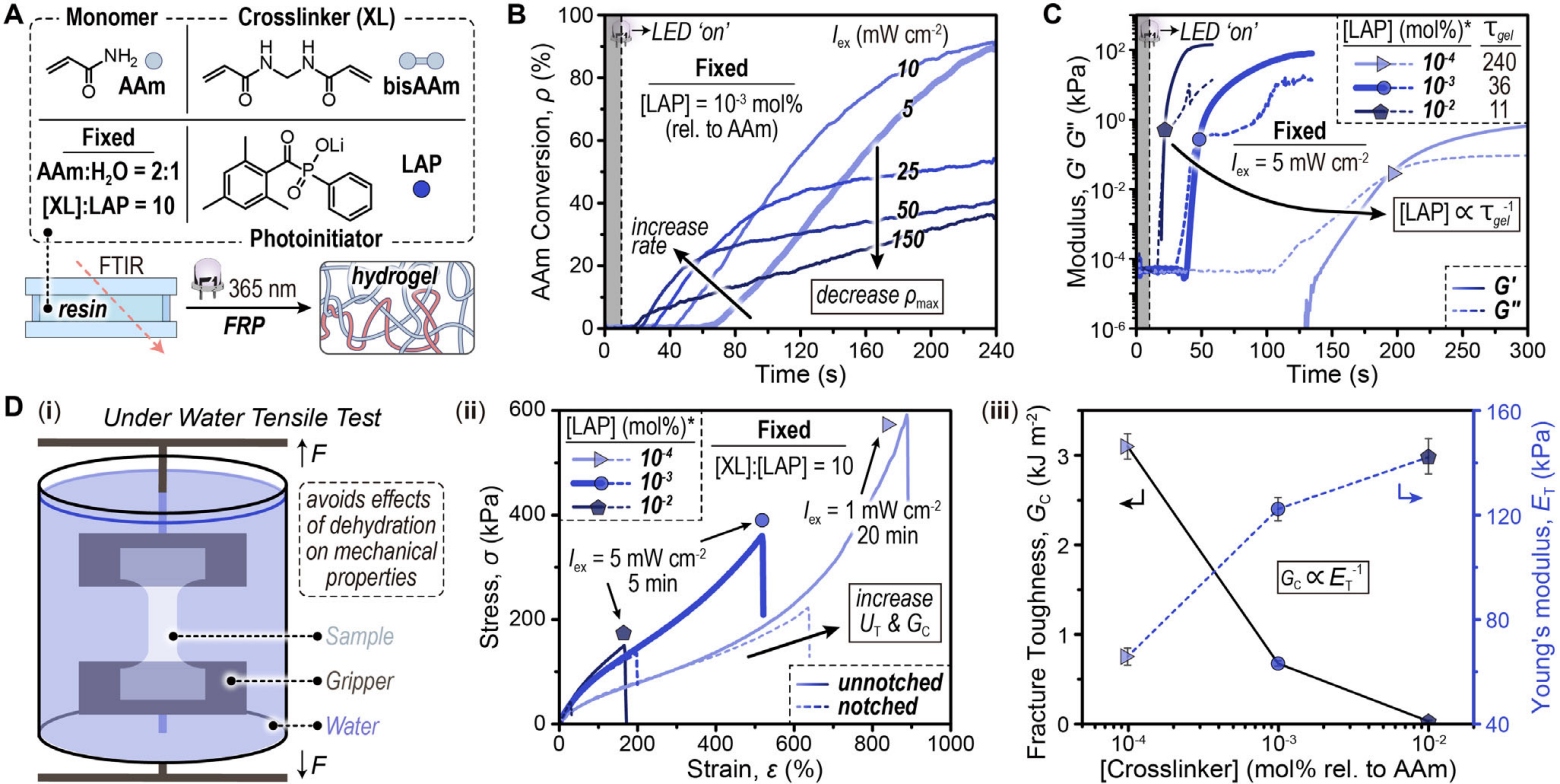
Kinetics and mechanical properties of poly(acrylamide) tanglemers. (A) Components and schematic of hydrogel synthesis. (B) Real‐time photopolymerization kinetics of acrylamide monitored by FTIR spectroscopy. Samples (100 µm thick) were irradiated with a 365 nm LED starting 10 s after the measurement began. (C) Photorheology showing gelation behavior as a function of [LAP]/[AAm]. Samples (100 µm thick) were irradiated with a 365 nm LED starting 10 s after the measurement began. (D) Mechanical characterization of hydrogels. (i) Schematic of underwater uniaxial tensile testing. (ii) Stress–strain curves at varying [LAP]/[AAm] and constant [XL]:[LAP] = 10 with casting conditions indicated. (iii) Summary of fracture toughness (*G*_c_) and tensile modulus (*E*_T_) as a function of LAP concentration. Data are presented as mean values (*n* ≥ 3) ± SD.

The influence of light intensity and initiator concentration on photopolymerization kinetics was next evaluated for LAP‐initiated resins. At fixed [XL]:[LAP] = 10 and 0.001 mol% LAP relative to AAm, increasing light intensity from 5 to 150 mW cm^−^^2^ resulted in behavior analogous to that of the BAPO‐initiated elastomer systems, with a higher initial polymerization rate accompanied by lower final monomer conversion (Figures 4B and S52, Tables S32 and S33). Correspondingly, photorheology of the same resin showed an inverse relationship between light intensity and gel point: the gelation time decreased from approximately 36 to 20 s, while the plateau *G*′ decreased from ∼85 to 3 kPa, consistent with reduced monomer conversion and lower effective density of load‐bearing network strands and trapped entanglements (Figure S53). Instead, decreasing LAP concentration from 0.01 to 0.0001 mol% relative to AAm, at fixed [XL]:[LAP] = 10 and 365 nm LED intensity of 5 mW cm^−^^2^, led to a substantial increase in gel point from 11 to 237 s (Figure 4C). Together, these results identify a processing window that balances high monomer conversion with DLP‐compatible gelation timescales, which was subsequently used to evaluate hydrogel mechanical properties under uniaxial tension.

Poly(AAm) networks were prepared for mechanical characterization using the same casting protocol described for elastomers (Section 2.2), followed by equilibration in water for 48 h prior to testing. At equilibrium, all LAP‐derived hydrogels exhibited swelling ratios of ∼4, corresponding to ≈ 80 wt% H_2_O (Table S34, Equation S6). To avoid dehydration during uniaxial tensile testing (initial strain rate = 1 s^−^^1^), measurements were conducted underwater (Figures 4D‐i and S54). Decreasing initiator concentration from 0.01 to 0.0001 mol%, while irradiating for sufficient time to reach the plateau storage modulus (*G*′) by photorheology (5 min for 0.01 and 0.001 mol%, and 20 min for 0.0001 mol%), reduced the tensile modulus, *E_T_*, from 143 to 66 kPa, with an accompanying increase in water content from 81% to 86%. Together with this decrease in stiffness, the fracture toughness, *G_c_*, increased from 0.03 to 3.10 kJ m^−^^2^ (Figures 4Dii, iii and S55). This unusual increase in toughness, despite a reduction in modulus, is consistent with tanglemer‐like behavior, in which longer chains between crosslinks and physically trapped entanglements are inferred to enhance stress delocalization. However, in contrast to the poly(EA) elastomers, elastically ineffective chains likely play a less prominent role in toughening these poly(AAm) hydrogels because swelling reduces interchain friction. Compared with prior ultrasoft (*E*_T_ <100 kPa) and tough (*G*_c_ >1 kJ m^−^^2^) hydrogel tanglemers that require hours of irradiation [18, 21] or redox‐based curing [25], selected formulations in the present study reach mechanically robust states after ≤ 5 min of irradiation, supporting their translation to DLP printing under the low‐intensity processing windows evaluated below.

### 2.4 DLP 3D Printing and Performance of Elastomers and Hydrogels

Translation of selected resin formulations and processing conditions to DLP 3D printing was next evaluated for both elastomers and hydrogels, with emphasis on identifying processing windows that balance feature fidelity, gelation timescale, and mechanical performance. Feature fidelity was first evaluated using a custom resolution test target, for which exposure time per 100 µm layer was varied locally from 10 to 60 s (365 nm LED, 5 mW cm^−^^2^), followed by optical microscopy. To confine light within the irradiation voxels, Sudan I (0.02 wt%) and tartrazine (0.05 wt%) were incorporated into elastomer and hydrogel resins, respectively (Figures S56–S63, Tables S35–S40). For elastomers cured from resins with 0.01 mol% BAPO and 0.1 mol% crosslinker, sub‐100 µm lateral features were achieved irrespective of UV post‐curing and drying (Figures S64–S70, Table S41), with only a ∼5% decrease in volume observed in the absence of UV post‐curing (Figure S66). Increasing initiator loading to 0.1 mol% enabled faster printing (∼10 s per layer at 5 mW cm^−^^2^), albeit with reduced feature fidelity (∼500 µm), whereas lowering intensity (0.5 mW cm^−^^2^, 40 s per layer) improved feature fidelity to ∼100 µm (Figure S67). Cure depth analysis using Jacob's working curves [46, 47] under these conditions gave a penetration depth (*D*_p_) of ∼170 µm and a critical curing energy (*E*_c_) of ∼250 mJ cm^−^^2^ (Figure S68).

For hydrogels photopolymerized from resins with 0.001 mol% LAP and 0.01 mol% bisacrylamide relative to AAm, minimum lateral feature sizes of ∼100 µm were observed under identical irradiation conditions. Although smaller features were present, they were difficult to resolve by optical microscopy prior to swelling; however, they became apparent as ∼70 µm features after swelling, corresponding to an estimated initial feature size of ∼15 µm (Figure S69, Table S42). Cure depth analysis for the hydrogel formulation revealed a larger penetration depth (*D*_p_) of ∼630 µm with a comparable critical energy (*E*_c_) of ∼270 mJ cm^−^^2^ (Figure S70). Collectively, these results establish processing windows that balance print speed and feature fidelity for subsequent mechanical evaluation, while both elastomer and hydrogel resins also exhibited stable shelf life with no apparent changes in polymerization kinetics after storage in the dark at room temperature for at least 14 days (Figures S71 and S72, Tables S43 and S44).

Under selected low‐intensity printing conditions (365 nm LED, 5 mW cm^−^^2^, 30 s per 100 µm layer), elastomers with complex geometries were fabricated, including a 7_7_ knot and gyroid structures comprising continuous soft material along tortuous paths (Figures 5A, S73, and S74). To quantify mechanical performance, dogbone specimens (20% scale ASTM D638 Type IV) were printed, dried under vacuum at 40°C for 24 h to remove unreacted monomer, and subjected to uniaxial tensile testing. Gel fractions of 77 and 87% were measured for as‐printed (green‐body) specimens prepared at 30 and 60 s exposures per 100 µm layer, respectively. After drying, the printed elastomers were soft (*E*_T_ ≈ 0.8 MPa), highly extensible (ε_f_ ≈ 800–900%), and tough (*U*_t_ ≈ 6 and 9 MJ m^−^^3^; *G*_c_ ≈ 0.9 and 0.8 kJ m^−^^2^) (Figure 5B, Table S45). The printed elastomers retained similar mechanical behavior after accelerated weathering using a modified ASTM G154 Cycle 1 protocol consisting of five cycles (72 h total) of 8 h UVA exposure at 60°C and 50% relative humidity, followed by 4 h at 50°C and 50% relative humidity (Figure 5B, dotted line).

**FIGURE 5:**
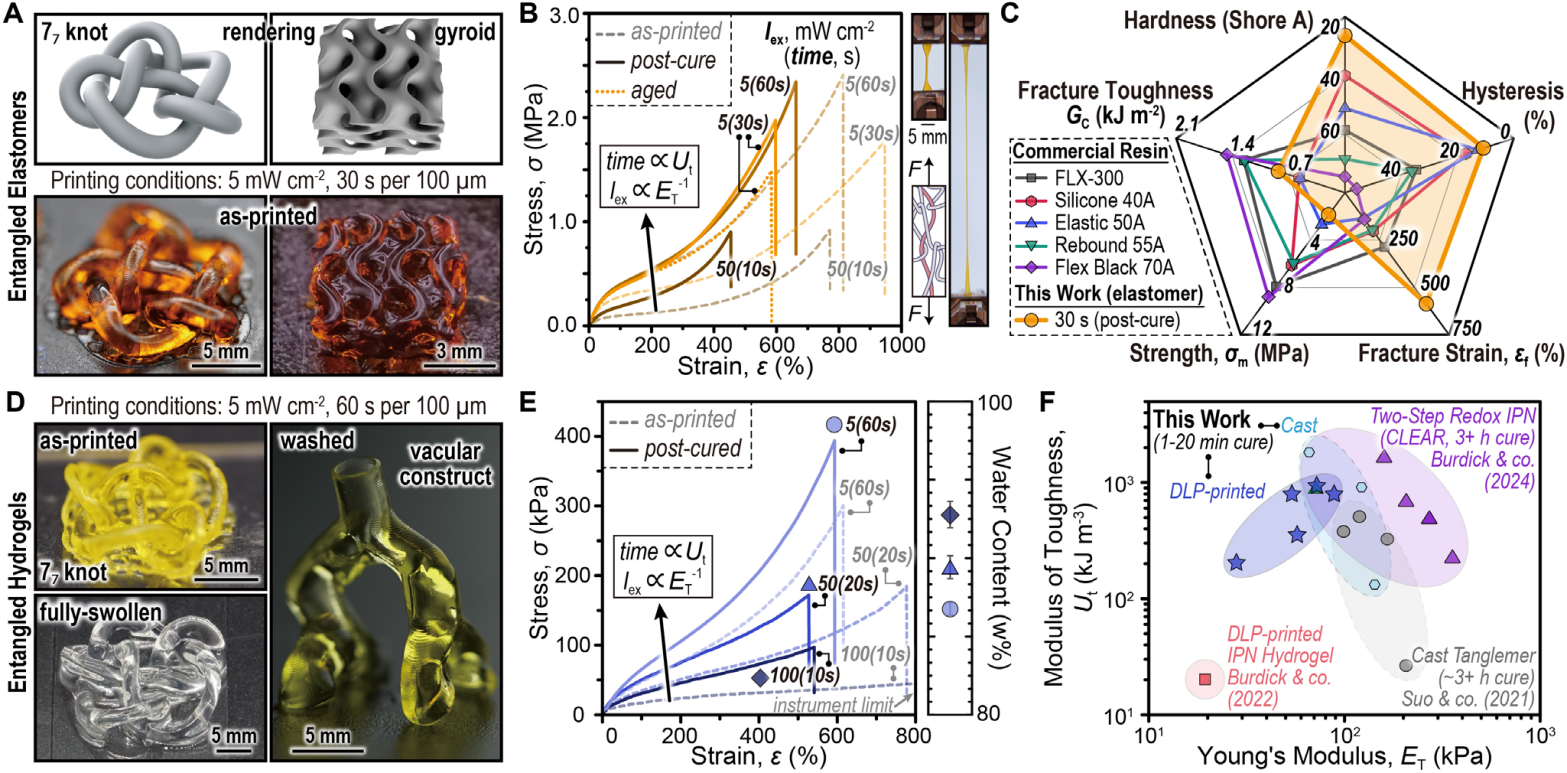
DLP 3D printing and mechanical performance of entangled elastomers and hydrogels. (A) Digital renderings and photographs of as‐printed elastomer structures (7₇ knot and gyroid) fabricated at 5 mW cm^−^^2^ (365 nm, 30 s per 100 µm layer). (B) Uniaxial tensile stress–strain curves of 3D‐printed elastomer dogbone samples at varying printing conditions; samples were dried prior to testing. Accelerated weathering conditions: 5 cycles of ASTM G154 (72 h total). (C) Comparison of mechanical performance with commercial elastomer resins. Data are presented as mean values (*n* ≥ 3). (D) Photographs of hydrogel structures, including as‐printed and fully swollen 7₇ knot and a vascular construct (5 mW cm^−^^2^, 60 s per 100 µm layer). (E) Uniaxial tensile stress–strain curves of 3D‐printed hydrogel dogbone samples at varying printing conditions; samples were tested in the fully swollen state. Data are presented as mean values (*n* ≥ 3) ± SD. (F) Modulus of toughness (*U_t_*) versus Young's modulus (*E_T_*) comparing this work to previously reported 3D‐printed hydrogels [16, 21, 34].

For elastomers printed at 5 mW cm^−^^2^ using 30 or 60 s exposures per 100 µm layer, post‐curing with 5 mW cm^−^^2^ 365 nm light for 10 min before drying increased gel fractions from 77% to 91% and from 87% to 95%, respectively, without significantly altering mechanical properties, apart from a slight shift toward earlier strain‐hardening and reduced strain at failure. These results indicate that post‐curing is not required to access the primary mechanical performance reported here, but can be used to increase gel fraction. Faster layer times were accessible using higher light intensity (50 mW cm^−^^2^, 10 s per layer) or increased initiator loading (0.1 mol% BAPO, 5 mW cm^−^^2^, 15 s per layer), albeit with a modest reduction in mechanical performance, including lower strength. This behavior is consistent with trends observed in cast films, where higher radical fluxes reduce monomer conversion and effective entanglement formation (Figures 5B and S75–S77, Tables S46–S47). These results demonstrate that DLP printing translates the cast‐film structure–property relationships within a defined processing window, while also identifying faster printing without loss of mechanical performance.

To benchmark performance against state‐of‐the‐art materials, five commercially available resins designed for soft and tough applications (FLX300, Amera Labs; Rebound 55A, Tenacious Flex Black 70A, Siraya Tech; Silicone 40A and E50A, Formlabs) were evaluated under the manufacturers' recommended printing, washing, and post‐curing conditions. Dogbone specimens (20% scale ASTM D638 Type IV) were printed and subjected to uniaxial tensile testing. Resolution test patterns printed on our DLP system (405 nm LED, 5 mW cm^−^^2^) identified optimal exposure conditions and revealed faster fabrication speeds (≤ 10 s per 100 µm layer) than the EA‐based resin, but with lower lateral feature fidelity (≥ 100 µm) (Figures S78 and S79, Table S48). Under comparable conditions, the printed poly(EA) elastomers (365 nm LED, 5 mW cm^−^^2^, 30 s per 100 µm layer) exhibited a distinct combination of softness, elasticity, and extensibility relative to commercial resins (Figures 5C and S80–S87, Table S49–S50), including lower hardness (Shore A ≈ 26 vs. ∼40–70), reduced hysteresis loss (∼10% vs. 12%–56% from step‐cyclic loads), and higher elongation at break (∼600% vs. ∼130%–300%). Toughness was comparable but intermediate, with *U*_t_ ≈ 5 MJ m^−^^3^ and *G_c_* ≈ 0.8 kJ m^−^^2^ relative to ∼2–8 MJ m^−^^3^ and 0.6–1.5 kJ m^−^^2^ for commercial resins, while tensile strength was lower (σ_m_ ≈ 1.9 MPa vs. ∼2.7–8.8 MPa). Together, these results show that entangled poly(EA) elastomers access a complementary mechanical performance space relative to commercial soft DLP resins, prioritizing softness, elasticity, and extensibility while maintaining competitive toughness under the selected low‐intensity processing conditions.

In analogy to the elastomers, hydrogels were fabricated in complex geometries under selected low‐intensity conditions, including a 7_7_ knot and a vascular model (365 nm LED, 5 mW cm^−^^2^, 60 s per 100 µm layer) (Figure 5D, Figures S88–S91). Mechanical properties were evaluated using dogbone specimens (20% scale ASTM D638 Type IV) printed across a range of light intensities and exposure times, with and without post‐curing (365 nm LED, 5 mW cm^−^^2^, 10 min) prior to equilibration in water, revealing a tradeoff between fabrication speed and mechanical performance. Post‐curing produced a modest increase in stiffness and strength, accompanied by reduced elongation at break, while increasing light intensity from 5 to 50 and 100 mW cm^−^^2^ enabled faster printing (60 to 20 and 10 s per layer, respectively), albeit with reduced strength and toughness (Figure 5E, Figure S92, Table S51). For post‐cured parts, increasing light intensity decreased Young's modulus from ∼70 to 30 kPa and increased water content from ∼87% to 93%, consistent with reduced monomer conversion and lower effective entanglement density.

Benchmarking against prior reports places these materials in a competitive performance regime, as under optimized conditions (365 nm LED, 5 mW cm^−^^2^, 60 s per 100 µm layer), the printed hydrogels exhibited *E*_T_ ≈ 70 kPa and *U*_t_ ≈ 1000 kJ m^−^^3^, comparable to cast hydrogel tanglemers (*E_T_* ≈ 100–200 kPa, *U_t_* ≈ 20–600 kJ m^−^^3^) [18] and CLEAR‐printed hydrogels (*E_T_* ≈ 50–290 kPa, *U_t_* ≈ 150–1650 kJ m^−^^3^) [25], representing a significant advance over other DLP‐printed interpenetrating network hydrogels [48] (Figures 5F and S93–S94, Table S52). Overall, these results demonstrate that simple free radical polymerization formulations, operated under low‐intensity irradiation, enable DLP 3D printing of mechanically robust hydrogels with performance comparable to more complex multistage state‐of‐the‐art systems.

## 3 Concluding Remarks

Herein, we demonstrate a simple strategy to produce highly entangled polymer networks (“tanglemers”) through free radical photopolymerization, with translation from casting to DLP 3D printing. Although controlled polymerizations via efficient photoiniferter mechanisms were systematically investigated, uncontrolled free radical polymerization at low photoinitiator loadings and light intensities produced ultrahigh molecular weight chains with orders‐of‐magnitude faster apparent polymerization rates, giving rise to tanglemer architectures that decouple stiffness from toughness. This approach provides elastomers and hydrogels that simultaneously combine softness, elasticity, strength, and toughness, with elastomers exhibiting *E*_T_ ≤ 1 MPa, ε_f_ > 500%, σ_m_ ≈ 2 MPa, and *G*_c_ ≈ 1 kJ m^−^^2^, and hydrogels exhibiting *E_T_* ≈ 50 kPa and *U_t_* ≈ 1 MJ m^−^^3^. Importantly, the structure–property relationships established in cast films were preserved during DLP printing, enabling tunable mechanical performance through straightforward control of resin composition and processing parameters, such as light intensity and exposure time. Benchmarking against commercial resins and prior reports highlighted that these materials occupy a complementary mechanical performance space, particularly for elastomers, which combine low modulus (<30 Shore A), low hysteresis (∼10%), and high extensibility (>500%), competitive toughness (*G*_c_ ≈ 1 kJ m^−^^2^), and retention of mechanical performance after initial accelerated weathering.

Beyond mechanical performance, the present approach is potentially translational because the resins are derived primarily from industrially abundant acrylic and AAm monomers rather than the specialty oligomers or multicomponent curing systems commonly used in high‐performance soft DLP resins. The use of sub‐mol% acylphosphine oxide photoinitiator loadings, together with near‐complete initiator consumption during polymerization, represents a favorable formulation feature for reducing potential residual photoinitiator leachables. However, cytotoxicity studies that consider residual monomer and initiator are necessary next steps. Despite these advances, several opportunities remain to further improve performance and expand the scope of this platform. From a manufacturing perspective, increasing printing speed without compromising entanglement density and mechanical properties remains a central challenge of the present approach, as higher radical flux currently leads to reduced monomer conversion and diminished mechanical performance. Strategies to address this limitation may include the use of more efficient photoinitiators or chain‐transfer agents, as well as dynamic control of light intensity during printing to maintain high polymerization rates while mitigating termination pathways that limit molecular weight and entanglement density. Additionally, extending this approach beyond EA and AAm to a broader range of monomers, particularly those with low entanglement molecular weights, will further expand the applicability of this strategy across diverse material classes. Looking forward, these results establish a foundation for DLP‐printable, defect‐tolerant soft materials based on simple and scalable formulations, while also defining the key processing, validation, and application‐specific studies needed to advance this platform toward soft robotics, wearable electronics, and biomedical technologies.

## Author Contributions

**Yutong Liu**: conceptualization, methodology, data curation, investigation, validation, formal analysis, visualization, writing – original draft, writing – review and editing. **Tiffany N. Jeng**: methodology, data curation, investigation, validation, formal analysis, writing – review and editing. **Elizabeth A. Recker**: methodology, data curation, investigation, validation, formal analysis, writing – review and editing. **Ellen J. Forehand**: methodology, data curation, investigation, validation, formal analysis, writing – review and editing. **Gabriel E. Sanoja**: methodology, supervision, funding acquisition, project administration, resources, writing – review and editing. **Zachariah A. Page**: conceptualization, methodology, supervision, funding acquisition, visualization, project administration, resources, writing – original draft, writing – review and editing.

## Conflicts of Interest

The authors declare no conflicts of interest.

## Supporting information




**Supporting File**: adma74661‐sup‐0001‐SuppMat.pdf.

## Data Availability

The datasets supporting the findings of this study are publicly available in the Texas Data Repository: https://dataverse.tdl.org/dataverse/Tanglemers. • 3D Printing Files: https://doi.org/10.18738/T8/WMN3MQ • Replication Data for Rheology: https://doi.org/10.18738/T8/SJHBBE • Replication Data for Tensile Testing: https://doi.org/10.18738/T8/TTWEUG • Data for GPC: https://doi.org/10.18738/T8/VW6MN1 • Replication Data for FTIR: https://doi.org/10.18738/T8/ORDQX3
