# Supplementary material for: Soft Yet Tough Tanglemers Through Rapid Photopolymerization for DLP 3D Printing
Source: Adv Mater. 2026 Aug 19;38(58):e74661. doi: 10.1002/adma.74661 (PMC13674616; doi:10.1002/adma.74661)
Supplement: Supplementary file 1 [file ADMA-38-e74661-s001.pdf]

Supporting Information

Soft yet Tough Tanglemers through Rapid Photopolymerization for DLP 3D Printing

Authors: Yutong Liu[1], Tiffany N. Jeng[2], Elizabeth A. Recker[2], Ellen J. Forehand[1], Gabriel E. Sanoja[2]*, and Zachariah A. Page[12]*

[1]Department of Chemistry, The University of Texas at Austin; Austin, TX 78712, USA
[2]McKetta Department of Chemical Engineering, The University of Texas at Austin; Austin, TX, 78712, USA

*Corresponding author. Email: gesanoja@che.utexas.edu, zpage@utexas.edu

This PDF file includes:

Methods
Supplementary Text
Figures S1 to S94
Tables S1 to S52

Table of Contents

S1 Experimental Details S3

S1.1 Materials S3

S1.2 Methods S3

S1.3 Synthesis S9

S2 Supplementary Text S14

S2.1 ^{1}H and ^{13}C NMR and mass spectra S14

S2.2 Influence of PPh_3 ..... S21

S2.3 Influence of Monomer Inhibitors S24

S2.4 Kinetics of EA polymerization with different CTAs S26

S2.5 Kinetics of EA polymerization with conventional free radical initiator S32

S2.6 Casted EA elastomers Film S40

S2.7 Mechanical properties of EA elastomers prepared with photoiniferter S42

S2.8 Mechanical properties of EA elastomers prepared with free radical polymerization S43

S2.9 Mechanical properties of hydrogels prepared via photoiniferter vs free-radical S51

S2.10 Kinetics of acrylamide polymerization S52

S2.11 Mechanical properties of highly entangled hydrogels S55

S2.12 Influence of opaquing agent on photopolymerization kinetics S57

S2.13 Photo-rheology of resins for 3D Printing S62

S2.14 Resolution prints of highly entangled elastomers S64

S2.15 Cure depth of highly entangled elastomer resin S66

S2.16 Resolution prints of highly entangled hydrogels S68

S2.17 Cure depth of highly entangled hydrogels resins S69

S2.18 Shelf life of the 3D printing resin S70

S2.19 Additional 3D printed structures S72

S2.20 Mechanical properties of 3D printed highly entangled elastomers S73

S2.21 Resolution prints of commercial resins S76

S2.22 Mechanical properties of materials prepared from commercial resins S78

S2.23 Weathering tests of 3D prints S82

S2.24 Additional photos of 3D prints S84

S2.25 Mechanical properties of 3D printed highly entangled hydrogels S86

S3 References S89

S1 Experimental Details

S1.1 Materials

Chemicals. All reagents were used as received unless otherwise noted. ACS Certified solvents were used as received from Fisher Scientific unless otherwise noted. Acrylamide (>98%) and ethyl acrylate (>99.0%, stabilized with MEHQ), Acid Yellow 23 (Tartrazine) (>98.0%), 2,2-Dimethoxy-2-phenylacetophenone (DMPA) (>98.0%) were purchased from TCI. Di(ethylene glycol) diacrylate (technical grade, 75%), Sudan I (Dye content ≥95 %), Methyl 2-bromopropionate (98%), *N*,*N*'-Methylenebisacrylamide (99%), 4-methoxyphenol (99%) were purchased from Sigma-Aldrich. Lithium phenyl-2,4,6-trimethylbenzoylphosphinate (97%), phenylbis(2,4,6-trimethylbenzoyl)phosphine oxide (99%), *O*-(5,6,7,8-Tetrahydronaphthalen-2-yl) carbonochloridothioate (98%), 6-Methoxy-3-methyl-1H-indazole (97%), *O*-phenyl carbonochloridothioate (95%) were purchased from Ambeed, Inc. Triphenylphosphine (≥ 99%) was purchased from Chem-impex. $CDCl_3$ (D, 99.8%) were purchased from Cambridge Isotope Laboratories. Ethyl acrylate (97%) and di(ethylene glycol) diacrylate (technical grade, 75%) were passed through basic alumina Al_2O_3 column before use unless otherwise noted.

Commercial 3D Printing Resin: FLX-300 was purchased from AmeraLabs; Silicone 40A and Elastic 40A were purchased from Formlabs; Rebound 55A and Flex Black 70A were purchased from Siraya Tech.

Methyl 2-[(Ethoxycarbonothioyl)thio]propionate (XAN) was synthesized according to literature procedure.[S1]

Non-Chemicals. Silicone rubber spacer was purchased from McMaster-Carr. Extreme-Temperature Silicone Rubber, Translucent, Plain Back, 1/32" Thick, (#86465K21)

S1.2 Methods

Nuclear Magnetic Resonance (NMR) Spectroscopy. NMR spectra were recorded on a Bruker Prodigy MR 500 MHz spectrometer utilizing $CDCl_3$ as solvents. ^{1}H-NMR were carried out coupled and referenced to the $CDCl_3$ chemical shift at 7.26 ppm. ^{13}C-NMR were carried out decoupled and referenced to the $CDCl_3$ chemical shift at 77.16 ppm.

High Resolution Mass Spectrometry (HRMS): HRMS was performed on Agilent Technologies 6530 Accurate-Mass Q-TOF LC/MS using ESI, and the data was subsequently analyzed using Agilent Mass Hunter Qualitative Analysis Software.

Size Exclusion Chromatography (SEC): Size exclusion chromatography (a.k.a., gel permeation chromatography, GPC) was performed on a TOSOH EcoSEC HLC-8320 GPC equipped with a series of TSKgel SuperHM-H, -M, and -N columns, and refractive index (RI) detector. The instrument was run with HPLC-grade tetrahydrofuran as the eluent, at 0.35 mL/min flow rate at 40 °C. Prior to injection, all samples were fully dissolved in the mobile phase and passed through a 0.45 µm PTFE syringe filter from Aijiren. Molecular weight and molecular weight distributions for the samples were estimated vs. polystyrene standards.

Real-Time Fourier Transform Infrared (RT-FTIR) Spectroscopy (Transmission): Resin formulations were injected between two 1 mm thick glass microscope slides (cat. no. 12-550-A3, Fisher Scientific) separated by ~100 µm polyester plastic shims (cat. no. 9513K66, McMaster-Carr) to maintain a constant sample thickness over the course of the photopolymerization. Each sample was placed in a horizontal transmission accessory (A043-N/Q, Bruker) equipped on the FTIR spectrometer (INVENIO-R, Bruker), which was controlled using OPUS spectroscopy software. Spectra were collected using a liquid nitrogen cooled (LN-MCTMid) detector from 2000 to 7000 cm^{-1} at a rate of 1 scan every 0.13 s. All samples were monitored without degassing at

room temperature. The light exposure was performed using the 365 nm (LCS0365-76-22) or 405 nm LED (LCS0405-50-22) light sources from Mightex Systems. Light was delivered via a 5 mm liquid light guide (LLG5-4H, Thorlabs) with a collimator (LGC-019-022-05-V, Mightex). The emission profiles for each LED (Figure S1) were measured using a calibrated Ocean Insight spectrometer. Irradiation intensity was measured with a ThorLabs PM100D photometer equipped with a silicon-based photodiode power sensor (S120VC and S130C). The functional group conversion upon light exposure was determined by monitoring the disappearance of the peak area centered at ~ 6100 − 6250 cm^{-1} corresponding to the C=C–H stretch and ~ 4450 − 4550 cm^{-1} corresponding to the combination of C=C and =C–H stretch.[S2,3] Each sample was tested in triplicate at the same light intensity. The light was not turned on until 10 seconds after the measurement had started.

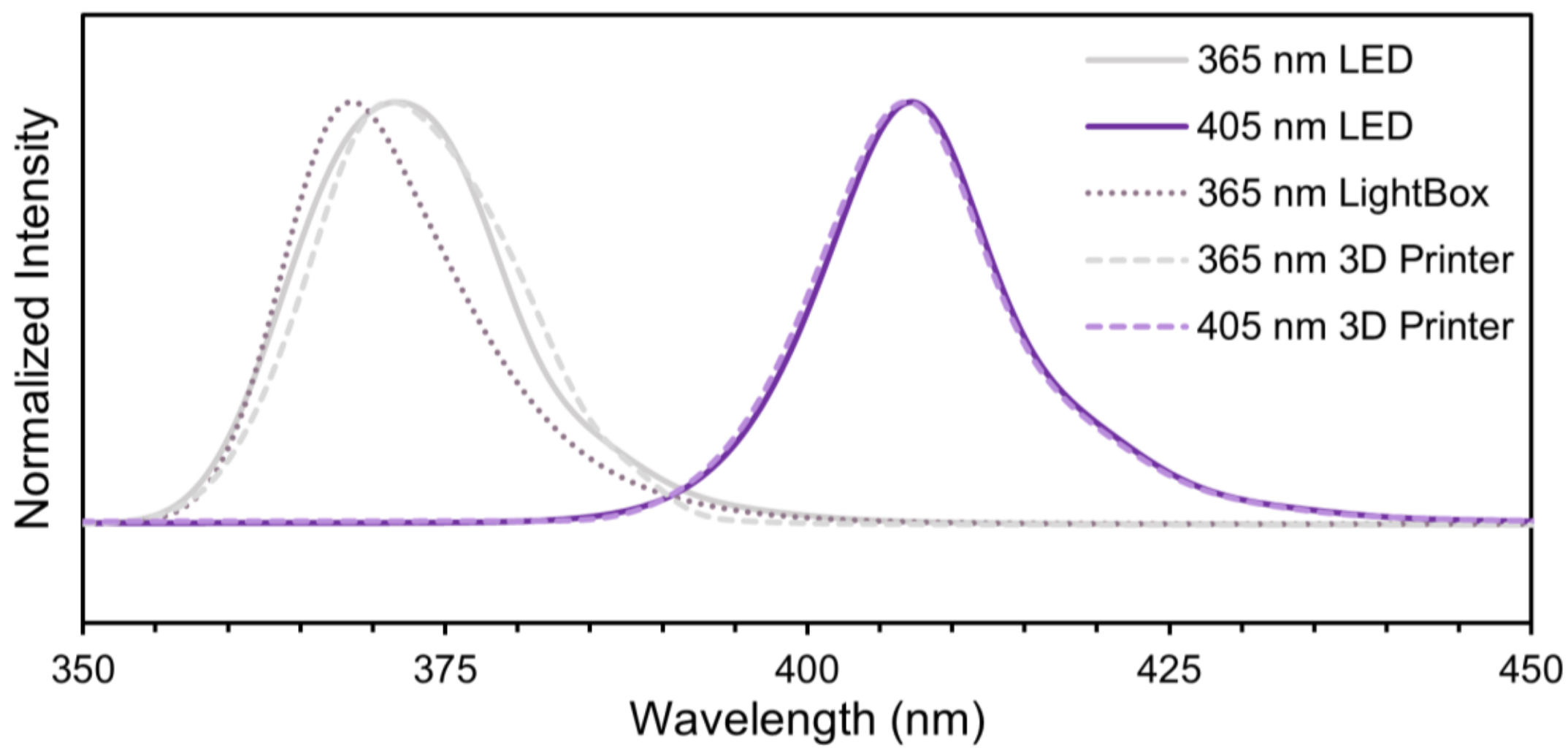

Figure S1. 365 nm and 405 nm LED emission profiles used for FTIR spectroscopy and photo-rheology; 365 nm Lightbox emission profiles used for film casting; 365 nm and 405 nm 3D Printer emission profiles used for 3D Printing.

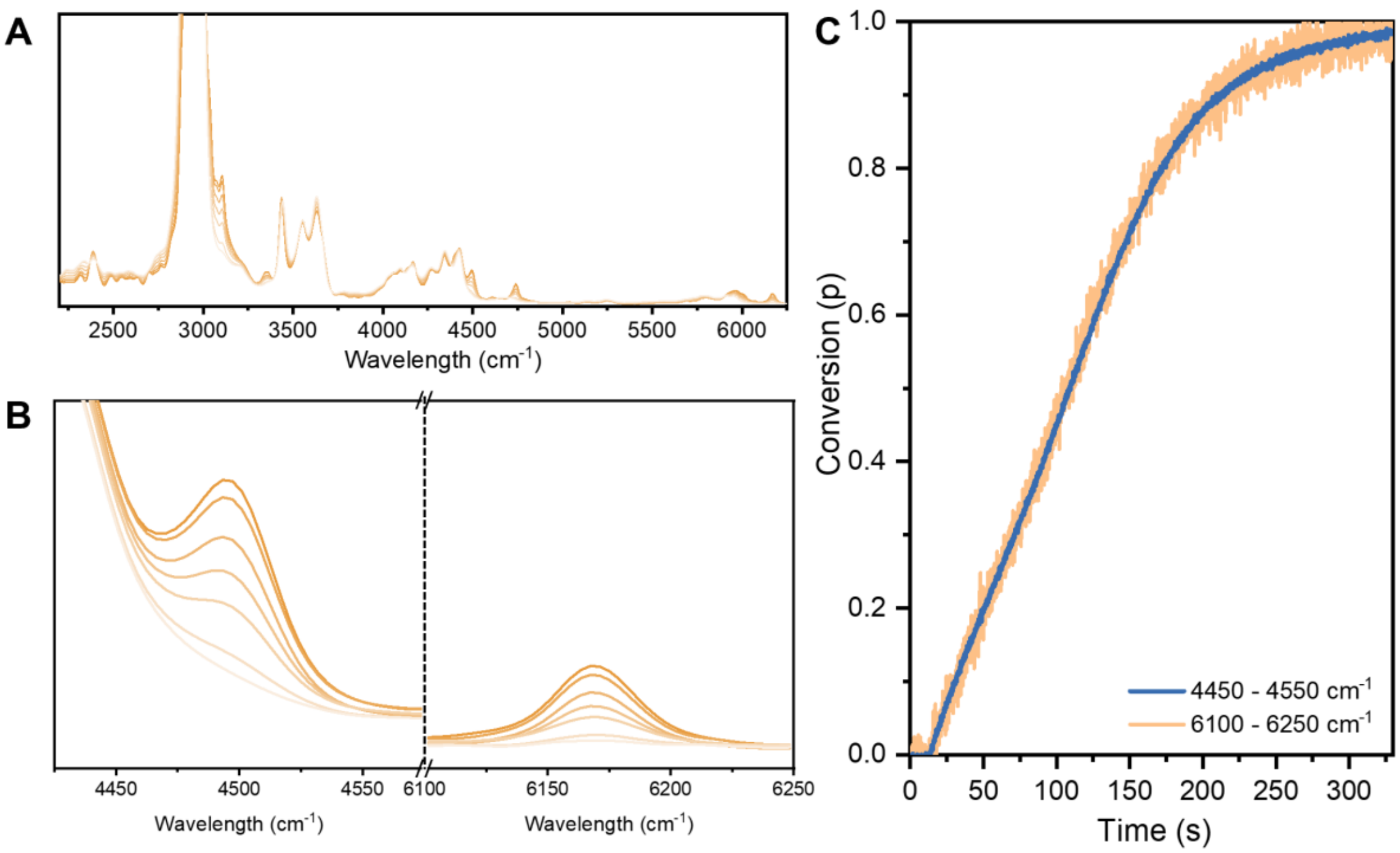

Figure S2. (A) A representative real-time FTIR spectra of ethyl acrylate on glass slides with a 100 µm spacer, along with its magnification (B) to show the C=C and C=C-H combination peak at 4450 - 4550 cm^{-1} and C=C–H stretch overtone at 6100 - 6250 cm^{-1} regions. (C) Overlaid ethyl acrylate conversion as a function of irradiation time when monitoring distinct absorption bands, showing consistency in the results.

Lightbox: Sample irradiation for film casting was performed with a LightBox developed by Monoprinter that contains a 365 nm LED array with a 24 V 350 W internal power supply. The LED power can be intuitive control via the 7-inch touch screen. Light intensity was measured with a ThorLabs PM100D photometer equipped with a silicon-based photodiode power sensor (S120VC and S130C).

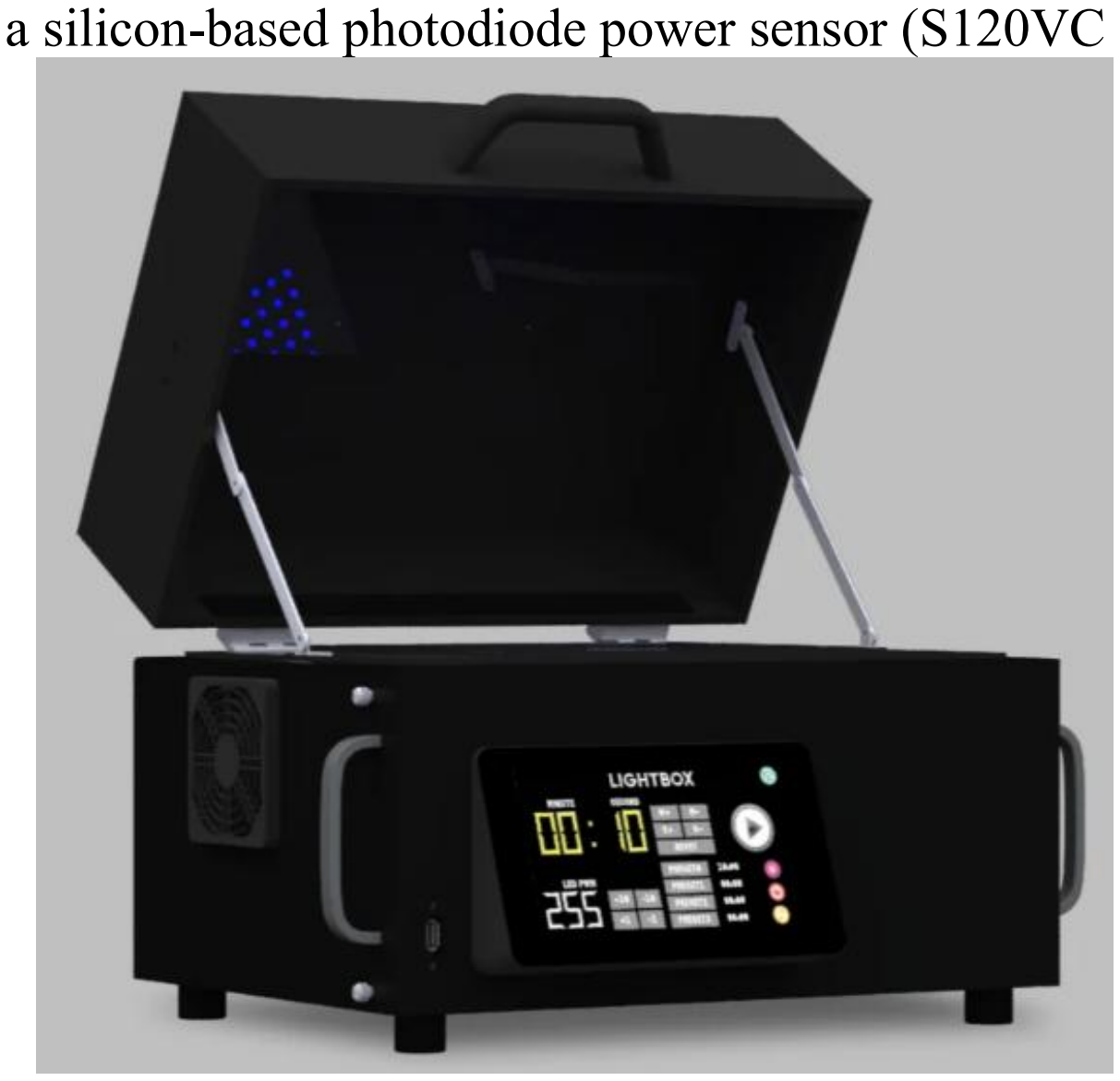

Figure S3. Lightbox from Monoprinter equipped with 365 nm LED.

Rheology: Storage modulus G' and loss modulus G'' measurements of highly entangled elastomers and hydrogels were carried out using a TA instrument Discovery Hybrid Rheometer (HR2). An 8 mm parallel stainless steel plate head (511080.946, TA Instruments) was used with the stainless-steel Smart Swap Peltier Plate Peltier plate base (533210.901, TA Instruments). A strain sweep was conducted at a frequency of 10 rad/s from 0.01 – 10% strain to identify the linear viscoelastic region, followed by a frequency sweep at 0.1% strain from 0.1 – 100 rad/s, at 25 °C.

Photo-Rheology: The time to gelation or "gel point" was estimated from $G' = G''$ using a TA instrument Discovery Hybrid Rheometer (HR2) equipped with a "UV Light Guide" accessory (part # 546301.901, TA Instruments), a 20 mm diameter quartz bottom plate (403153.001, TA Instruments), and a 20 mm diameter geometry stainless steel upper parallel plate (534519.941, TA Instruments). A 5mm liquid light guide (LLG05-4H, Thorlabs) with a collimator (LGC-019-022-05-V, Mightex) was used to illuminate the samples with 365 nm LED, (LCS0365-76-22, Mightex Systems) connected to a driver from which light intensity could be controlled manually. For a typical photo-rheology measurement, the gap height was set to 100 µm for each experiment to best match the printing conditions. The oscillation time sweep was used as the procedure with 1% strain and 10 rad/s frequency. The light intensity of the 365 nm LED was set to the desired value as measured at the top surface of the quartz plate using a silicon photometer (Thorlabs, Model: S401C). The light was not turned on until 10 seconds into the oscillation time sweep step. The crossover of G' and G'' (gel point) was determined using the "moduli cross" data analysis function in TRIOS software (TA Iinstruments).

Uniaxial Tensile Stress-Strain Measurements (Elastomers). For elastomers, uniaxial tensile testing was performed using a Shimadzu AGS-X universal testing machine equipped with a 100 N load cell. Cast samples were cut using an ASTM Standard D-1708 punch (width = 5 mm, gauge length = 20 mm) from films approximately 0.7 – 1 mm in thickness. The uniaxial testing of printed material was performed on ASTM D638 Type IV dogbones that were produced using a projection scaled to 20% of the original size: gauge length = 6.75 mm, width = 1.2 mm, thickness = 1.0 mm, total length = 23.5 mm samples. Dimensions (length, width, thickness) of each dogbone sample was measured prior to performing tensile testing. Samples were stretched until break at a constant strain rate of 0.02 s^{-1}. The Young's modulus E was estimated as the slope of the stress-strain curve between 1 – 5% strain. The modulus of toughness (total strain energy density, U_t) was determined by integrating the area under the stress-strain curve $U_t = \int_1^{\varepsilon_{max}} \sigma d\varepsilon_{max}$, where σ is stress, $\varepsilon_{max} = \frac{L_{max}-L_0}{L_0}$ is stain at break.

Uniaxial Tensile Stress-Strain Measurements (Hydrogels). For hydrogels, uniaxial tensile testing was performed using an Instron 34TM5 equipped with a 100 N load cell. Samples were cut using an ASTM Standard D-1708 punch (width = 5 mm, gauge length = 20 mm) from films approximately 1 – 2 mm in thickness. The uniaxial testing of printed material was performed on ASTM D638 Type IV dogbones that were produced using a projection scaled to 20% of the original size: gauge length = 6.75 mm, width = 1.2 mm, thickness = 1.0 mm, total length = 23.5 mm samples. Then the samples were submerged into water to fully swell before testing. Samples were stretched until break at a constant strain rate of 1 s^{-1}. All hydrogel samples were tested in the fully swollen state under water. The Young's modulus E_T was estimated as the slope of the stress-strain curve between 1 – 10% strain. The modulus of toughness (total strain energy density, U_t) was determined by integrating the area under the stress-strain curve $U_t = \int_1^{\varepsilon_{max}} \sigma d\varepsilon_{max}$, where σ is stress, $\varepsilon_{max} = \frac{L_{max}-L_0}{L_0}$ is stain at break.

Stepwise cyclic tensile testing. Stepwise cyclic uniaxial tensile tests were performed using a Shimadzu AGS-X universal testing machine equipped with a 100 N load cell for all elastomers. Cyclic strains of 10, 20, 30, 40, 50, 75, 100, 200, 300, 400, 500, and 1000% were set, with relaxing to 0.005N between cycles. All loading and unloading were under 0.02 s^{-1} strain rate. The hysteresis of each cycle was calculated as the ratio of the difference between the areas under the loading $A_{loading}$ and unloading curves $A_{unloading}$ to the area under the loading curve (**Eq. S1**). The elastic deformation $\epsilon_{elastic}$ and plastic deformation $\epsilon_{plastic}$ was calculated from the recoverable(elastic) strain $\varepsilon_{elastic}$ and (quasi-)permanent strain $\varepsilon_{plastic}$ based on the literature (**Eq. S2-3**).[S4,5]

Stepwise cyclic tensile testing was used to quantify the elastic and plastic deformation components and hysteresis during step-cyclic tensile testing.

Hysteresis calculation:

$$Hysteresis\ (\%) = \frac{A_{loading} - A_{unloading}}{A_{loading}} \times 100\% \qquad \textbf{Eq. S1}$$

True Strain calculation:

$$\epsilon_{true\ strain} = \ln\lambda = \ln(\frac{L_{loading}}{L_0}) = \ln(1+\varepsilon) \qquad \textbf{Eq. S2}$$

$$\epsilon_{true\ strain} = \epsilon_{plastic} + \epsilon_{elastic} \qquad \textbf{Eq. S3}$$

Single-Edge-Notch Crack Propagation. Cast films were punch-cut into 40 mm × 20 mm rectangle specimens (length × width, and ≈1 mm thickness). These were cut with a fresh razor blade to introduce a crack of ≈1 mm length for elastomers and ≈2 mm length for hydrogels and deformed using the same strain rate as uniaxial tensile testing. The resulting stress–stretch curves can be used to estimate the fracture energy, G_c, using Greensmith's approximation[S6], where c_0 is the crack length in the initial state, $\lambda_c = \frac{L}{L_0}$ is the critical stretch for the crack to start propagating, σ is the stress, and $W(\lambda_c)$ is the strain energy density at the fracture point, $W(\lambda_c) = \int_1^{\lambda_c} \sigma d\lambda$.

$$G_c = \frac{6c_0}{\sqrt{\lambda_c}} W(\lambda_c) \qquad \textbf{Eq. S4}$$

Digital Light Processing 3D Printer: A custom digital light processing (DLP) 3D printer from Monoprinter equipped (MONO3-VZ4, monoprinter) with a 365 nm LED was used for all fabrication and operated at a light intensity of 5 mW/cm^2. Detailed information about the printer can be found in our previous reports[S7]. For commercial resins from Formlabs (Silicone 40A and Elastic 50A), a Form 3 SLA printer from Formlabs was used to match their companies reported processing parameters. For other commercial resins, the same custom digital light processing (DLP) 3D printer from Monoprinter equipped with 405 nm LED was used at a light intensity of 5 mW/cm^2. A transparent fluorinated polymer film (Clear Slippery High-Temperature FEP Film, McMaster-Carr, 127 µm thick), coated with a thin layer of RainX water repellent when needed, was used as the base of the resin tank to provide a non-stick surface. For highly entangled hydrogel 3D printing, the printing environment was maintained at 35 °C using a heating chamber to prevent acrylamide crystallization from the resin at high monomer concentrations.

Shore Hardness (Shore A): Shore A hardness measurements were performed with an HT-6510A durometer. Cylindrical samples were 3D printed with 12 mm × 7 mm (diameter × height) dimensions.

Accelerated Weather Testing: Environmental aging experiments were conducted using a Binder KBF-LQC 240-UL climate chamber (Art. No. 9020-0333) under modified ASTM G154 Cycle 1

conditions. Chamber temperature was cycled between 60 and 50 °C while maintaining 50% relative humidity throughout the experiment. The exposure cycle consisted of 8 h UVA irradiation at 60 °C followed by 4 h at 50 °C without irradiation, repeated for the desired number of cycles. UVA irradiation was provided using a BINDER Q1B Synergy Light T8 fluorescent tube (26 mm diameter, 600 mm length) with emission spanning 320–400 nm (UVA) and 400–800 nm (visible). Environmental conditions were continuously monitored and recorded throughout testing.

S1.3 Synthesis

S1.3.1 CTA synthesis

Table S1. Summary of the CTAs used for photoiniferter polymerization of entangled elastomers or hydrogels.

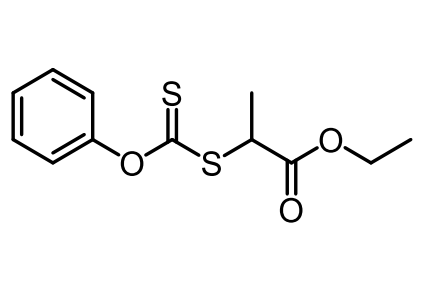	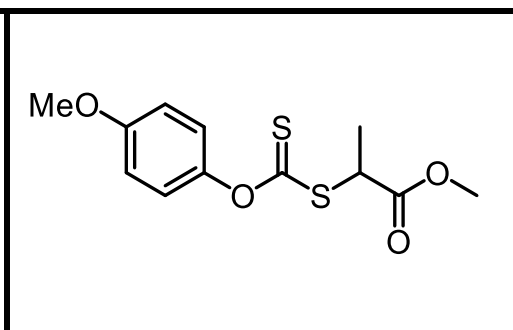	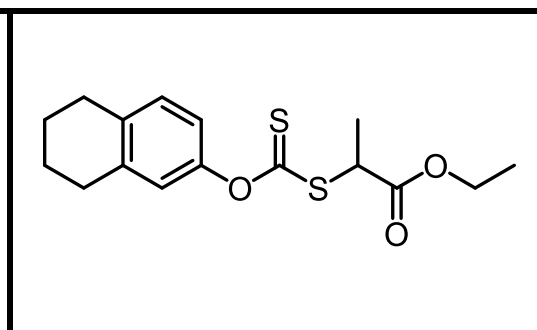	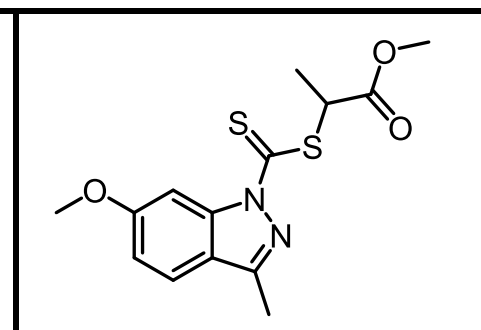	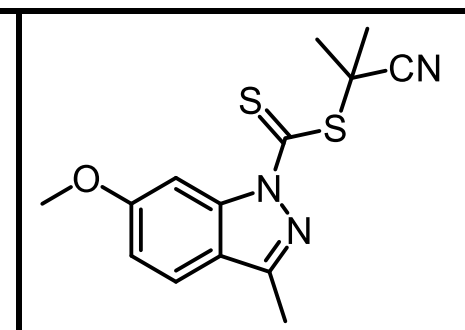
PXEP (CTA 1)	MeOPXEP (CTA 2)	TNXEP (CTA 3)	DTI-ester (CTA 4)	DTI-CN (CTA 5)
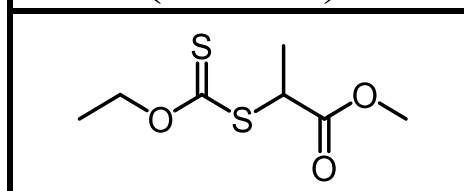				
XAN				

Scheme S1. Synthesis of PXEP (CTA 1)

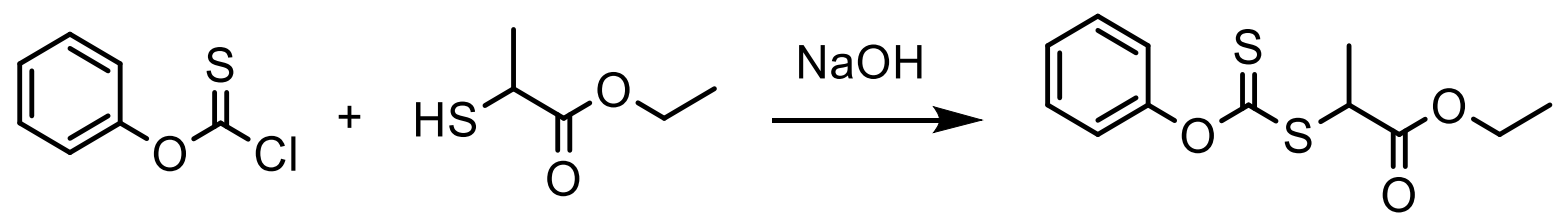

The synthesis of PXEP (CTA 1) was adapted from literature[S8,9]. To a 250 mL round-bottom flask equipped with a magnetic stir bar, sodium hydroxide (0.65 g, 16 mmol, 2 eq.) and 16 mL deionized H_2O were added, followed by cooling the flask in an ice bath. Ethyl 2-mercaptopropanote (1.3 mL, 10 mmol, 1.25 eq.) was added into the reaction mixture dropwise. After addition, the reaction mixture was stirred at room temperature for 30 min. Then, *O*-phenyl carbonochloridothioate (1.1 mL, 8 mmol, 1 eq.) was added dropwise. After addition, the reaction mixture was stirred at room temperature for 3 hours. Then the reaction was diluted with 150 mL water and extracted with 100 mL ethyl acetate three times. The ethyl acetate phases are combined and then extracted with water followed with brine. Then the organic phase was dried over anhydrous $MgSO_4$ and concentrated under reduce pressure. The crude product was purified via silica column chromatography using hexanes:dichloromethane = 2:1 as the mobile phase, isolating the desired band and concentrating it under reduced pressure to afford the product as a yellow liquid (865 mg, 40%).

^{1}H NMR (500 MHz, Chloroform-d) δ 7.42 (m, 2H), 7.30 (m, 1H), 7.09 (m, 2H), 4.49 (m, 1H), 4.25 (m, 2H), 1.67 (d, 3H),1.30 (t, 3H). ^{13}C NMR (125 MHz, Chloroform-d) δ 212.24, 171.29, 154.45, 129.72, 126.89, 122.19, 62.09, 48.68, 17.07, 14.29. ESI-HRMS: m/z = 293.0281 ([M+Na]$^+$), (calcd. 293.0277).

Scheme S2. Synthesis of MeOPXEP (CTA 2)

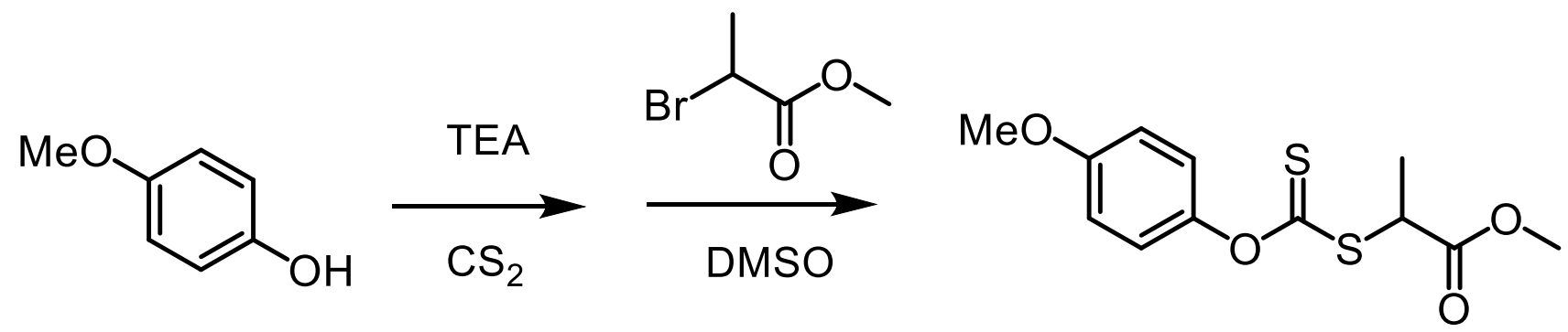

To a 100 mL round-bottom flask equipped with a magnetic stir bar, 4-methoxyphenol (2.5 g, 20 mmol, 1 eq.) and 30 mL of carbon disulfide were added. Triethylamine (2.3 mL, 20 mmol, 1 eq.) was added into the reaction mixture dropwise. After addition, the reaction mixture was stirred for 12 hours at 40 °C. Then, methyl 2-bromopropionate (2.8 mL, 20 mmol, 1 eq.) in 20 mL of dimethyl sulfoxide (DMSO) was added dropwise to the reaction mixture. The reaction mixture was stirred at room temperature for 12 hours at 40 °C. The crude product was purified via silica column chromatography using hexanes:dichloromethane = 3:1 as the mobile phase, isolating the desired band and concentrating it under reduced pressure to afford the desired product as a yellow liquid (687 mg, 12%).

^{1}H NMR (500 MHz, Chloroform-d) δ 6.99 (dd, 2H), 6.93 (dd, 2H), 4.51 (m, 1H), 3.81 (m, 3H), 3.78 (m, 3H), 1.66 (d, 3H). ^{13}C NMR (125 MHz, Chloroform-d) δ 212.73, 171.83, 158.04, 147.99, 122.86, 114.67, 55.72, 53.05, 48.45, 17.09. ESI-HRMS: m/z = 309.0230 ([M+Na]$^{+}$), (calcd. 309.0226).

Scheme S3. Synthesis of TNXEP (CTA 3)

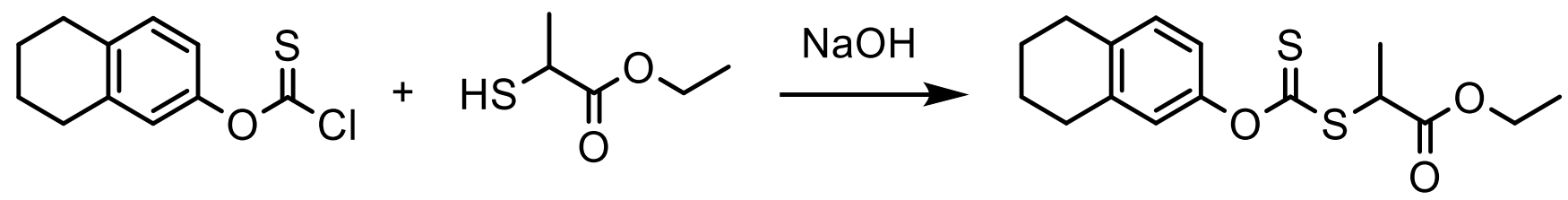

The synthesis of TNXEP was adapted from literature[S8]. To a 250 mL round-bottom flask equipped with a magnetic stir bar, sodium hydroxide (0.67 g, 16.8 mmol, 1.86 eq.) and 30 mL of deionized H_2O were added. Ethyl 2-mercaptopropanote (1.46 mL, 11.25 mmol, 1.25 eq.) was added into reaction mixture dropwise. After addition, the reaction mixture was stirred at room temperature for 30 min. Then, *O*-(5,6,7,8-Tetrahydronaphthalen-2-yl) carbonochloridothioate (2.04 g, 9 mmol, 1 eq.) was added dropwise. After addition, the reaction mixture was stirred at room temperature for 12 hours. Then the reaction was diluted with 150 mL of water and extracted with 100 mL of ethyl acetate three times. The ethyl acetate phases were combined and then extracted with water followed with brine. Then the organic phase was dried over anhydrous $MgSO_4$ and concentrated under reduce pressure. The crude product was purified via silica column chromatography using hexanes:dichloromethane = 2:1 as the mobile phase, isolating the desired band and concentrating it under reduced pressure to afford the product as a yellow liquid (724 mg, 25%).

^{1}H NMR (500 MHz, Chloroform-d) δ 7.08 (d, 1H), 6.82-6.78 (m, 2H), 4.48 (m, 1H), 4.24 (m, 2H), 2.76 (m, 4H), 1.79 (m, 4H), 1.66 (d, 3H), 1.30 (t, 3H). ^{13}C NMR (125 MHz, Chloroform-d) δ 212.52, 171.33, 152.11, 138.94, 135.85, 130.19, 121.92, 118.97, 62.05, 48.58, 29.56, 29.08, 23.12, 22.89, 17.12, 14.30. ESI-HRMS: m/z = 325.0928 ([M+H]$^{+}$), (calcd. 325.0927).

Scheme S4. Synthesis of DTI-ester (CTA 4)

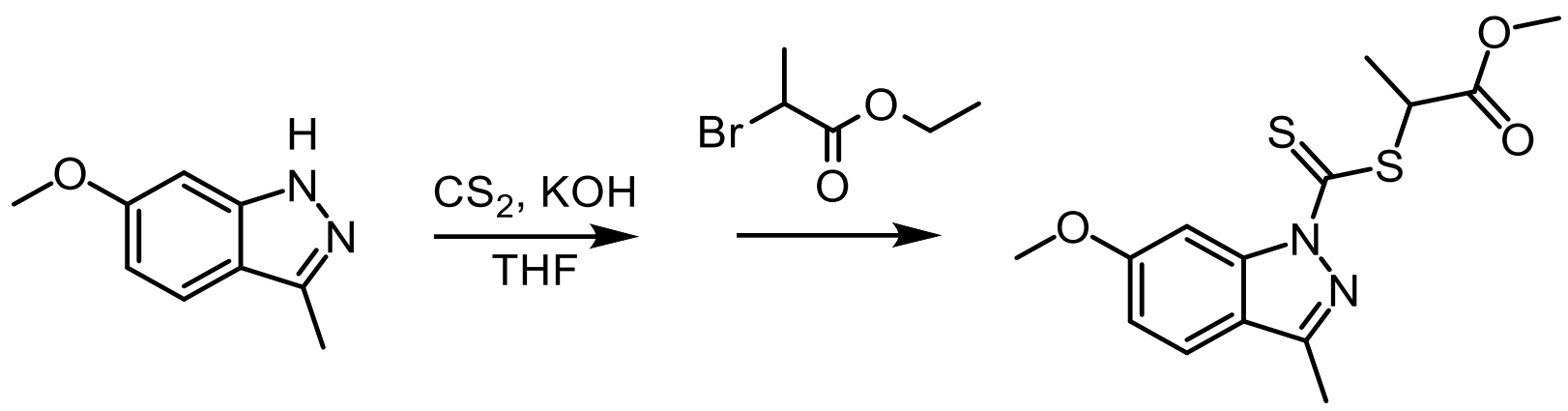

The synthesis of DTI was adapted from literature[S10]. To a 250 mL round-bottom flask equipped with a magnetic stir bar, potassium hydroxide (347 mg, 6.2 mmol, 1 eq.) and 40 mL of anhydrous tetrahydrofuran were added, followed by cooling the flask in an ice bath. 6-Methoxy-3-methyl-1H-indazole (1 g, 6.2 mmol, 1 eq.) in 10 mL anhydrous tetrahydrofuran was added dropwise. After addition, the reaction mixture was stirred for 20 min in an ice bath. Then, carbon disulfide (0.74 mL, 12.3 mmol, 2 eq.) was added dropwise to the reaction mixture that was in an ice bath, followed by stirring while on ice for 10 h. Then methyl 2-bromopropionate (0.83 mL, 7.4 mmol, 1.2 eq.) in 5 mL of anhydrous tetrahydrofuran was added dropwise into the reaction mixture, followed by stirring at room temperature for 10h. The crude product was purified via silica column chromatography using hexanes:ethyl acetate = 1:5 as the mobile phase, isolating the desired band and concentrating it under reduced pressure to afford the product as a yellow solid (262 mg, 13%).

^{1}H NMR (500 MHz, Chloroform-d) δ 8.69 (m, 1H), 7.52 (m, 1H), 7.01 (m, 1H), 4.70 (m, 1H), 3.91 (s, 3H), 3.77 (s, 3H), 2.57 (s, 3H), 1.68 (d, 3H). ^{13}C NMR (125 MHz, Chloroform-d) δ 196.03, 172.56, 162.64, 150.30, 142.76, 122.13, 121.46, 115.62, 100.08, 55.97, 52.91, 46.64, 16.73, 12.11. ESI-HRMS: m/z = 325.0677 ([M+H]$^{+}$), (calcd. 325.0675).

Scheme S5. Synthesis of DTI-CN (CTA 5)

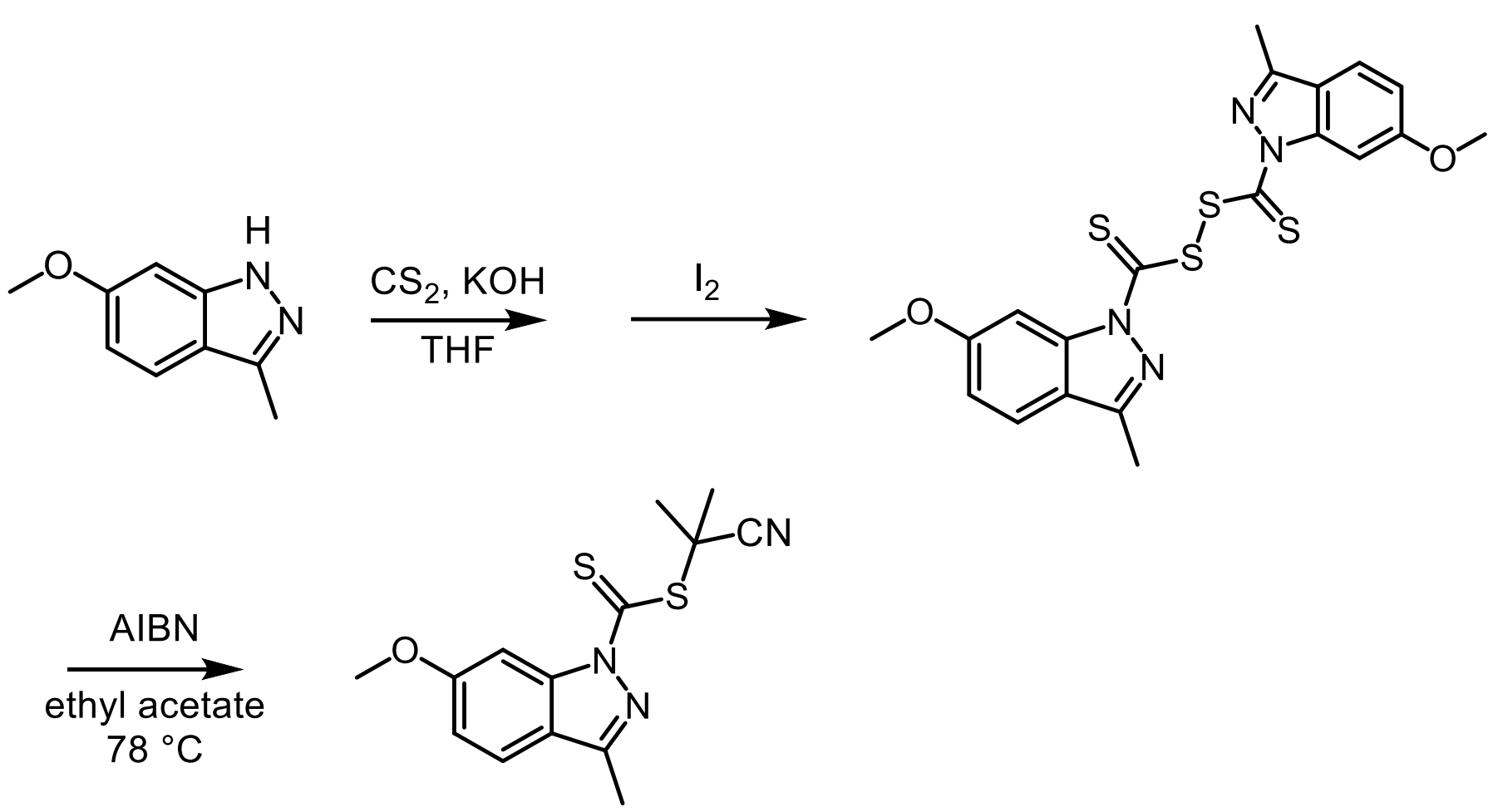

The synthesis of DTI was adapted and modified from literature[S10]. To a 50 mL round-bottom flask equipped with a magnetic stir bar, potassium hydroxide (174 mg, 3.1 mmol, 1 eq.) and 10 mL of anhydrous tetrahydrofuran were added, followed by cooling the flask in an ice bath. 6-Methoxy-3-methyl-1H-indazole (500 mg, 3.1 mmol, 1 eq.) in 10 mL of anhydrous tetrahydrofuran was added dropwise, followed by stirring the reaction mixture for 20 min while remaining on ice. Then, carbon disulfide (0.37 mL, 6.2 mmol, 2 eq.) was added dropwise to the reaction mixture that

was in an ice bath. The reaction mixture was stirred in ice bath for 10 h. The reaction mixture was concentrated under reduced pressure, followed by adding 10 mL of methanol into the flask, then iodine (780 mg, 3.1 mmol, 1 eq.) in 10 mL of methanol was added dropwise. The reaction mixture was then stirred for 1 hour. The resulting yellow solid was filtered and rinsed with 10 mL of diethyl ether three time and then dried under reduced pressure. The product was used for the next step without further purification.

To a 100 mL round bottom flask equipped with a magnetic stir bar and condenser, the product from the previous step (360 mg, 0.76 mmol, 1eq.), 2,2′-azobis(2-methylpropionitrile) (500 mg, 3 mmol, 5eq.), and ethyl acetate (40 mL) was added. The reaction mixture was refluxed at 78 ℃ under nitrogen for ~12 hours. The reaction mixture was then concentrated under reduced pressure and the crude product was purified via silica column chromatography using hexanes:ethyl acetate = 1:5 as the mobile phase, isolating the desired band and concentrating it under reduced pressure to afford the product as a yellow solid (86 mg, 37% second-step, 9% overall).

^{1}H NMR (500 MHz, Chloroform-d) δ 8.67 (m, 1H), 7.52 (m, 1H), 7.01 (m, 1H), 3.91 (s, 3H), 2.54 (s, 3H), 1.97 (s, 6H). ^{13}C NMR (125 MHz, Chloroform-d) δ ^{13}C NMR (126 MHz, CDCl3) δ 191.95, 162.82, 150.10, 142.49, 121.92, 121.55, 121.21, 115.91, 100.03, 56.00, 40.71, 27.17, 12.08. ESI-HRMS: m/z = 306.0732 ($[M+H]^{+}$), (calcd. 306.0729).

S1.3.2 Synthesis of highly entangled elastomer

Table S2. Summary of the resin formulation for highly entangled elastomer synthesis.

Monomer	Crosslinker (XL)	Photo-initiator	Oxygen Scavenger
Ethyl Acrylate (EA)	Diethylene glycol diacrylate	phenylbis(2,4,6-trimethylbenzoyl) phosphine oxide (BAPO)	Triphenylphosphine (PPh_3)

Stock solutions containing the photo-initiator (BAPO) and crosslinker (XL) at varying ratios were prepared in ethyl acrylate, which was preloaded with 0.1 wt% PPh_3. The stocks were then diluted with ethyl acrylate (containing 0.1 wt% PPh_3) to reach the desired BAPO concentration. After thorough mixing, the reaction mixture was injected in between two pieces of glass (**Figure S36**) using a syringe and needle. The silicone rubber (McMaster-Carr, #86465K21) was used as a spacer. And the glass was treated with a thin layer of RainX water repellent to reduce adhesion and facilitate sample isolation from the glass slides after curing. The mold used for casting the reaction mixture was placed on a lightbox (**Figure S3**) equipped with a 365 nm LED panel at the desired light intensity. After irradiation for a specific duration, the film was removed from the glass slides and dried in a vacuum oven at 40 °C for 24 hours to remove unreacted monomer. The sample mass before (m_0) and after reduced-pressure drying (m_{dry}) was recorded to determine the sol-gel fraction:

$$Sol - Gel\ Fraction = \frac{m_{dry}}{m_0} \quad \textbf{Eq. S5}$$

S1.3.3 Synthesis of highly entangled hydrogel

Table S3. Summary of the resin formulation for highly entangled hydrogel synthesis.

Monomer	Crosslinker (XL)	Photo-initiator	Solvent
Acrylamide (AAm)	*N*,*N*'-Methylenebisacrylamide	Lithium phenyl-2,4,6-trimethylbenzoylphosphinate (LAP)	H_2O

Acrylamide was dissolved with the desired amount of water (m[AAm]:m[H_2O]=2:1) at 40 °C. Stock solution containing the photo-initiator (LAP) and crosslinker (XL) at varying ratios were prepared in water. For photoiniferter sample, the photoinitiator stock solution was prepared with a small amount of DMSO for better solubility. The stock solution was added into the reaction mixture to reach the desired [LAP]/[AAm]. After thorough mixing, the reaction mixture was injected in between two pieces of glass (**Figure S36**) using a syringe and needle. The silicone rubber was used as a spacer. And the glass was treated with a thin layer of RainX water repellent to reduce the adhesion and facilitate sample separate from the glass slides.

The casting mold containing the reaction mixture was placed on a lightbox (**Figure S3**) equipped with a 365 nm LED panel at the desired light intensity. After irradiation, the films were removed from the mold and weighed to obtain the initial mass (m_0). The samples were then immersed in deionized water for 48 hour and allowed to equilibrate and fully swell before being weighed again ($m_{swollen}$). The water was exchanged every 6 hours for a total of four or more exchanges to effectively remove unreacted monomer. The swelling ratio was calculated as:

$$Swelling\ ratio = \frac{m_{swollen}}{m_0} \qquad \textbf{Eq. S6}$$

Subsequently, the swollen samples were dried in a vacuum oven at 80 °C for 24 hours and weighed to obtain the dry mass (m_{dry}). The water content of the fully swollen hydrogel was calculated as:

$$Water\ Content\ (\%) = \frac{m_{swollen} - m_{dry}}{m_{swollen}} \times 100\% \qquad \textbf{Eq. S7}$$

The sol–gel fraction was estimated as:

$$Sol - Gel\ Fraction = \frac{m_{dry}}{m_0 \times 0.67} \qquad \textbf{Eq. S8}$$

where the factor 0.67 accounts for the initial 1:2 water-to-monomer mass ratio in the as-prepared resin.

All mechanical tests were performed on fully swollen samples.

S2 Supplementary Text

S2.1 ^{1}H and ^{13}C NMR and mass spectra

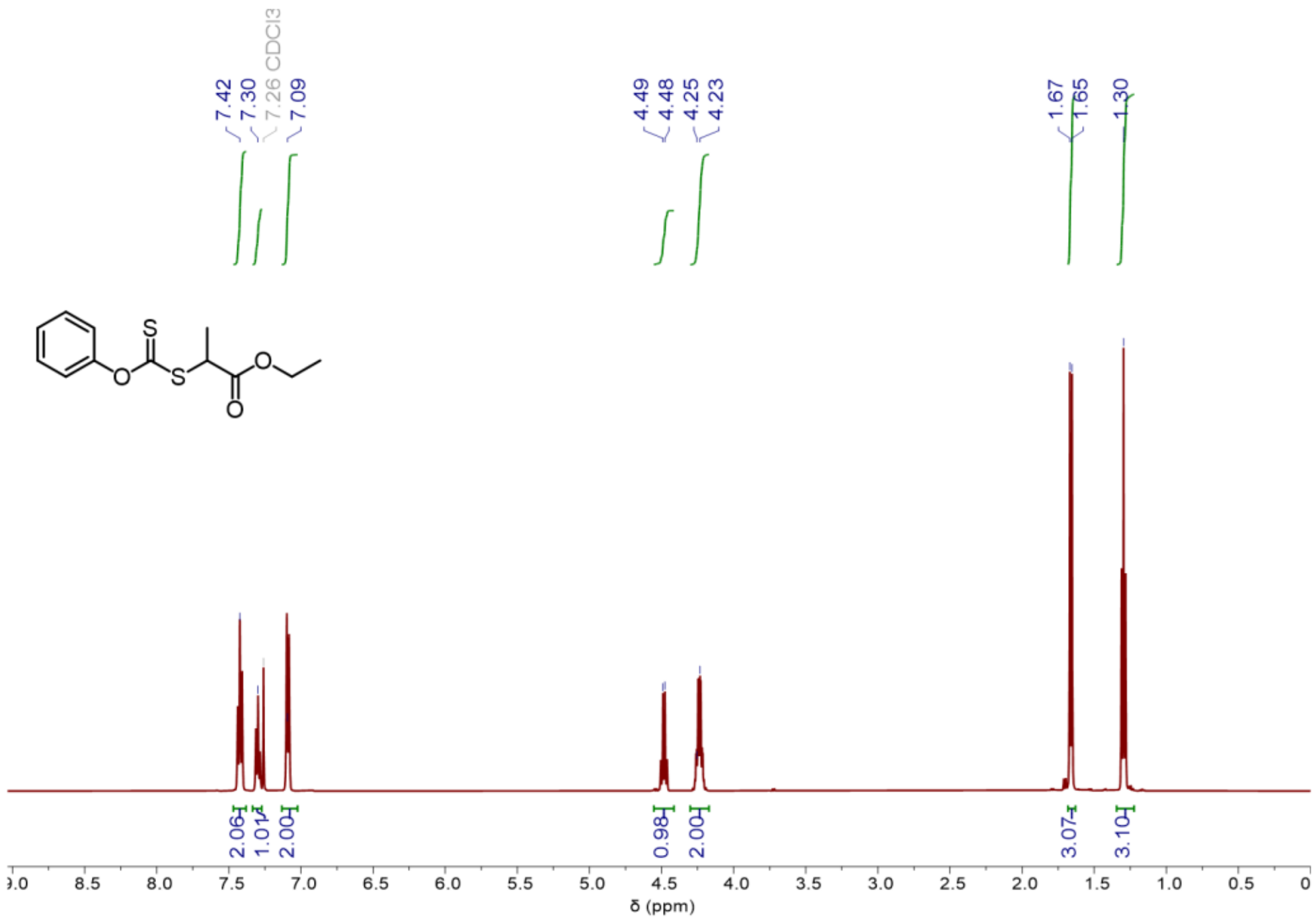

Figure S4. ^{1}H-NMR spectrum (500 MHz) of PXEP (CTA 1) in $CDCl_3$.

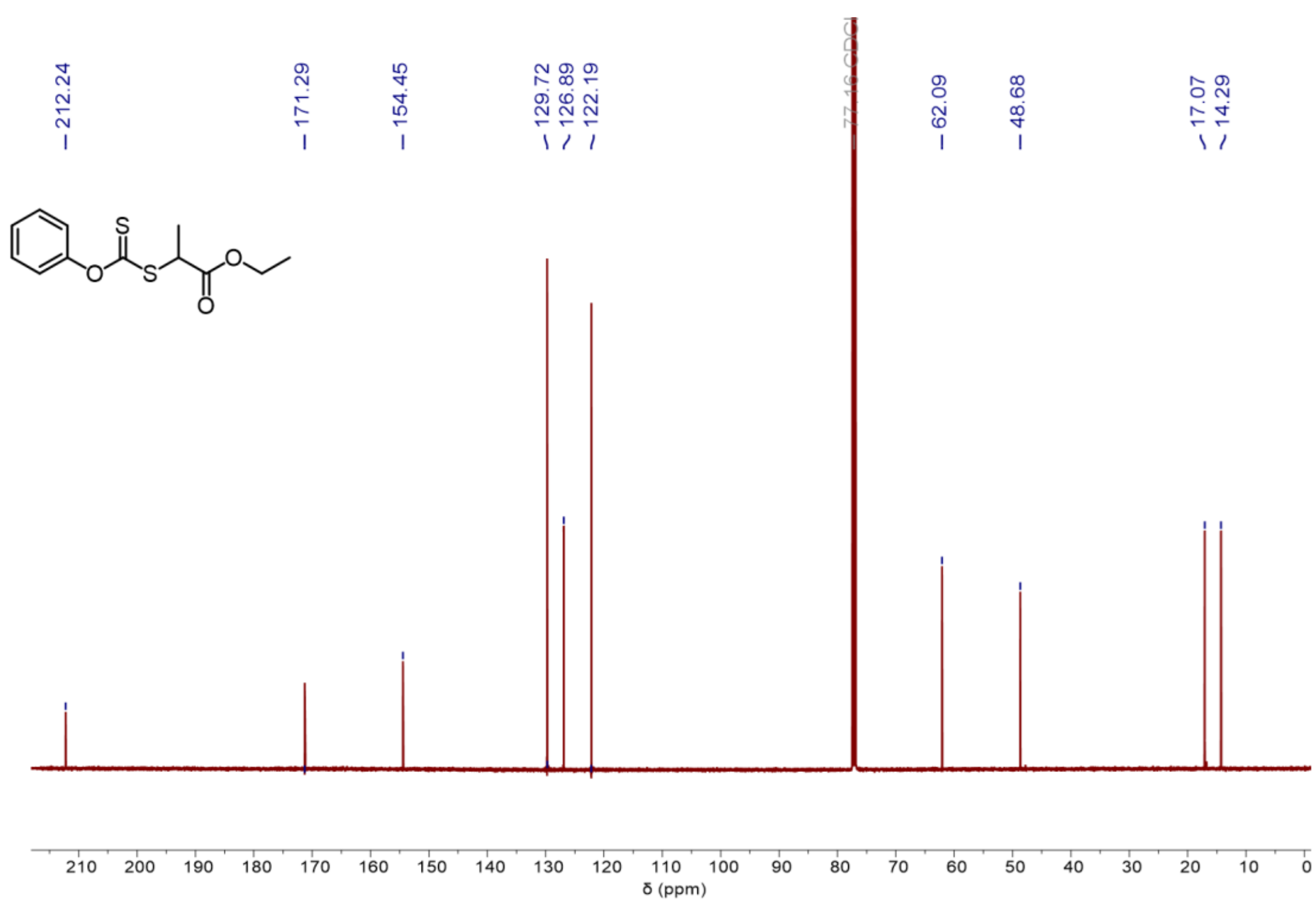

Figure S5. ^{13}C-NMR spectrum (125 MHz) of PXEP (CTA 1) in $CDCl_3$.

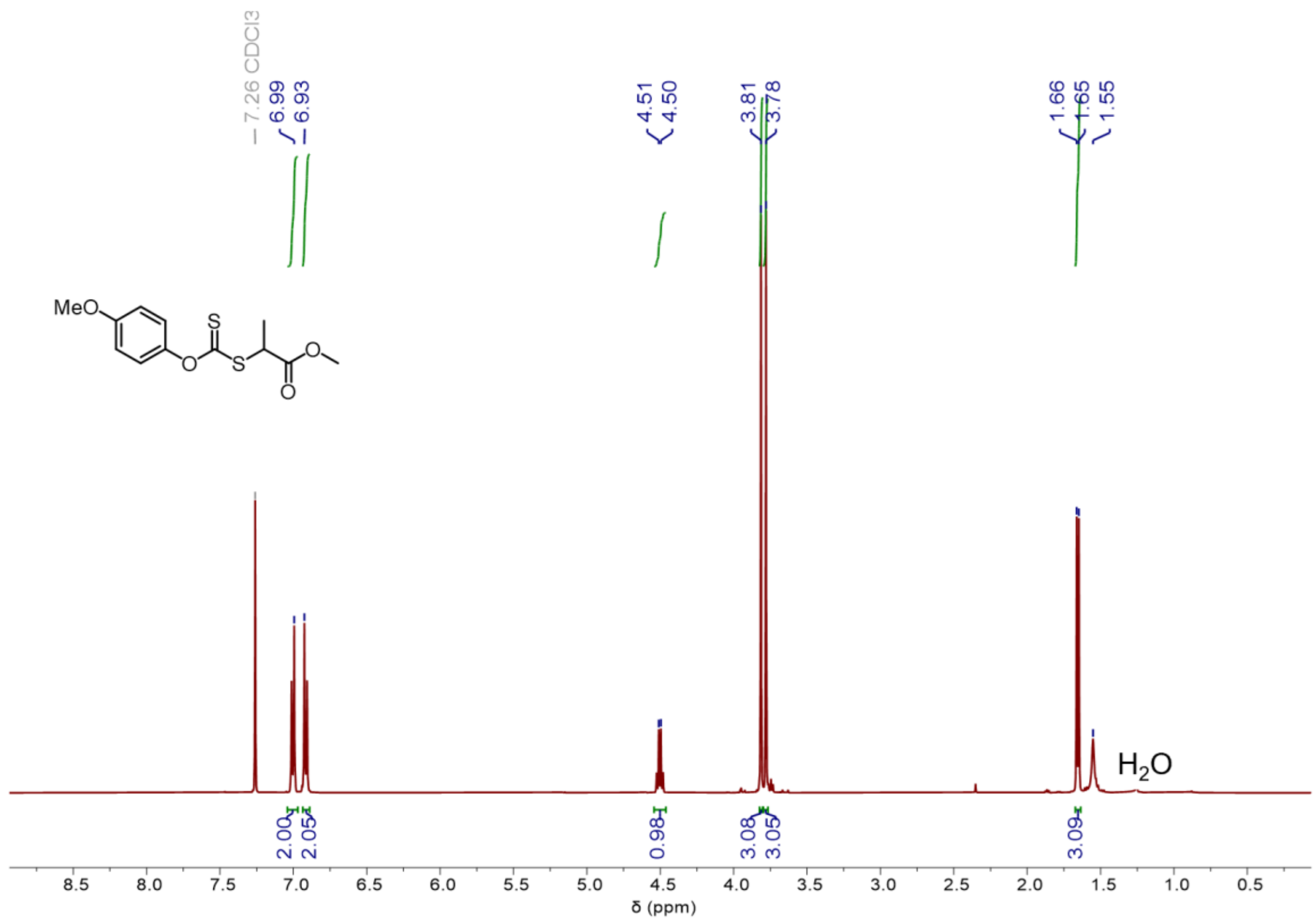

Figure S6. ^{1}H-NMR spectrum (500 MHz) of MeOPXEP (CTA 2) in $CDCl_3$.

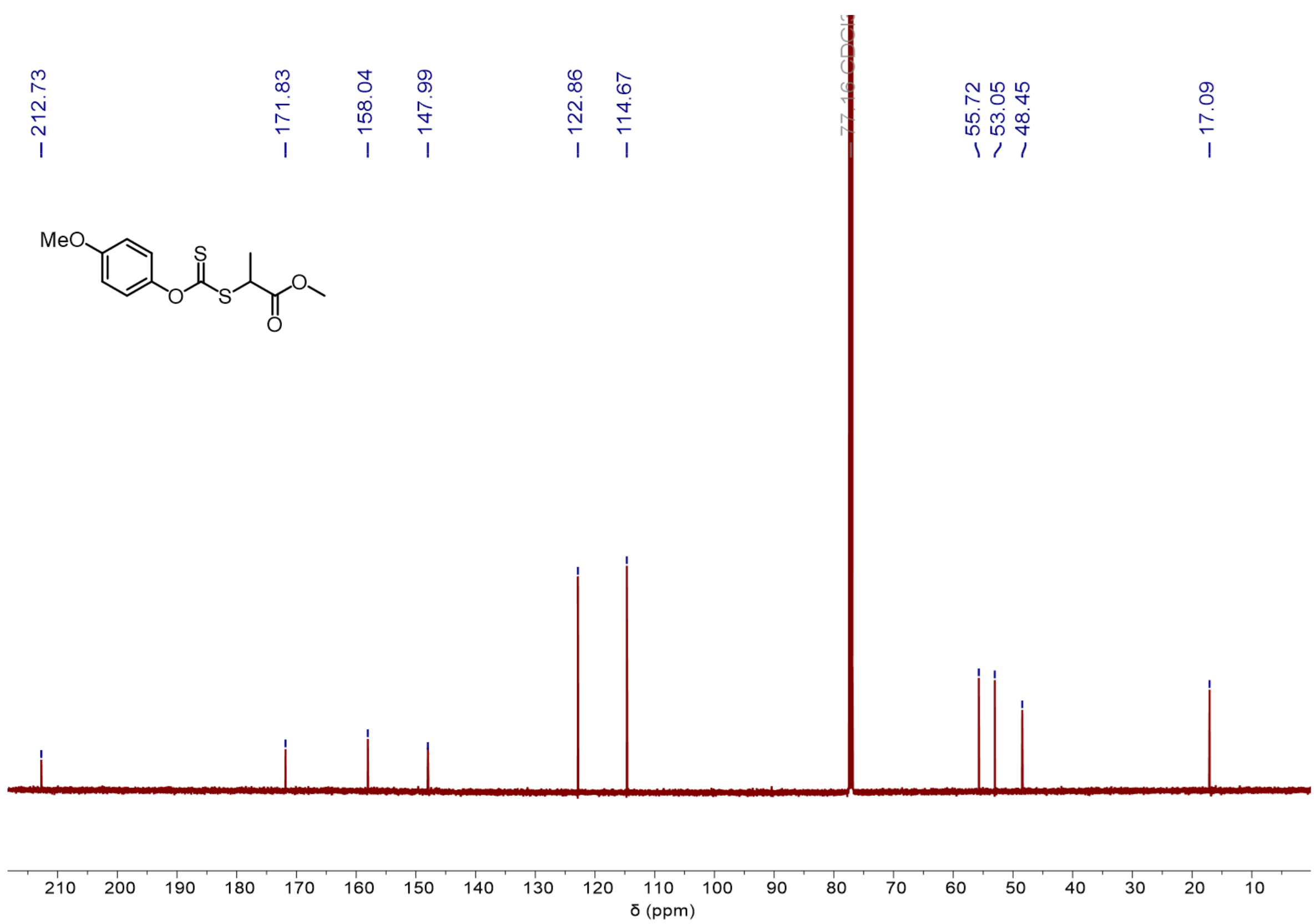

Figure S7. ^{13}C-NMR spectrum (125 MHz) of MeOPXEP (CTA 2) in $CDCl_3$.

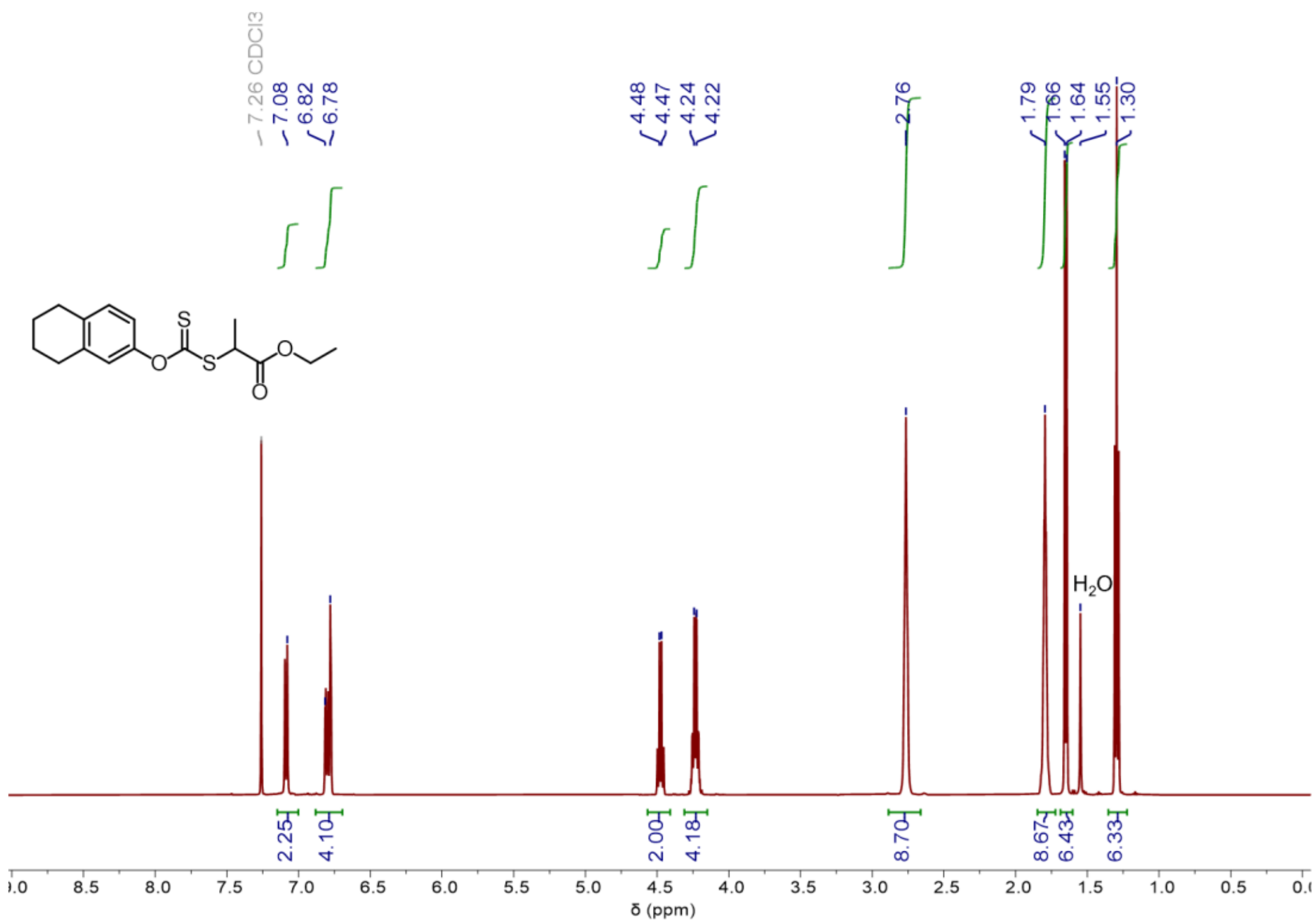

Figure S8. ^{1}H-NMR spectrum (500 MHz) of TNXEP (CTA 3) in $CDCl_3$.

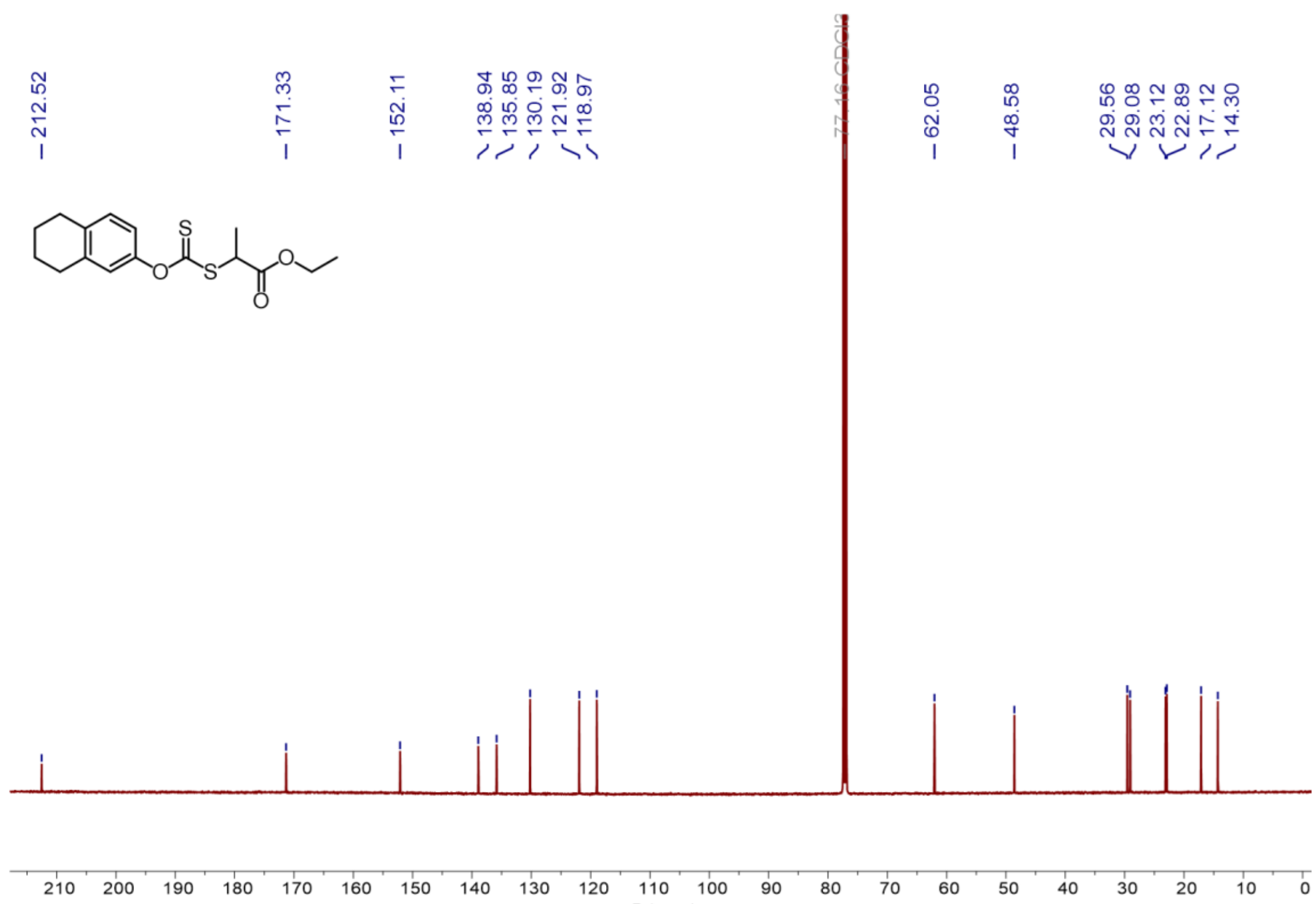

Figure S9. ^{13}C-NMR spectrum (125 MHz) of TNXEP (CTA 3) in $CDCl_3$.

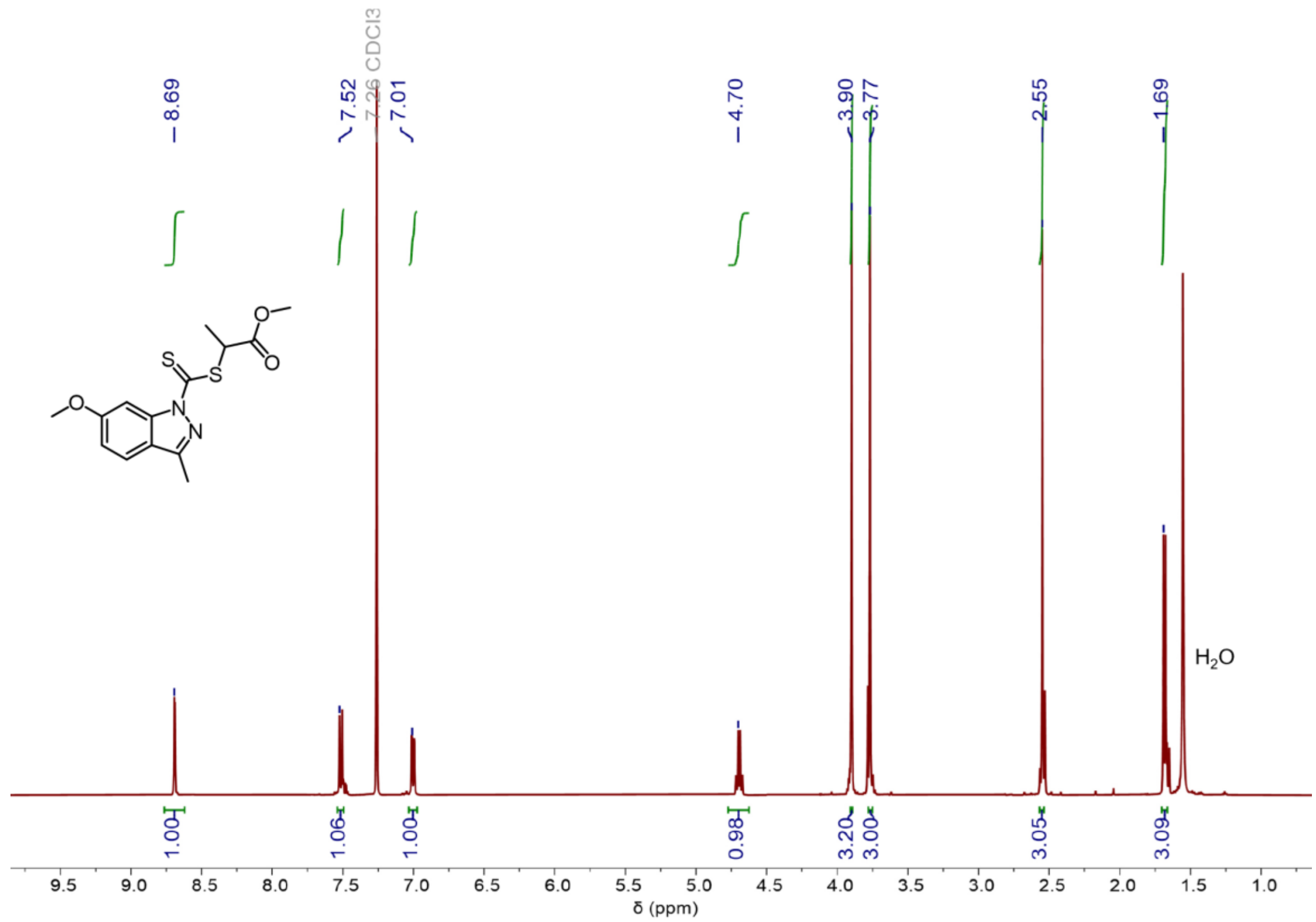

Figure S10. ^{1}H-NMR spectrum (500 MHz) of DTI-ester (CTA 4) in $CDCl_3$.

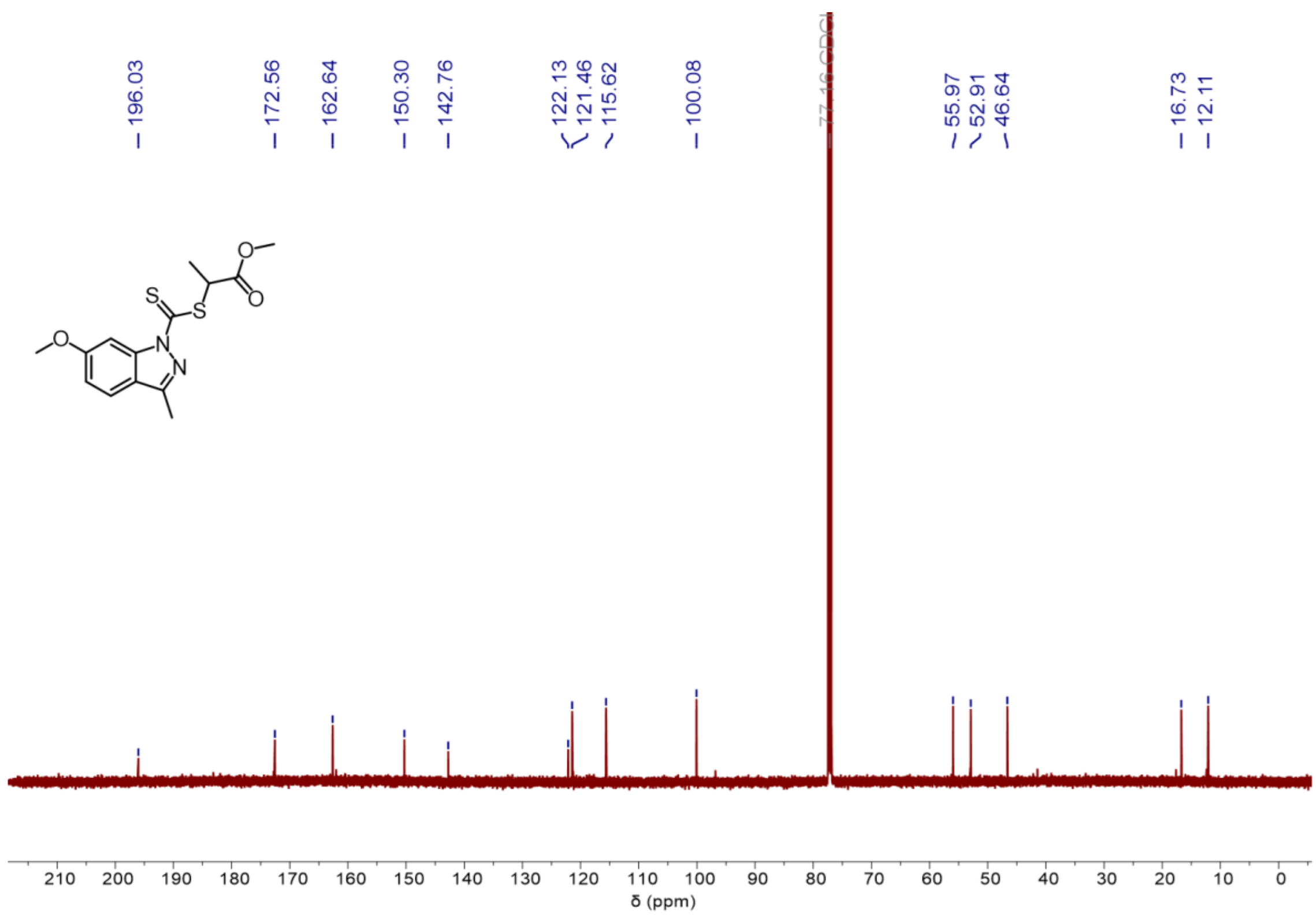

Figure S11. ^{13}C-NMR spectrum (125 MHz) of DTI-ester (CTA 4) in $CDCl_3$.

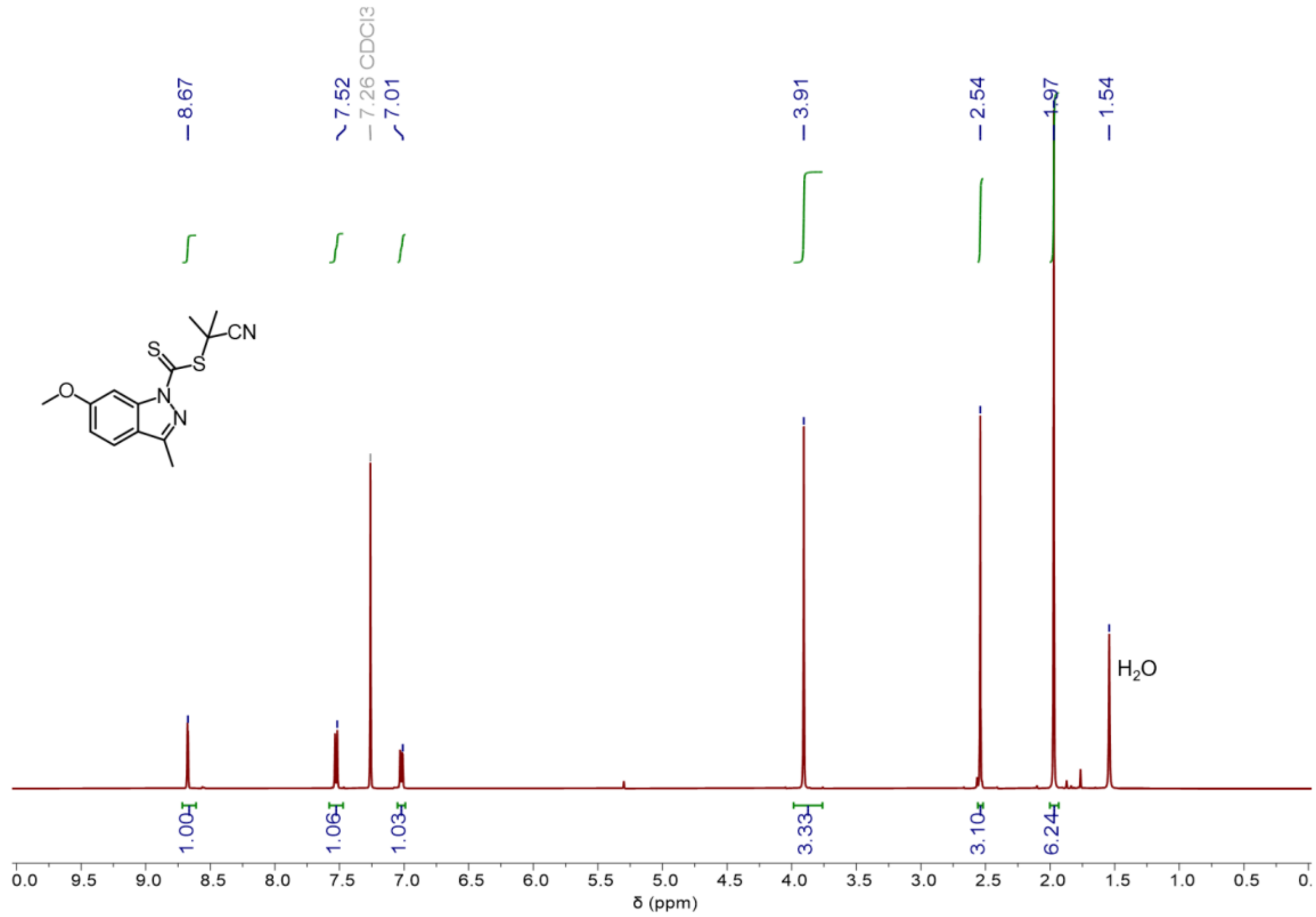

Figure S12. ^{1}H-NMR spectrum (500 MHz) of DTI-CN (CTA 5) in $CDCl_3$.

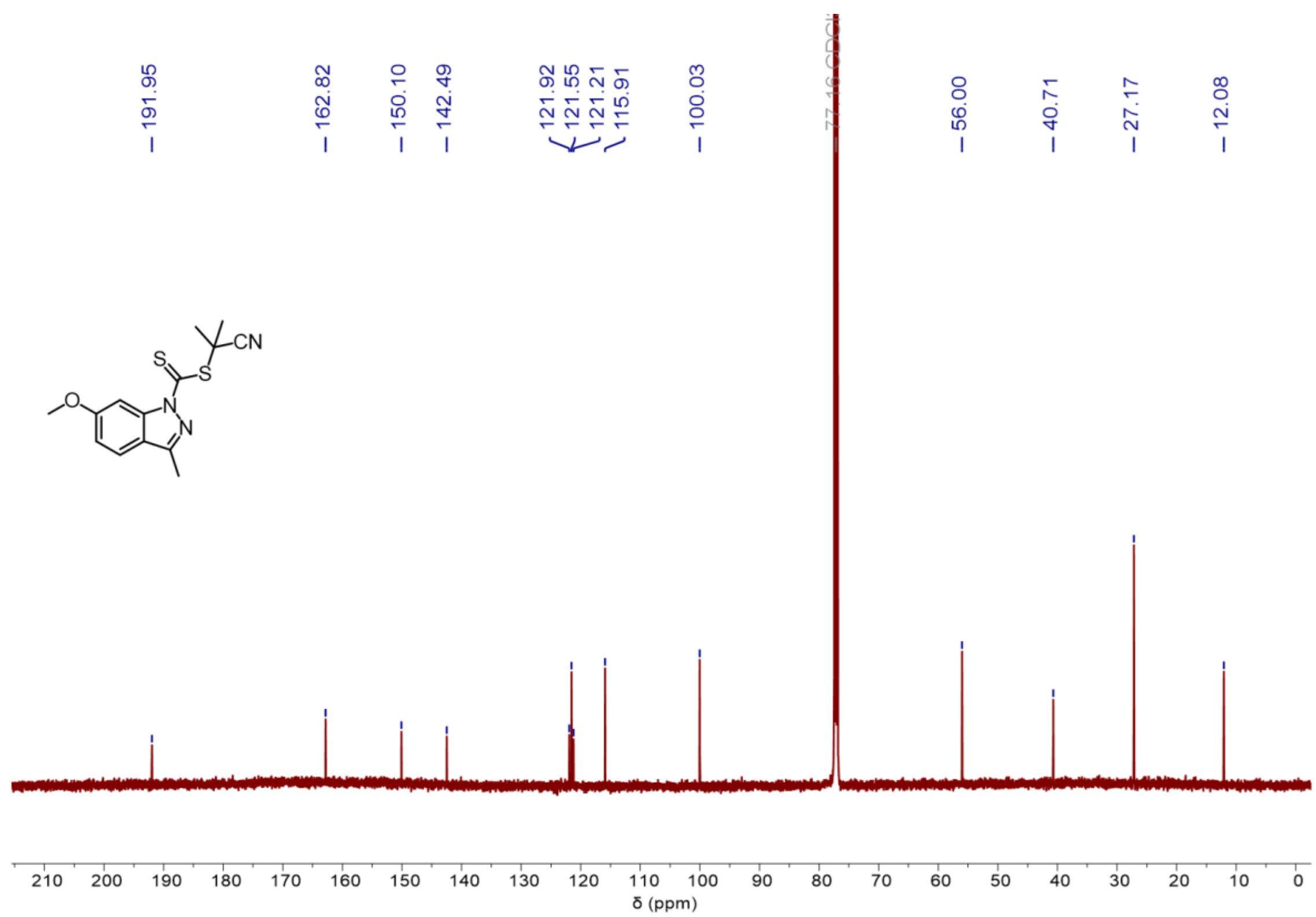

Figure S13. ^{13}C-NMR spectrum (125 MHz) of DTI-CN (CTA 5) in $CDCl_3$.

Target Compound Screening Report

Results Acquired by The University of Texas at Austin Mass Spectrometry Facility

**Data File**	MSF26-0438(YL-03-75)_hrESIpos1.d	**Sample Name**	0438(YL-03-75)	**Comment**	0438(YL-03-75)
**Position**	P1-D4	**Instrument Name**	6530	**User Name**	
**Acq Method**	FIA_pos.m	**Acquired Time**	2/24/2026 2:30:21 PM	**DA Method**	MSF.m

MS Zoomed Spectrum

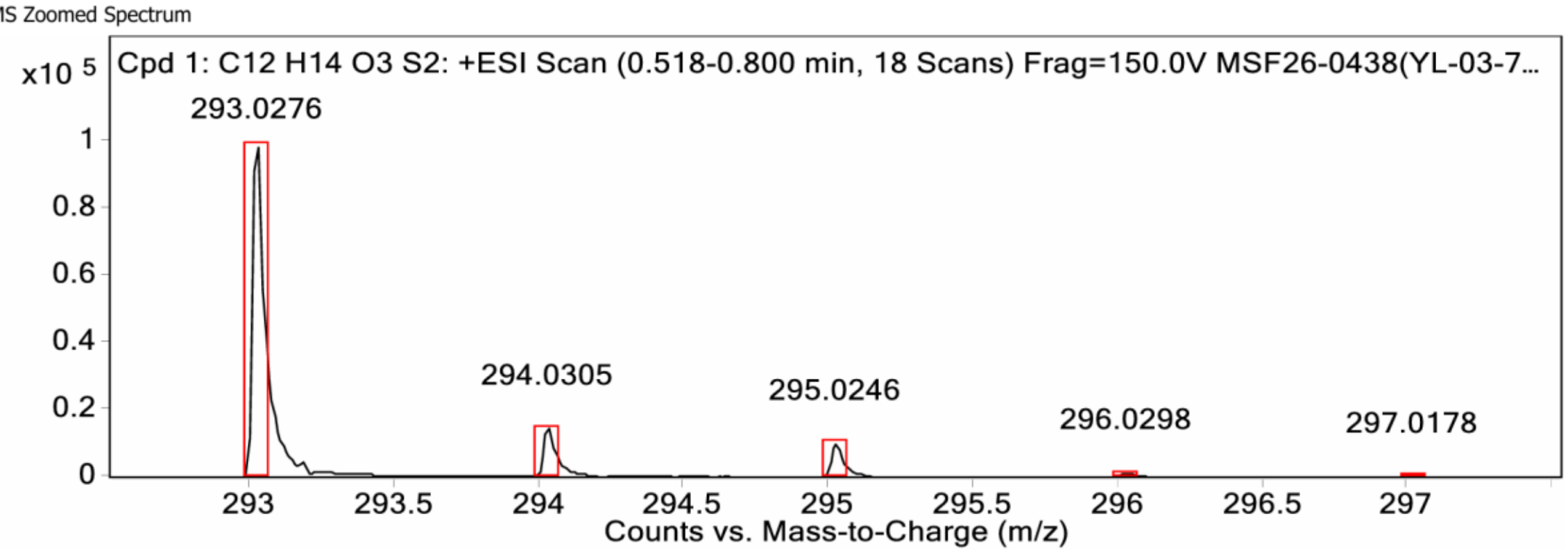

MS Spectrum Peak List

Obs. m/z	Calc. m/z	Charge	Abundance	Formula	Ion Species	Tgt Mass Error (ppm)
293.0276	293.0277	1	99308	C12H14O3S2	(M+Na)+	0.18
294.0305	294.0306	1	14680	C12H14O3S2	(M+Na)+	0.3
295.0246	295.0249	1	10173	C12H14O3S2	(M+Na)+	0.96
296.0298	296.0275	1	1435	C12H14O3S2	(M+Na)+	-7.82
297.0178	297.0235	1	335	C12H14O3S2	(M+Na)+	19.11

--- End Of Report ---

Figure S14. HRMS of PXEP (CTA 1).

Target Screening Report

Agilent | Trusted Answers

Results Acquired by The University of Texas at Austin Mass Spectrometry Facility

**Data File**	MSF26-0432(YL-03-83)_hrESIpos1.d	**Sample Name**	0432(YL-03-83)	**Comment**	0432(YL-03-83)
**Position**	P1-A9	**Instrument Name**	Instrument 1		
**Acquisition Method**	FIA_Pos.m	**Acquired Time**	2/23/2026 2:58:36 PM (UTC-06:00)	**DA Method**	MSF.m

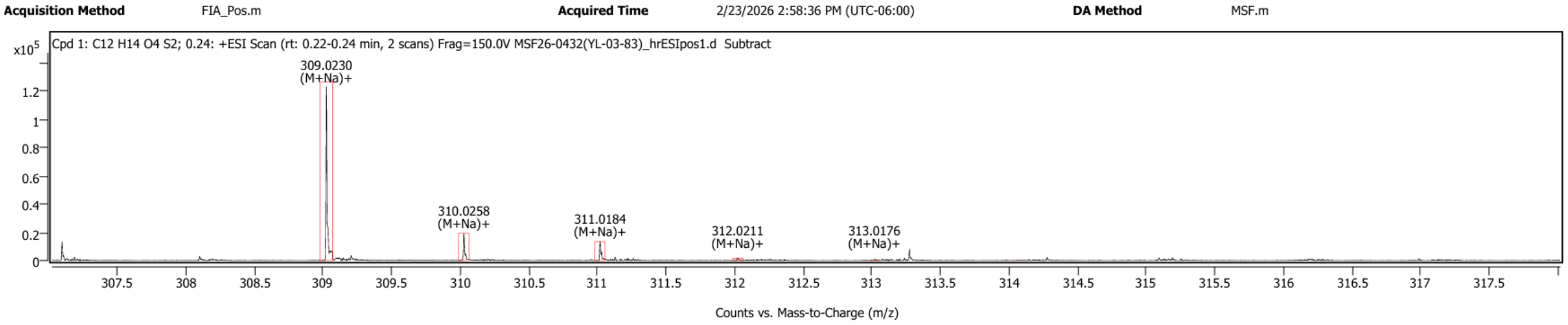

Spectrum Peaks

Obs. m/z	Calc. m/z	Charge	Abund	Formula	Ion Species	Tgt Mass Error (PPM)
309.0230	309.0226	1	124825	C12H14O4S2	(M+Na)+	1.41
310.0258	310.0255	1	19215	C12H14O4S2	(M+Na)+	0.84
311.0184	311.0200	1	13214	C12H14O4S2	(M+Na)+	-4.92
312.0211	312.0226	1	1913	C12H14O4S2	(M+Na)+	-4.72
313.0176	313.0188	1	674	C12H14O4S2	(M+Na)+	-3.79
228.2440			1938162			

MassHunter Qual 10.0
(End of Report)

Figure S15. HRMS of MeOPXEP (CTA 2).

Target Screening Report

Results Acquired by The University of Texas at Austin Mass Spectrometry Facility

**Data File**	MSF26-0433(YL-03-99)_hrESIpos1.d	**Sample Name**	0433(YL-03-99)	**Comment**	0433(YL-03-99)
**Position**	P1-B1	**Instrument Name**	Instrument 1		
**Acquisition Method**	FIA_Pos.m	**Acquired Time**	2/23/2026 3:01:10 PM (UTC-06:00)	**DA Method**	MSF.m

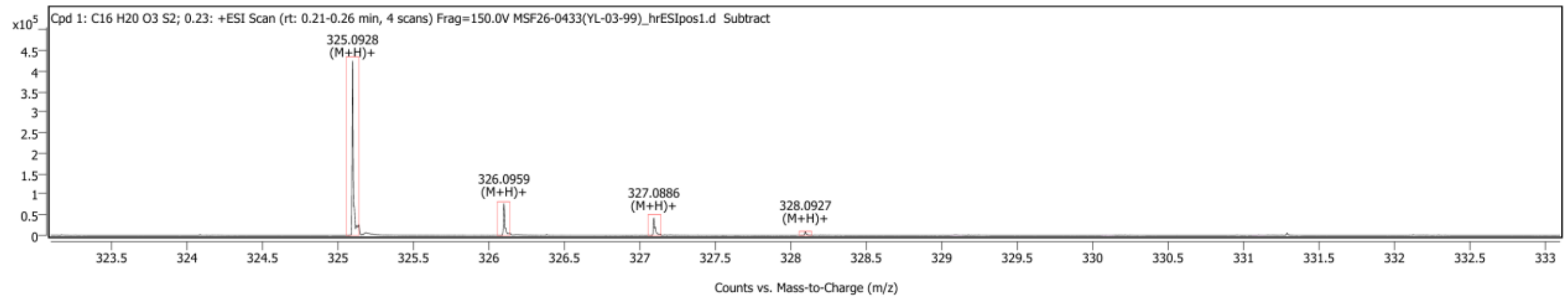

Spectrum Peaks

Obs. m/z	Calc. m/z	Charge	Abund	Formula	Ion Species	Tgt Mass Error (PPM)
325.0928	325.0927	1	432789	C16H20O3S2	(M+H)+	0.55
326.0959	326.0957	1	78185	C16H20O3S2	(M+H)+	0.36
327.0886	327.0905	1	42669	C16H20O3S2	(M+H)+	-5.88
328.0927	328.0928	1	9017	C16H20O3S2	(M+H)+	-0.14
191.0528			2878010			

MassHunter Qual 10.0
(End of Report)

Figure S16. HRMS (ESI) of TNXEP (CTA 3).

Target Screening Report

Results Acquired by The University of Texas at Austin Mass Spectrometry Facility

**Data File**	MSF26-0434(YL-03-106)_hrESIpos1.d	**Sample Name**	0434(YL-03-106)	**Comment**	0434(YL-03-106)
**Position**	P1-B2	**Instrument Name**	Instrument 1		
**Acquisition Method**	FIA_Pos.m	**Acquired Time**	2/23/2026 3:03:36 PM (UTC-06:00)	**DA Method**	MSF.m

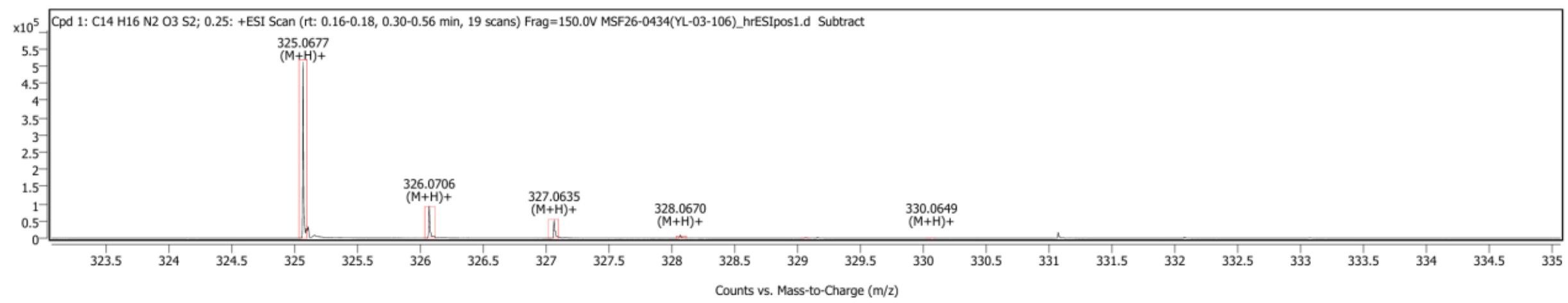

Spectrum Peaks

Obs. m/z	Calc. m/z	Charge	Abund	Formula	Ion Species	Tgt Mass Error (PPM)
325.0677	325.0675	1	515309	C14H16N2O3S2	(M+H)+	0.62
326.0706	326.0703	1	93987	C14H16N2O3S2	(M+H)+	1.00
327.0635	327.0651	1	50644	C14H16N2O3S2	(M+H)+	-4.71
328.0670	328.0672	1	9071	C14H16N2O3S2	(M+H)+	-0.56
330.0649	330.0653	1	294	C14H16N2O3S2	(M+H)+	-1.26

MassHunter Qual 10.0
(End of Report)

Figure S17. HRMS of DTI-ester (CTA 4).

Target Screening Report

Results Acquired by The University of Texas at Austin Mass Spectrometry Facility

**Data File**	MSF26-0435(YL-03-108)_hrESIpos1.d	**Sample Name**		**Comment**	
**Position**		**Instrument Name**			
**Acquisition Method**		**Acquired Time**	2/23/2026 3:06:02 PM (UTC-06:00)	**DA Method**	MSF.m

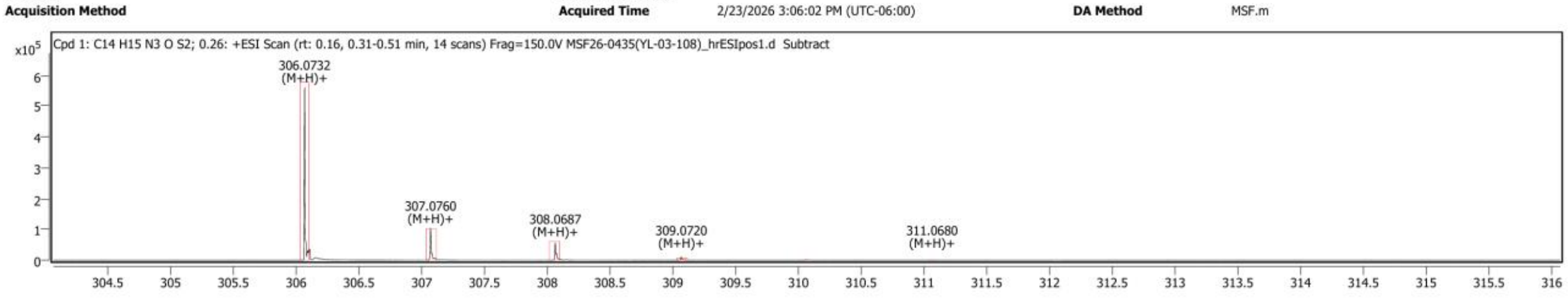

Spectrum Peaks

Obs. m/z	Calc. m/z	Charge	Abund	Formula	Ion Species	Tgt Mass Error (PPM)
306.0732	306.0729	1	573467	C14H15N3OS2	(M+H)+	0.81
307.0760	307.0756	1	103004	C14H15N3OS2	(M+H)+	1.37
308.0687	308.0703	1	56478	C14H15N3OS2	(M+H)+	-4.94
309.0720	309.0722	1	10292	C14H15N3OS2	(M+H)+	-0.66
311.0680	311.0697	1	245	C14H15N3OS2	(M+H)+	-5.42

MassHunter Qual 10.0
(End of Report)

Figure S18. HRMS of DTI-CN (CTA 5).

S2.2 Influence of PPh_3

The reaction kinetics for ethyl acrylate were characterized using real time Fourier transform infrared (FTIR) spectroscopy in a transmission configuration. The polymerization rate was calculated from the initial slope of the conversion vs. time plots from 1% to 10% conversion. Additionally, t_{inhib} was determined as the time required to reach 1% monomer (C=C–H) conversion, excluding the initial 10 seconds before the LED was turned on. ρ_{max} was determined as the max conversion at the end of light irradiation.

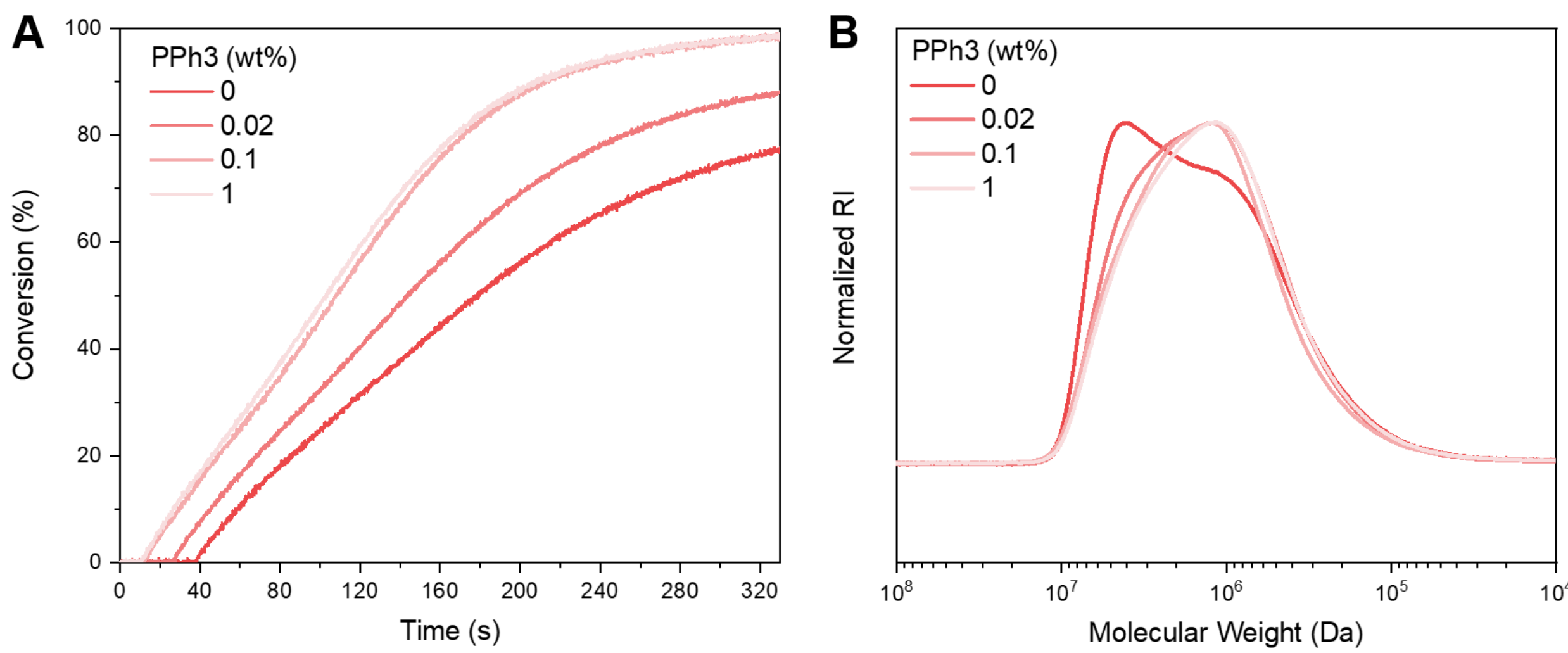

Figure S19. Ethyl acrylate homopolymerization with [BAPO] = 0.01 mol% under 5 mW/cm^2 365 nm LED with varying the amount of PPh_3. (A). Conversion vs time plot of ethyl acrylate polymerization initiated with varying the amount of PPh_3. (B). The corresponding GPC traces of the polymers at the end of the irradiation (320 s).

Table S4. Summary of **Figure S19A**. Real-time FTIR kinetics of ethyl acrylate homopolymerization with [BAPO] = 0.01 mol% and varying [PPh_3]. Samples (100 µm thick) were irradiated for 320s at 5 mW/cm^2 365 nm LED. Values are averages (n ≥3) with ±1 standard deviation from the mean provided.

[BAPO] (mol%)	PPh_3 (wt%)	Initial Rate (%s^{-1})	Initial Rate (mM/s)	ρ_{max} in run time (%)	Inhibition time t_{inhib} (s)
0.01	0	0.44 ± 0.02	41 ± 2	77.9 ± 0.7	28.6 ± 0.3
0.01	0.02	0.497 ± 0.009	46.6 ± 0.8	88.4 ± 0.3	17.6 ± 0.2
0.01	0.1	0.58 ± 0.01	54 ± 1	98.53 ± 0.07	3.3 ± 0.2
0.01	1	0.64 ± 0.03	60 ± 3	99.12 ± 0.03	1.7 ± 0.1

Table S5. Summary of **Figure S19B**. GPC results of poly(ethyl acrylate), relative to polystyrene standards, prepared using [BAPO] = 0.01 mol%, varying PPh^3, 5 mW/cm^2 365 nm LED irradiation (320s), and 100 µm sample thickness.

[BAPO] (mol%)	PPh_3 (wt%)	$M_{n,GPC}$ (kDa)	M_w/M_n	Conversion, ρ^a (%)
0.01	0	672.3	3.23	78
0.01	0.02	600.6	3.07	88
0.01	0.1	621.7	2.64	99
0.01	1	600.5	2.91	99

[a]Monomer conversion obtained from real-time FTIR spectroscopy

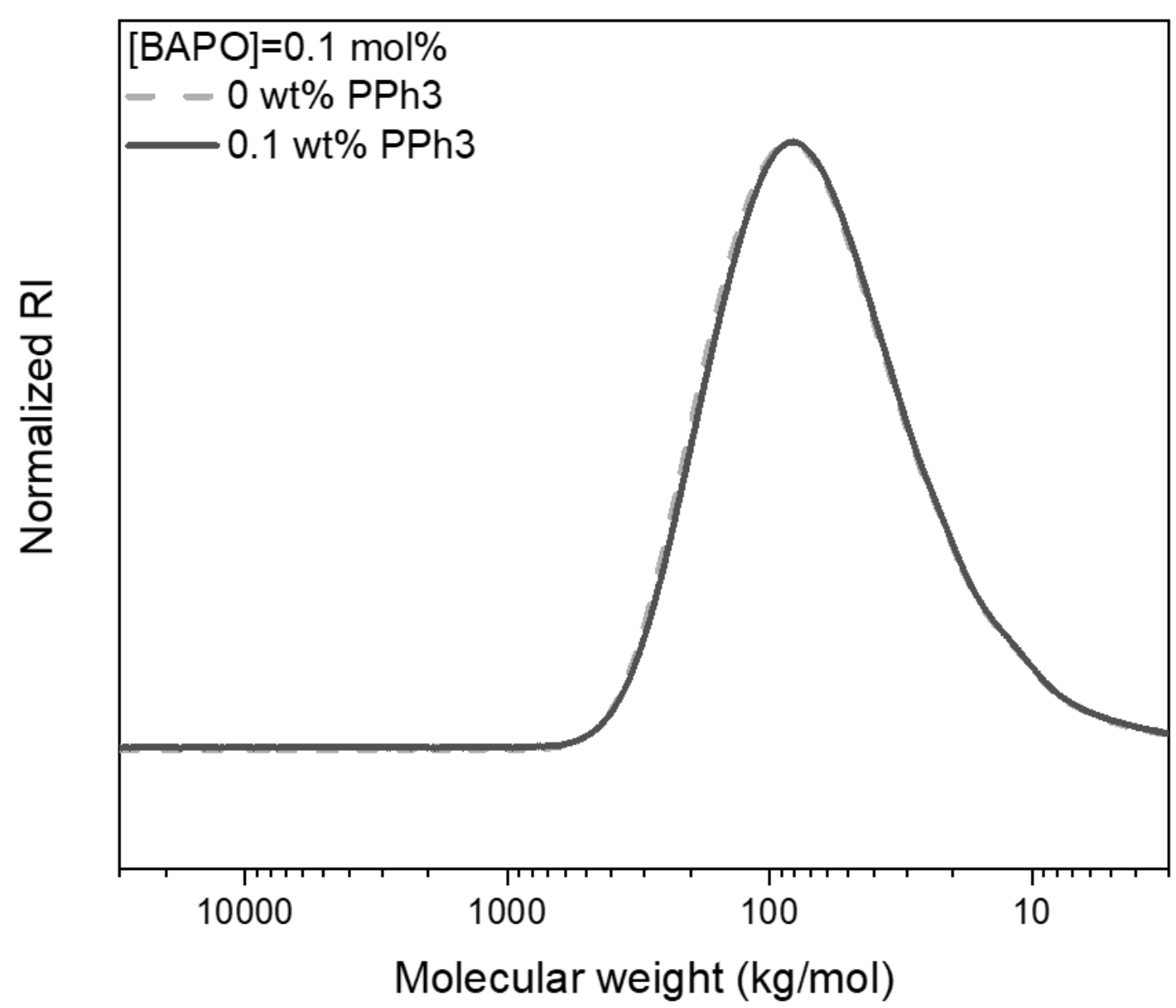

Figure S20. The GPC traces of poly(ethyl acrylate) prepared using [BAPO] = 0.1 mol%, varying PPh_3, 5 mW/cm^2 365 nm LED irradiation (320s), and 100 µm sample thickness.

Table S6. Summary of **Figure S20**. GPC results of poly(ethyl acrylate), relative to polystyrene standards, prepared using [BAPO] = 0.1 mol%, varying PPh^3, 5 mW/cm^2 365 nm LED irradiation (320s), and 100 µm sample thickness.

[BAPO] (mol%)	PPh_3 (wt%)	$M_{n,GPC}$ (kDa)	M_w/M_n
0.1	0	39.9	2.28
0.1	0.1	41.1	2.17

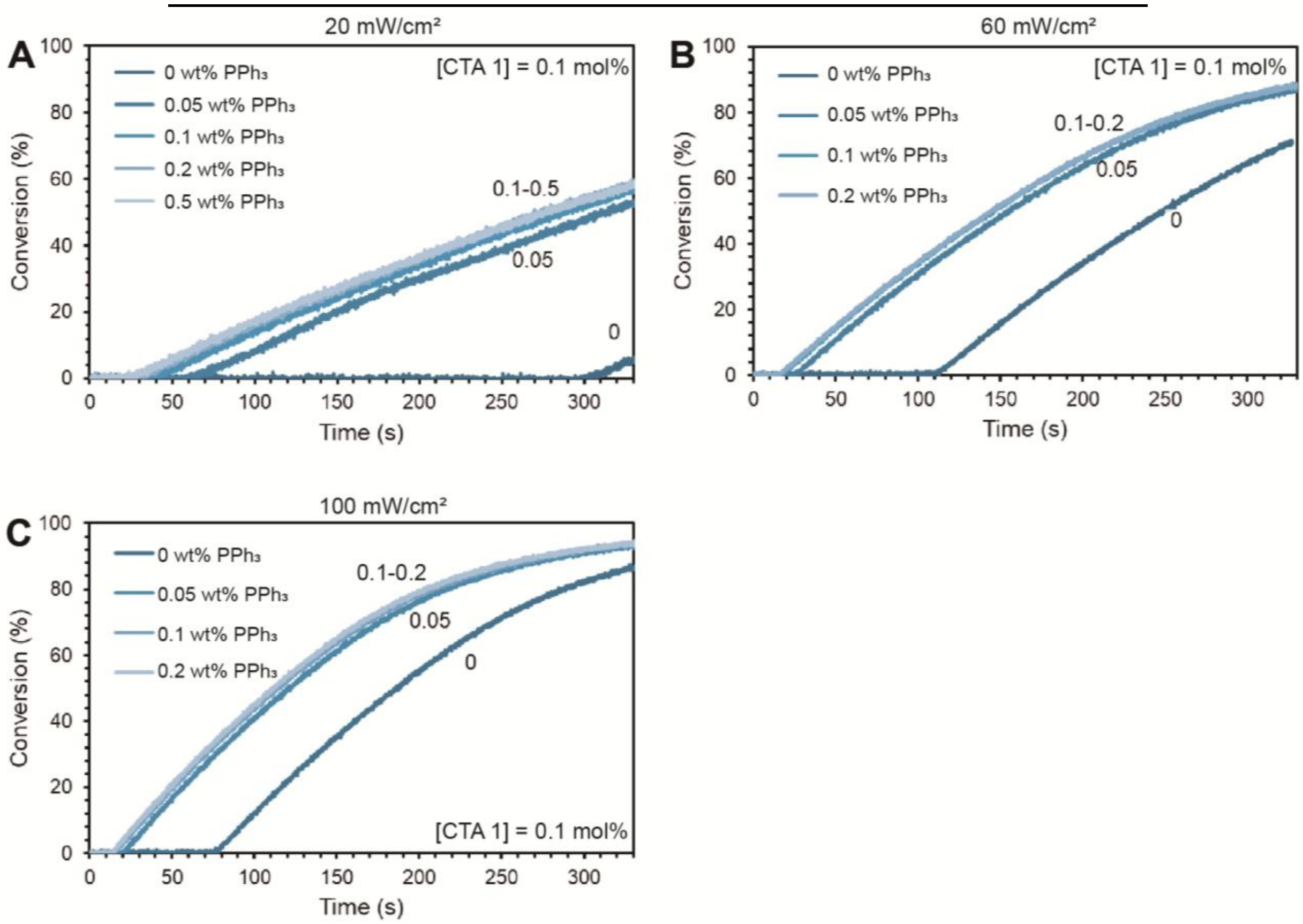

Figure S21. Conversion vs time plots obtained using real-time FTIR spectroscopy of ethyl acrylate photoiniferter polymerization with PXEP (CTA 1), [CTA 1] = 0.1 mol% under varying intensity 365 nm LED irradiation and varying the amount of PPh_3. (A) 20 mW/cm^2 365 nm LED; (B) 60 mW/cm^2 365 nm LED; (C) 100 mW/cm^2 365 nm LED.

S2.3 Influence of Monomer Inhibitors

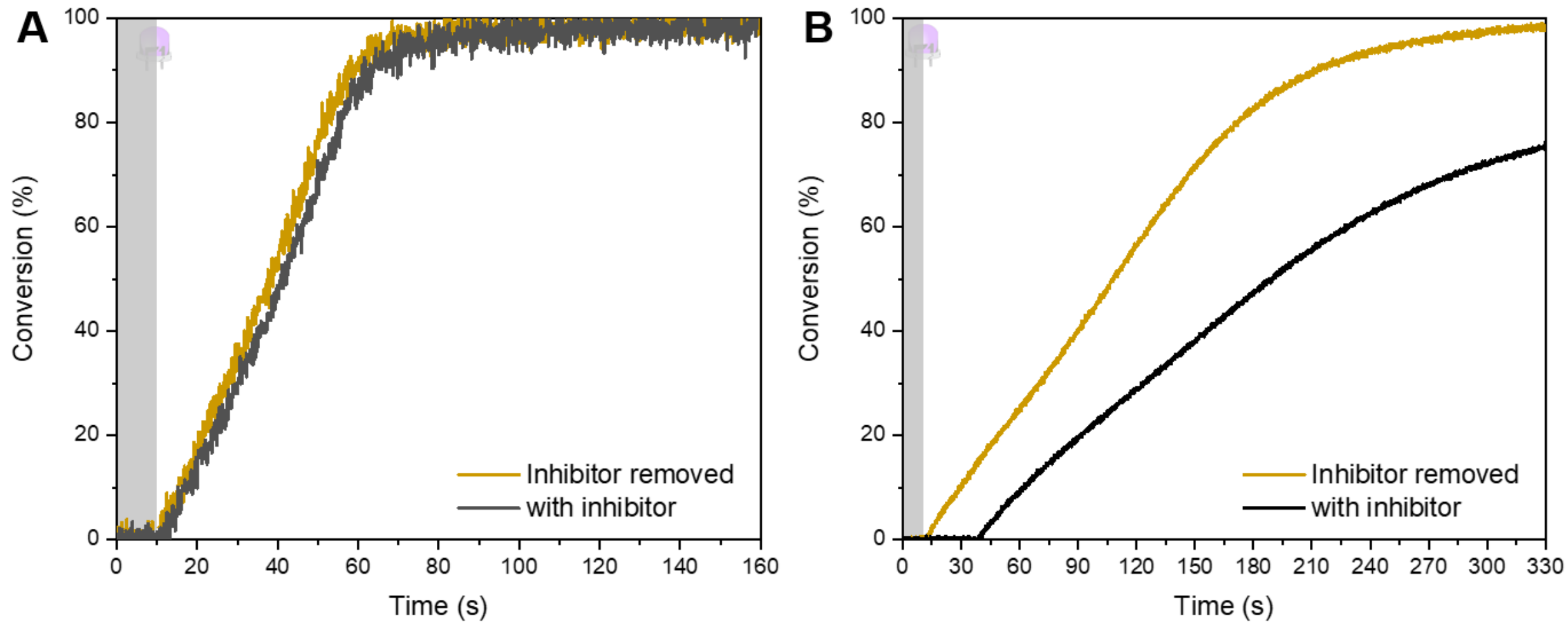

Figure S22. Ethyl acrylate (stabilized with MEHQ, TCI chemical) used with and without inhibitor removal. To remove inhibitor, monomer was passed through Brockmann I activated basic alumina Al_2O_3 column before use. After removal, PPh_3 (0.1 wt%) was added to minimize oxygen inhibition. Conversion vs time plots of ethyl acrylate polymerizations from real-time FTIR spectroscopy of 100 µm thick samples with and without monomer inhibitor removal under 5 mW/cm^2 365 nm LED exposure for (A) 150 s with [BAPO] = 0.1 mol% or (B) 320 s with [BAPO] = 0.01 mol%.

Table S7. Summary of **Figure S22**. Examining the effect of inhibitor on the kinetics of ethyl acrylate homopolymerization with BAPO. PPh_3 (0.1 wt%) was added to minimize oxygen inhibition. Samples were 100 µm thick and exposed to a 5 mW/cm^2 365 nm LED for 150 s (0.1 mol% BAPO) or 320 s (0.01 mol% BAPO). Values are averages (n ≥3) with ±1 standard deviation from the mean provided.

[BAPO] (mol%)	Inhibitor	Initial Rate (%s^{-1})	Initial Rate (mM/s)	ρ_{max} in run time (%)	Inhibition time t_{inhib} (s)
0.1	with	1.21 ± 0.07	114 ± 7	98.3 ± 0.5	3.9 ± 1.1
0.1	removed	1.28 ± 0.05	120 ± 4	98.5 ± 0.3	2.2 ± 0.5
0.01	with	0.42 ± 0.01	39.6 ± 0.5	73 ± 4	30 ± 1
0.01	removed	0.58 ± 0.01	54 ± 1	98.53 ± 0.07	3.3 ± 0.2

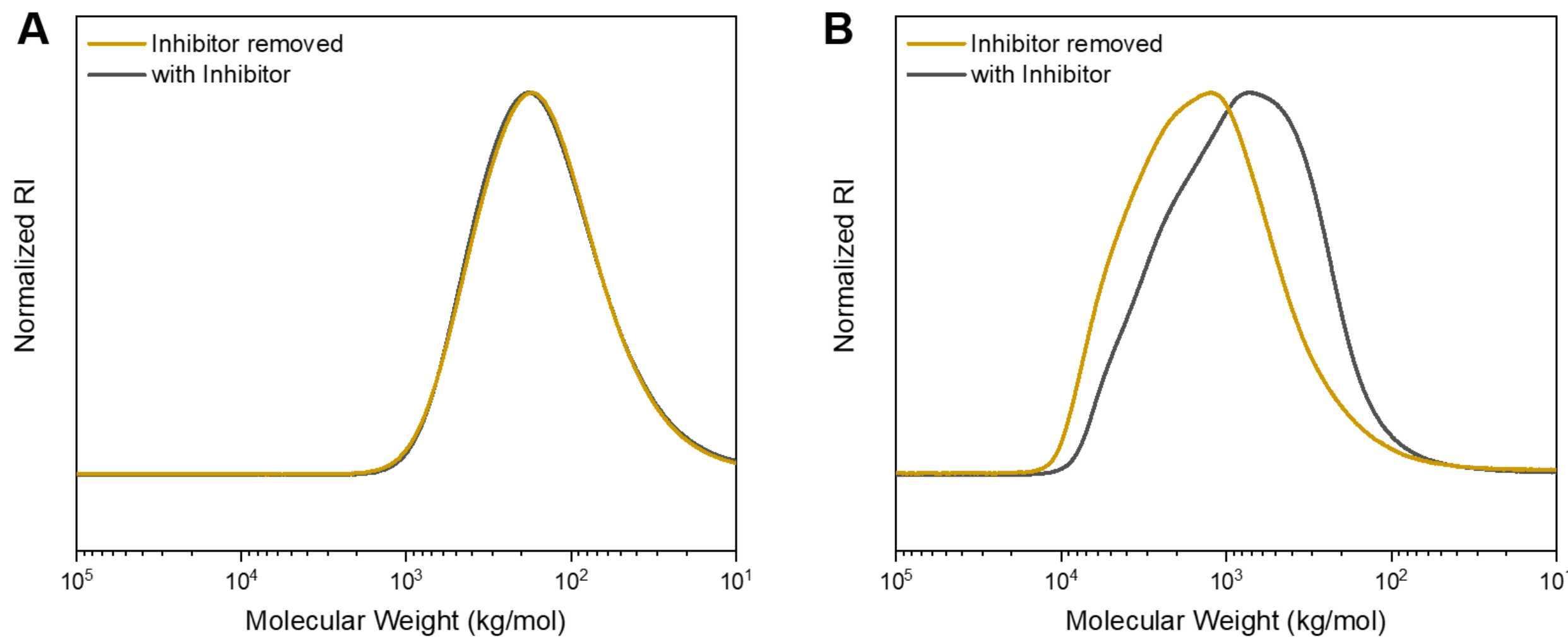

Figure S23. The corresponding GPC traces of the polymers at the end of the irradiation in **Figure S22**. Ethyl acrylate used with and without inhibitor removal. PPh_3 (0.1 wt%) was added to minimize oxygen inhibition. Samples were 100 µm thick and exposed to a 5 mW/cm^2 365 nm LED. (A) [BAPO]= 0.1 mol%, 150 s irradiation. (B) [BAPO]= 0.01 mol%, 320 s irradiation.

Table S8. Summary of **Figure S23**. GPC results of poly(ethyl acrylate), relative to polystyrene standards, prepared from of 100 µm thick samples with and without monomer inhibitor removal under 5 mW/cm^2 365 nm LED exposure.

[BAPO] (mol%)	Inhibitor	Irradiation Time (s)	$M_{n,GPC}$ (kDa)	M_w/M_n	Conv.[a] (%)
0.1	with	150	96.3	2.15	98
0.1	removed	150	98.5	2.14	99
0.01	with	320	441.6	2.72	73
0.01	removed	320	678.7	2.78	99

[a]Monomer conversion is obtained from real-time FTIR spectroscopy

S2.4 Kinetics of EA polymerization with different CTAs

S2.4.1 Comparison between different CTAs

The reaction kinetics for the photoiniferter polymerization of ethyl acrylate were characterized using real time Fourier transform infrared (FTIR) spectroscopy in a transmission configuration. The polymerization rate was calculated from the initial slope of the conversion vs. time plots from 3% to 10% conversion. Additionally, t_{inhib} was determined as the time required to reach 3% monomer (C=C–H) conversion, excluding the initial 10 seconds before the LED was turned on. ρ_{max} was determined as the max conversion at the end of light irradiation.

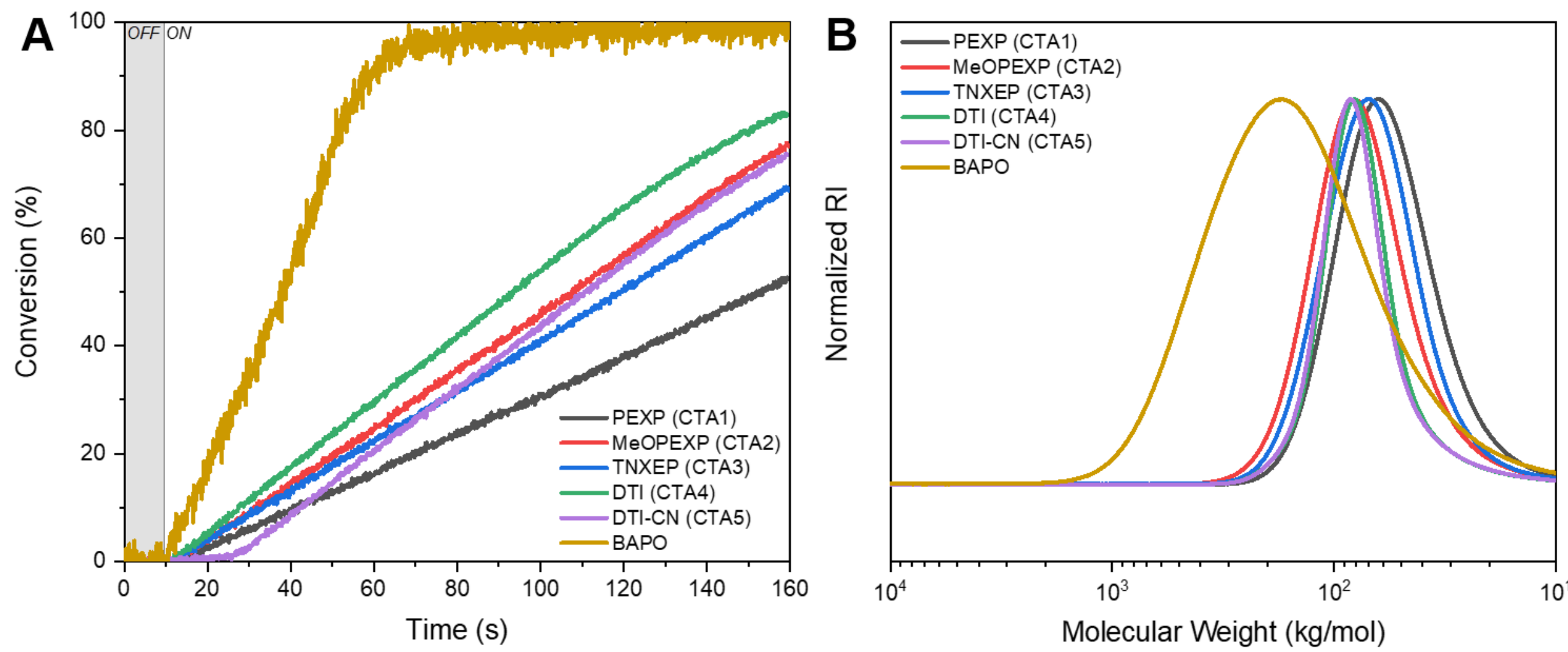

Figure S24. Photoiniferter polymerization of ethyl acrylate with different CTAs (0.1 mol%) under 60 mW/cm^2 365 nm LED (CTA1-3) or under 60 mW/cm^2 405 nm LED (CTA4-5) exposure. As a control, free radical polymerization with [BAPO] = 0.1 mol% under 5 mW/cm^2 365 nm LED is provided. PPh_3 (0.1 wt%) was added to minimize the oxygen inhibition. (A) Conversion vs time plot from real time FTIR spectroscopy. (B) Corresponding GPC traces of the polymers at the end of the irradiation (150 s).

Table S9. Summary of **Figure S24A**. Kinetics of ethyl acrylate homopolymerization with various CTAs and BAPO. [Initiator] = 0.1 mol% for 150 s light irradiation. Values are averages ($n \geq 3$) with ±1 standard deviation from the mean provided.

Initiator	LED (nm)	Intensity (mW/cm^2)	Initial Rate (%s^{-1})	Initial Rate (mM/s)	ρ_{max} in run time (%)	Inhibition time t_{inhib} (s)
CTA 1	365	60	0.33 ± 0.01	31 ± 1	52.23 ± 0.06	5.0 ± 0.4
CTA 2	365	60	0.446 ± 0.006	41.8 ± 0.5	77.0 ± 0.2	3.0 ± 0.1
CTA 3	365	60	0.427 ± 0.009	40.1 ± 0.9	68.7 ± 0.4	3.3 ± 0.6
CTA 4	405	60	0.53 ± 0.02	50 ± 1	83 ± 1	2.0 ± 0.3
CTA 5	405	60	0.51 ± 0.02	48 ± 2	74 ± 1	15 ± 1
BAPO	365	5	1.28 ± 0.05	120 ± 4	98.5 ± 0.3	2.2 ± 0.5

Table S10. Summary of **Figure S24B**. GPC results of poly(ethyl acrylate), relative to polystyrene standards, prepared using various CTAs or BAPO at an [Initiator] = 0.1 mol% and light irradiation time of 150 s.

Initiator	[CTA] (mol%)	Wavelength (nm)	Intensity (mW/cm^2)	$M_{n,GPC}$ (kDa)	M_w/M_n	Conv.[a] (%)	$M_{n,thereo.}$[b] (kDa)
CTA 1	0.1	365	60	48.9	1.35	52	53
CTA 2	0.1	365	60	63.8	1.34	77	77
CTA 3	0.1	365	60	57.8	1.32	67	69
CTA 4	0.1	405	60	67.4	1.23	83	83
CTA 5	0.1	405	60	67.6	1.25	74	74
BAPO	0.1	365	5	98.5	2.14	99	/

[a.] Monomer conversion is obtained from real-time FTIR spectroscopy

[b.] $M_{n,thereo.}$ was estimated based on monomer conversion

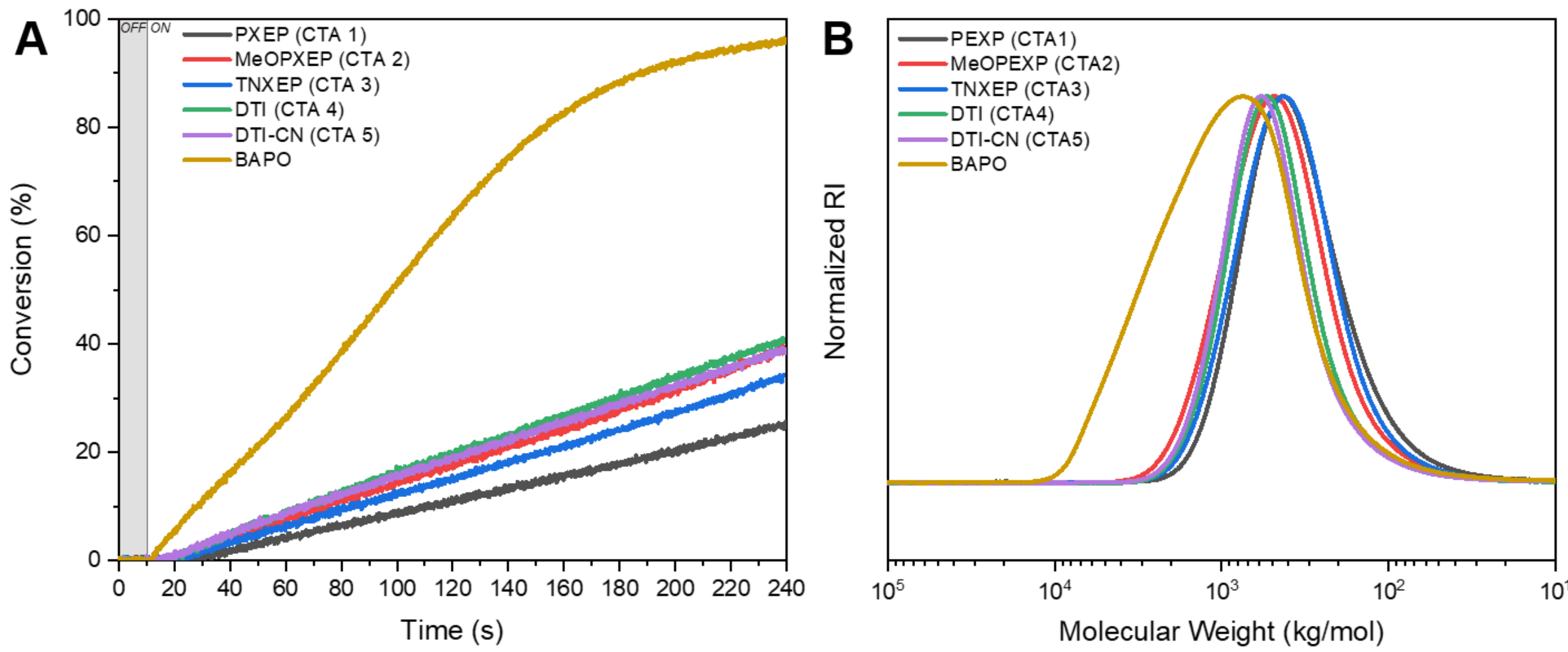

Figure S25. Photoiniferter polymerization of ethyl acrylate with different CTAs (0.01 mol%) under 60 mW/cm^2 365 nm LED (CTA1-3) or under 60 mW/cm^2 405 nm LED (CTA4-5) exposure. As a control, free radical polymerization with [BAPO] = 0.01 mol% under 5 mW/cm^2 365 nm LED is provided. PPh_3 (0.1 wt%) was added to minimize the oxygen inhibition. (A) Conversion vs time plot from real time FTIR spectroscopy. (B) Corresponding GPC traces of the polymers at the end of the irradiation (230 s).

Table S11. Summary of **Figure S25A**. Kinetics of ethyl acrylate homopolymerization with various CTAs and BAPO. [Initiator] = 0.01 mol% for 230 s light irradiation. Values are averages ($n \geq 3$) with ±1 standard deviation from the mean provided.

Initiator	LED (nm)	Intensity (mW/cm^2)	Initial Rate ($\% s^{-1}$)	Initial Rate (mM/s)	ρ_{max} in run time (%)	Inhibition time t_{inhib} (s)
CTA 1	365	60	0.113 ± 0.003	10.6 ± 0.2	25.1 ± 0.3	20.0 ± 0.6
CTA 2	365	60	0.180 ± 0.003	16.9 ± 0.2	39.7 ± 0.5	10.4 ± 0.9
CTA 3	365	60	0.155 ± 0.003	14.6 ± 0.2	36.6 ± 2	13 ± 1
CTA 4	405	60	0.205 ± 0.002	19.3 ± 0.2	40.9 ± 0.2	9.4 ± 0.2
CTA 4	415	60	0.182 ± 0.007	17.1 ± 0.7	38.1 ± 0.9	13.4 ± 0.6
CTA 5	405	60	0.192 ± 0.001	18.07 ± 0.05	39.0 ± 0.3	8 ± 1
CTA 5	415	60	0.176 ± 0.005	16.6 ± 0.5	38.3 ± 0.2	8 ± 1
BAPO	365	5	0.58 ± 0.01	54 ± 1	93.6 ± 0.1	3.3 ± 0.2

Table S12. Summary of **Figure S25B**. GPC results of poly(ethyl acrylate), relative to polystyrene standards, prepared using various CTAs or BAPO at an [initiator] = 0.01 mol% and light irradiation time of 230 s.

Initiator	[CTA] (mol%)	Wavelength (nm)	Intensity (mW/cm^2)	$M_{n,GPC}$ (kDa)	M_w/M_n	Conv.[a] (%)	$M_{n,thereo.}$[b] (kDa)
CTA 1	0.01	365	60	266.0	1.66	25	252
CTA 2	0.01	365	60	327.1	1.74	40	398
CTA 3	0.01	365	60	290.7	1.66	37	367
CTA 4	0.01	405	60	350.4	1.63	41	410
CTA 5	0.01	405	60	379.3	1.61	39	391
BAPO	0.01	365	5	516.1	2.53	94	/

[a.] Monomer conversion is obtained from real-time FTIR spectroscopy

[b.] $M_{n,thereo.}$ was estimated based on monomer conversion

S2.4.2 EA polymerization with TNXEP (CTA3) at varying conditions

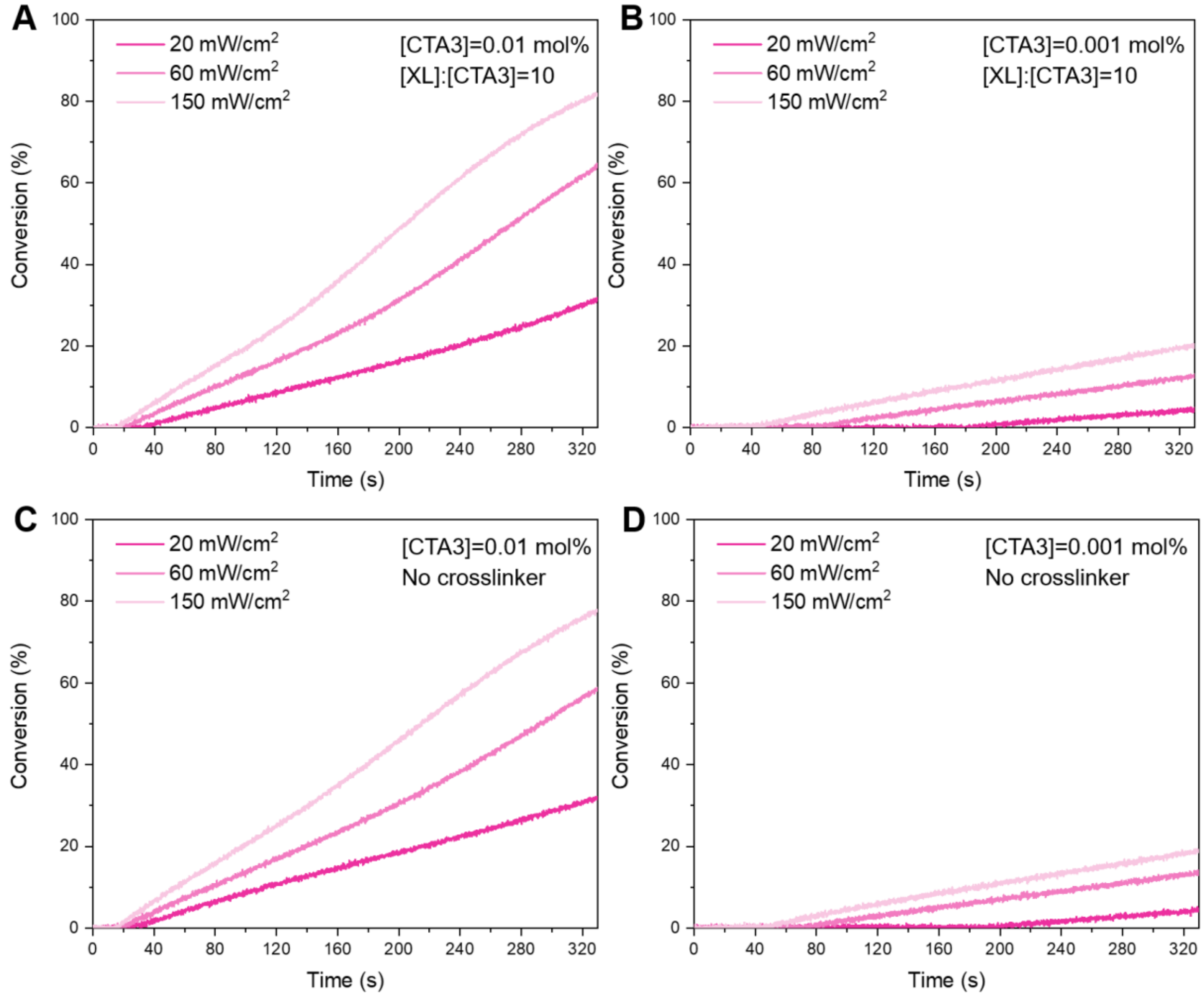

Figure S26. Ethyl acrylate homopolymerization initiated by CTA3. Conversion vs time plot obtained via real-time FTIR under varying intensity of 365 nm LED. PPh_3 (0.1 wt%) was added to minimize the oxygen inhibition. (A) [CTA3] = 0.01 mmol%, [XL] = 0.1 mol%. (B) [CTA3] = 0.001 mmol%, [XL] = 0.01 mol%. (C) [CTA3] = 0.01 mmol%, no crosslinker. (D) [CTA3] = 0.001 mmol%, no crosslinker.

Table S13. Summary of **Figure S26**. Kinetics of ethyl acrylate homopolymerization with CTA3 at varying [CTA3] concentration under varying intensities of 365 nm LED exposure (320 s) and a sample thickness of 100 µm. PPh3 (0.1 wt%) was added to minimize the oxygen inhibition. Values are averages ($n \geq 3$) with ±1 standard deviation from the mean provided.

[CTA3] (mol%)	[XL]: [CTA]	Intensity (mW/cm^2)	Initial Rate (%s^{-1})	Initial Rate (mM/s)	ρ_{max} in run time (%)	Inhibition time t_{inhib} (s)
0.01	0	20	0.10 ± 0.01	9 ± 1	30 ± 1	26 ± 3
		60	0.156 ± 0.003	14.7 ± 0.2	58.9 ± 0.4	12.2 ± 0.6
		150	0.241 ± 0.004	22.6 ± 0.4	77.9 ± 0.2	7.7 ± 0.3
	10	20	0.093 ± 0.003	8.7 ± 0.2	31.8 ± 0.2	27 ± 2
		60	0.166 ± 0.005	15.6 ± 0.5	65.4 ± 0.7	12.2 ± 0.8
		150	0.236 ± 0.009	22.1 ± 0.8	82.8 ± 0.5	7.4 ± 0.8
0.001	0	20	0.018 ± 0.001	1.67 ± 0.08	4.1 ± 0.3	231 ± 8
		60	0.035 ± 0.004	3.1 ± 0.4	13.0 ± 0.4	83 ± 2
		150	0.069 ± 0.008	6.5 ± 0.8	20.0 ± 0.6	44 ± 2
	10	20	0.021 ± 0.003	2.0 ± 0.3	4.3 ± 0.5	214 ± 9
		60	0.041 ± 0.001	3.8 ± 0.1	12.2 ± 0.5	96 ± 3
		150	0.068 ± 0.004	6.3 ± 0.4	20.2 ± 0.2	40.8 ± 0.9

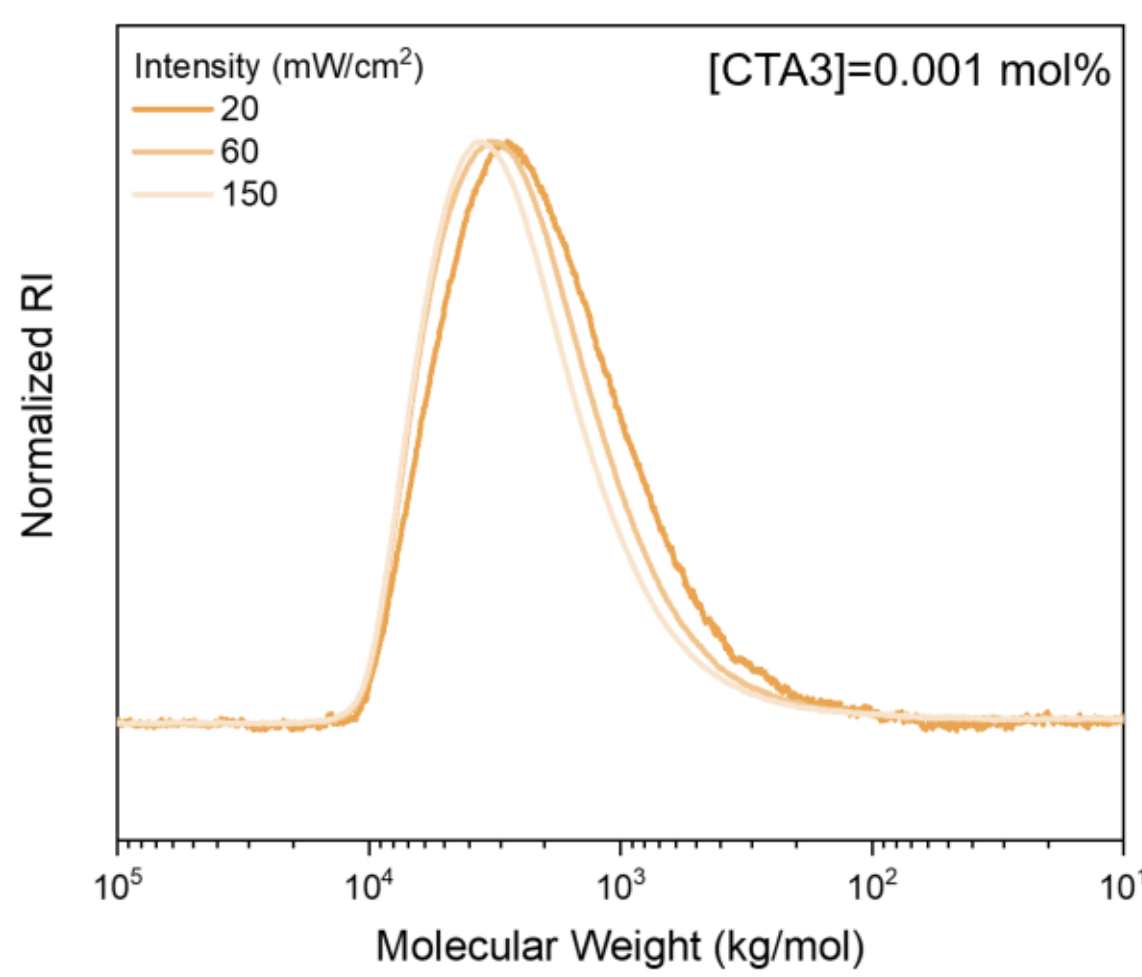

Figure S27. GPC traces of poly(ethyl acrylate) prepared using [CTA3] = 0.001 mol% under 365 nm LED irradiation (320s) at varying intensities. [PPh_3] = 0.1 wt%.

Table S14. Summary of **Figure 2D** and **Figure S27**. GPC results of poly(ethyl acrylate), relative to polystyrene standards, prepared using [CTA3] = 0.01 or 0.001 mol% under 365 nm LED for 320s at varying intensity. [PPh_3] = 0.1 wt%.

Initiator	[Initiator] (mol%)	Intensity (mW/cm^2)	$M_{n,GPC}$ (kDa)	M_w/M_n	Conv.[a] (%)	$M_{n,thereo.}$[b] (kDa)
CTA3	0.01	20	330.2	1.61	30	304
		60	509.3	1.65	59	590
		150	703.1	1.98	78	780
	0.001	20	1452.4	1.84	4	407
		60	1606.8	1.86	13	1305
		150	1804.6	1.77	20	2002

a. Monomer conversion is obtained from real-time FTIR spectroscopy

b. $M_{n,thereo.}$ was estimated based on monomer conversion

S2.5 Kinetics of EA polymerization with conventional free radical initiator

The reaction kinetics for free radical polymerizations of ethyl acrylate were characterized using real time Fourier transform infrared (FTIR) spectroscopy in a transmission configuration. The polymerization rate was calculated from the initial slope of the conversion vs. time plots from the slope between 3% to 10% ethyl acrylate conversion. Additionally, t_{inhib} was determined as the time required to reach 3% monomer (C=C–H) conversion, excluding the initial 10 seconds before the LED was turned on. ρ_{max} was determined as the max conversion at the end of light irradiation.

BAPO depletion during irradiation was estimated using a discrete numerical model based on the initial BAPO concentration, the measured BAPO UV–vis absorbance spectrum, the 365 nm LED emission profile, and a BAPO dissociation quantum yield of 0.6.[S11,12] The raw absorbance spectrum was converted to molar absorptivity using the Beer–Lambert relationship, and the LED spectrum was normalized to the applied light intensity. At each 1 s time increment, the wavelength-dependent absorbance of the formulation was recalculated from the remaining BAPO concentration, and the fraction of absorbed photons was determined from the overlap between the LED emission profile and the BAPO absorption spectrum. The number of BAPO molecules consumed at each time point was calculated from the total absorbed photons, and the remaining BAPO concentration was updated iteratively for the next time step. The calculation assumed a 1 cm² irradiation area, 100 µm optical pathlength.

Table S15. Summary of **Figure 2E**. Kinetics of ethyl acrylate homopolymerization with [BAPO] = 0.01 mol% without crosslinker under 365 nm LED (320 s irradiation) with varying light intensity. Values are averages ($n \geq 3$) with ±1 standard deviation from the mean provided.

[XL]:[BAPO]	Intensity (mW/cm^2)	Initial Rate (%s^{-1})	Initial Rate (mM/s)	ρ_{max} in run time (%)	Inhibition time t_{inhib} (s)
0	1	0.27 ± 0.01	25 ± 1	98.9 ± 0.6	9.7 ± 0.3
	5	0.58 ± 0.01	54 ± 1	98.53 ± 0.07	3.3 ± 0.2
	10	0.82 ± 0.02	77 ± 2	77.3 ± 0.6	1.9 ± 0.2
	20	1.09 ± 0.04	102 ± 4	49 ± 1	0.8 ± 0.1
	60	1.5 ± 0.1	142 ± 13	30.0 ± 0.1	0.4 ± 0.1

Table S16. Summary of **Figure 2G**. GPC results of poly(ethyl acrylate), relative to polystyrene standards, prepared using [BAPO] = 0.01 mol% under 365 nm LED for 320s at varying light intensity. [PPh_3] = 0.1 wt%.

Intensity (mW/cm^2)	$M_{n,GPC}$ (kDa)	M_w/M_n	Conv.[a] (%)
1	822.7	2.32	99
5	678.7	2.78	99
10	1071.2	2.62	77
20	940.7	2.63	49
60	936.0	3.11	30

a. Monomer conversion is obtained from real-time FTIR spectroscopy

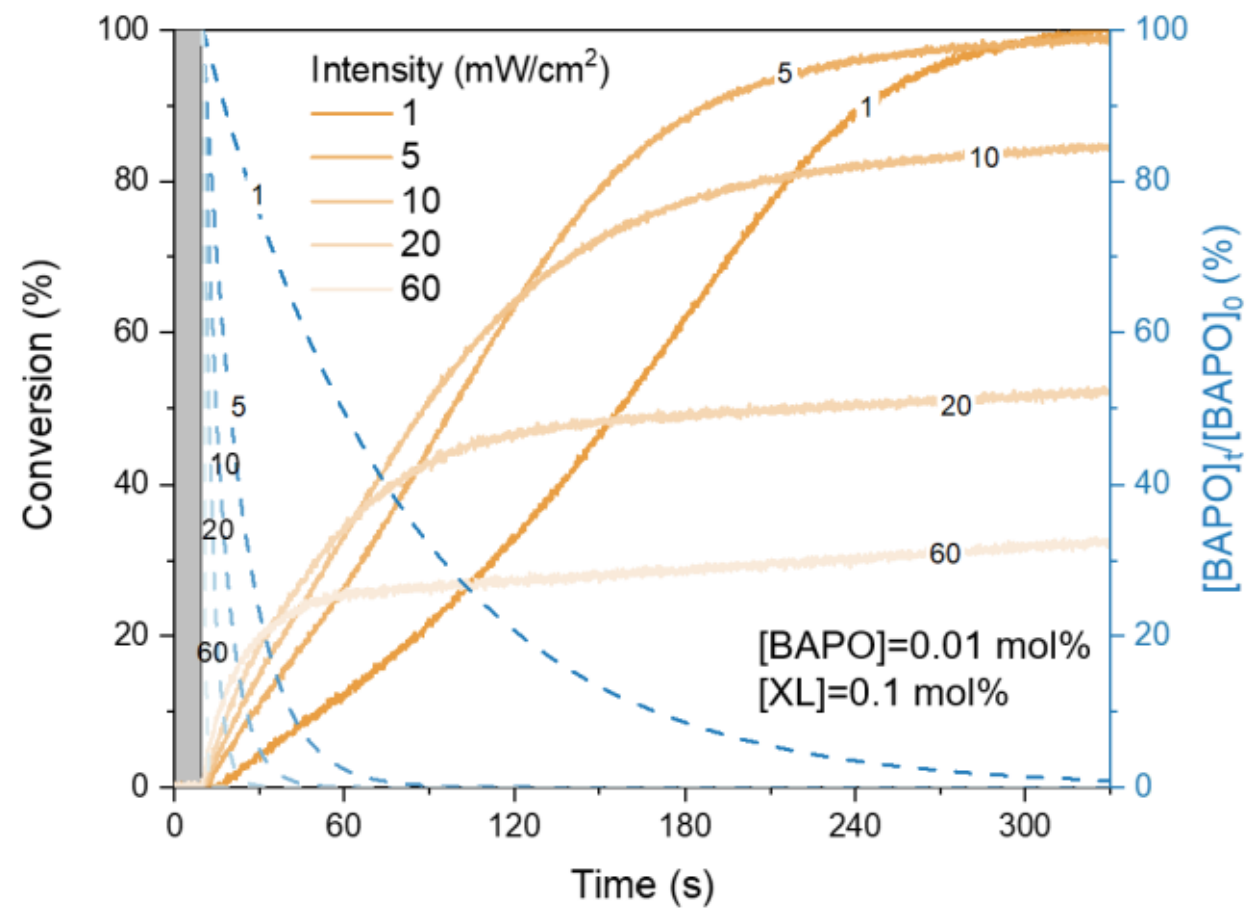

Figure S28. Conversion vs time plot of ethyl acrylate polymerization using [BAPO] = 0.01 mol% with crosslinker under 365 nm LED with varying intensities for 320 s of irradiation (solid orange lines). Simulated BAPO depletion as a function of exposure time under different light intensities and initial BAPO concentration $[BAPO]_0$ = 0.9389 mM, corresponding to 0.01 mol% BAPO in ethyl acrylate (blue dashed lines). [BAPO] = 0.01 mol%, [crosslinker] = 0 mol%, $[PPh_3]$ = 0.1 wt%. (B) [BAPO] = 0.01 mol%, [crosslinker] = 0.1 mol%, $[PPh_3]$ = 0.1 wt%.

Table S17. Summary of **Figure S28**. Kinetics of ethyl acrylate homopolymerization using [BAPO] = 0.01 mol% with crosslinker under 365 nm LED exposure at varying intensities for 320 s of irradiation. Values are averages ($n \geq 3$) with ±1 standard deviation from the mean provided.

[XL]: [BAPO]	Intensity (mW/cm^2)	Initial Rate ($\%s^{-1}$)	Initial Rate (mM/s)	ρ_{max} in run time (%)	Inhibition time t_{inhib} (s)
	1	0.276 ± 0.002	25.9 ± 0.2	99.82 ± 0.07	9 ± 1
	5	0.59 ± 0.01	55.1 ± 0.9	99.7 ± 0.5	2.8 ± 0.2
10	10	0.83 ± 0.03	78 ± 3	84.8 ± 0.3	1.5 ± 0.1
	20	1.08 ± 0.06	101 ± 6	52.5 ± 0.2	0.7 ± 0.4
	60	1.6 ± 0.2	154 ± 19	32.2 ± 0.4	0.2 ± 0.1

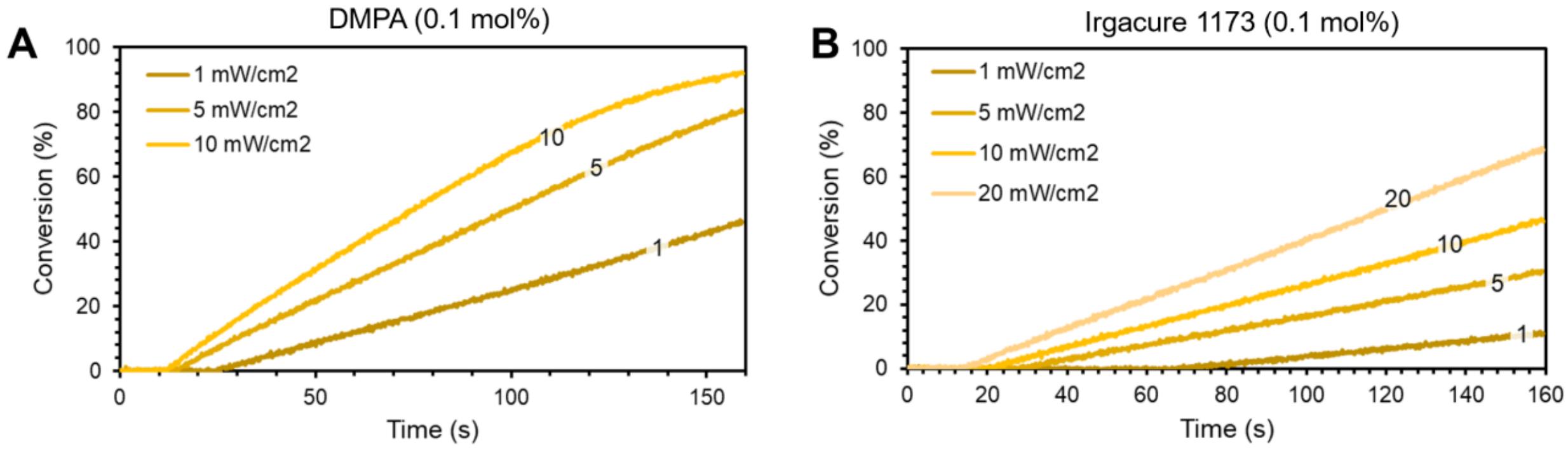

Figure S29. Conversion vs time plot of ethyl acrylate homopolymerizations using [initiator] = 0.1 mol%, 0.1 wt% PPh_3 under 365 nm LED with varying intensities. (A). 2,2-Dimethoxy-2-phenylacetophenone (DMPA) as the initiator (B). Irgacure 1173 as the initiator.

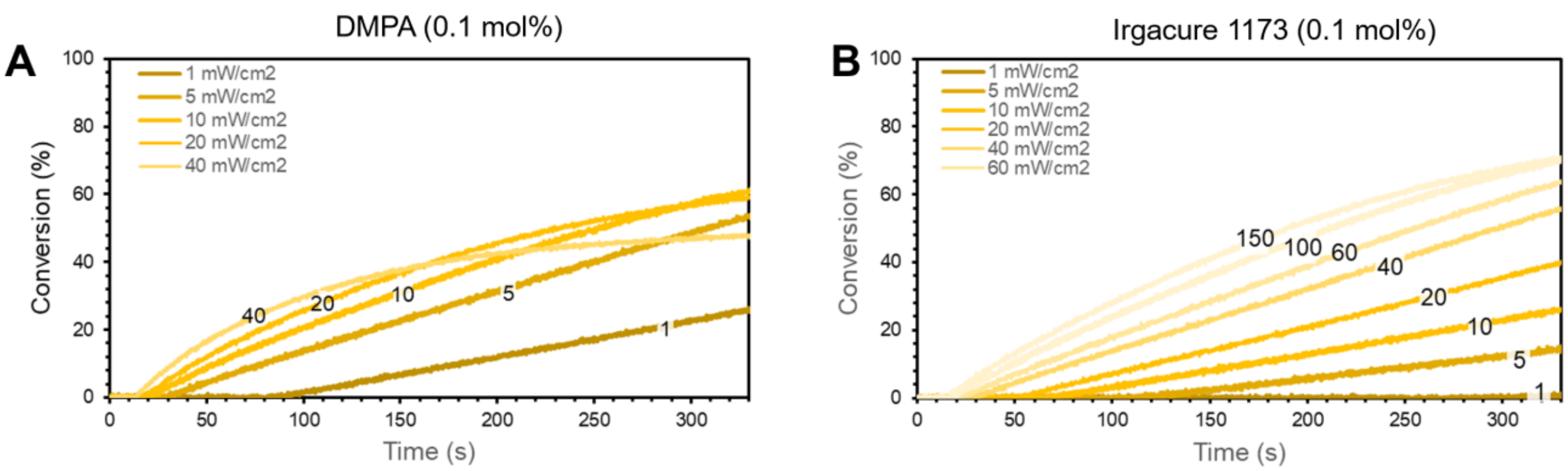

Figure S30. Conversion vs time plot ethyl acrylate homopolymerizations using [initiator] = 0.01 mol%, 0.1 wt% PPh_3 under 365 nm LED with varying intensities. (A). 2,2-Dimethoxy-2-phenylacetophenone (DMPA) as the initiator (B). Irgacure 1173 as the initiator.

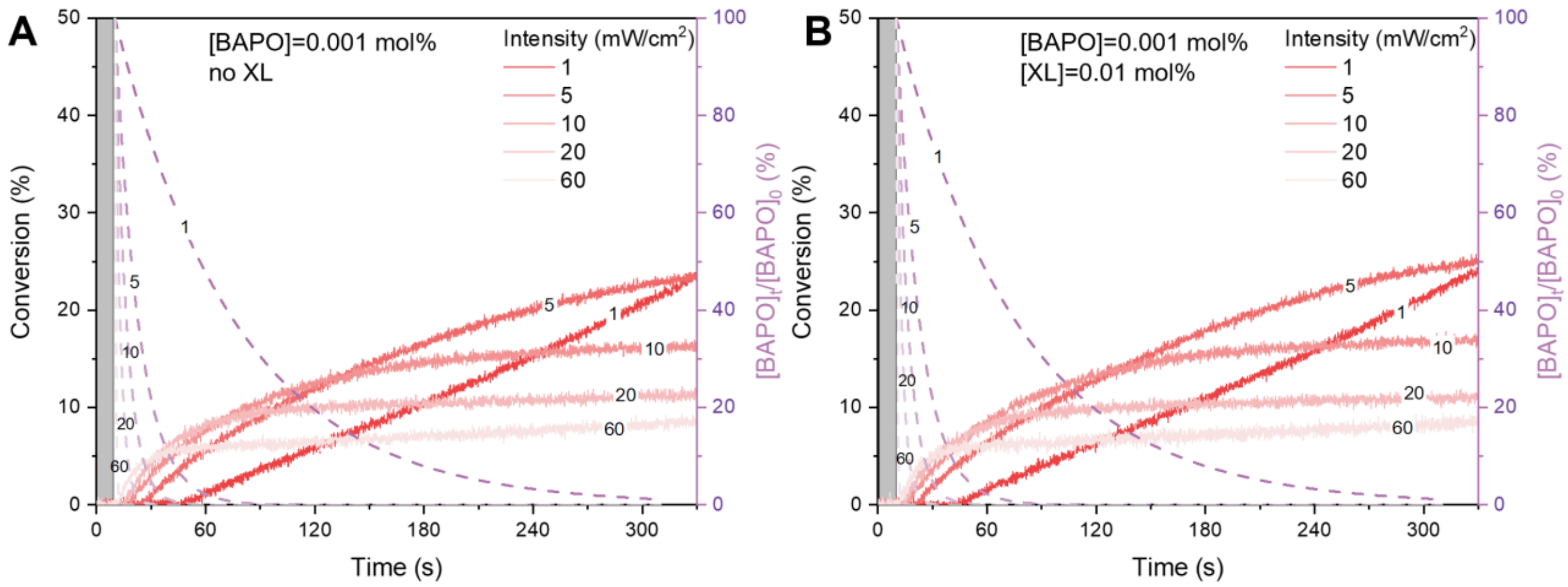

Figure S31. Conversion vs time plot of ethyl acrylate polymerizations using [BAPO] = 0.001 mol% with or without crosslinker under 365 nm LED with varying intensities for 320 s. (A) [BAPO] = 0.001 mol%, [crosslinker] = 0 mol%, $[PPh_3]$ = 0.1 wt%. (B) [BAPO] = 0.001 mol%, [crosslinker] = 0.01 mol%, $[PPh_3]$ = 0.1 wt%.

Table S18. Summary of **Figure S31**. Kinetics of ethyl acrylate homopolymerizations using [BAPO] = 0.001 mol% with or without crosslinker under 365 nm LED irradiation at varying intensities for 320 s. Values are averages ($n \geq 3$) with ±1 standard deviation from the mean provided.

[XL]: [BAPO]	Intensity (mW/cm^2)	Initial Rate ($\%s^{-1}$)	Initial Rate (mM/s)	ρ_{max} in run time (%)	Inhibition time t_{inhib} (s)
0	1	0.077 ± 0.001	7.3 ± 0.1	23.7 ± 0.1	42 ± 2
	5	0.14 ± 0.02	13 ± 2	23 ± 2	17 ± 2
	10	0.188 ± 0.004	17.6 ± 0.4	16.1 ± 0.6	11 ± 2
	20	0.23 ± 0.01	21 ± 1	11.1 ± 0.2	6.7 ± 0.1
	60	0.23 ± 0.04	22 ± 3	8.6 ± 0.4	3.0 ± 0.2
10	1	0.079 ± 0.003	7.4 ± 0.3	25 ± 1	29 ± 10
	5	0.153 ± 0.002	14.3 ± 0.2	24.89 ± 0.05	13.1 ± 0.6
	10	0.205 ± 0.004	19.2 ± 0.3	16.7 ± 0.3	8.9 ± 0.3
	20	0.240 ± 0.003	22.5 ± 0.3	11.7 ± 0.6	5.5 ± 0.7
	60	0.24 ± 0.02	23 ± 2	8.4 ± 0.1	3.0 ± 0.4

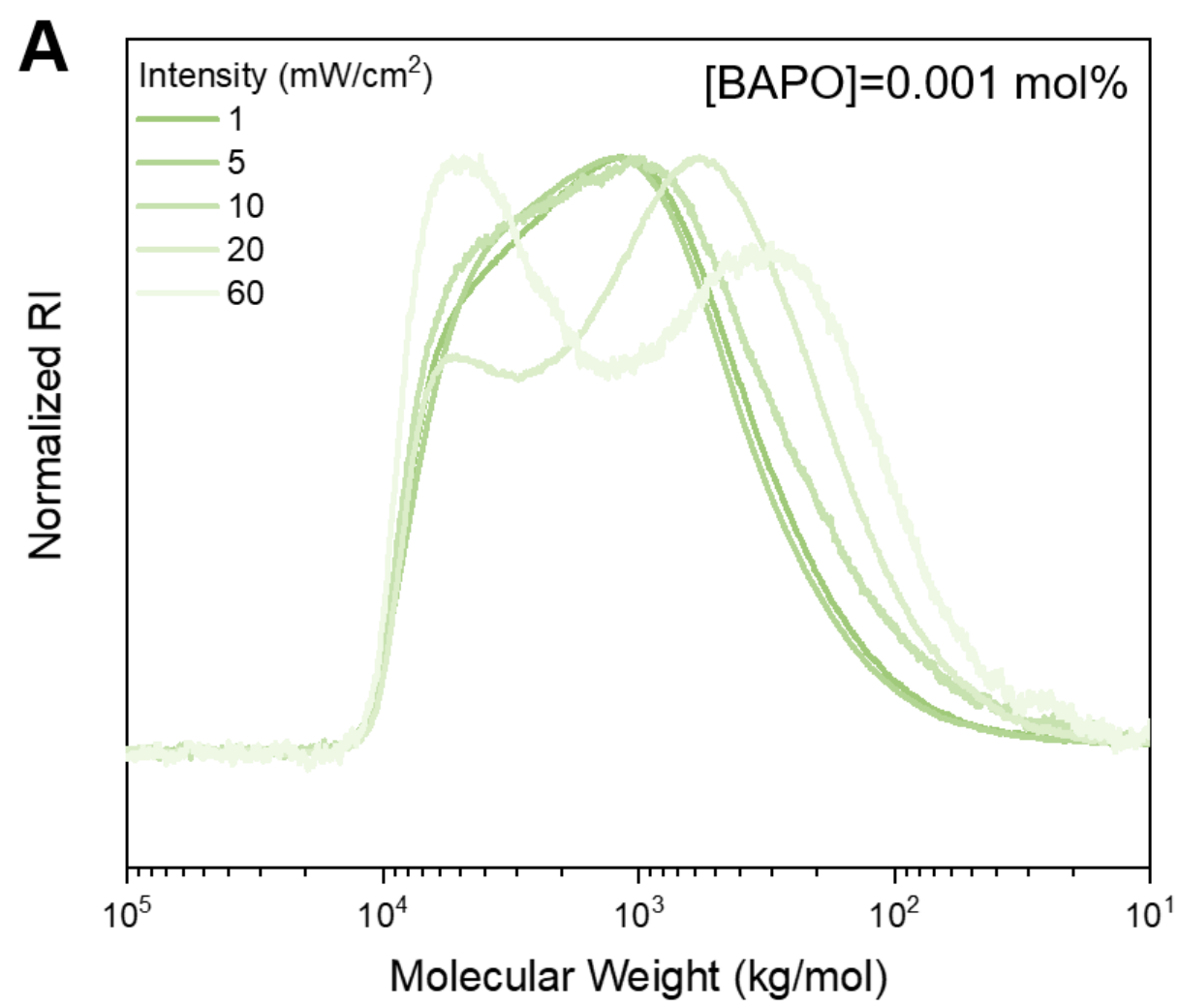

Figure S32. GPC traces of poly(ethyl acrylate), relative to polystyrene standards, prepared using [BAPO] = 0.001 mol% under 365 nm LED for 320 s at varying intensity. $[PPh_3]$ = 0.1 wt%.

Table S19. Summary of **Figure S32**. GPC results of poly(ethyl acrylate), relative to polystyrene standards, prepared using [BAPO] = 0.001 mol% under 365 nm LED for 320 s at varying intensity. $[PPh_3]$ = 0.1 wt%.

Intensity (mW/cm^2)	$M_{n,GPC}$ (kDa)	M_w/M_n	Conv.[a] (%)
1	639.4	3.08	24
5	643.0	3.07	23
10	439.6	4.21	16
20	338.0	4.49	11
60	252.7	7.01	9

c. Monomer conversion obtained from real-time FTIR spectroscopy

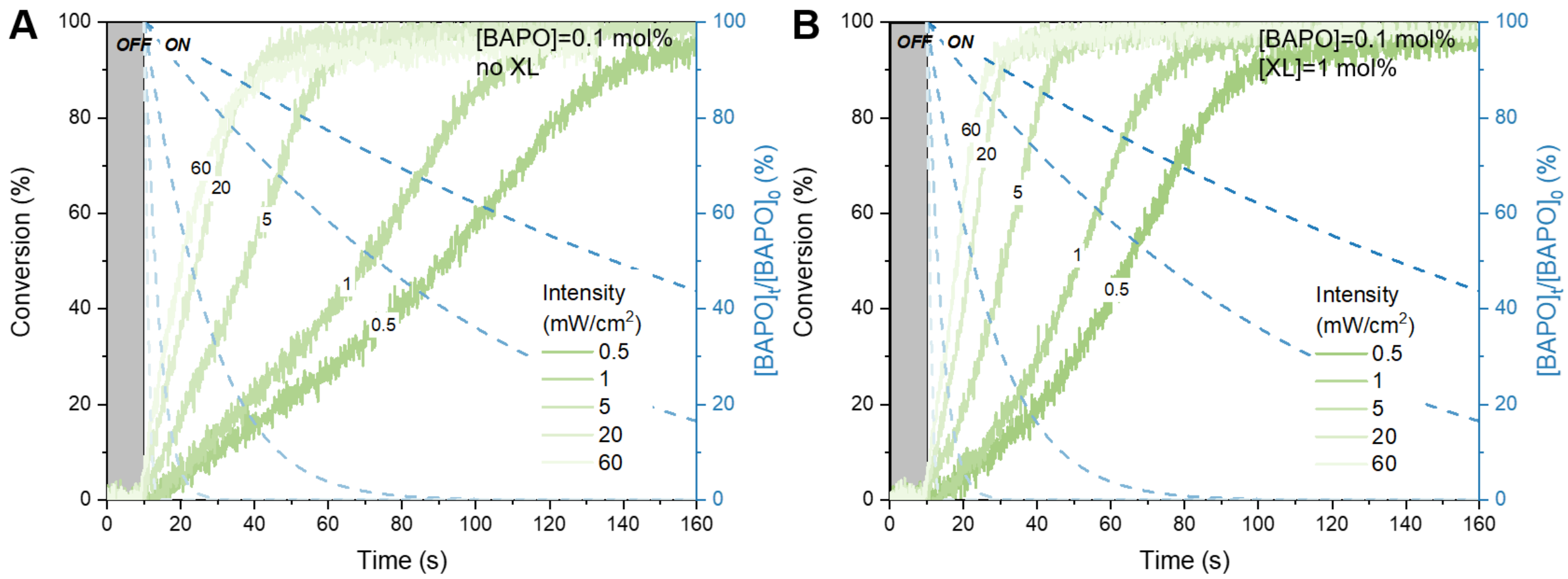

Figure S33. Conversion vs time plot of ethyl acrylate polymerizations using [BAPO] = 0.1 mol% with or without crosslinker (XL) under 365 nm LED irradiation with varying intensities for 150 s. Simulated BAPO depletion as a function of exposure time under different light intensities, initial BAPO concentration $[BAPO]_0$ = 9.389 mM, corresponding to 0.1 mol% BAPO in ethyl acrylate, and $[PPh_3]$=0.1 wt%. (A) [crosslinker] = 0 mol%. (B) [crosslinker] = 1 mol%.

Table S20. Summary of **Figure S33**. Kinetics of ethyl acrylate homopolymerization using [BAPO] = 0.1 mol% with or without crosslinker under 365 nm LED irradiation with varying intensities for 150 s. Values are averages (*n* ≥3) with ±1 standard deviation from the mean provided.

[XL]:<br>[BAPO]	Intensity<br>(mW/cm^2)	Initial Rate (%s^{-1})	Initial Rate (mM/s)	ρ_{max} in<br>run time (%)	Inhibition time<br>t_{inhib} (s)
0	0.5	0.42 ± 0.01	39 ± 1	97 ± 1	4 ± 1
	1	0.61 ± 0.01	57 ± 1	95.6 ± 0.1	5.7 ± 0.5
	5	1.28 ± 0.05	120 ± 4	98.5 ± 0.3	2.2 ± 0.5
	20	2.7 ± 0.3	252 ± 26	98.5 ± 0.4	0.15 ± 0.06
	60	4.3 ± 0.3	403 ± 28	95.2 ± 0.4	0.19 ± 0.07
10	0.5	0.54 ± 0.04	50 ± 4	98.2 ± 0.9	5.5 ± 0.4
	1	0.71 ± 0.03	66 ± 2	97.6 ± 0.5	6.1 ± 0.7
	5	1.3 ± 0.1	122 ± 12	99.9 ± 0.6	1.1 ± 0.2
	20	2.68 ± 0.09	251 ± 8	98.9 ± 0.1	0.58 ± 0.09
	60	4.9 ± 0.3	462 ± 32	98.4 ± 0.4	0.23 ± <0.01

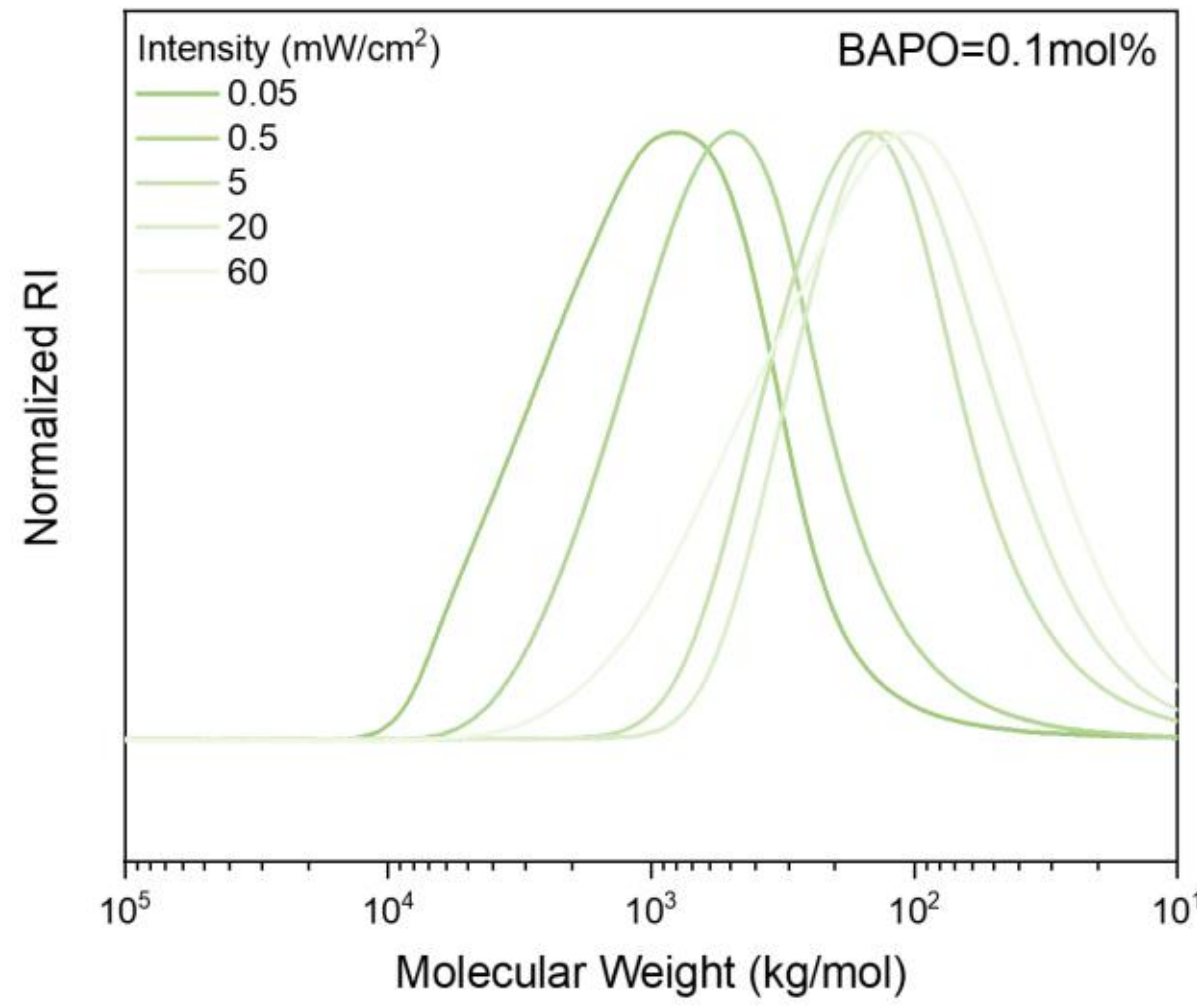

Figure S34. GPC traces of poly(ethyl acrylate), relative to polystyrene standards, prepared using [BAPO] = 0.1 mol% under 365 nm LED irradiation for 320 s at varying light intensity. [PPh_3] = 0.1 wt%.

Table S21. Summary of **Figure S34**. GPC results of poly(ethyl acrylate), relative to polystyrene standards, prepared using [BAPO] = 0.1 mol% under 365 nm LED irradiation for 320 s at varying light intensity. [PPh_3] = 0.1 wt%.

Intensity (mW/cm^2)	$M_{n,GPC}$ (kDa)	M_w/M_n	Conv.[a] (%)
0.05	529.7	2.14	97
0.5	304.7	2.02	96
5	93.8	1.74	99
20	71.1	1.74	99
60	63.4	4.20	95

a. Monomer conversion is obtained from real-time FTIR spectroscopy

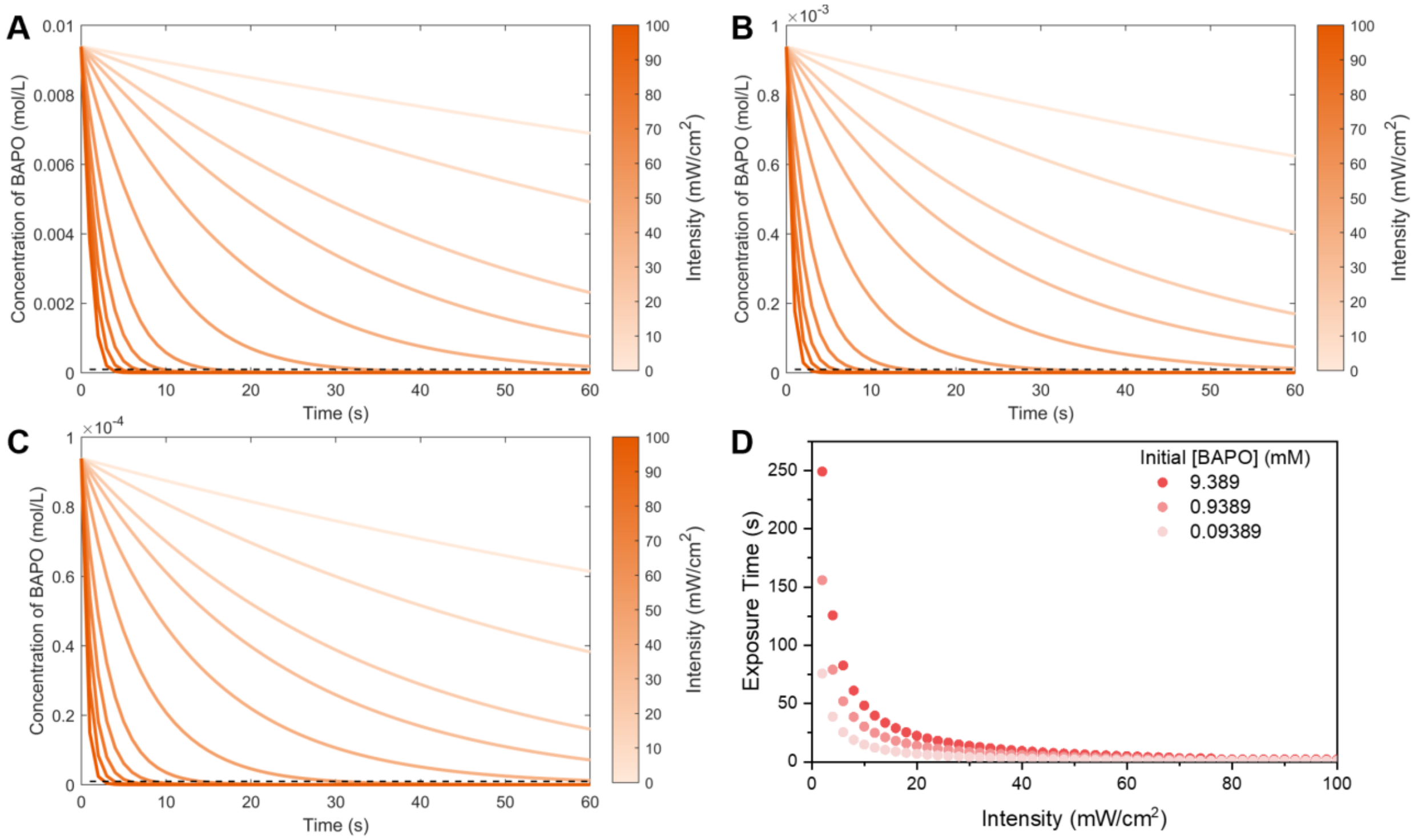

Figure S35. Simulated depletion of [BAPO] as a function of exposure time under different light intensities and initial BAPO concentrations with a dashed line representing remaining [BAPO] = 0.01 mM: (A) $[BAPO]_0$ = 9.389 mM, corresponding to 0.1 mol% BAPO in ethyl acrylate); (B) $[BAPO]_0$ = 0.9389 mM, corresponding to 0.01 mol% BAPO in ethyl acrylate; and (C) $[BAPO]_0$ = 0.09389 mM, corresponding to 0.001 mol% BAPO in ethyl acrylate. (D) Calculated exposure time required for [BAPO] to decrease to 0.01 mM as a function of light intensity for each initial BAPO concentration.

S2.6 Casted EA elastomers Film

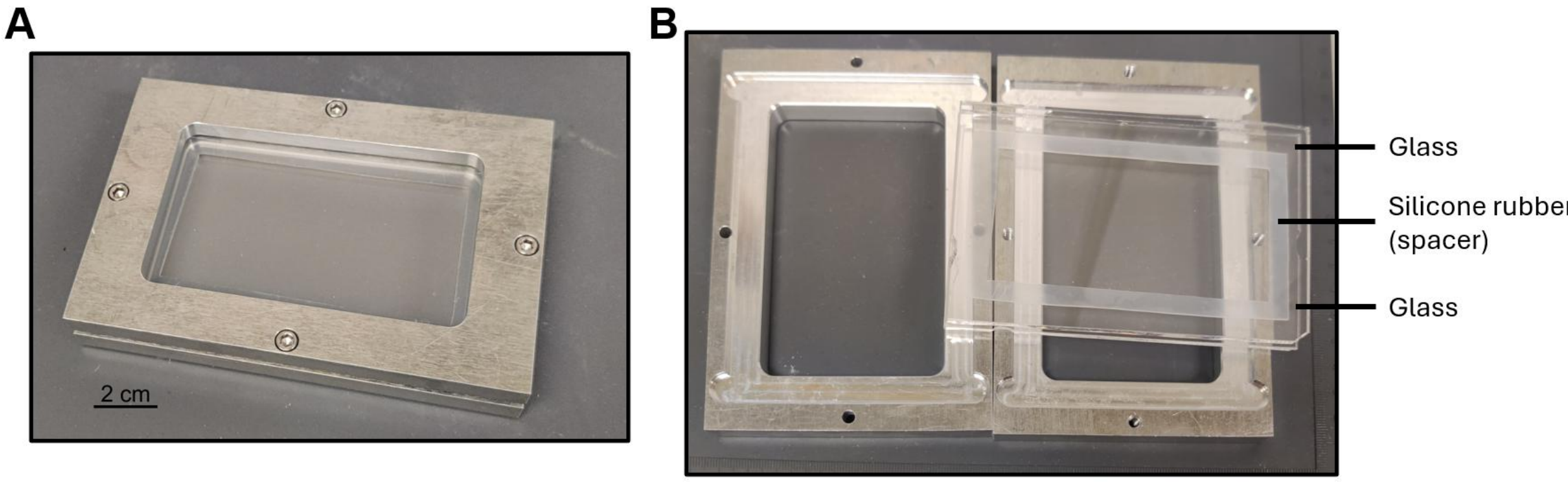

Figure S36. (A) Photo of the assembled mold used for film casting. (B) Disassembled view of the mold, showing the internal glass–silicone rubber spacer–glass sandwich configuration used for resin injection and film casting. Silicone rubber spacer: McMaster-Carr, extreme-temperature silicone rubber, translucent, plain back, 1/32" thick (catalog #86465K21).

Table S22. Sol-gel fraction obtained via different drying/washing process. For samples: varying [BAPO] concentration at a constant [XL]:[BAPO] = 10. PPh_3 (0.1 wt%) was added to minimize the oxygen inhibition. For samples with a [BAPO] = 0.1 and 0.01mol%, the films are cast under 5 mW/cm^2 365 nm LED exposure for 5 min. For samples with a [BAPO] = 0.001 mol%, the films are cast under 1 mW/cm^2 365 nm LED exposure for 20 min. Sol-gel fractions given as gel content. Values are averages ($n \geq 3$) with ±1 standard deviation from the mean provided.

[BAPO] (mol%)	Sol-Gel Fraction (vacuum dried)	Swelling Ratio (in ethyl acetate)	Sol-Gel Fraction (swell then vacuum dried)
0.1	0.98 ± 0.01	3.3 ± 0.1	0.95 ± 0.02
0.01	0.94 ± 0.02	5.9 ± 0.4	0.92 ± 0.02
0.001	0.84 ± 0.02	7.8 ± 0.1	0.80 ± 0.02

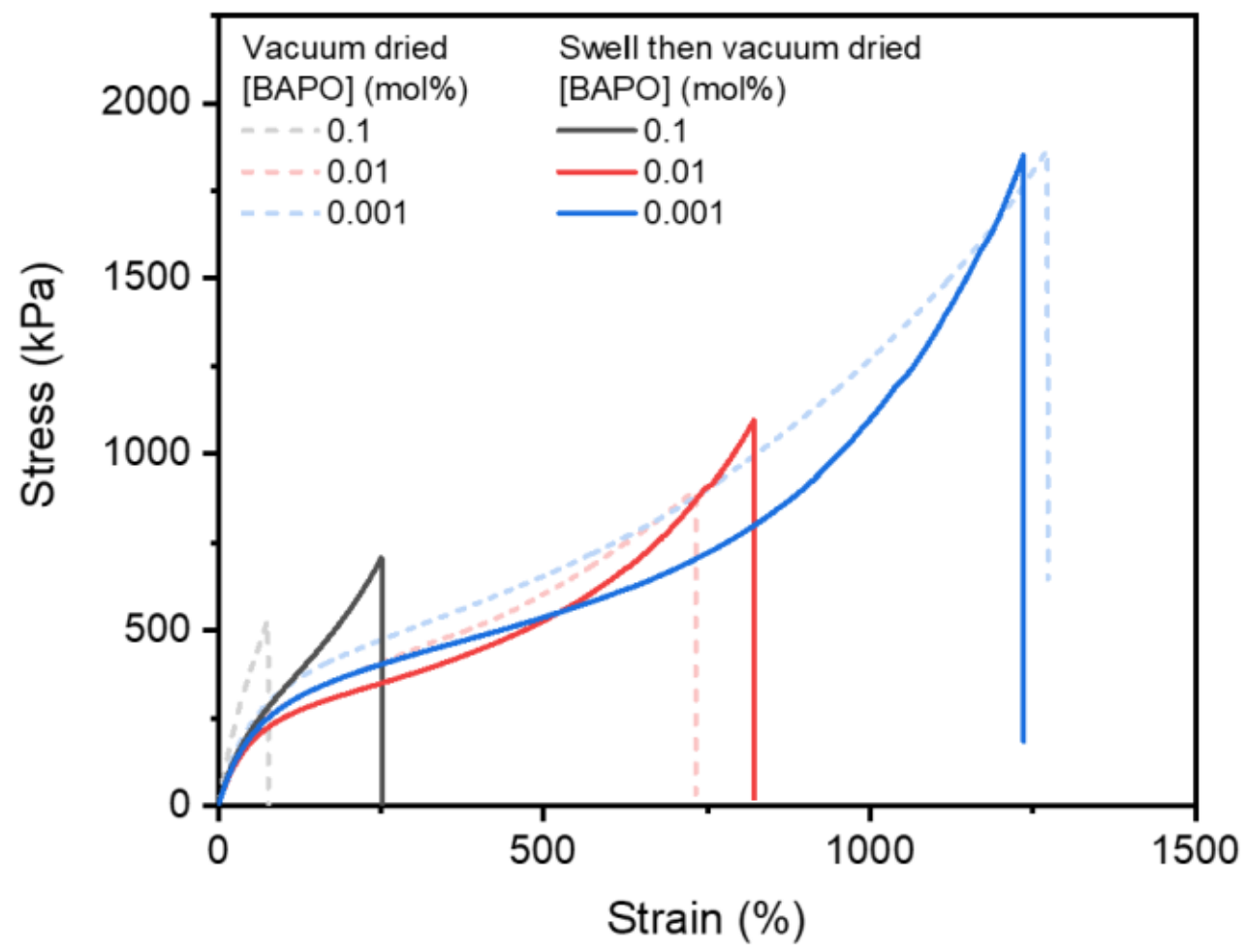

Figure S37. Representative uniaxial tensile stress-strain curves of entangled poly(ethyl acrylate) elastomers dried under different conditions: Vacuum dried right after casting versus fully swelling in ethyl acetate then vacuum dried. Strain rate = 0.02 s^{-1}. For samples: varying [BAPO] at a constant [XL]:[BAPO] = 10. PPh_3 (0.1 wt%) was added to minimize the oxygen inhibition. For samples with a [BAPO] = 0.1 and 0.01 mol%, the films are cast under 5 mW/cm^2 365 nm LED exposure for 5 min. For samples with a [BAPO] = 0.001 mol%, the films are cast under 1 mW/cm^2 365 nm LED exposure for 20 min.

Table S23. Tabulated results from **Figure S37**. Mechanical properties of poly(ethyl acrylate) samples dried under different conditions. Values are averages ($n \geq 3$) with ±1 standard deviation from the mean provided.

Drying Conditions	[BAPO] (mol%)	Young's Modulus E_T (MPa)	Sol-Gel Fraction	Tensile Strength σ_m (MPa)	Strain at break ε_f (%)	Modulus of Toughness U_t (MJ/m^3)
vacuum dried	0.1	0.92 ± 0.01	0.98 ± 0.01	0.56 ± <0.01	97 ± 2	0.31 ± 0.01
	0.01	0.61± 0.03	0.94 ± 0.02	0.8 ± 0.2	743 ± 67	3.5 ± 0.2
	0.001	0.67 ± 0.03	0.86 ± 0.01	2.1 ± 0.6	1276 ± 172	12 ± 4
swell then vacuum dried	0.1	0.64 ± 0.04	0.95 ± 0.02	0.67 ± 0.08	249 ± 19	0.9 ± 0.1
	0.01	0.60 ± 0.01	0.92 ± 0.02	1.1 ± 0.1	819 ± 23	4.9 ± 0.3
	0.001	0.62 ± 0.01	0.80 ± 0.02	1.6 ± 0.2	1167 ± 49	7.8 ± 0.8

S2.7 Mechanical properties of EA elastomers prepared with photoiniferter

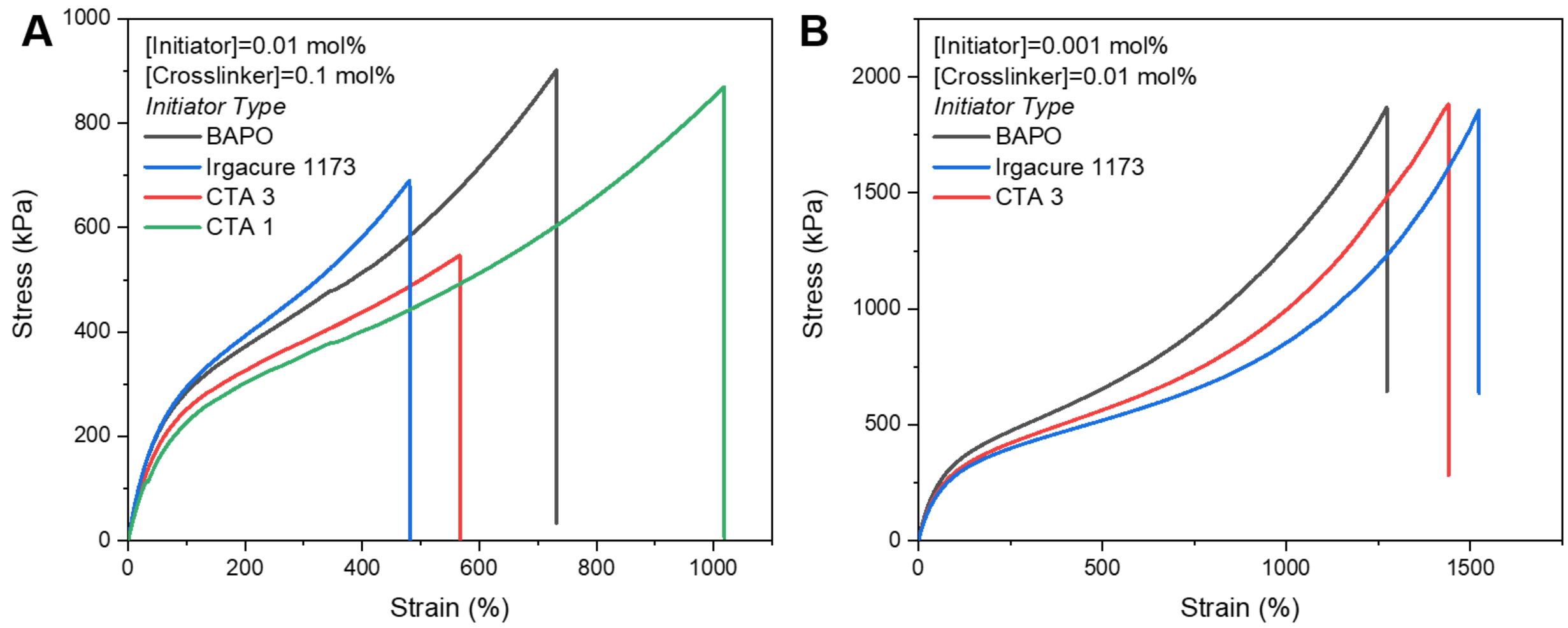

Figure S38. Representative uniaxial tensile stress and strain curves of entangled poly(ethyl acrylate) elastomers prepared using different initiators. Strain rate = 0.02 s^{-1}. (A) [Initiator] = 0.01 mol%, [crosslinker] = 0.1 mol%. Casting conditions: BAPO with 5 mW/cm^2 365 nm LED irradiation for 5 min; Irgacure 1173 and CTA with 60 mW/cm^2 365 nm LED irradiation for 10 min. (B) [Initiator] = 0.001 mol%, [crosslinker] = 0.01 mol%. Casting conditions: BAPO with 1 mW/cm^2 365 nm LED irradiation for 20 min; Irgacure 1173 and CTA3 with 60 mW/cm^2 365 nm LED irradiation for 40 min.

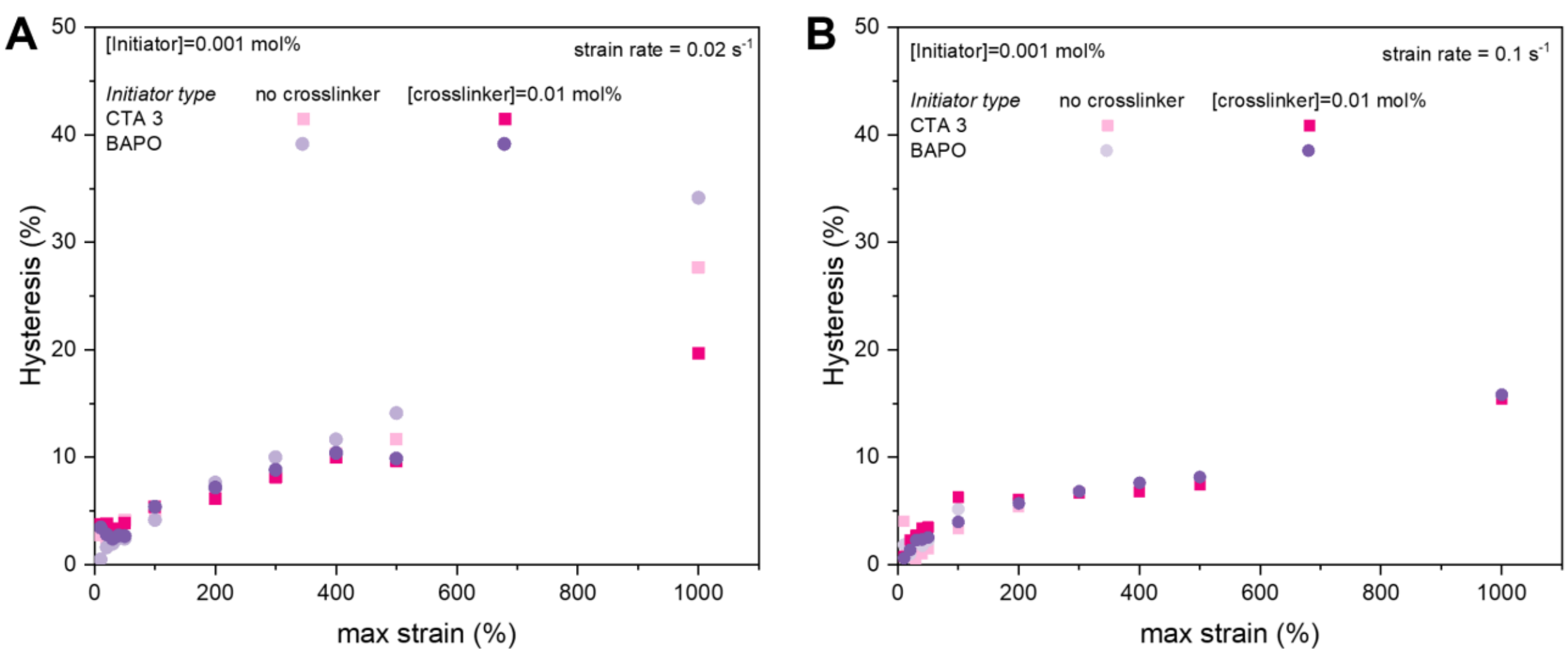

Figure S39. Hysteresis loss vs. max strain as a function of strain rate, obtained via stepwise cyclic tensile testing analysis of entangled poly(ethyl acrylate) elastomers prepared using free radical or photiniferter polymerization with and without crosslinkers. [Initiator] = 0.001 mol%, [crosslinker] = 0 or 0.01 mol%. Casting conditions: BAPO with 1 mW/cm^2 365 nm LED irradiation for 20 min; CTA 3 with 60 mW/cm^2 365 nm LED irradiation for 40 min. (A) Strain rate = 0.02 s^{-1}. (B) Strain rate = 0.1 s^{-1}

S2.8 Mechanical properties of EA elastomers prepared with free radical polymerization

Table S24. Summary of **Figure 3B**. Mechanical properties of samples prepared using variable 365nm LED intensities with 5 min of irradiation. [BAPO] = 0.01mol%, [XL]:[BAPO] = 10, PPh_3 = 0.1 wt%. Values are averages ($n \geq 3$) with ±1 standard deviation from the mean provided.

Intensity	Young's Modulus E_T (MPa)	Sol-Gel Fraction	Tensile Strength σ_m (MPa)	Strain at break ε_f (%)
5	0.61 ± 0.03	0.94 ± 0.02	0.8 ± 0.2	743 ± 67
20	0.488 ± 0.004	0.55 ± 0.02	1.8 ± 0.2	2540 ± 274
60	0.49 ± 0.03	0.34 ± 0.03	0.69 ± 0.08	2745 ± 40

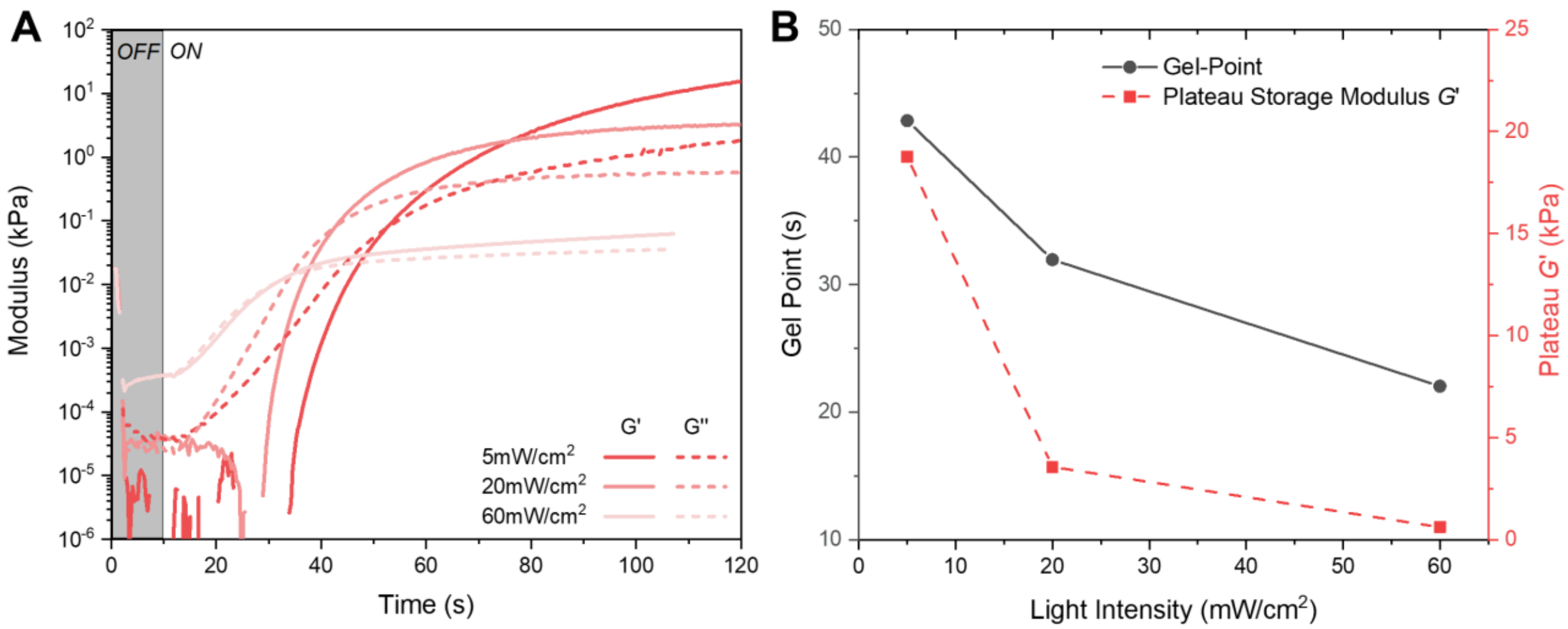

Figure S40. Photo-rheology of ethyl acrylate resins containing [BAPO] = 0.01 mol% and [XL] = 0.1 mol% under 365 nm LED irradiation with varying light intensity. PPh_3 (0.1 wt%) was added to minimize oxygen inhibition. (A) Storage modulus *G*' and loss modulus *G*'' were obtained via fast sampling to estimate the gel point as the *G*' and *G*'' crossover. LED was turned off 10 seconds after beginning the rheology measurement. (B) Corresponding gel-point and plateau *G*' within the 110 s of irradiation as a function of LED exposure intensity.

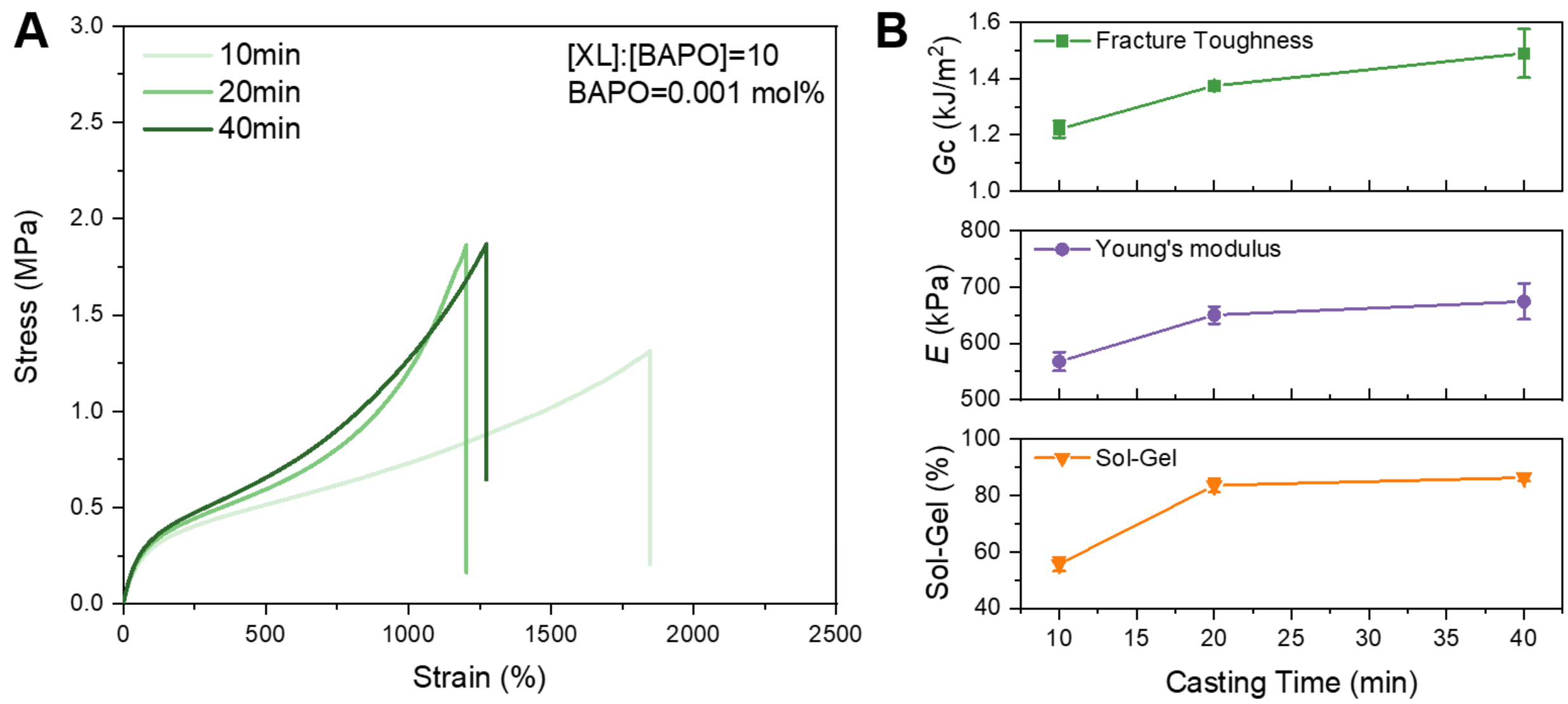

Figure S41. Influence of curing time on poly(ethyl acrylate) elastomer properties for samples prepared using [BAPO] = 0.001 mol%, [XL]:[BAPO] = 10, PPh_3 = 0.1 wt%, and 365 nm LED irradiation at 1 mW/cm^2. (A) Representative uniaxial tensile stress and strain curves. (B) Corresponding fracture toughness G_c, Young's modulus E_T, and sol-gel fraction (percent gel provided).

Table S25. Tabulated results from **Figure S41**. Mechanical properties of samples prepared using 1 mW/cm^2 365nm LED irradiation for different lengths of time. [BAPO] = 0.001mol%, [XL]:[BAPO] = 10, [PPh_3] = 0.1 wt%. Sol-gel fractions given as gel content. Values are averages ($n \geq 3$) with ±1 standard deviation from the mean provided.

Time (min)	Fracture Toughness G_c (kJ/m^2)	Young's Modulus E_T (kPa)	Sol-Gel fraction	Tensile Strength σ_m (MPa)	Strain at break ε_f (%)	Modulus of Toughness U_t (MJ/m^3)	Fracto-cohesive length $L_f = G_c / W_F$ (mm)
10	1.22 ± 0.03	599 ± 7	0.56 ± 0.02	1.2 ± 0.3	1833 ± 270	12 ± 3	0.10 ± 0.03
20	1.37 ± 0.01	673 ± 27	0.84 ± 0.02	2.1 ± 0.4	1260 ± 114	11 ± 2	0.13 ± 0.03
40	1.41 ± 0.03	715 ± 29	0.86 ± 0.01	2.1 ± 0.6	1276 ± 172	12 ± 4	0.12 ± 0.04

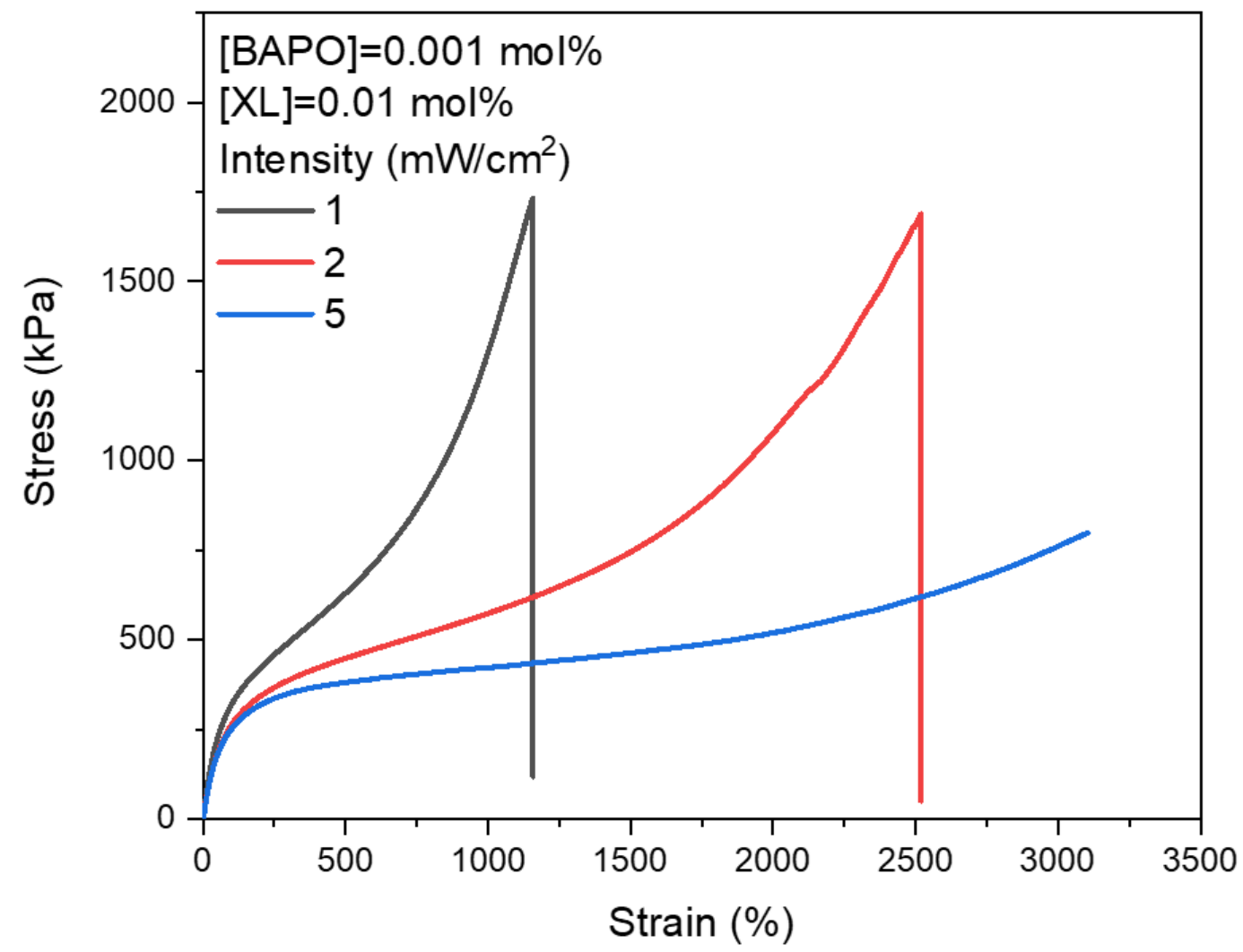

Figure S42. Representative stress vs. strain plots for entangled poly(ethyl acrylate) samples prepared as a function of 365 nm LED exposure intensity at a fixed exposure time of 20 minutes. Resins contained [BAPO] = 0.001 mol%, [XL] = 0.1 mol%, and PPh_3 = 0.1 wt%.

Table S26. Summary of **Figure 42**. Mechanical properties of poly(ethyl acrylate) samples prepared using 365nm LED exposure for 20 min. Resins contained [BAPO] = 0.001 mol%, [XL] = 0.1mol%, and PPh_3 = 0.1 wt%. Sol-gel fractions given as gel content. Values are averages ($n \geq 3$) with ±1 standard deviation from the mean provided unless noted.

Intensity	Young's Modulus E_T (MPa)	Sol-Gel Fraction	Tensile Strength σ_m (MPa)	Strain at break ε_f (%)
1	0.67 ± 0.03	0.84 ± 0.02	2.1 ± 0.4	1260 ± 114
2	0.59 ± 0.03	0.65 ± 0.03	1.7 ± 0.2	2346 ± 134
5	0.56*	0.43 ± 0.02	/	/

*Value is reported from a single tested sample owing to the low sol-gel fraction.

Table S27. Tabulated results from **Figure 3C**. Mechanical properties of poly(ethyl acrylate) samples as a function of crosslinker to BAPO ratio. Samples were prepared using [BAPO] = 0.01mol%, PPh_3 = 0.1 wt%, and 5 mW/cm^2 365 nm LED irradiation for 5 min. Sol-gel fractions given as gel content. Values are averages ($n \geq 3$) with ±1 standard deviation from the mean provided.

[BAPO] (mol%)	Young's Modulus E_T (MPa)	Sol-Gel Fraction	Tensile Strength σ_m (MPa)	Strain at break ε_f (%)	Fracture Toughness G_c (kJ/m^2)	Modulus of Toughness U_t (MJ/m^3)	Fracto-cohesive length $L_f = G_c / W_F$ (mm)
0.1	0.92 ± 0.01	0.98 ± 0.01	0.56 ± <0.01	97 ± 2	0.40 ± 0.01	0.31 ± 0.01	1.29 ± 0.05
0.01	0.61± 0.03	0.94 ± 0.02	0.8 ± 0.2	743 ± 67	1.1 ± 0.1	3.5 ± 0.2	0.34 ± 0.04
0.001	0.67 ± 0.03	0.86 ± 0.01	2.1 ± 0.6	1276 ± 172	1.49 ± 0.09	12 ± 4	0.12 ± 0.04

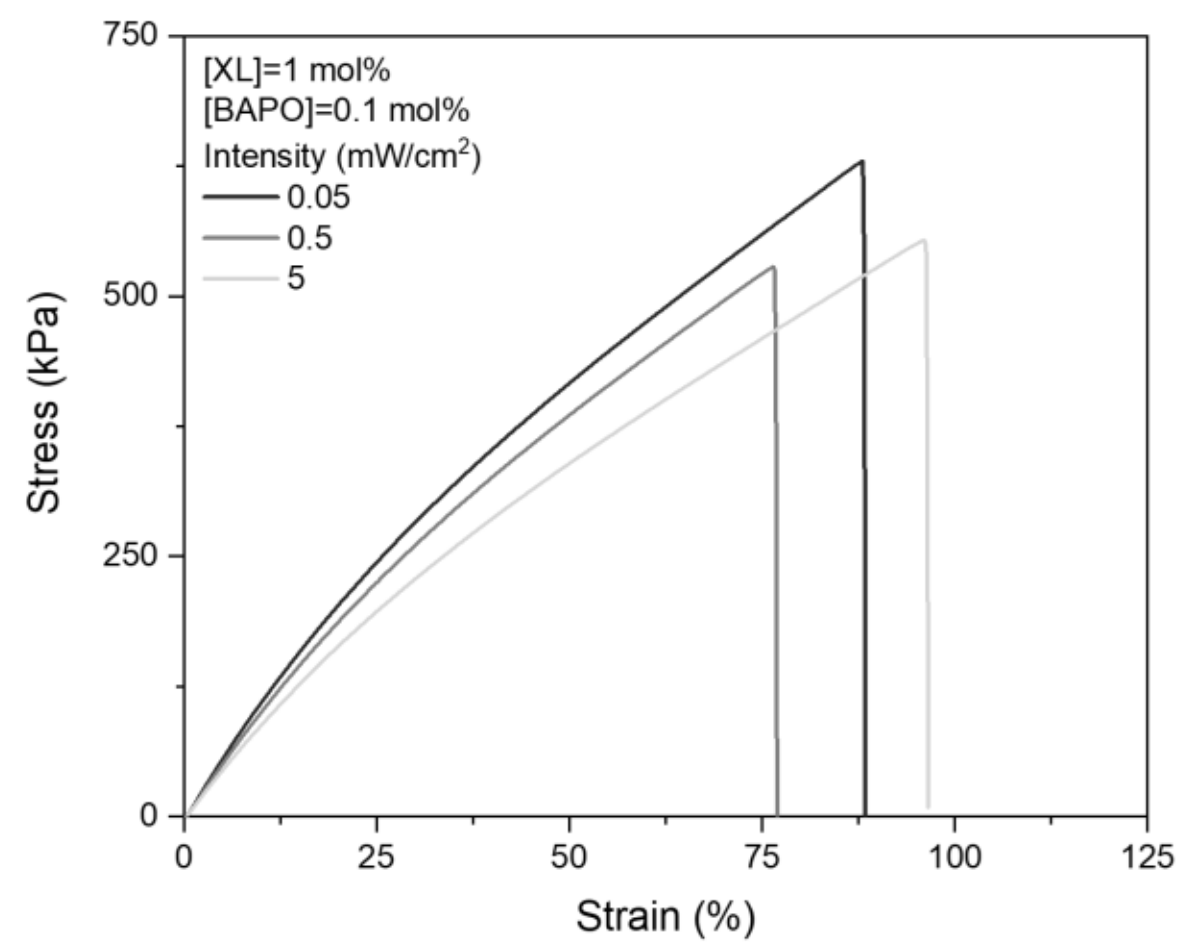

Figure S43. Representative uniaxial stress vs. strain traces for poly(ethyl acrylate) samples prepared using [BAPO] = 0.1 mol%, [XL] = 1 mol% resin, and PPh_3 = 0.1 wt% under 365 nm LED irradiation for 5 minutes, varying light intensity.

Table S28. Tabulated results from **Figure S43**. Mechanical properties of poly(ethyl acrylate) samples prepared using [BAPO] = 0.1 mol%, [XL] = 1 mol% resin, and PPh_3 = 0.1 wt% under 365 nm LED irradiation for 5 minutes. Sol-gel fractions given as gel content. Values are averages ($n \geq 3$) with ±1 standard deviation from the mean provided.

Intensity (mW/cm^2)	Young's Modulus E_T (MPa)	Sol-Gel Fraction	Tensile Strength σ_m (MPa)	Strain at break ε_f (%)	Modulus of Toughness U_t (MJ/m^3)
0.05	1.14 ± 0.03	0.93 ± 0.01	0.6 ± 0.1	82 ± 18	0.3 ± 0.1
0.5	1.12 ± 0.04	0.96 ± 0.02	0.51 ± 0.01	72 ± 3	0.21 ± 0.01
5	0.96 ± 0.02	0.98 ± 0.01	0.56 ± < 0.01	97 ± 2	0.31 ± 0.01

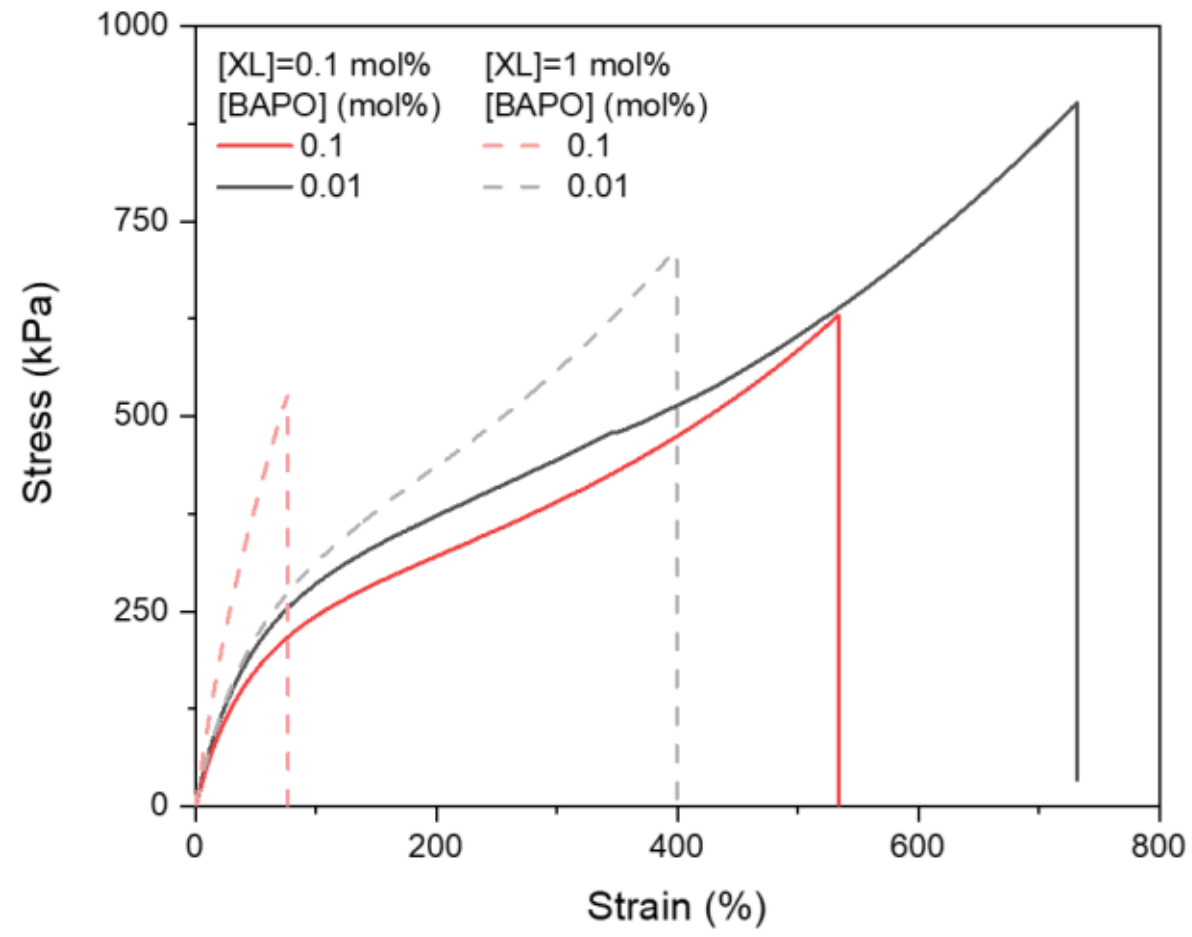

Figure S44. Representative uniaxial tensile stress strain curves of poly(ethyl acrylate) elastomers prepared with varying BAPO and crosslinker concentrations. Formulations contained [BAPO] = 0.1 or 0.01 mol% and [XL] = 1 or 0.1 mol%. PPh_3 (0.1 wt%) was added to minimize oxygen inhibition. Samples containing 0.1 mol% BAPO were irradiated with a 365 nm LED at 0.5 mW/cm^2 for 5 min, whereas samples containing 0.01 mol% BAPO were irradiated with a 365 nm LED at 5 mW/cm^2 for 5 min.

Table S29. Tabulated results from **Figure S44**. Mechanical properties of poly(ethyl acrylate) elastomers prepared with varying BAPO and crosslinker concentrations. Formulations contained [BAPO] = 0.1 or 0.01 mol% and [XL] = 1 or 0.1 mol%. PPh_3 (0.1 wt%) was added to minimize oxygen inhibition. Sol-gel fractions given as gel content. Values are averages (n ≥3) with ±1 standard deviation from the mean provided.

[BAPO] (mol%)	[XL] (mol%)	Intensity (mW/cm^2)	Young's Modulus E_T (MPa)	Sol-Gel Fraction	Tensile Strength σ_m (MPa)	Strain at break ε_f (%)	Modulus of Toughness U_t (MJ/m^3)
0.1	0.1	0.5	0.58 ± 0.02	0.98 ± < 0.01	0.63 ± 0.01	536 ± 17	1.96 ± 0.06
0.1	1	0.5	1.12 ± 0.04	0.96 ± 0.02	0.51 ± 0.01	72 ± 3	0.21 ± 0.01
0.01	0.1	5	0.61 ± 0.03	0.94 ± 0.02	0.8 ± 0.2	743 ± 67	3.5 ± 0.2
0.01	1	5	0.65 ± 0.01	0.97 ± 0.02	0.8 ± 0.1	423 ± 34	1.9 ± 0.1

Table S30. Tabulated results from **Figure 3D**. Mechanical properties of poly(ethyl acrylate) elastomers with varying crosslinker to BAPO ratio, while holding constant [BAPO] = 0.01mol% and PPh_3 = 0.1 wt%. Films were cast under 5 mW/cm^2 365 nm LED exposure for 5 min. Sol-gel fractions given as gel content. Values are averages ($n \geq 3$) with ±1 standard deviation from the mean provided.

[XL]:[BAPO]	Fracture Toughness G_c (kJ/m^2)	Young's Modulus E_T (kPa)	Sol-Gel Fraction	Tensile Strength σ_m (MPa)	Strain at break ε_f (%)	Modulus of Toughness U_t (MJ/m^3)	Fracto-cohesive length L_f=G_c / W_F (mm)
0	1.62 ± 0.03	619 ± 6	0.94 ± 0.01	0.29 ± 0.01	/	/	
1	12.9 ± 0.8	615 ± 27	0.97 ± 0.02	0.88 ± 0.02	2846 ± 151	12.23 ± 0.02	1.06 ± 0.06
2	10.5 ± 0.4	606 ± 12	0.96 ± 0.01	2.7 ± 0.2	2863 ± 100	23.8 ± 0.2	0.44 ± 0.02
5	2.35 ± 0.02	615 ± 10	0.96 ± 0.02	1.2 ± 0.2	1128 ± 105	6.3 ± 0.2	0.37 ± 0.01
10	1.2 ± 0.1	612 ± 28	0.94 ± 0.02	0.8 ± 0.2	743 ± 67	3.5 ± 0.2	0.34 ± 0.04
20	1.2 ± 0.3	643 ± 22	0.95 ± 0.02	0.86 ± 0.08	497 ± 35	2.41 ± 0.07	0.50 ± 0.01
100	1.34 ± 0.09	650 ± 14	0.97 ± 0.02	0.8 ± 0.1	423 ± 34	1.9 ± 0.1	0.70 ± 0.06

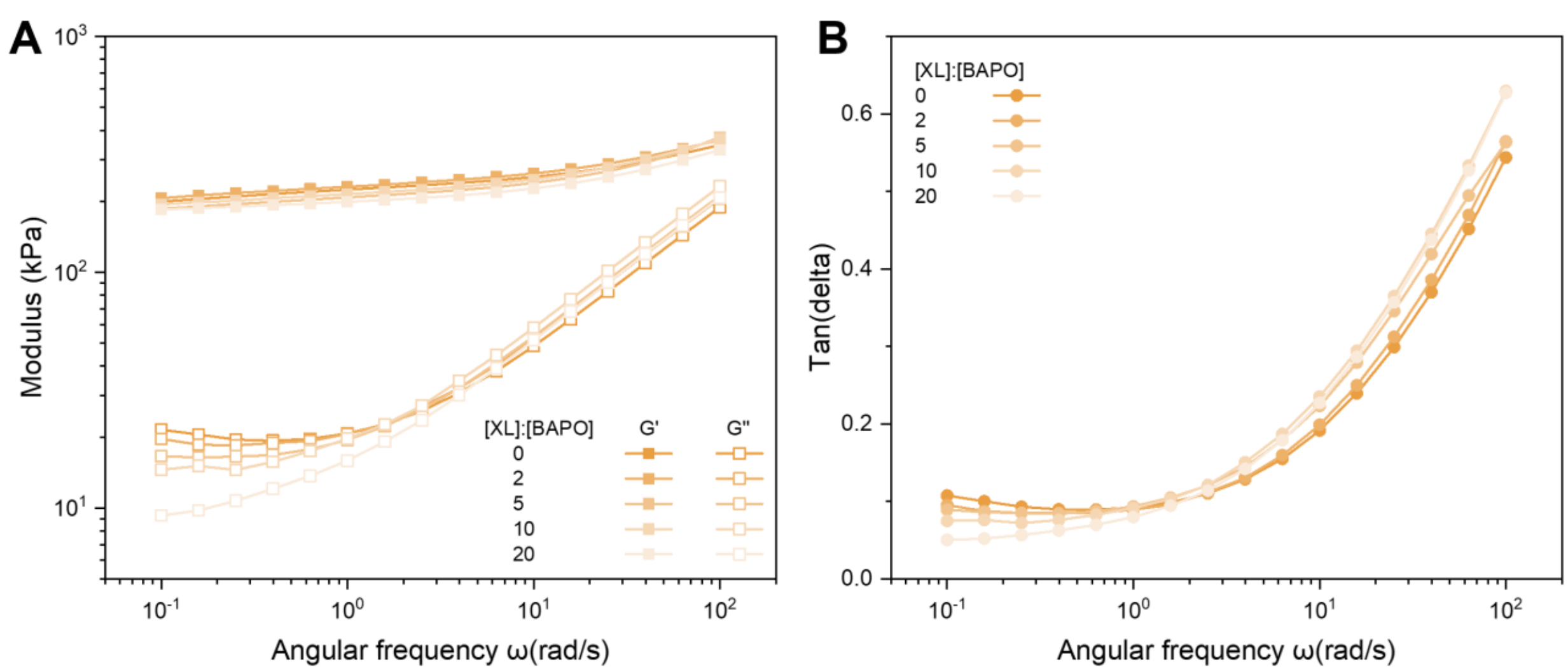

Figure S45. Rheology of entangled poly(ethyl acrylate) elastomers synthesized using [BAPO] = 0.01 mol% and PPh_3 = 0.1 wt% under 5 mW/cm^2 365 nm LED exposure for 5 min, varying [BAPO]:[XL]. (A) Storage modulus G' and loss modulus G'' obtained using a frequency sweep from 0.1 rad/s to 100 rad/s. (B) Corresponding tan(delta) results from the frequency sweep.

Step-cyclic tensile testing of highly entangled poly(ethyl acrylate) elastomers

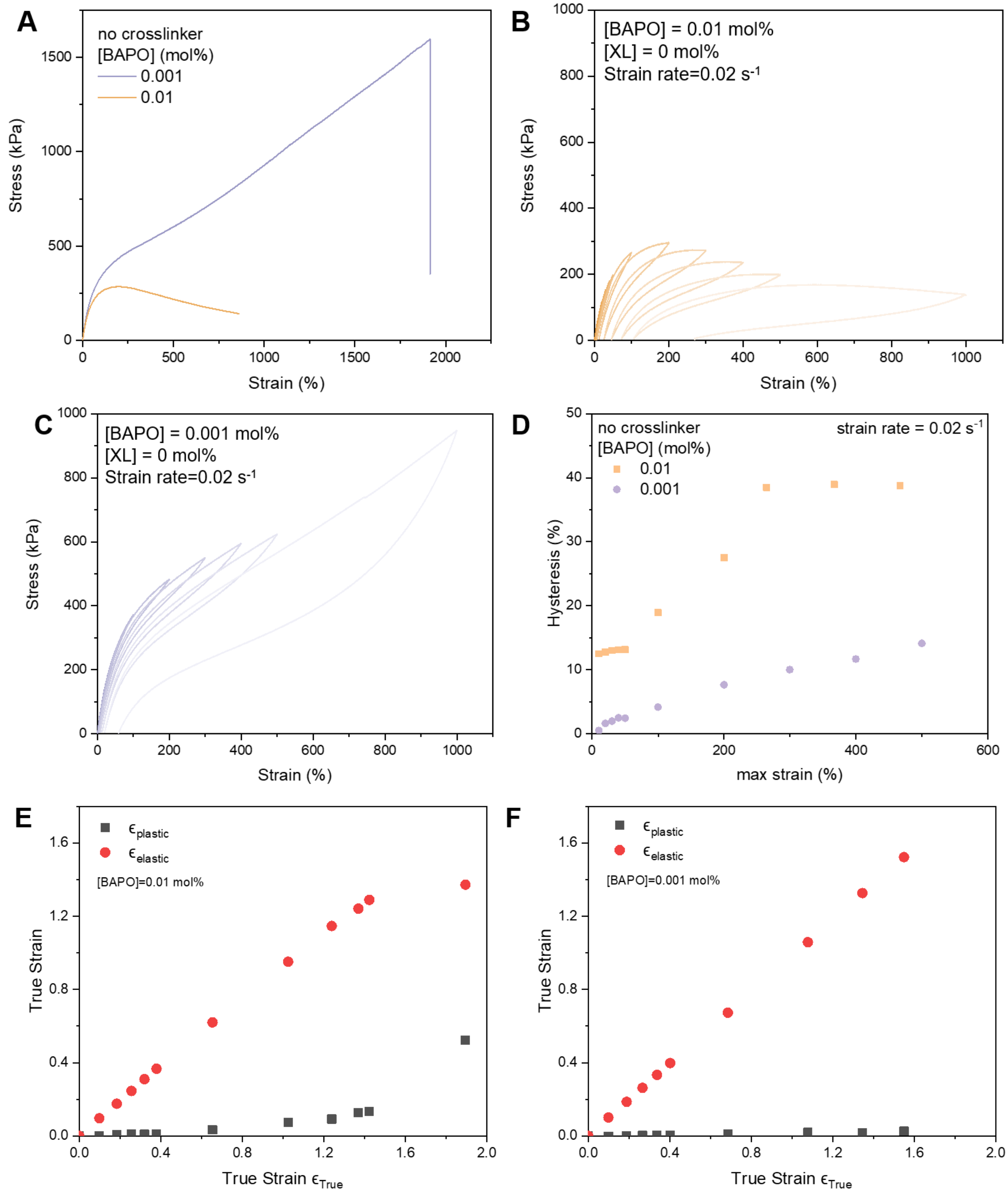

Figure S46. Mechanical analysis of highly entangled poly(ethyl acrylate) elastomers prepared without crosslinker, while using [BAPO] = 0.01 or 0.001 mol% and PPh_3 = 0.1 wt%. Strain rate = 0.02 s^{-1}.(A) Uniaxial tensile stress-strain curves deformed to fracture. (B) Step-cyclic tensile testing (stepwise cyclic tensile testing) of samples cast using [BAPO] = 0.01 mol%, 5 mW/cm^2 365 nm LED irradiation for 5 min. (C) Step-cyclic tensile testing (stepwise cyclic tensile testing) of samples cast using [BAPO] = 0.001 mol%, 1 mW/cm^2 365 nm LED irradiation for 20 min. (D) Corresponding hysteresis loss from stepwise cyclic tensile testing. (E) Corresponding true strain, showing elastic and plastic behavior from stepwise cyclic tensile testing.

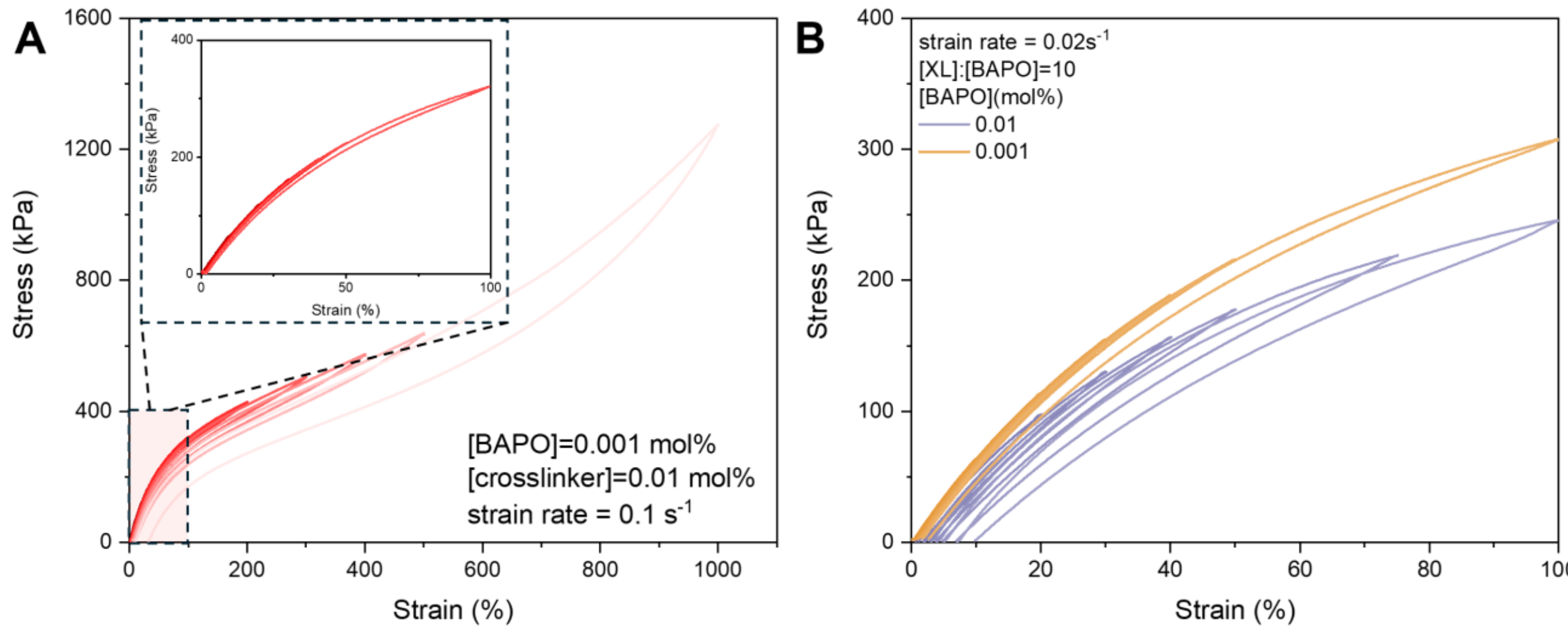

Figure S47. Stepwise cyclic tensile testing of highly entangled poly(ethyl acrylate) elastomers prepared with [XL]:[BAPO] = 10, PPh_3 = 0.1 wt%, and variable [BAPO] and strain rate. (A) Samples cast with [BAPO] = 0.001 mol%, [XL] = 0.01 mol%, 1 mW/cm^2 365 nm LED irradiation for 20 min. Strain rate = 0.1 s^{-1} (B) Samples cast with [BAPO] = 0.01 mol%, [XL] = 0.1 mol%, 5 mW/cm^2 365 nm LED irradiation for 5 min (purple line) or [BAPO] = 0.001 mol%, [XL] = 0.01 mol%, 1 mW/cm^2 365 nm LED irradiation for 20 min (yellow line). Strain rate = 0.02 s^{-1}.

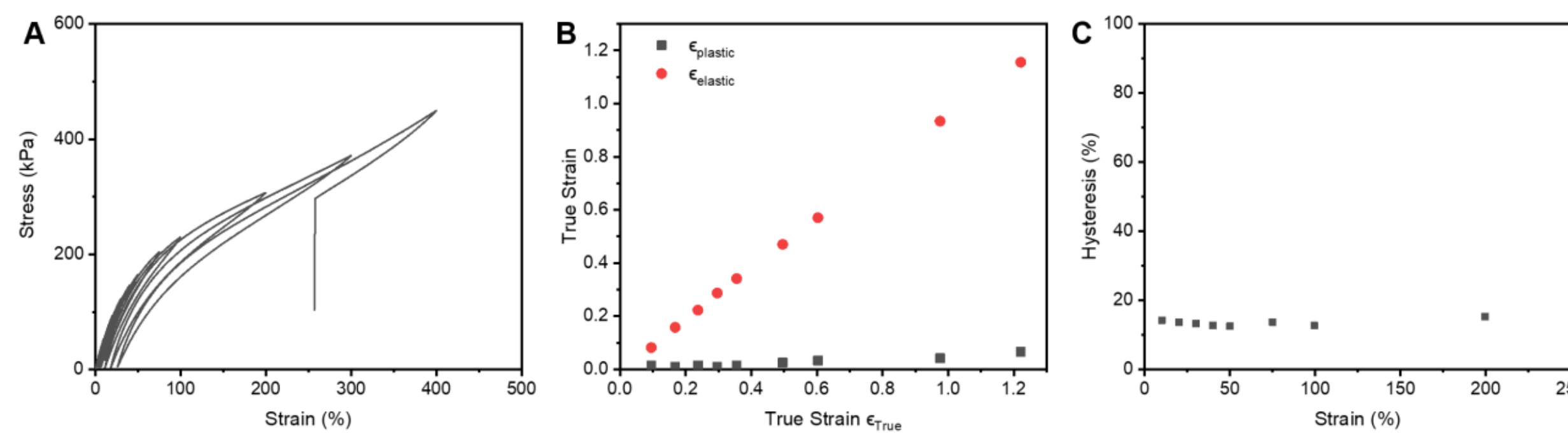

Figure S48. Stepwise cyclic tensile testing of highly entangled poly(ethyl acrylate) elastomers prepared using [BAPO] = 0.1 mol%, [XL] = 0.1 mol%, and [PPh_3] = 0.1 wt% under 0.5 mW/cm^2 365 nm LED irradiation for 5 min. Strain rate = 0.02 s^{-1}. (A) Cyclic uniaxial deformation with stress-strain plot for stepwise cyclic tensile testing. (B) Corresponding true strain, showing elastic and plastic behavior from stepwise cyclic tensile testing. (C) Corresponding hysteresis loss from stepwise cyclic tensile testing.

S2.9 Mechanical properties of hydrogels prepared via photoiniferter vs free-radical

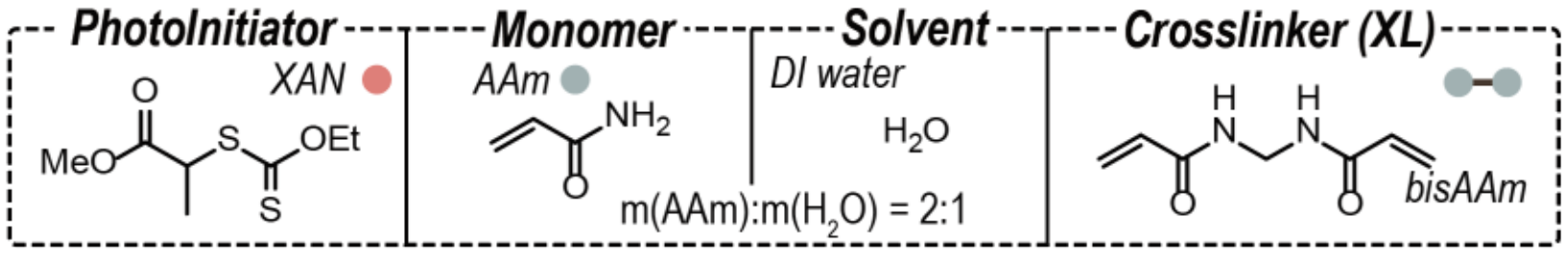

Figure S49. Chemical structures for components implemented in the preparation of entangled poly(acrylamide) hydrogels via photoiniferter with XAN.

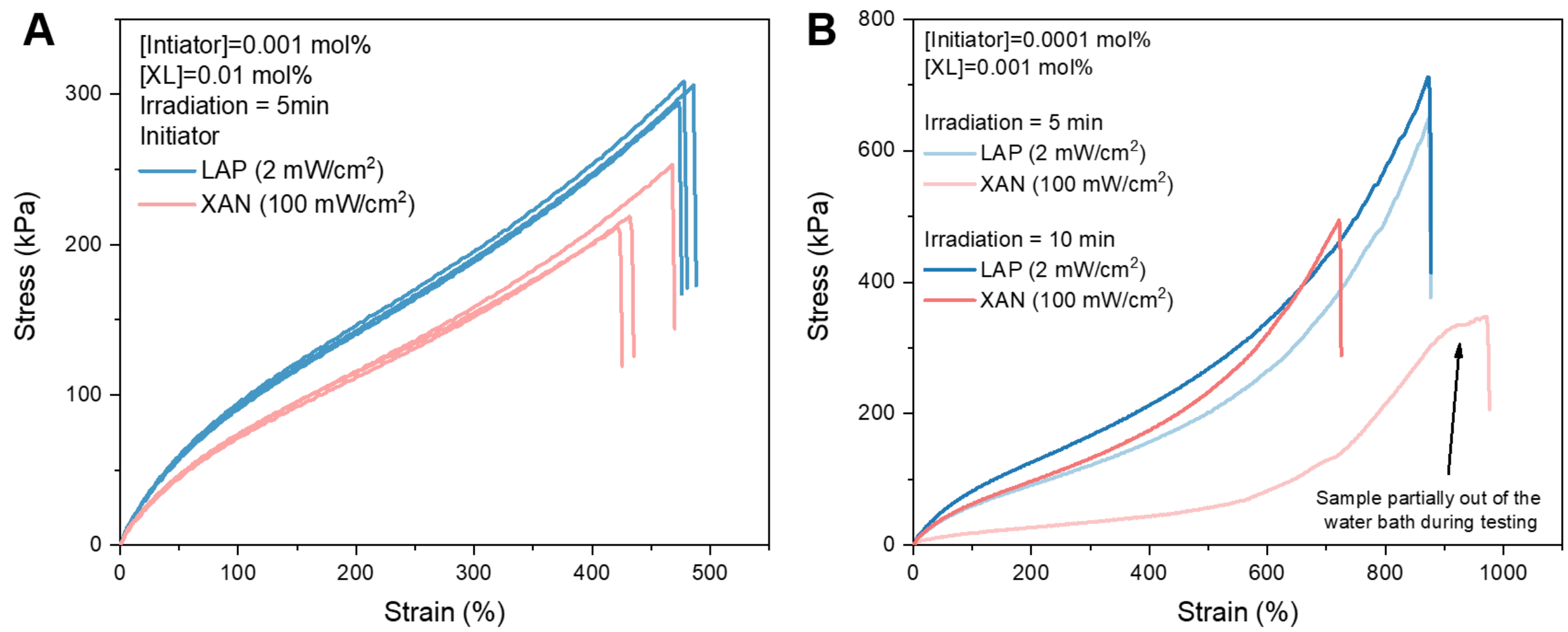

Figure S50. Stress-strain curves from uniaxial tensile testing of highly entangled hydrogels prepared with different initiators and initiator concentrations. Strain rate = 1 s^{-1}. Casting conditions: LAP using 2 mW/cm^2 365 nm LED exposure; XAN using 100 mW/cm^2 365 nm LED exposure. (A) [Initiator]/[AAm] = 0.001 mol%, [XL]/[AAm] = 0.01 mol%. Irradiation for 5 min. (B) [Initiator]/[AAm] = 0.0001 mol%, [XL]/[AAm] = 0.001 mol%. Irradiation for 5 or 10 min as noted.

Table S31. Summary of the **Figure S50**. Highly entangled hydrogels synthesized with LAP or XAN under different initiator concentration and curing conditions. Values are averages (n ≥3) with ±1 standard deviation from the mean provided.

Initiator	[Initiator]/ [AAm] (mol%)	Intensity (mW/cm^2)	Curing Time (min)	Water Content (wt%)	Swollen ratio	Young's Modulus E_T (kPa)
LAP	0.001	2	5	83.1 ± < 0.1	4.1 ± < 0.1	122 ± 3
XAN	0.001	100	5	84 ± 2	4.4 < 0.1	94 ± 4
LAP	0.0001	2	5	86.0 ± 0.2	4.2 ± 0.1	80 ± 3
LAP	0.0001	2	10	83.8 ± 0.1	4.2 < 0.1	111 ± 6
XAN	0.0001	100	5	94 ± 1	8 ± 1	21 ± 1
XAN	0.0001	100	10	85.7 ± 0.3	4.7 ± 0.1	79 ± 2

S2.10 Kinetics of acrylamide polymerization

S2.10.1 Acrylamide free radical polymerizations with LAP

Table S32. Summary of **Figure 4B**. Kinetics of acrylamide monomer homopolymerizations using LAP or XAN at an [initiator]/[AAm] = 0.001 mol%, crosslinker [bisacrylamide]/[AAm] = 0.01 mol% and irradiation with a 365 nm LED at variable intensity for 230 s. Values are averages (*n* ≥3) with ±1 standard deviation from the mean provided.

Initiator	Intensity (mW/cm^2)	Initial Rate ($\%s^{-1}$)	Initial Rate (mM/s)	ρ_{max} in run time (%)	Inhibition time t_{inhib} (s)
	5	0.44 ± 0.05	41 ± 4	87 ± 3	68 ± 4
	10	0.52 ± 0.03	49 ± 3	90 ± 2	35 ± 2
LAP	25	0.56 ± 0.03	53 ± 3	52 ± 3	23 ± 3
	50	0.52 ± 0.04	48 ± 3	40 ± 3	16 ± 1
	150	0.13 ± 0.03	12 ± 3	34 ± 8	12 ± 3

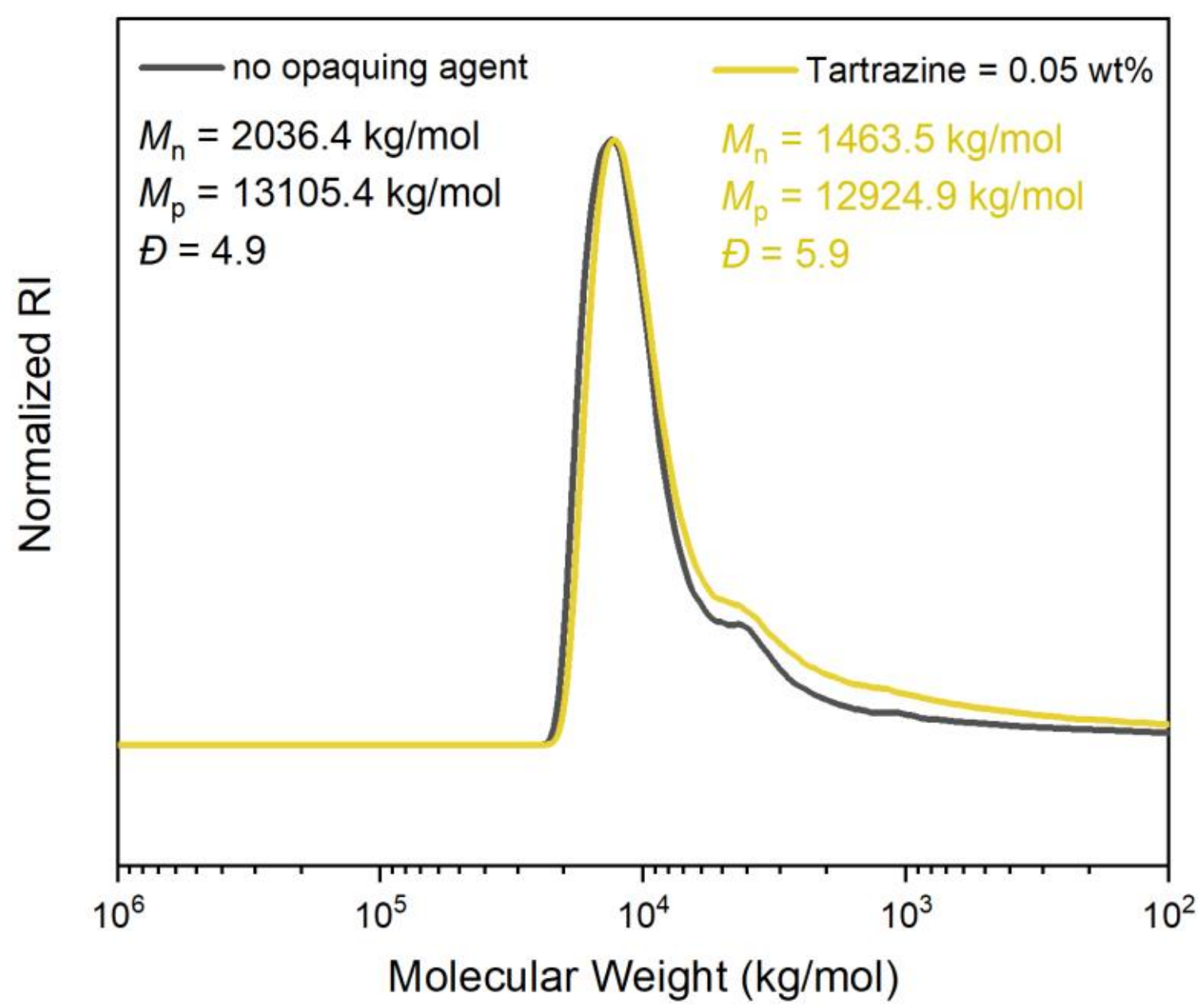

Figure S51. Aqueous SEC traces of polyacrylamide, relative to PEG/PEO standards, prepared using [LAP]/[AAm] = 0.001 mol% under 5 mW/cm^2 365 nm LED irradiation for 300 s, with and without opaquing agent.

S2.10.2 Acrylamide photoiniferter polymerization with XAN

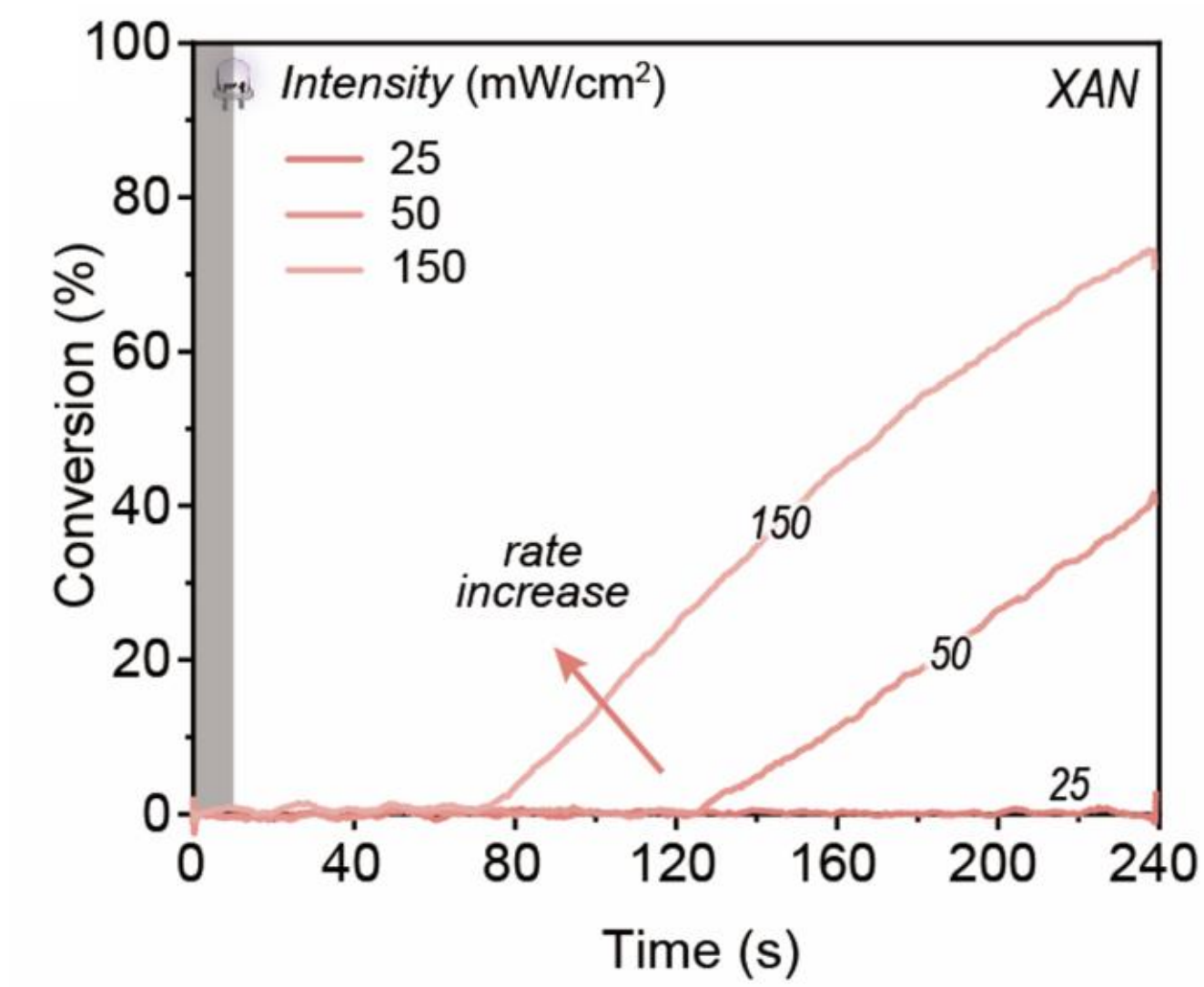

Figure S52. Conversion vs time traces from real time FTIR spectroscopy, showing photopolymerization kinetics of acrylamide monomer via photoiniferter. [XAN] = 0.001 mol%, [XL] = 0.01 wt%. Samples were 100 µm thick and irradiated with a 365 nm LED at variable intensity. LEDs were turned on after 10 s.

Table S33. Summary of **Figure S52**. Kinetics of acrylamide homopolymerizations using LAP or XAN at [initiator]/[AAm] = 0.001 mol%, crosslinker [bisacrylamide]/[AAm] = 0.01 mol% under 365 nm LED exposure with varying intensity for 230 s. Values are averages ($n \geq 3$) with ±1 standard deviation from the mean provided.

Initiator	Intensity (mW/cm^2)	Initial Rate (%s^{-1})	Initial Rate (mM/s)	ρ_{max} in run time (%)	Inhibition time t_{inhib} (s)
XAN	25	No conversion observed in 230 s			
	50	0.27 ± 0.01	25 ± 1	41.1 ± 0.2	121 ± 6
	150	0.45 ± 0.02	43 ± 2	75 ± 2	65 ± 1

S2.10.3 Photo-Rheology of highly entangled hydrogel formation

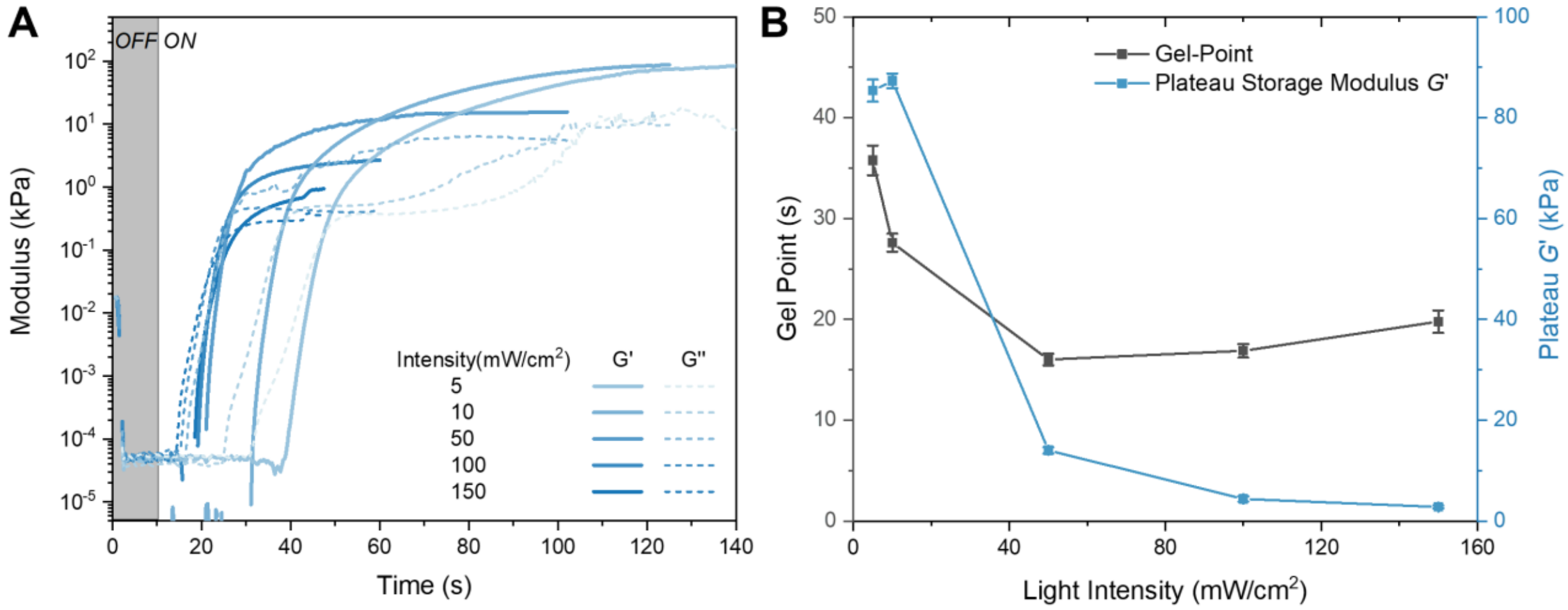

Figure S53. Photo-rheology of highly entangled poly(acrylamide) hydrogel formation using resins containing [LAP] = 0.01 mol%, [XL] = 0.1 mol% exposed to 365 nm LEDs at varying light intensity. (A) Storage modulus *G*' and loss modulus *G*'' obtained via fast sampling to estimate a gel point as the *G*' and *G*'' crossover. (B) Corresponding gel-point and plateau *G*'.

S2.11 Mechanical properties of highly entangled hydrogels

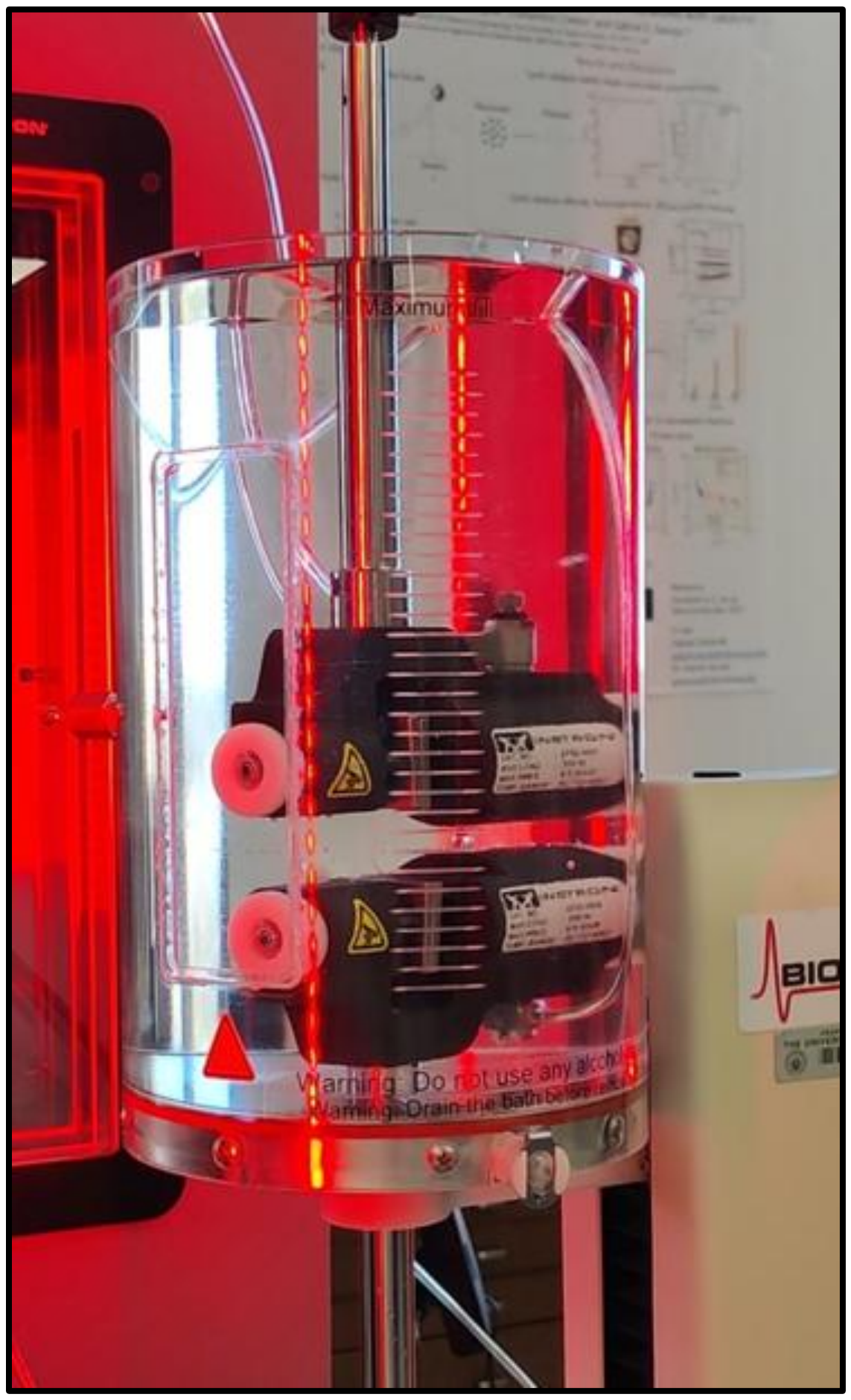

Figure S54. Image of the uniaxial tensile testing setup used to deform hydrogel samples fully submerged in water and avoid dehydration during measurement.

Table S34. Summary of **Figure 4D(ii)** tensile testing results. Samples prepared using [LAP]:[XL] = 10 and variable casting conditions: For [LAP]/[AAm] = 0.01 and 0.001 mol% samples, 5 mW/cm^2 365 nm LED irradiation for 5 min was used, while samples with [LAP]/[AAm] = 0.0001 mol% were irradiated with 1 mW/cm^2 365 nm LED for 20 min to provide more comparable monomer conversions. Values are averages ($n \geq 3$) with ±1 standard deviation from the mean provided.

[LAP]/ [AAm] (mol%)	Water Content (wt%)	Swollen ratio	Fracture Toughness G_c (kJ/m^2)	Young's Modulus E_T (kPa)	Strain at break ε_f (%)	Tensile Strength σ_m (kPa)	Modulus of Toughness U_t (MJ/m^3)	Fracto-cohesive length L_f=G_c / W_F (mm)
0.01	80.6 ± 0.2	3.4 ± < 0.1	0.03 ± 0.01	143 ± 7	175 ± 3	141 ± 5	0.1 ± < 0.1	0.31 ± 0.05
0.001	81.5 ± 0.2	3.6 ± < 0.1	0.68 ± 0.03	122 ± 4	515 ± 19	370 ± 42	0.9 ± 0.1	0.75 ± 0.09
0.0001	86.2 ± 0.3	4.0 ± < 0.1	3.1 ± 0.1	66 ± 3	876 ± 16	628 ± 68	1.8 ± 0.2	1.7 ± 0.2

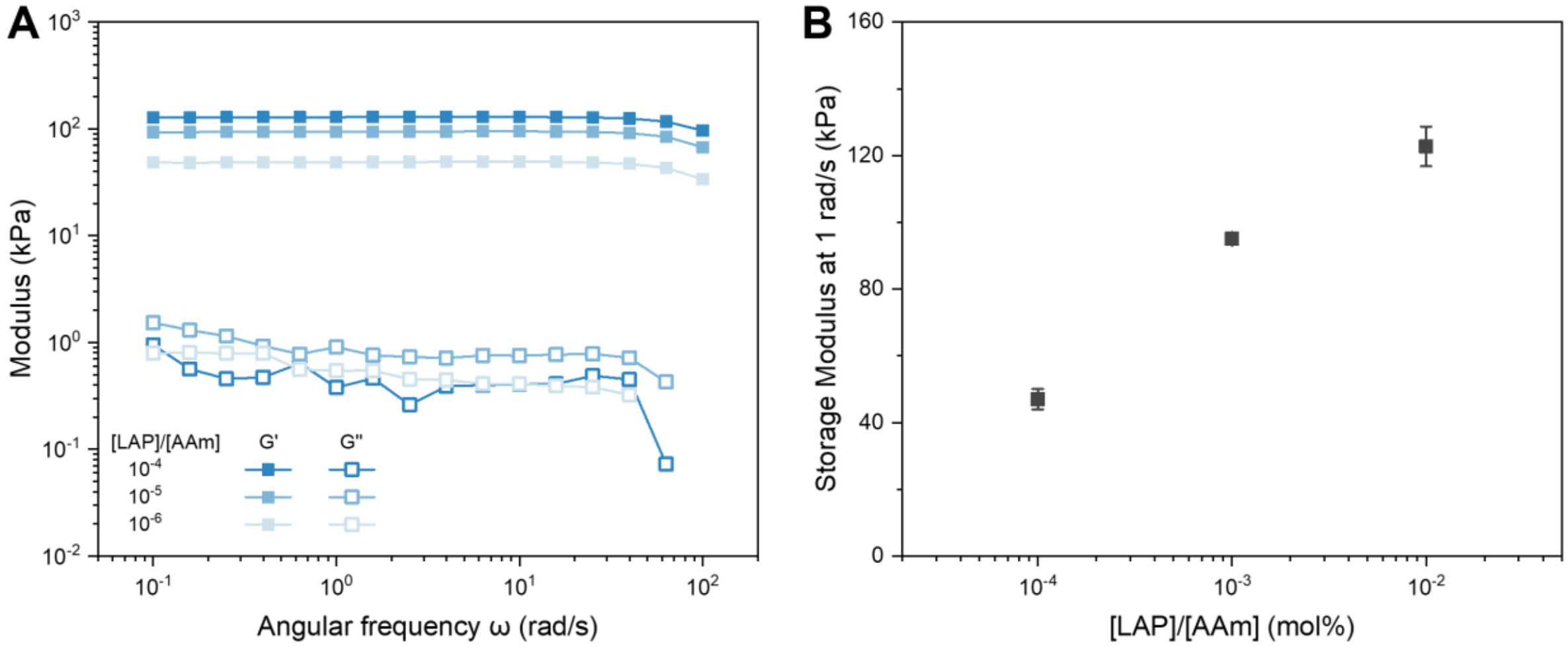

Figure S55. Rheology of fully swollen highly entangled hydrogels that were synthesized with varying LAP concentration as noted. (A) Representative storage modulus *G*' and loss modulus *G*'' obtained via frequency sweep from 0.1 rad/s to 100 rad/s. (B) Corresponding *G*' values at 1 rad/s vs. [LAP]/[AAm] concentration. Values are averages ($n \geq 3$) with ±1 standard deviation from the mean provided.

S2.12 Influence of opaquing agent on photopolymerization kinetics

S2.12.1 Ethyl acrylate resins

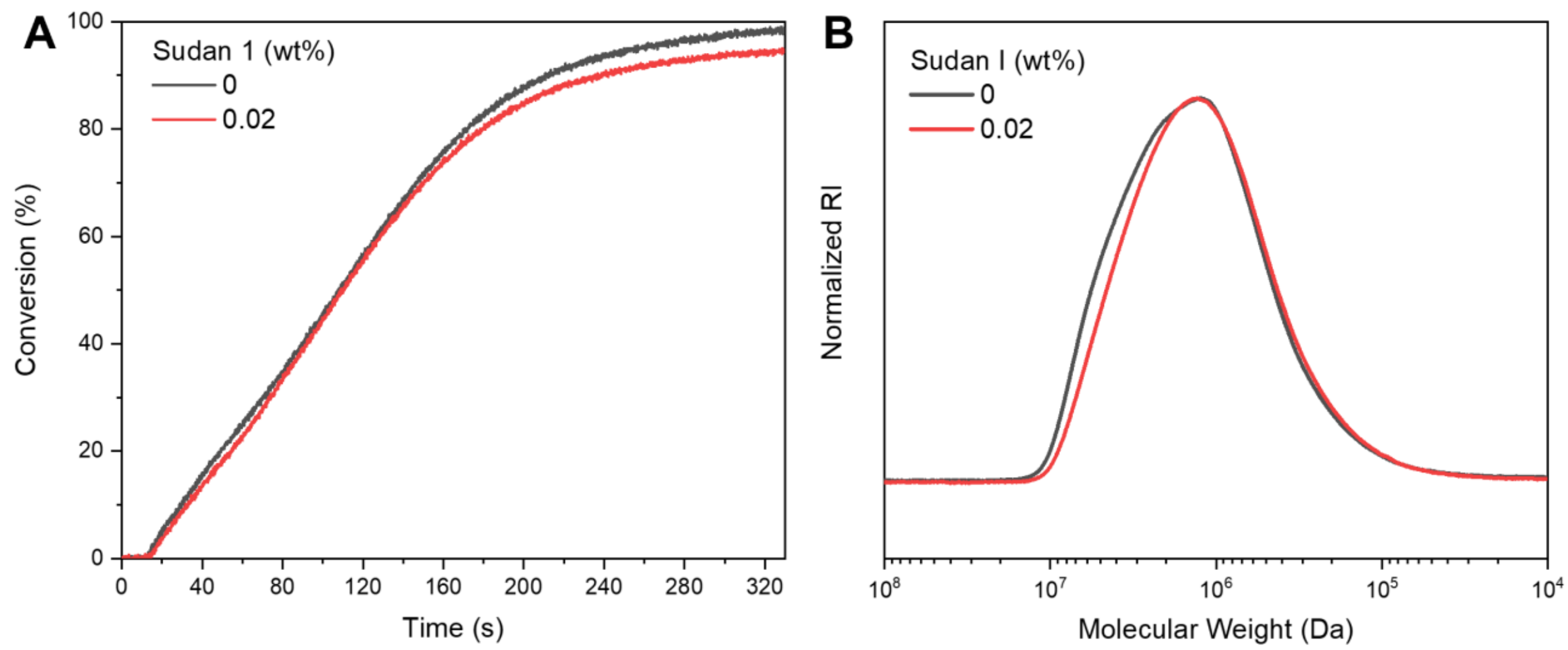

Figure S56. Ethyl acrylate homopolymerizations with [BAPO] = 0.01 mol% and 0.1 wt% PPh_3 under 5 mW/cm^2 365 nm LED irradiation (320 s) with or without opaquing agent (0.02 wt% Sudan 1). (A) Representative conversion vs time plot from real time FTIR spectroscopy monitoring. LED turned on 10 seconds after beginning the measurement. (B) Corresponding GPC traces of the polymers at the end of the irradiation (320 s).

Table S35. Summary of **Figure S56A**. Kinetics of ethyl acrylate homopolymerization using [BAPO] = 0.01 mol% and 0.1 wt% PPh_3 under 5 min 5 mW/cm^2 365 nm LED irradiation. Samples were 100 µm thick, with or without opaquing agent (0.02 wt% Sudan 1). Values are averages (n ≥3) with ±1 standard deviation from the mean provided.

[BAPO] (mol%)	Sudan 1 (wt%)	Initial Rate (%s^{-1})	Initial Rate (mM/s)	ρ_{max} in run time (%)	Inhibition time (s)
0.01	0	0.58 ± 0.01	54 ± 1	98.53 ± 0.07	3.3 ± 0.2
0.01	0.02	0.50 ± 0.01	46.9 ± 0.6	93.1 ± 0.2	5.1 ± 0.3

Table S36. Summary of **Figure S56B.** GPC results for poly(ethyl acrylate) samples (100 µm thick) prepared using [BAPO] = 0.01 mol% and 0.1 wt% PPh_3 under 5 min 5 mW/cm^2 365 nm LED irradiation**.**

[BAPO] (mol%)	Sudan 1 (wt%)	$M_{n,GPC}$ (kDa)	M_w/M_n	Conv.[a] (%)
0.01	0	678.7	2.78	99
0.01	0.02	621.7	2.64	95

[a.] Monomer conversion is obtained via real-time FTIR

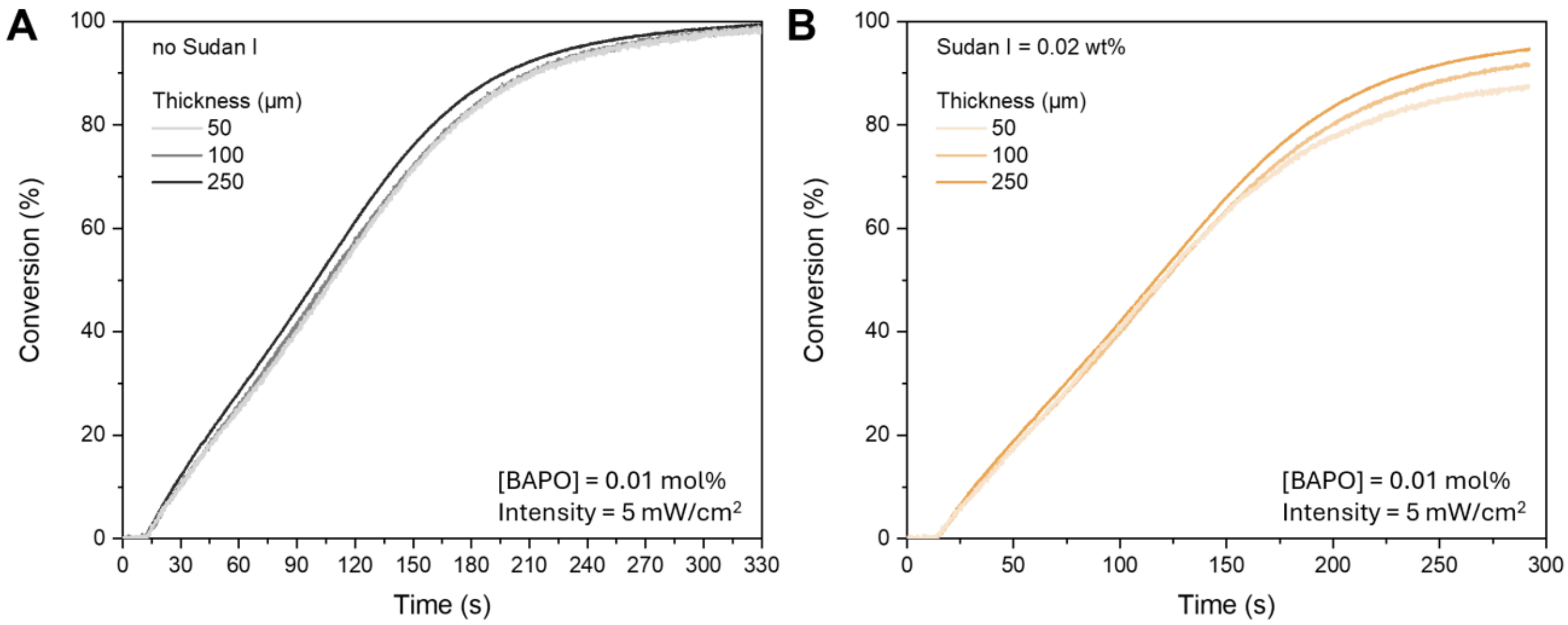

Figure S57. Ethyl acrylate homopolymerizations with [BAPO] = 0.01 mol% and 0.1 wt% PPh_3 under 5 mW/cm^2 365 nm LED irradiation (330 s or 290 s) with or without opaquing agent at varying sample thickness: 50, 100, and 250 µm. Representative conversion vs time plot from real time FTIR spectroscopy monitoring. LED turned on 10 seconds after beginning the measurement. (A) Without opaquing agent. (B) With opaquing agent, [Sudan I] = 0.02 wt%.

Table S37. Summary of **Figure S57**. Kinetics of ethyl acrylate homopolymerization using [BAPO] = 0.01 mol% and 0.1 wt% PPh_3 under 5 mW/cm^2 365 nm LED irradiation (330 s or 290 s) at varying sample thickness with or without opaquing agent (0.02 wt% Sudan 1). Values are averages (n ≥3) with ±1 standard deviation from the mean provided.

[BAPO] (mol%)	Sudan 1 (wt%)	Sample Thickness (µm)	Initial Rate (%s^{-1})	Initial Rate (mM/s)	ρ_{max} in run time (%)	Inhibition time (s)
0.01	0	50	0.59 ± 0.02	56 ± 2	97 ± 2	3.64 ± 0.06
0.01	0	100	0.58 ± 0.01	54 ± 1	98.53 ± 0.07	3.3 ± 0.2
0.01	0	250	0.69 ± 0.01	64.4 ± 0.9	99.6 ± 0.3	2.8 ± 0.1
0.01	0.02	50	0.49 ± 0.02	46 ± 2	86.5 ± 0.9	4.9 ± 0.2
0.01	0.02	100	0.50 ± 0.01	46.9 ± 0.6	91.4 ± 0.3	5.1 ± 0.3
0.01	0.02	250	0.567 ± 0.008	53.2 ± 0.7	94.7 ± 0.1	5.7 ± 0.2

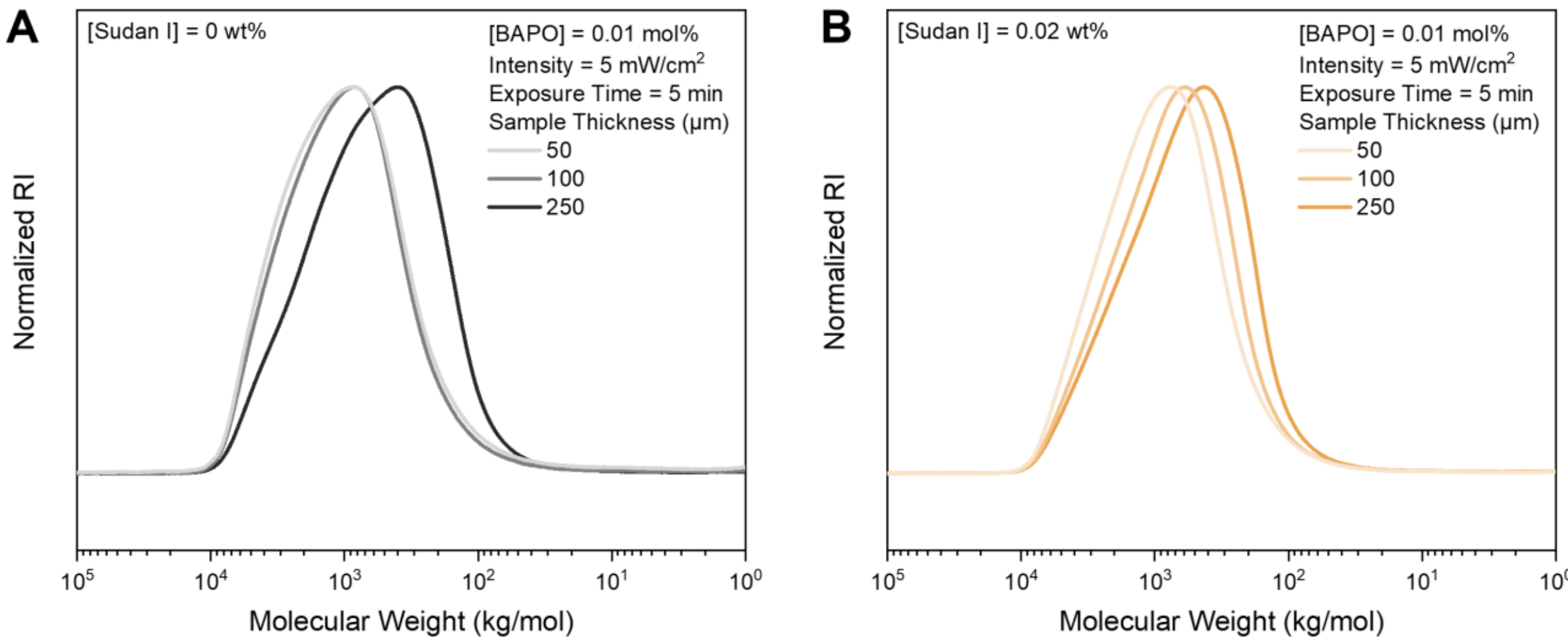

Figure S58. Ethyl acrylate homopolymerizations with [BAPO] = 0.01 mol% and 0.1 wt% PPh_3 under 5 mW/cm^2 365 nm LED irradiation (300 s) with or without opaquing agent at varying sample thickness: 50, 100, and 250 µm. (A) Without opaquing agent. (B) With opaquing agent, [Sudan I] = 0.02 wt%.

Table S38. Summary of **Figure S58.** GPC results for poly(ethyl acrylate) samples prepared using [BAPO] = 0.01 mol% and 0.1 wt% PPh_3 under 5 mW/cm^2 365 nm LED irradiation (300 s) with or without opaquing agent at varying sample thickness**.**

[BAPO] (mol%)	Sudan 1 (wt%)	Sample Thickness (µm)	$M_{n,GPC}$ (kDa)	M_w/M_n	Conv.[a] (%)
0.01	0	50	514.9	2.81	97
0.01	0	100	526.6	2.69	99
0.01	0	250	320.4	2.93	99
0.01	0.02	50	474.8	2.62	87
0.01	0.02	100	416.9	2.52	92
0.01	0.02	250	336.2	2.65	95

[a.] Monomer conversion is obtained via real-time FTIR

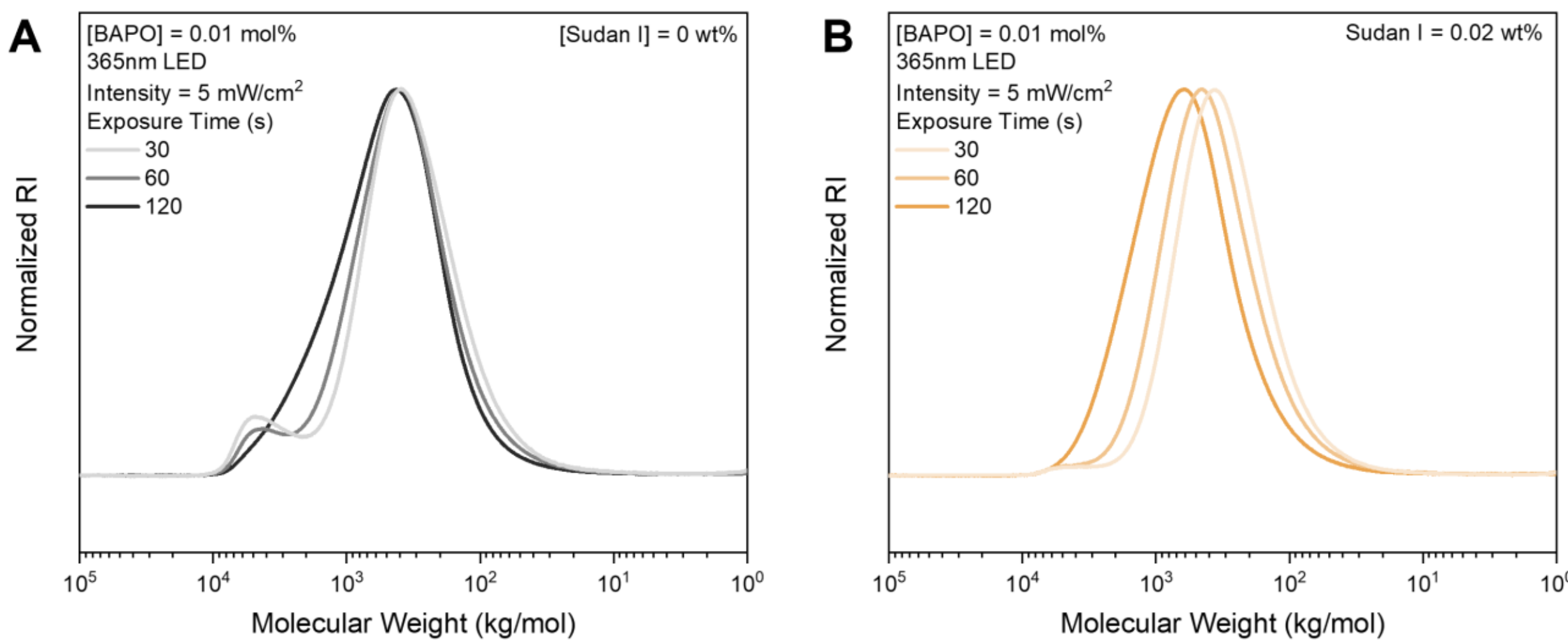

Figure S59. Ethyl acrylate homopolymerizations (100 µm thick) with [BAPO] = 0.01 mol% and 0.1 wt% PPh_3 under 5 mW/cm^2 365 nm LED irradiation with or without opaquing agent at varying irradiation time: 30, 60, and 120s. (A) Without opaquing agent. (B) With opaquing agent, [Sudan I] = 0.02 wt%.

Table S39. Summary of **Figure S59.** GPC results for poly(ethyl acrylate) samples (100 µm) prepared using [BAPO] = 0.01 mol% and 0.1 wt% PPh_3 under 5 mW/cm^2 365 nm LED irradiation with or without opaquing agent at varying irradiation time**.**

[BAPO] (mol%)	Sudan 1 (wt%)	Irradiation Time (s)	$M_{n,GPC}$ (kDa)	M_w/M_n	Conv.[a] (%)
0.01	0	30	215.8	3.00	15
0.01	0	60	235.5	2.74	30
0.01	0	120	287.2	2.54	62
0.01	0.02	30	210.5	2.02	14
0.01	0.02	60	253.4	2.11	28
0.01	0.02	120	371.9	2.20	61

a. Monomer conversion is obtained via real-time FTIR

S2.12.2 Acrylamide resins

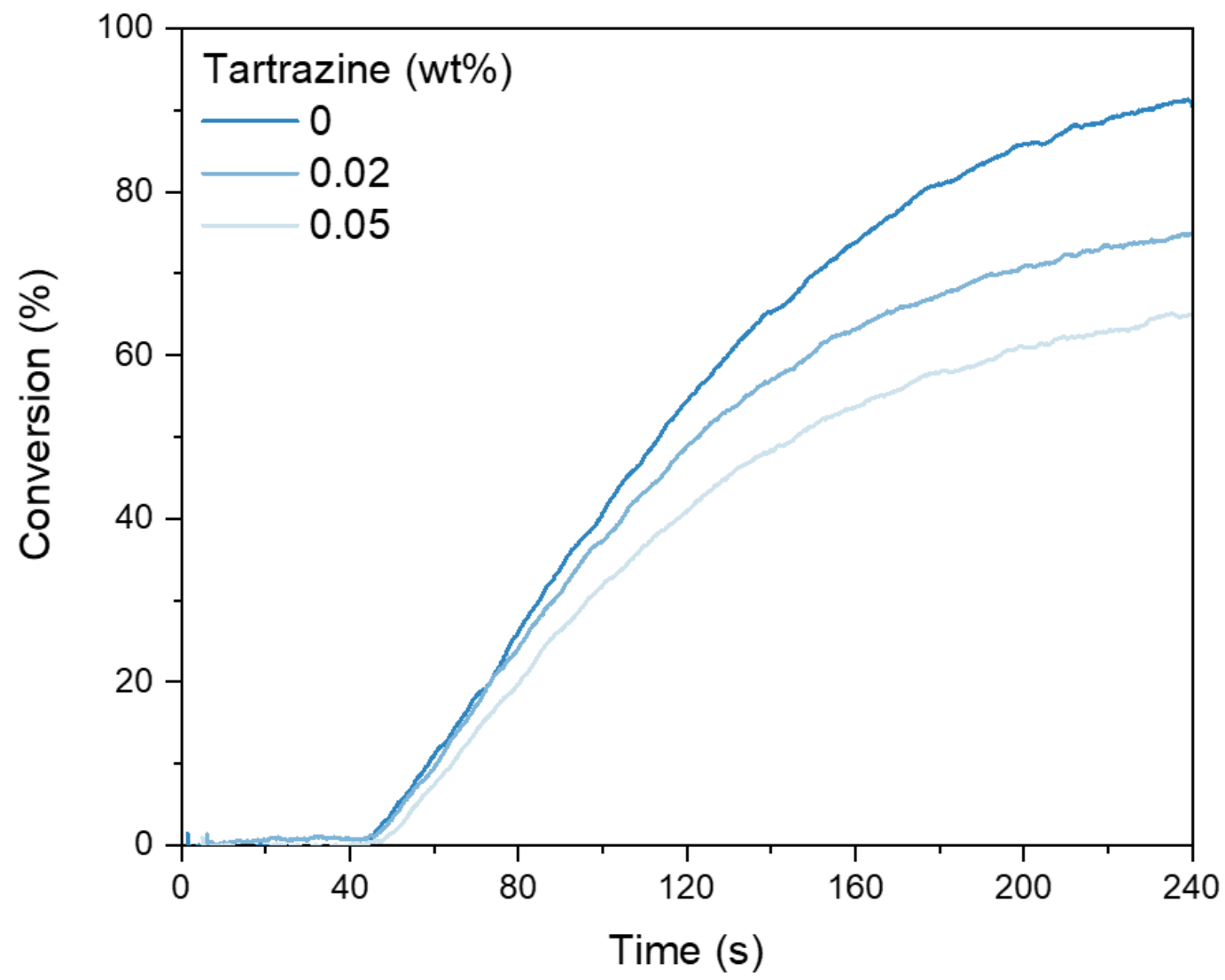

Figure S60. Representative conversion traces of acrylamide resins (100 µm thick) as a function of irradiation time monitored using real time FTIR spectroscopy (LED turned on after 10 seconds of measurement). Samples contained [LAP]/[AAm] = 0.001 mol%, crosslinker [bisacrylamide]/[AAm] = 0.01 mol%, with or without opaquing agent (Tartrazine) and were irradiated with 10 mW/cm^2 365 nm LED for 230 s.

Table S40. Tabulated results from **Figure S60**. Kinetics of acrylamide photopolymerizations (100 µm thick samples) using [LAP]/[AAm] = 0.001 mol%, crosslinker [bisacrylamide]/[AAm] = 0.01 mol%, with or without opaquing agent (Tartrazine) and were irradiated with 10 mW/cm^2 365 nm LED for 230 s.

[LAP]/ [AAm] (mol%)	Tartrazine (wt%)	Initial Rate (%s^{-1})	Initial Rate (mM/s)	ρ_{max} in run time (%)	Inhibition time (s)
0.001	0	0.52 ± 0.03	49 ± 3	90 ± 2	35 ± 2
0.001	0.02	0.45 ± 0.04	43 ± 4	71 ± 3	35 ± 2
0.001	0.05	0.42 ± 0.02	39 ± 1	63 ± 3	38.8 ± 0.8

S2.13 Photo-rheology of resins for 3D Printing

The gel point of the printing resin was characterized using photo-rheology. A 365 nm LED was used at an intensity of 5 mW/cm^2 to match the printing conditions, with the LED turned on 10 s after the start of the measurement. For elastomer resins, the environmental temperature was maintained at 25 °C; for hydrogel resins, it was maintained at 35 °C.

S2.13.1 Elastomer resin

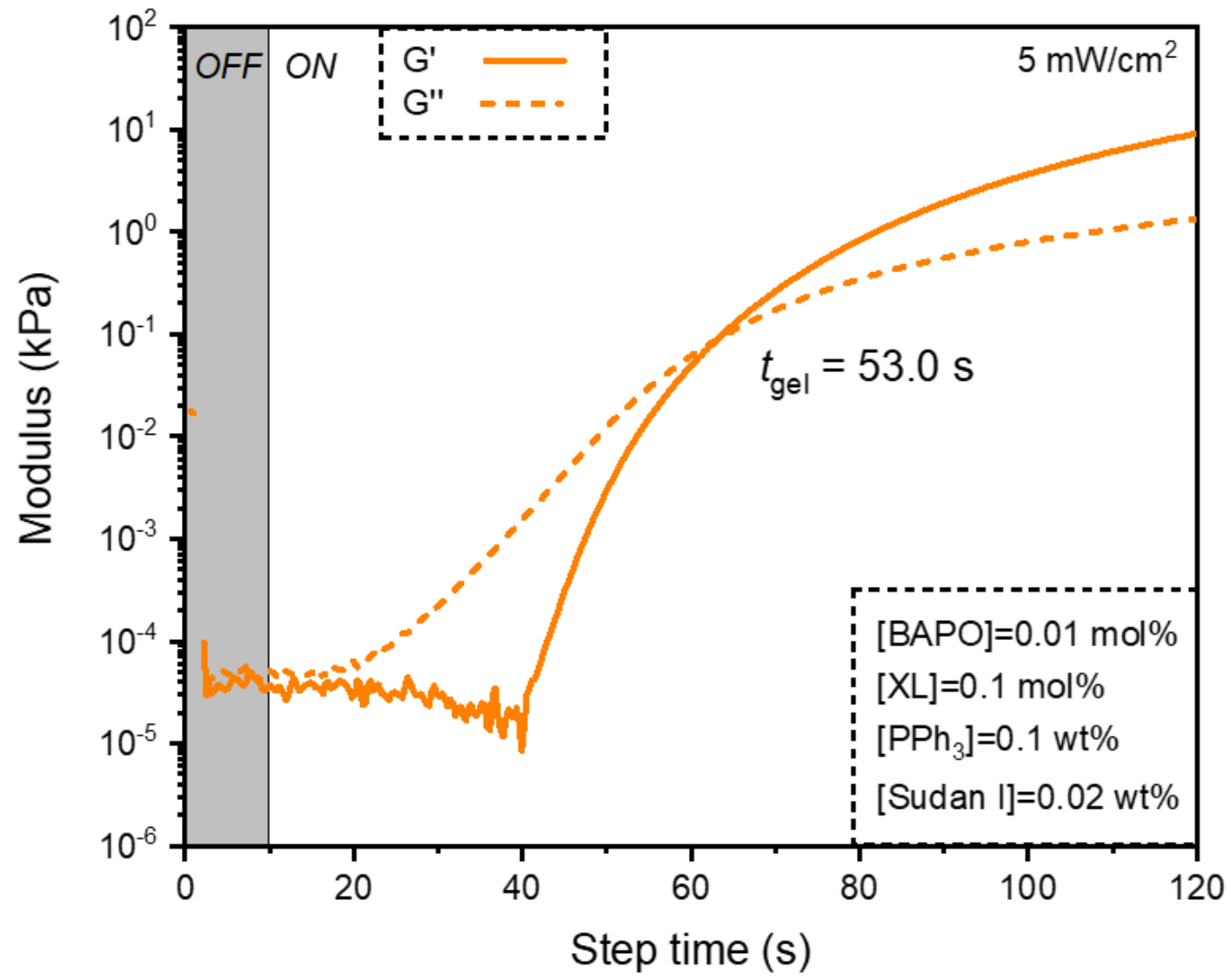

Figure S61. Photo-rheology of elastomer resin. Storage modulus G' and loss modulus G'' obtained via fast sampling to estimate the gel point as the G' and G'' crossover. Resin components: [BAPO] = 0.01 mol%, [XL] = 0.1 mol%, [PPh_3] = 0.1 wt%, [Sudan I] = 0.02 wt%.

S2.13.2 Hydrogel resin

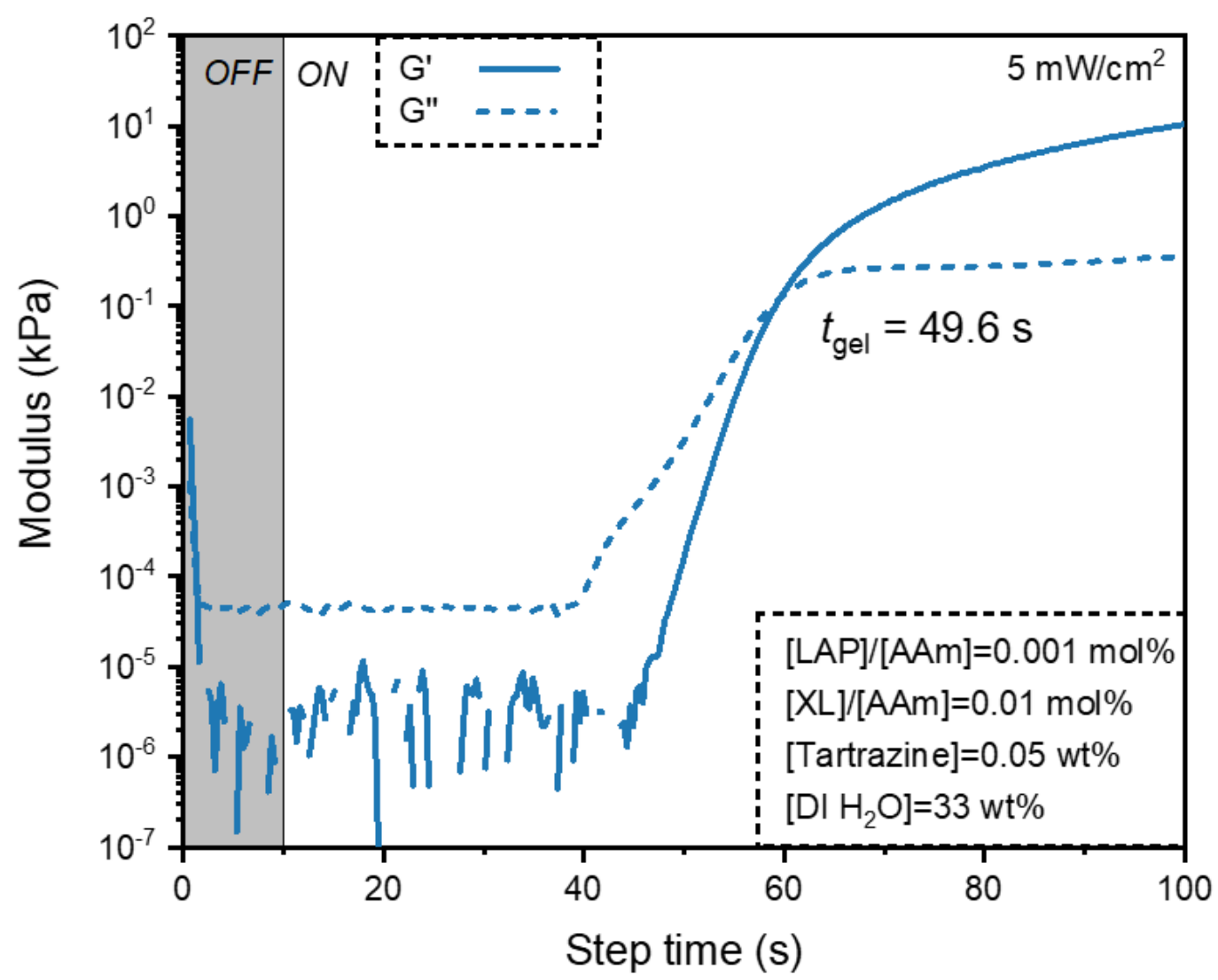

Figure S62. Photo-rheology of hydrogel resin. Storage modulus G' and loss modulus G'' obtained via fast sampling to estimate the gel point as the G' and G'' crossover. Resin components: [LAP]/[AAm] = 0.001 mol%, [XL]/[AAm] = 0.01 mol%, [Tartrazine] = 0.05 wt%, [DI H_2O] = 33 wt%.

S2.13.3 Photoabsorbor Effect

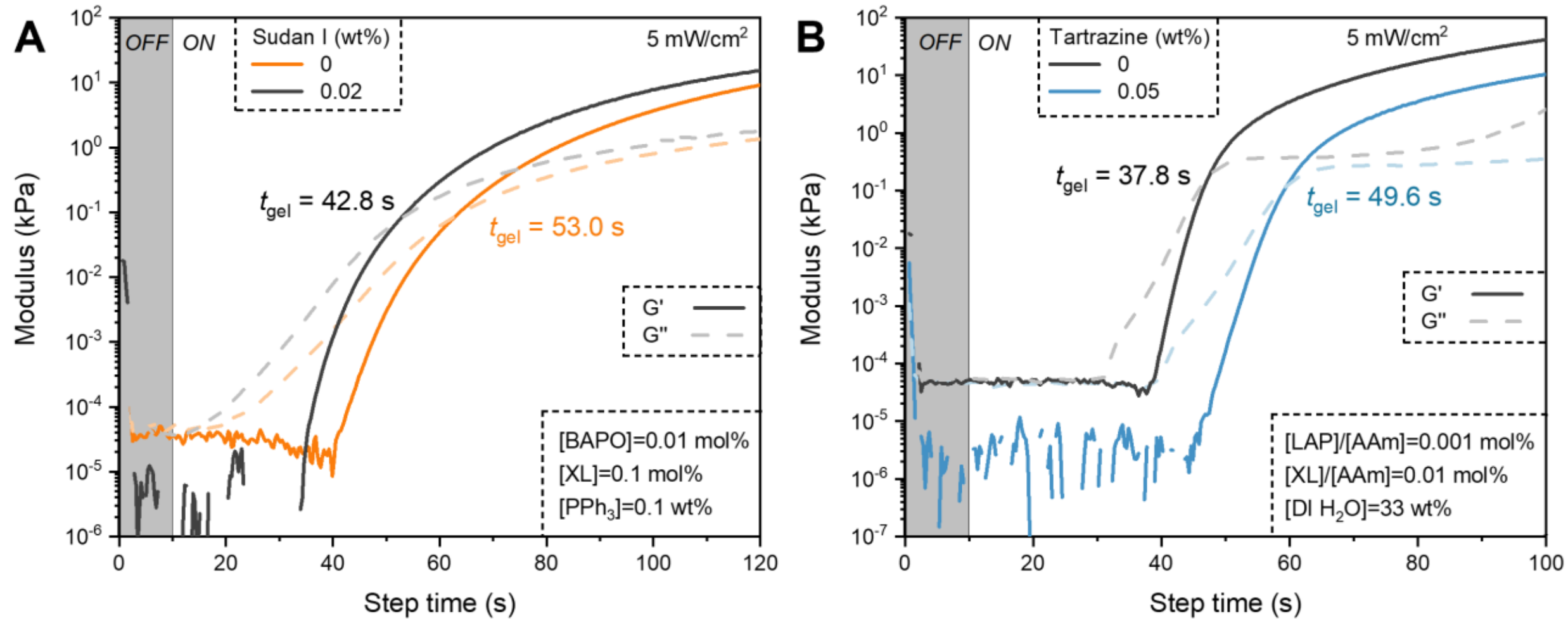

Figure S63. Investigation of photoabsorbor effects on photorheology for elastomer and hydrogel resins. Storage modulus G' and loss modulus G'' obtained via fast sampling to estimate the gel point as the G' and G'' crossover. (A) Elastomer resin components: [BAPO] = 0.01 mol%, [XL] = 0.1 mol%, [PPh_3] = 0.1 wt%, [Sudan I] = 0 or 0.02 wt%. (B) Hydrogel resin components: [LAP]/[AAm] = 0.001 mol%, [XL]/[AAm] = 0.01 mol%, [DI H_2O] = 33 wt%, [Tartrazine] = 0 or 0.05 wt%.

S2.14 Resolution prints of highly entangled elastomers

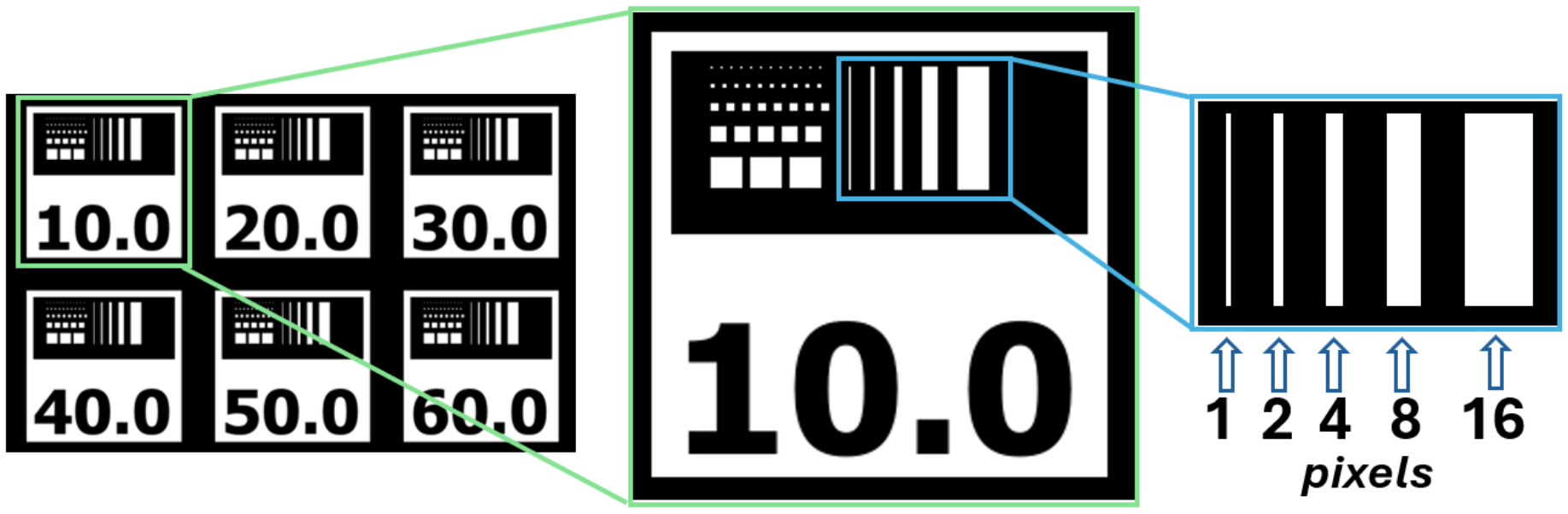

Figure S64. Representative section of a 3D printing .mnf file (slice) that contains different pixel arrays (pixel size = 15.1 µm) used to quantitatively assess 3D printing resolution and feature fidelity. White areas are illuminated and the numbers at the bottom correspond to an exposure time in seconds for every 100 µm thick slice. Prints contained 5 layers (500 µm) of a base and 5 layers (500 µm) of the pattern shown.

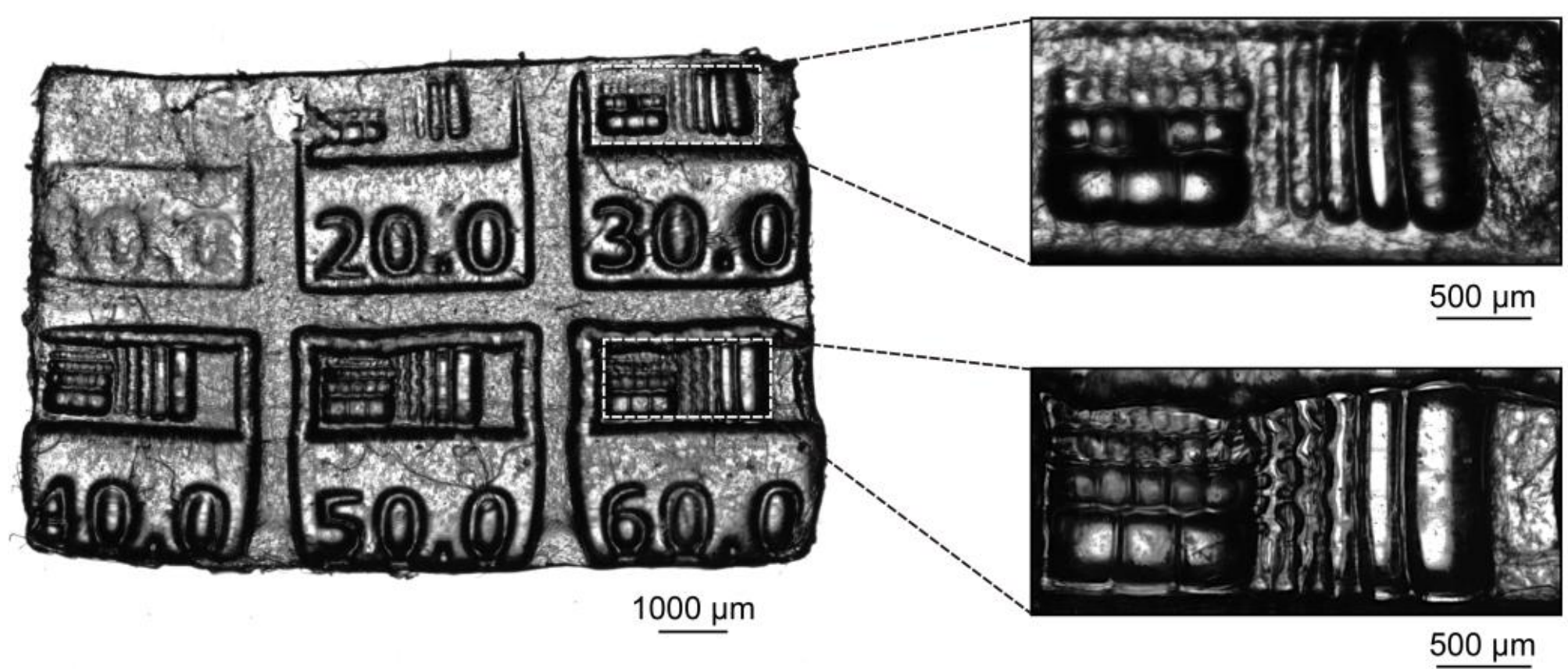

Figure S65. Optical microscope images of highly entangled poly(ethyl acrylate) elastomer prints prepared using [BAPO] = 0.01 mol%, [XL] = 0.1 mol%, [PPh_3] = 0.1 wt%, [Sudan I] = 0.02 wt%. Printing condition: 100 µm per layer, 5 mW/cm^2 365 nm LED. Smallest resolved features for each time point (20, 30, 40, 50, 60s) were 152, 113, 96, 97, 96 µm. After printing, samples were dried at 40 ºC for 12 hours (no UV post-curing).

Table S41. Summary of the smallest apparent features at each time point of the 3D resolution prints for highly entangled poly(ethyl acrylate) samples (**Figure S65**).

Time Point	20s	30s	40s	50s	60s
Measured Feature Size (µm)	152 ± 9	113 ± 8	96 ± 3	97 ± 4	96 ± 3

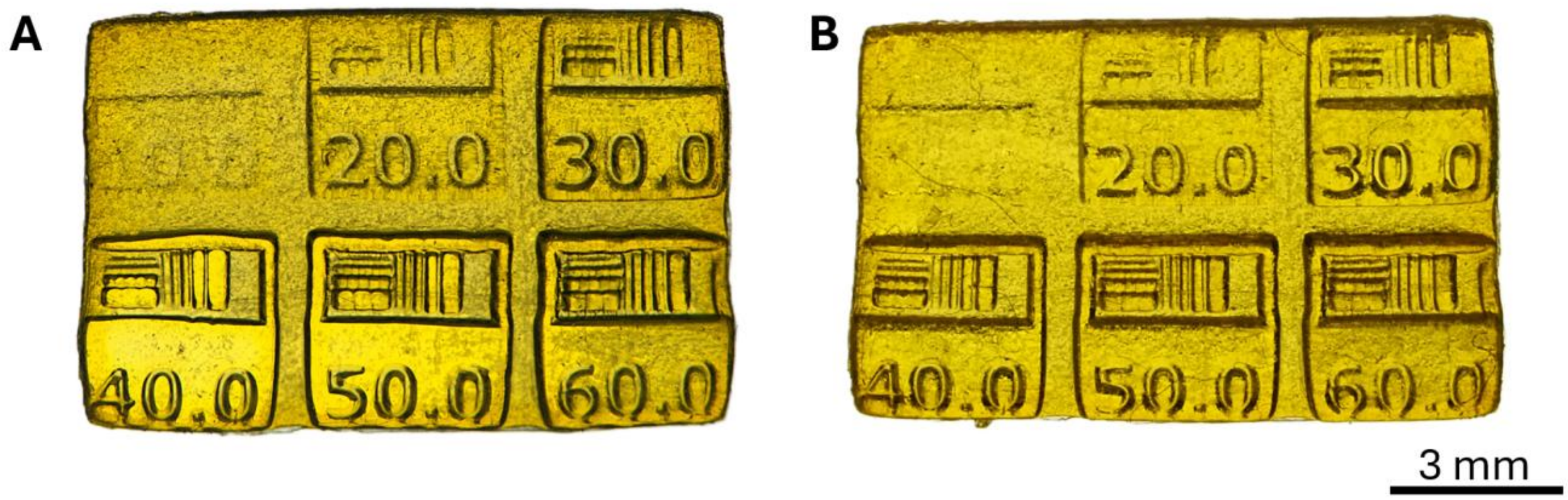

Figure S66. Camera images of poly(ethyl acrylate) elastomer prints (A) directly after printing and (B) after drying in a vacuum oven at 40 ºC for 12 hours without (no UV post-curing). Resins contained [BAPO] = 0.01 mol%, [XL] = 0.1 mol%, [PPh_3] = 0.1 wt%, and [Sudan I] = 0.02 wt%. Printing condition: 100 µm per layer, 5 mW/cm^2 365 nm LED.

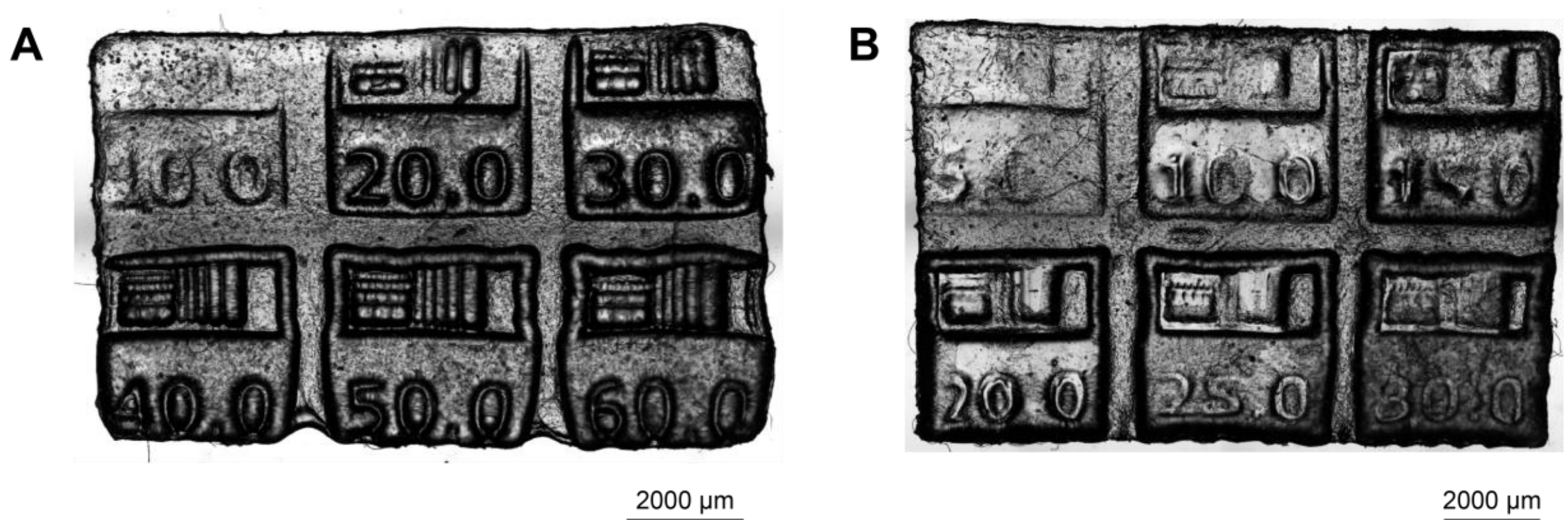

Figure S67. Optical microscope images of highly entangled poly(ethyl acrylate) elastomer prints prepared using [BAPO] = 0.1 mol%, [XL] = 0.1 mol%, [PPh_3] = 0.1 wt%, [Sudan I] = 0.02 wt%. (A) Printing condition: 100 µm per layer, 0.5 mW/cm^2 365 nm LED, 10 second increments. After printing, samples were dried at 40 ºC for 12 hours (no UV post-curing). (B) Printing condition: 100 µm per layer, 5 mW/cm^2 365 nm LED. After printing, samples were dried at 40 ºC for 12 hours (no UV post-curing).

S2.15 Cure depth of highly entangled elastomer resin

To characterize the relationship between cure depth and light dose, Jacob's working curves were obtained using the same 3D printer without a build platform. For each test, a single projected image containing six square patterns (5 mm × 5 mm) was applied to the resin using 365 nm light at 5 mW/cm^2. All six squares within a given test were exposed for the same duration, and the exposure time was varied between tests to obtain cure depths at different light doses.

For the elastomer system without post-curing, resin was added to a vat with an FEP film at the bottom as the base. For each test, resin was exposed to the projected pattern for the specified duration.
After exposure, excess resin was removed, and the printed objects were carefully rinsed three times with methanol. The cured features remained on the FEP film at the bottom of the vat, and the film was cut from the vat with a razor blade and placed under vacuum for 24 h to remove residual unreacted monomer prior to thickness measurement.

For the elastomer system with a 10 min post-cure at 5 mW/cm^2, resin was added to a vat with a silicone film at the bottom as the base to minimize the surface tension. For each test, resin was exposed to the projected pattern for the specified duration. After exposure, excess resin was removed, and the printed objects were placed inside the 365 nm lightbox for 10 min post-cure at 5 mW/cm^2. Then the film was cut from the vat with a razor blade and placed under vacuum for 24 h to remove residual unreacted monomer prior to thickness measurement.

Thickness was measured using a TA Discovery Hybrid Rheometer (HR2) by applying compression until an axial force of 0.2 N was reached. Cure depth (C_d, µm), was plotted verses light dose (E, mJ/cm^2) on a natural logarithmic scale (ln scale). The data were fitted using the Jacob's working curve equation (**Eq. S9**), where the slope represents the penetration depth (D_p, µm) and the x-intercept the critical energy (E_c, mJ/cm^2):

$$C_d = D_p ln\left(\frac{E}{E_c}\right) \quad \textbf{Eq. S9}$$

For the elastomer system, the cure depth versus ln(E) relationship shows two apparent linear regions, which may be attributed to (i) differences in volumetric shrinkage associated with variations in crosslink density at different light doses[S13], and (ii) potential differences in network swelling arising from varying exposure times and interaction with surrounding monomer.

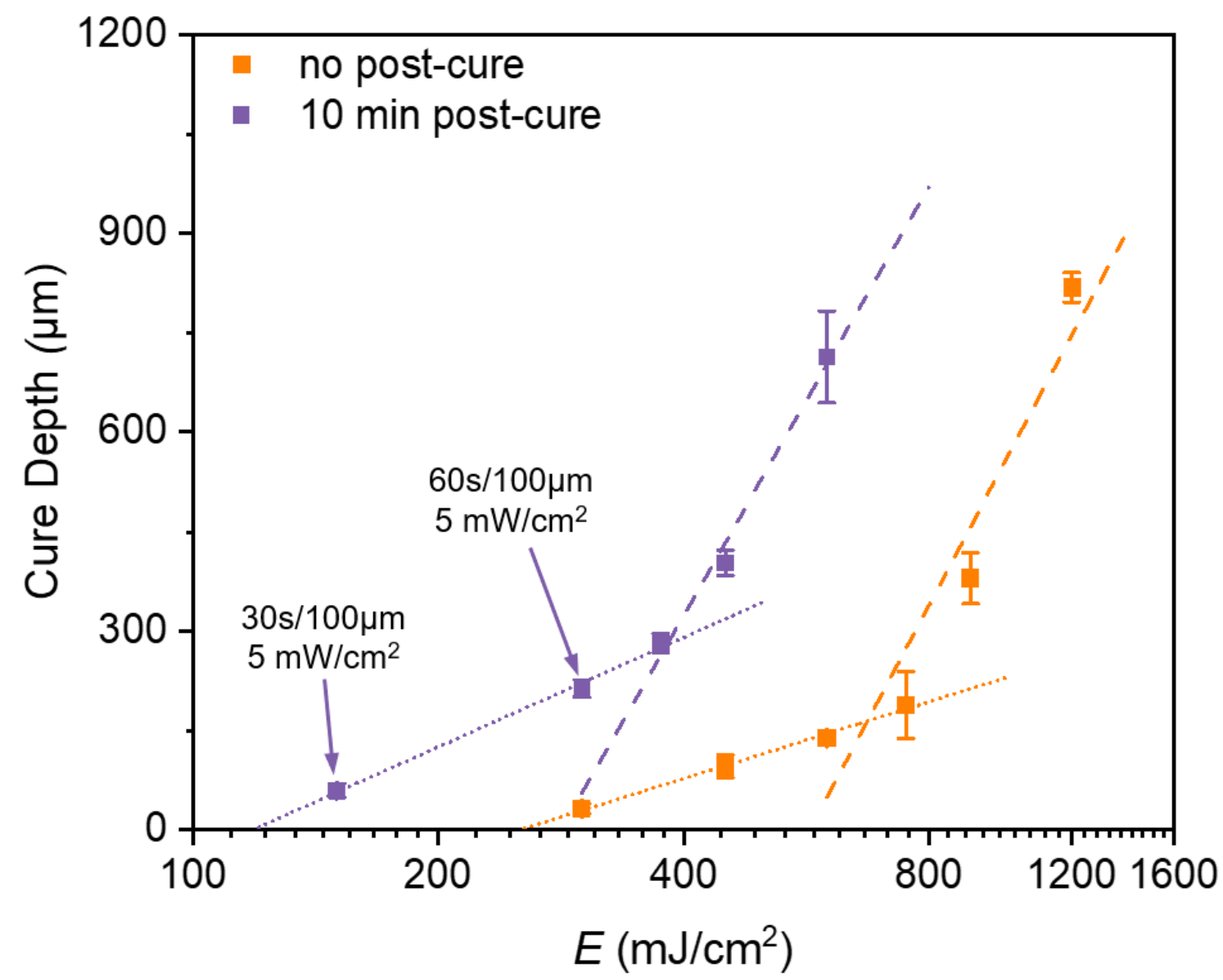

Figure S68. Jacob's working curves for ethyl acrylate resin under 5 mW/cm^2 365 nm LED irradiation. [BAPO] = 0.01 mol%, [XL] = 0.1 mol%, [PPh_3] = 0.1 wt%, [Sudan I] = 0.02 wt%. Data points represent averages (n = 6) with error bars ± 1 standard deviation from the mean. Dashed lines represent distinct Jacob's working curves that result from different dosage regimes.

S2.16 Resolution prints of highly entangled hydrogels

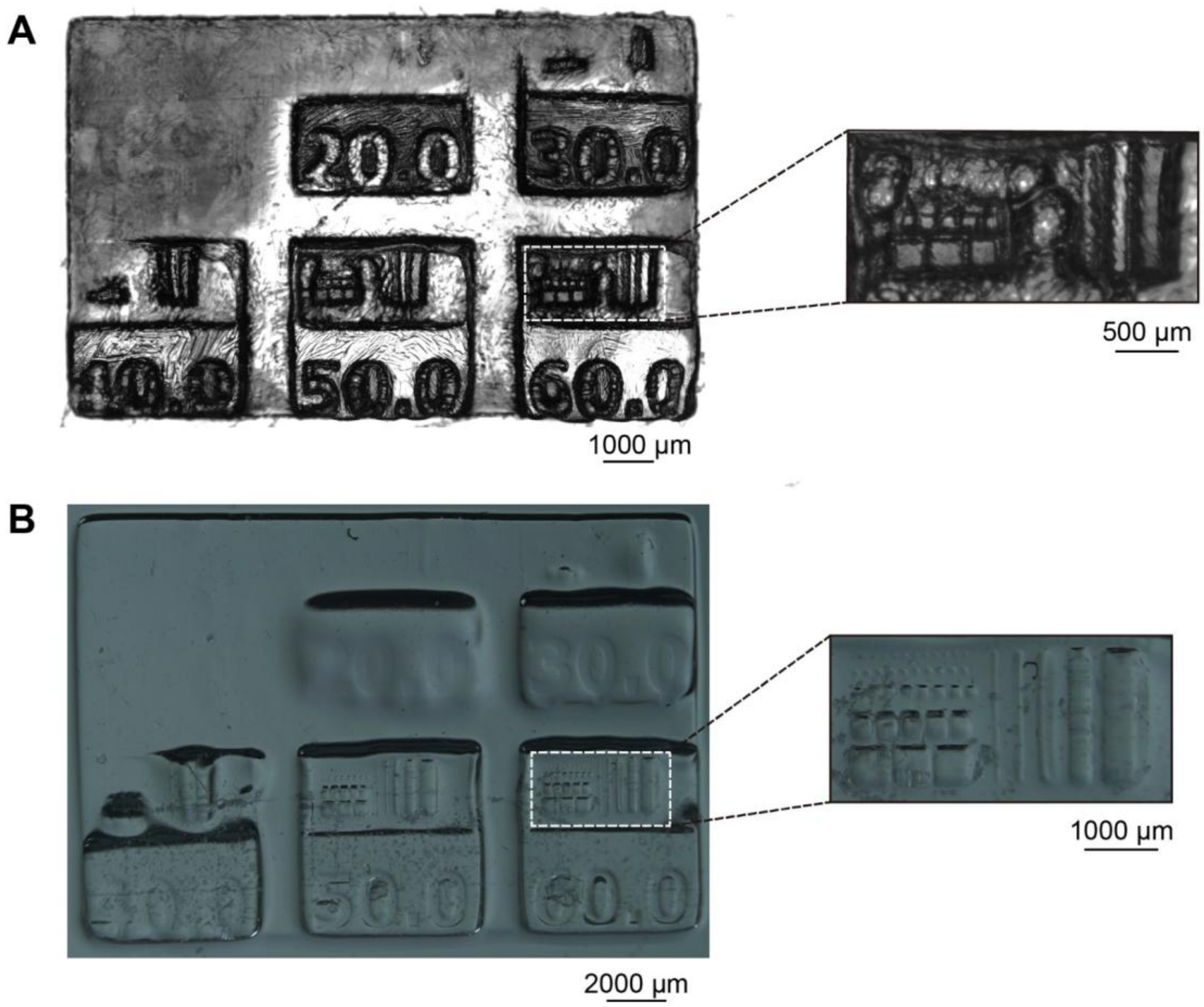

Figure S69. Optical microscope images of hydrogel resolution prints from m[AAm]/m[H_2O] = 2:1, [LAP]/[AAm] = 0.001 mol%, [XL]/[AAm] = 0.01 mol%, and Tartrazine=0.05 wt%. Printing condition: 100 µm per layer, 5 mW/cm^2 365 nm LED, 35 °C. (A) As-printed sample and (B) fully-hydrated sample. After printing, samples were immersed in deionized water for 48 hours (no UV post-curing). Smallest feature resolved on the 60s time point for the as-printed sample is ~100µm.

Table S42. Summary of the apparent features at each time point of the 3D resolution prints for fully-hydrated highly entangled polyacrylamide samples (**Figure S69B**).

Printing File Feature Size (µm)	Fully-hydrated Sample Feature Sizes at Different Time Points (µm)			
	30s	40s	50s	60s
15.1	N/A	N/A	N/A	69 ± 2
30.2	N/A	N/A	95 ± 4	108 ± 3
60.4	N/A	N/A	143 ± 12	164 ± 8
120.8	N/A	414 ± 17	351 ± 15	333 ± 19
241.6	430 ± 25	463 ± 35	615 ± 17	608 ± 25

S2.17 Cure depth of highly entangled hydrogels resins

For the hydrogel system, resin was added to a plastic Petri dish. For each test, resin was exposed to the projected pattern for the specified duration. After exposure, excess resin was removed, and the printed objects were carefully rinsed three times with methanol. Thickness was measured immediately after washing using a depth gauge (Mitutoyo 543-783B10 Absolute Digimatic Indicator).

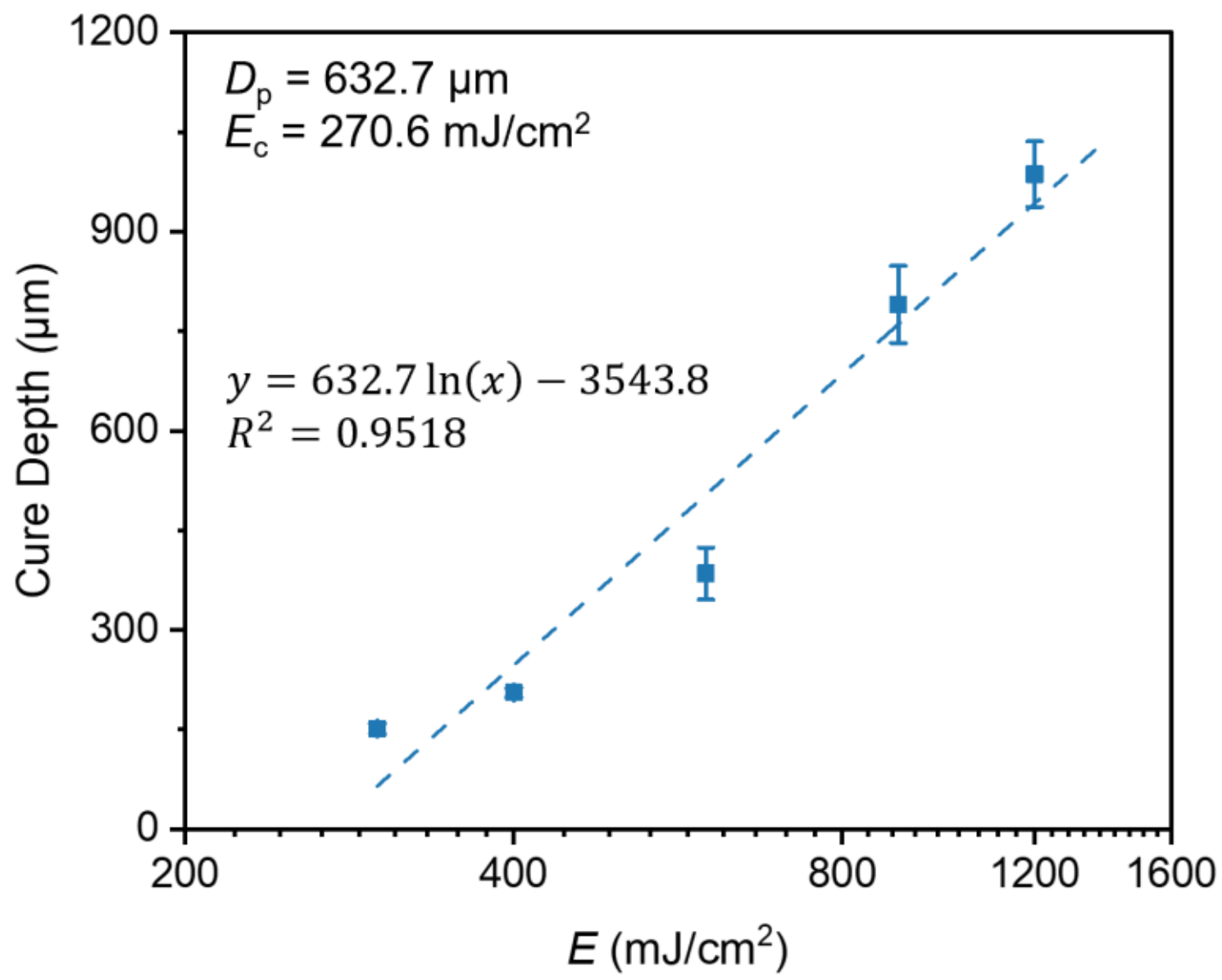

Figure S70. Jacob's working curve for hydrogel system with 365 nm LED at an intensity of 5 mW/cm^2. [LAP]/[AAm] = 0.001 mol%, [XL]/[AAm] = 0.01 mol%, [Tartrazine] = 0.05 wt%, [DI H_2O] = 33 wt%, 35 °C. Data points represent averages (n = 6) with error bars ± 1 standard deviation from the mean, fit with a working curve (dashed line).

S2.18 Shelf life of the 3D printing resin

To assess shelf life, resins were stored in the dark at room temperature for up to 14 days. Photopolymerization kinetics were characterized every 7 days using real-time FTIR, revealing no significant changes in the apparent polymerization rates of either the elastomer or hydrogel resins.

S2.18.1 Elastomer resin

The elastomer resin contained ethyl acrylate, [BAPO] = 0.01 mol%, [XL] = 0.1 mol%, [PPh_3] = 0.1 wt%, [Sudan I] = 0.02 wt%.

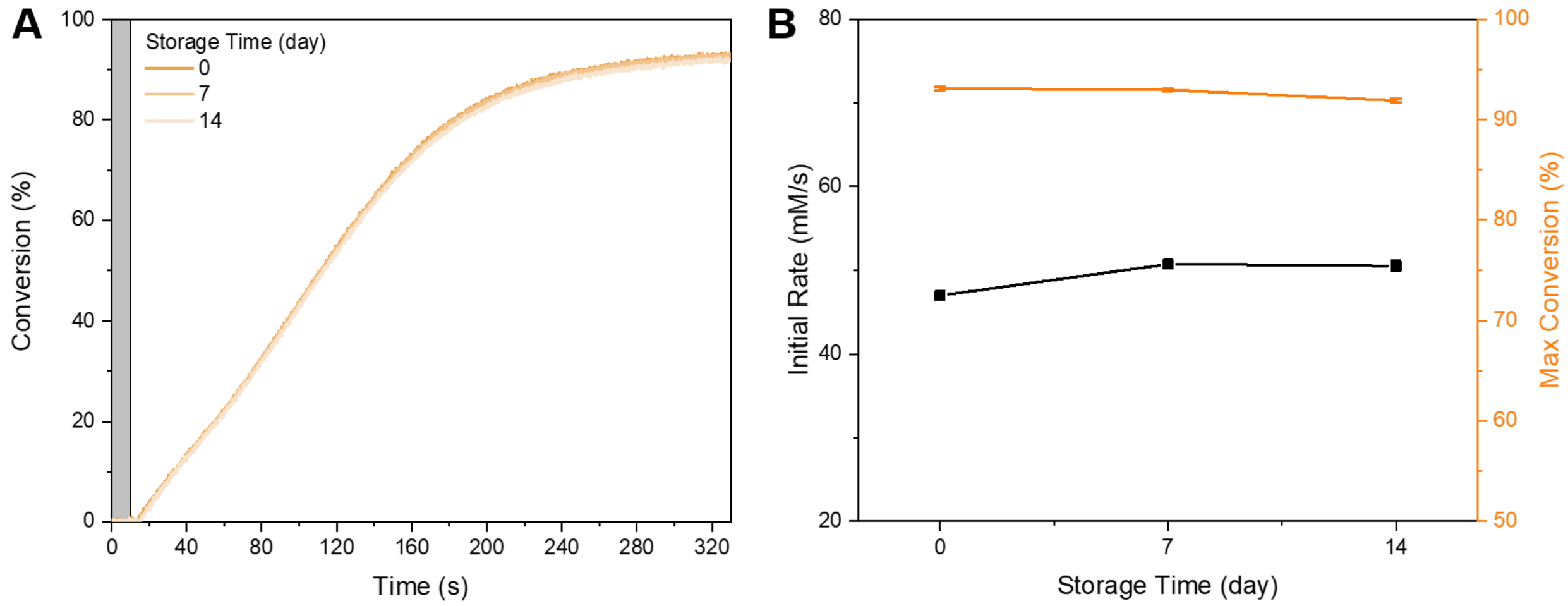

Figure S71. Kinetics of elastomer 3D printing resin stored for different time periods (0, 7, and 14 days). The resin was irradiated under 5 mW/cm^2 365 nm LED. (A) Representative conversion vs time plot of ethyl acrylate polymerization obtained via real-time FTIR spectroscopy. Samples were 100 µm thick. (B) Initial rate and max conversion at the end of the irradiation (320 s) after varying storage time. Symbols are averages ($n \geq 3$) with error bars representing ±1 standard deviation from the mean.

Table S43. Tabulated results from **Figure S71A**. Kinetics of highly entangled elastomer 3D printing resin irradiated with 5 mW/cm^2 365 nm LED for 320 s. Samples were 100 µm thick. Values are averages ($n \geq 3$) with ±1 standard deviation from the mean provided.

Storage Time (day)	Initial Rate (%s^{-1})	Initial Rate (mM/s)	ρ_{max} in run time (%)	Inhibition time (s)
0	0.50 ± 0.01	46.9 ± 0.6	93.1 ± 0.2	5.1 ± 0.3
7	0.54 ± <0.01	50.7 ± 0.2	93.0 ± 0.1	5.9 ± 0.4
14	0.54 ± 0.01	50.5 ± 0.7	91.9 ± 0.2	5.3 ± 0.6

S2.18.1 Hydrogel resin

The hydrogel resin contained [LAP]/[AAm] = 0.001 mol%, [XL]/[AAm] = 0.01 mol%, [Tartrazine] = 0.05 wt%, [DI H_2O] = 33 wt%.

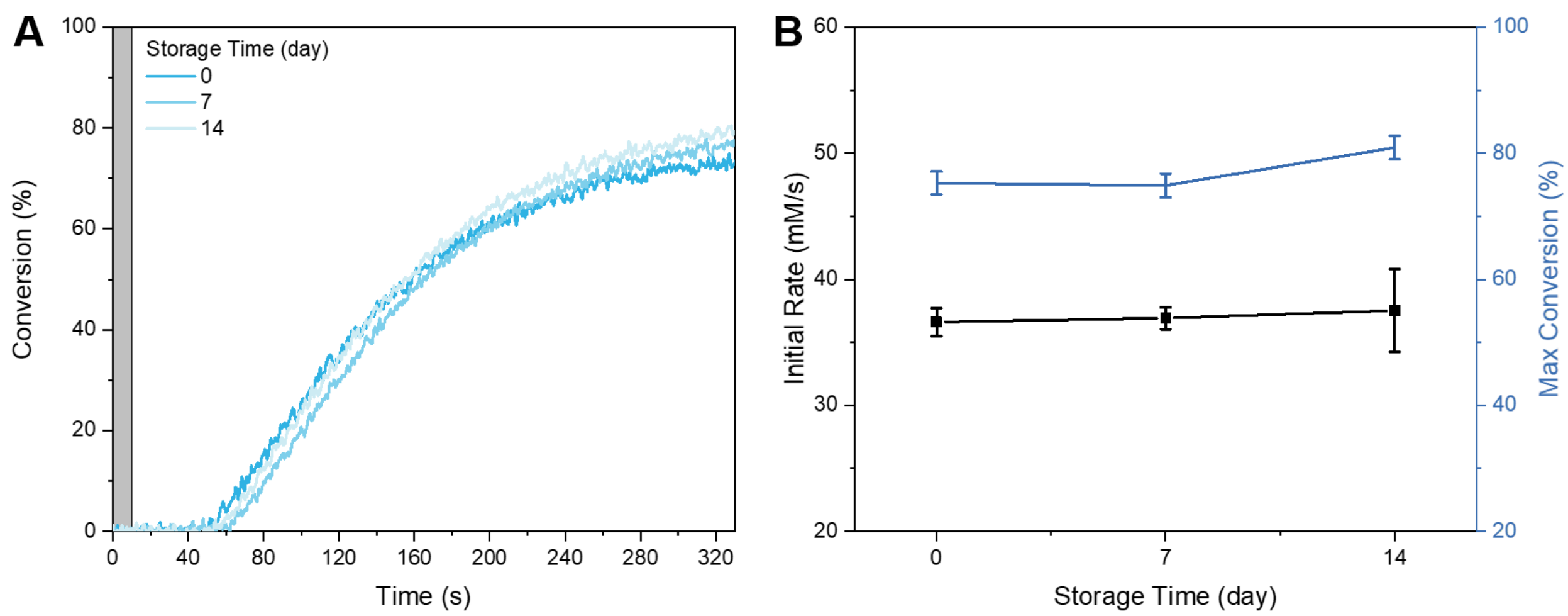

Figure S72. Kinetics of hydrogel 3D printing resin stored for different time periods (0, 7, and 14 days). The resin was irradiated under 5 mW/cm^2 365 nm LED. (A) Representative conversion vs time plot of ethyl acrylate polymerization obtained via real-time FTIR spectroscopy. Samples were 100 µm thick. (B) Initial rate and max conversion at the end of the irradiation (320 s) after varying storage time. Symbols are averages (n ≥3) with error bars representing ±1 standard deviation from the mean.

Table S44. Tabulated results from **Figure S72A**. Kinetics of highly entangled elastomer 3D printing resin irradiated with 5 mW/cm^2 365 nm LED for 320 s. Samples were 100 µm thick. Values are averages (n ≥3) with ±1 standard deviation from the mean provided.

Storage Time (day)	Initial Rate (%s^{-1})	Initial Rate (mM/s)	ρ_{max} in run time (%)	Inhibition time (s)
0	0.39 ± 0.01	37 ± 1	75 ± 2	54 ± 3
7	0.39 ± 0.01	36.9 ± 0.9	75 ± 2	59 ± 7
14	0.40 ± 0.03	38 ± 3	81 ± 2	47 ± 1

S2.19 Additional 3D printed structures

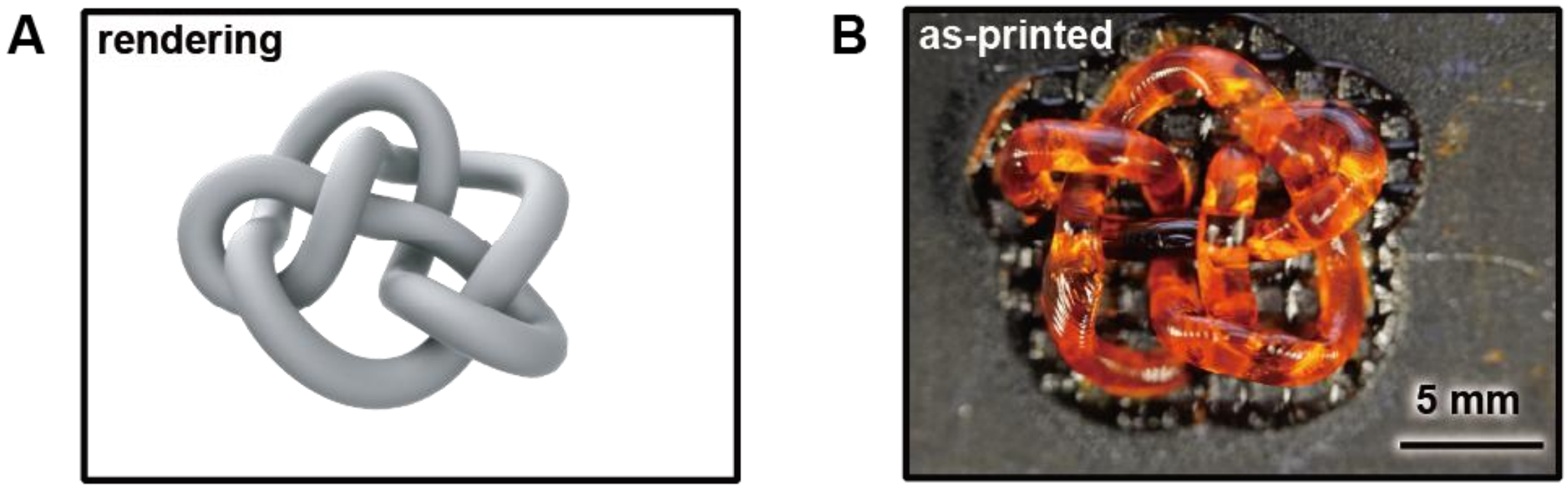

Figure S73. 7_7 knot Print[S14]. (A) Digital rendering of print file and (B) directly after printing photograph of an entangled poly(ethyl acrylate) as-printed knot. Conditions: [BAPO] = 0.01 mol%, [XL] = 0.1 mol%, [PPh_3] = 0.1 wt%, [Sudan I] = 0.02 wt%, 5 mW/cm^2 365 nm LED, 30 s per 100 µm layer.

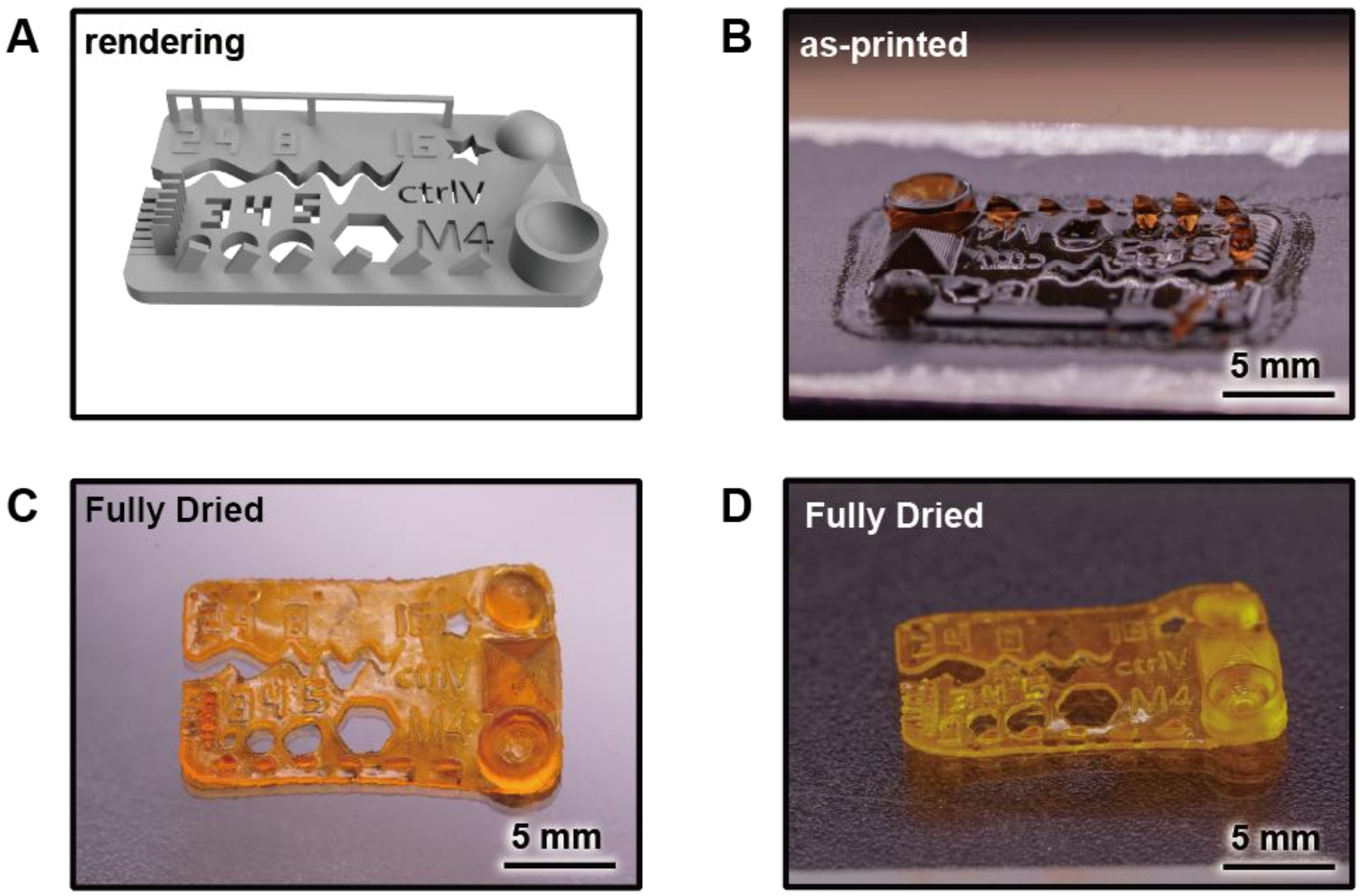

Figure S74. "Test your printer" print.[S15] (A) Digital rendering of print file and (B) directly after printing photograph of an entangled poly(ethyl acrylate) as-printed "Test your printer" print. Conditions: [BAPO] = 0.01 mol%, [XL] = 0.1 mol%, [PPh_3] = 0.1 wt%, [Sudan I] = 0.02 wt%, 5 mW/cm^2 365 nm LED, 30 s per 100 µm layer. (C/D) Photographs of the print after the sample shown in (B) was dried at 40 °C under vacuum for 24 h (no UV post-curing).

S2.20 Mechanical properties of 3D printed highly entangled elastomers

Table S45. Summary of the mechanical properties of highly entangled elastomers 3D printed with a 365 nm LED (5 mW cm^{-2}) at varying exposure times per 100 µm layer. Sol-gel fractions given as gel content. Values are averages ($n \geq 3$) with ±1 standard deviation from the mean provided.

Exposure Time	Sol-Gel Fraction	Young's Modulus E_T (MPa)	Strain at break ε_f (%)	Tensile Strength σ_m (MPa)	Modulus of Toughness U_t (MJ/m^3)	Hysteresis (%)	Hardness (Shore A)	Fracture Toughness G_c (kJ/m^2)	Fracto-cohesive length $L_f = G_c / W_F$ (mm)
30s	0.77 ± 0.01	0.80 ± 0.03	900 ± 34	1.6 ± 0.1	6.0 ± 0.6	/	/	0.9 ± 0.2	0.15 ± 0.04
60s	0.87 ± <0.01	0.84 ± 0.03	800 ± 78	3.0 ± 0.6	9 ± 2	/	/	0.81 ± 0.09	0.09 ± 0.02
30s *(post-cure)*	0.91 ± 0.01	0.79 ± 0.01	586 ± 35	1.9 ± 0.1	4.6 ± 0.4	10.7	26.3 ± 0.3	0.8 ± 0.1	0.18 ± 0.03
60s *(post-cure)*	0.95 ± 0.01	0.89 ± 0.04	656 ± 46	2.1 ± 0.4	6 ± 1	9.2	/	0.8 ± 0.2	0.13 ± 0.04

Table S46. Summary of the mechanical properties of highly entangled elastomers 3D printed with a 365 nm LED (50 mW cm^{-2}) at varying exposure times per 100 µm layer. Values are averages ($n \geq 3$) with ±1 standard deviation from the mean provided.

Printing conditions	Young's Modulus E_T (MPa)	Strain at break ε_f (%)	Tensile Strength σ_m (MPa)	Modulus of Toughness U_T (MJ/m^3)
50 mW/cm^2(10s)	0.35 ± 0.04	833 ± 55	1.3 ± 0.3	3.1 ± 0.6
50 mW/cm^2(10s) *post-cure*	0.46 ± 0.05	460 ± 85	0.8 ± 0.2	1.5 ± 0.4

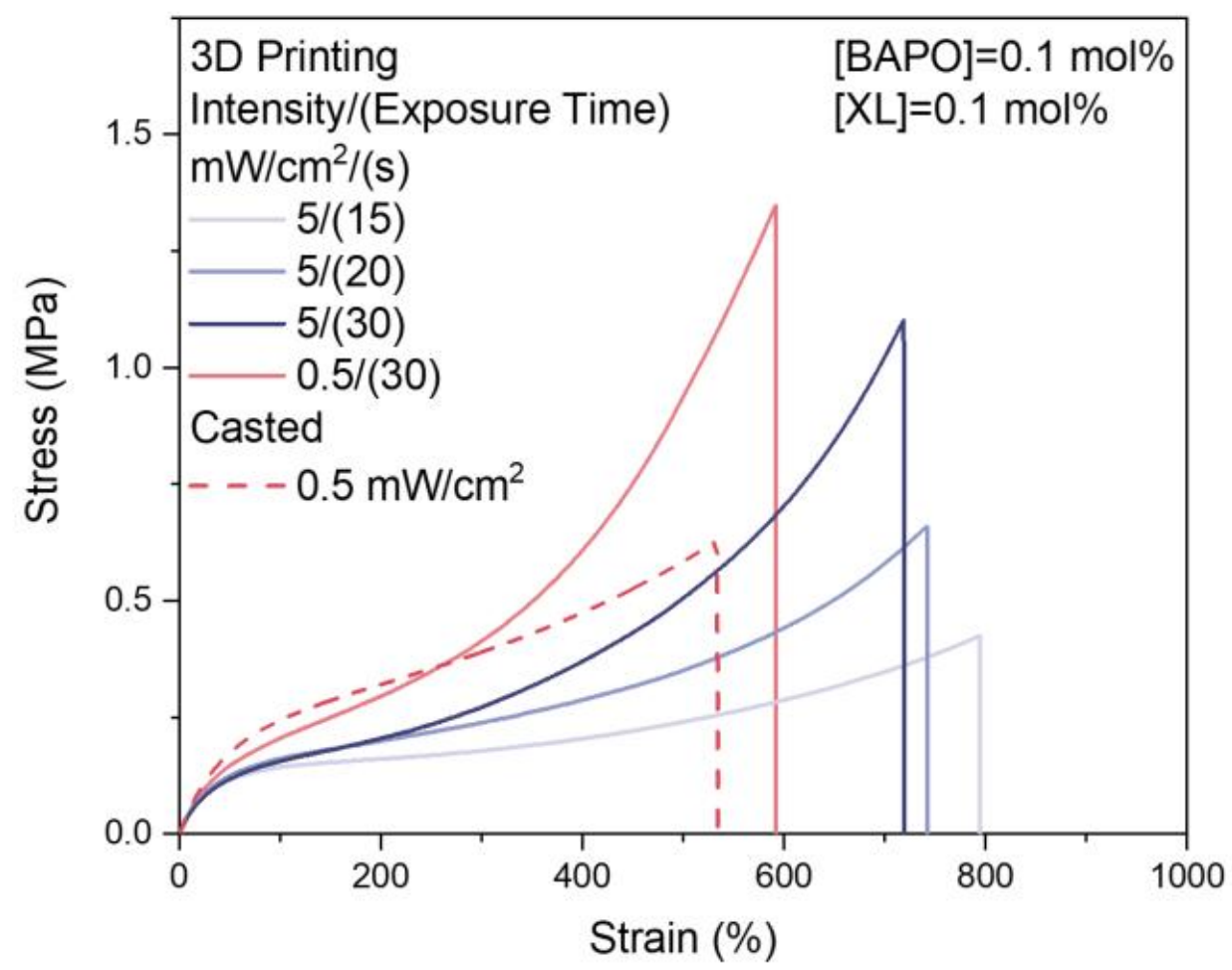

Figure S75. Representative uniaxial tensile stress-strain curves of 3D printed dog bone samples with varying printing conditions (as noted) after post-curing. [BAPO] = 0.1 mol%, [XL] = 0.1 mol%, [PPh_3] = 0.1 wt%, [Sudan I] = 0.02 wt%.

Table S47. Summary of **Figure S75**. Mechanical properties of highly entangled elastomers 3D printed with a 365 nm LED at varying intensity and exposure times per 100 µm layer and after post-curing under 5 mW/cm^2 for 5 min. [BAPO] = 0.1 mol%, [XL] = 0.1 mol%, [PPh_3] = 0.1 wt%, [Sudan I] = 0.02 wt%. Sol-gel fractions given as gel content. Values are averages ($n \geq 3$) with ±1 standard deviation from the mean provided.

Printing conditions	Sol-Gel Fraction	Young's Modulus E_T (MPa)	Strain at break ε_f (%)	Tensile Strength σ_m (MPa)	Modulus of Toughness U_t (MJ/m^3)
0.5 mW/cm^2(30s) *post-cure*	0.92 ± <0.01	0.53 ± 0.02	438 ± 131	0.9 ± 0.4	1.7 ± 0.9
5 mW/cm^2(15s) *post-cure*	0.92 ± 0.02	0.41 ± 0.03	769 ± 38	0.39 ± 0.04	1.6 ± 0.2
5 mW/cm^2(20s) *post-cure*	0.95 ± 0.01	0.46 ± 0.03	701 ± 46	0.58 ± 0.06	2.0 ± 0.3
5 mW/cm^2(30s) *post-cure*	0.95 ± 0.01	0.48 ± 0.02	649 ± 60	0.9 ± 0.2	2.4 ± 0.4

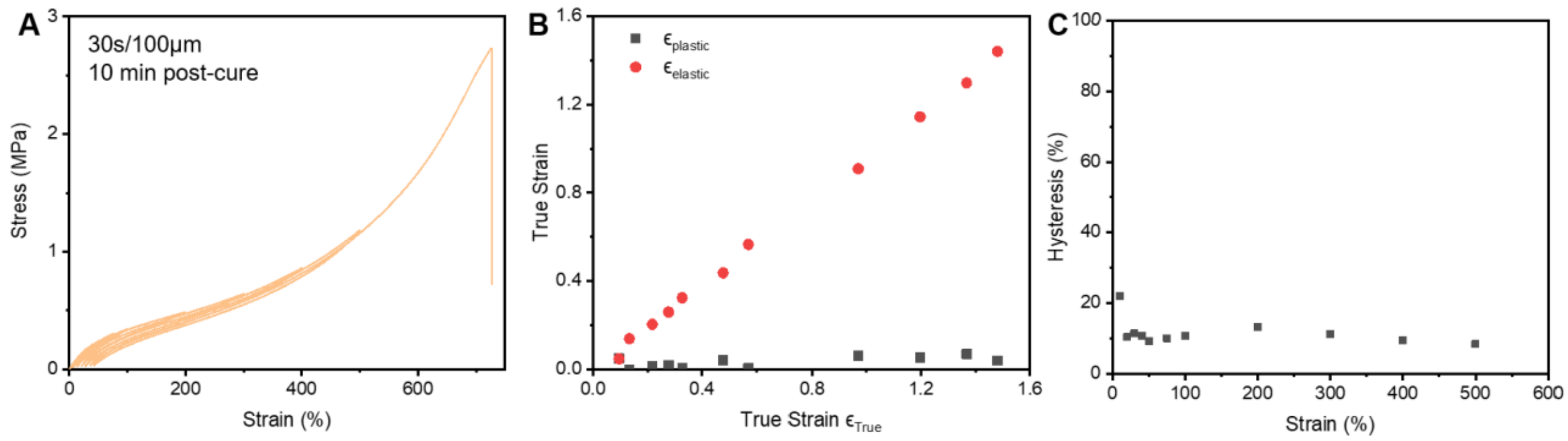

Figure S76. Stepwise cyclic tensile testing of highly entangled poly(ethyl acrylate) dog-bone samples printed at 30 s per 100 µm layer and post-cured under a 365 nm LED (5 mW/cm^2) for 10 min. (A) Stress-strain curve during the stepwise cyclic tensile testing. (B) Corresponding true strain, showing elastic and plastic behavior from the stepwise cyclic tensile testing. (C) Corresponding hysteresis from the stepwise cyclic tensile testing.

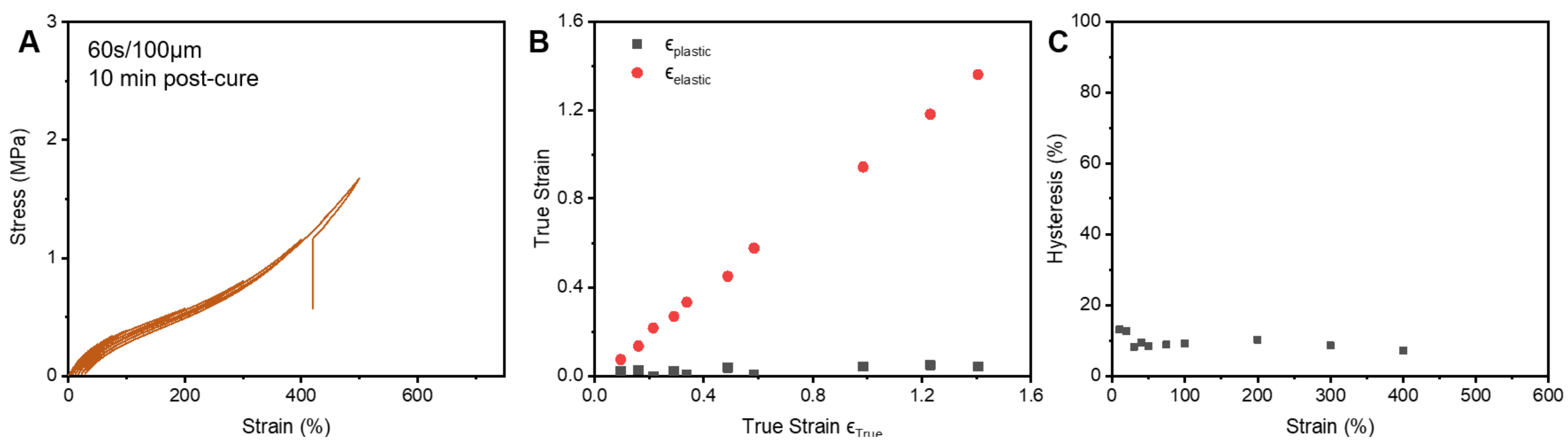

Figure S77. Stepwise cyclic tensile testing of highly entangled poly(ethyl acrylate) dog-bone samples printed at 60 s per 100 µm layer and post-cured under a 365 nm LED (5 mW/cm^2) for 10 min. (A) Stress-strain curve during the stepwise cyclic tensile testing. (B) Corresponding true strain, showing elastic and plastic behavior from the stepwise cyclic tensile testing. (C) Corresponding hysteresis from the stepwise cyclic tensile testing.

S2.21 Resolution prints of commercial resins

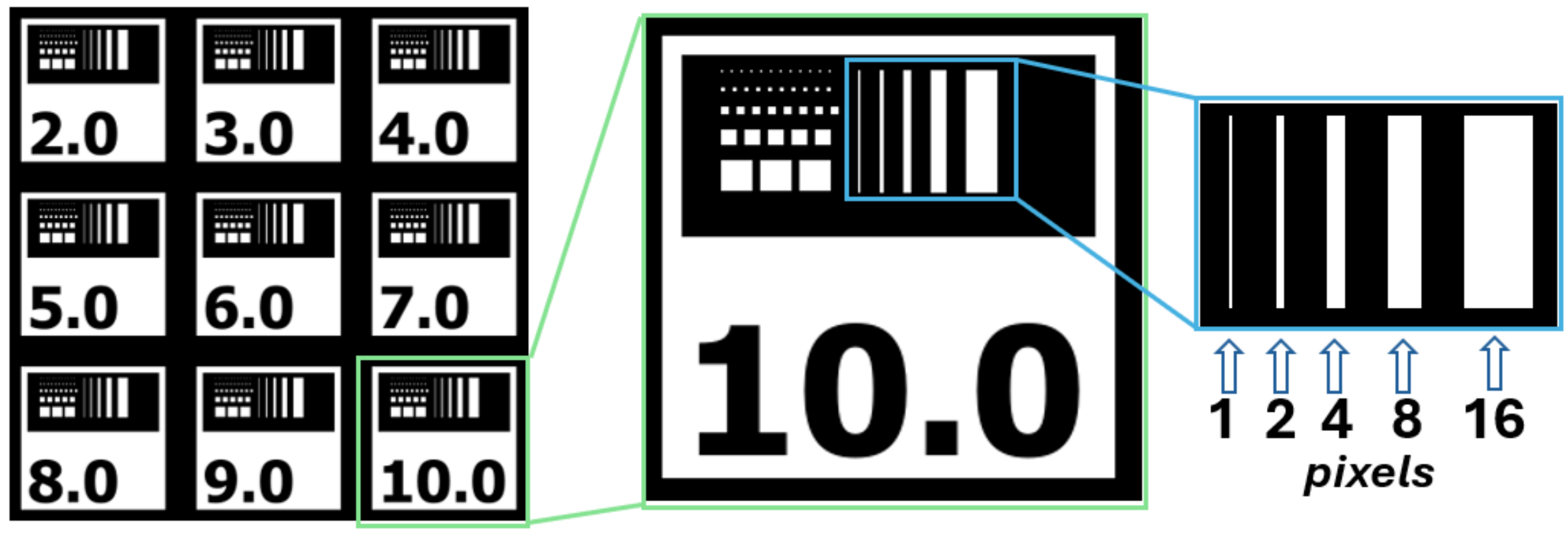

Figure S78. Representative section of a 3D printing .mnf file (slice) that contains different pixel arrays (pixel size = 15.1 µm) used to quantitatively assess 3D printing resolution and feature fidelity. White areas are illuminated and the numbers at the bottom correspond to an exposure time in seconds for every 100 µm thick slice. Prints contained 5 layers (500 µm) of a base and 5 layers (500 µm) of the pattern shown.

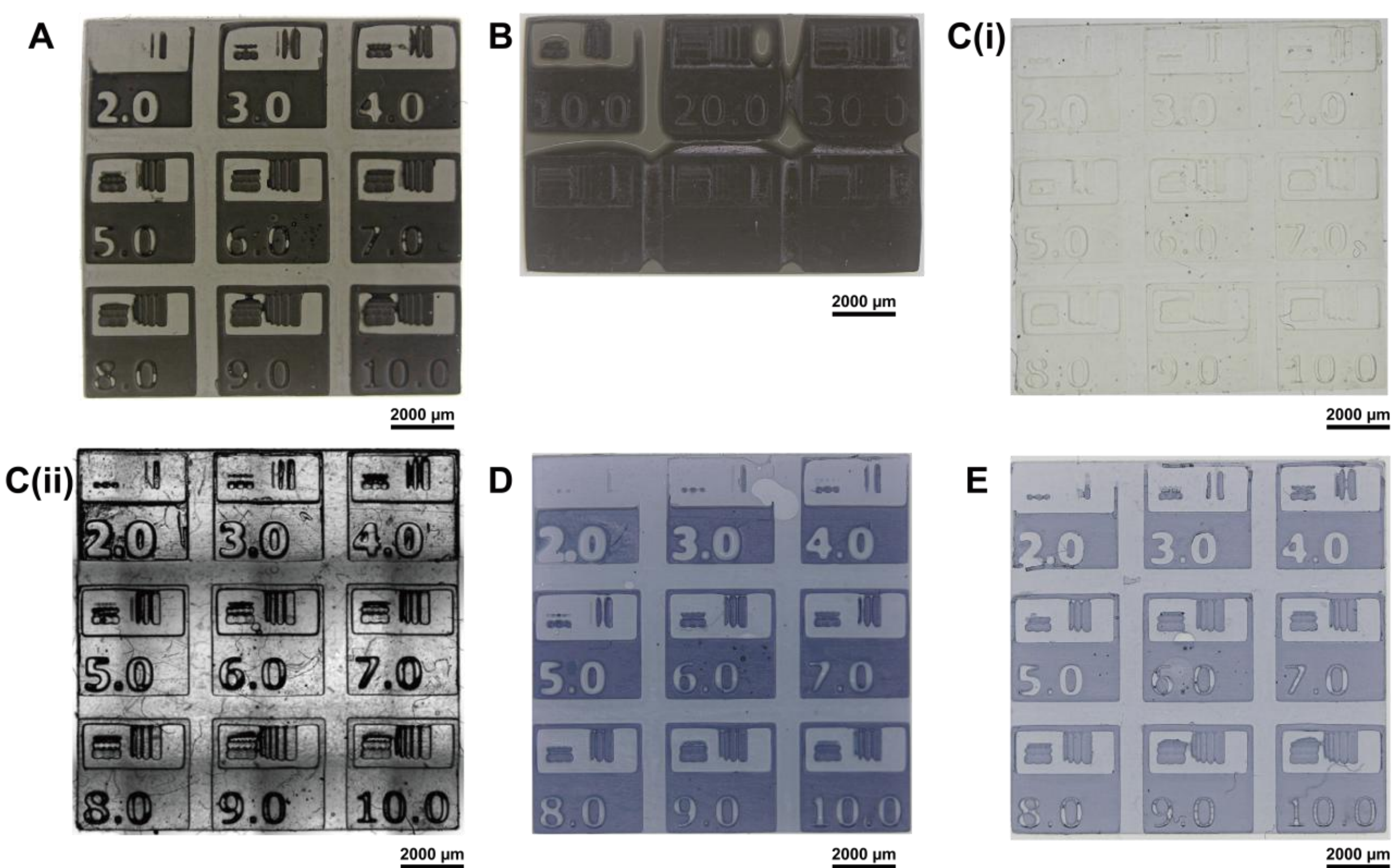

Figure S79. Resolution prints using commercial resins for soft and elastomeric materials. All prints had a slice thickness of 100 µm per layer (no UV post-curing). (A) **FLX-300** (AmeraLabs). Printing condition: 5 mW/cm^2 405 nm LED. (B) **Silicone 40A** (Formlabs). Printing condition: 10 mW/cm^2 405 nm LED. (C) **Elastic 40A** (Formlabs) (i) photograph and (ii) optical microscopy images. Printing condition: 5 mW/cm^2 405 nm LED. (E) **Rebound 55A** (Siraya Tech). Printing condition: 5 mW/cm^2 405 nm LED. (F) **Tenacious Flex Black 70A** (Siraya Tech). Printing condition: 2 mW/cm^2 405 nm LED.

Table S48. Summary from **Figure S79**. Resolution print of commercial resins.

Resin	Wavelength (nm)	Intensity (mW/cm^2)	Exposure time per layer (s)	Smallest Lateral Feature Resolution
FLX-300 (AmeraLabs)	405	5	2-10	~100 µm at 6s/100 µm layer
Silicone 40A (Formlabs)	450	10	10-60	~200 µm at 10s/100 µm layer
Elastic 40A (Formlabs)	405	5	2-10	~100 µm at 6s/100 µm layer
Rebound 55A (Siraya Tech)	450	5	2-10	~150 µm at 6s/100 µm layer
Tenacious Flex Black 70A (Siraya Tech)	450	5	2-10	~100 µm at 6s/100 µm layer

S2.22 Mechanical properties of materials prepared from commercial resins

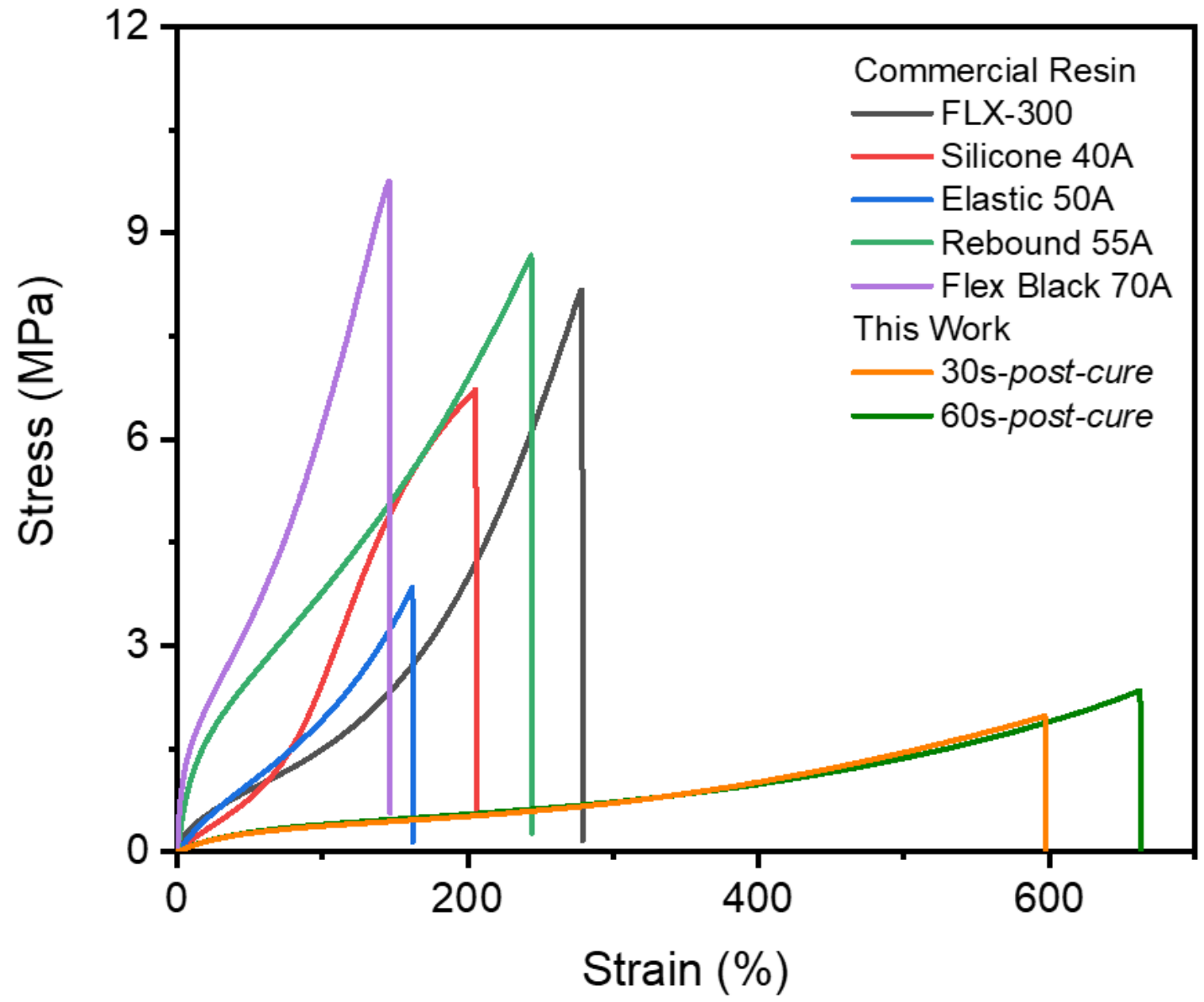

Figure S80. Representative uniaxial tensile stress-strain curves of 3D printed dog bone samples with commercial resins after post curing and drying, together with highly entangled poly(ethyl acrylate) elastomers from this work.

S2.22.1 FLX-300 (AmeraLabs)

Printing conditions: Monoprinter (MONO3-VZ4, monoprinter), 5 mW/cm^2 365 nm LED, 6 s per 100 µm layer.

Post-Curing procedure: The printed samples were rinsed in isopropanol (IPA) for 20 min, air-dried, and post-cured using a Form Cure system for 45 min at 60 °C. The samples were then dried under vacuum at 40 °C for 12 h before testing.

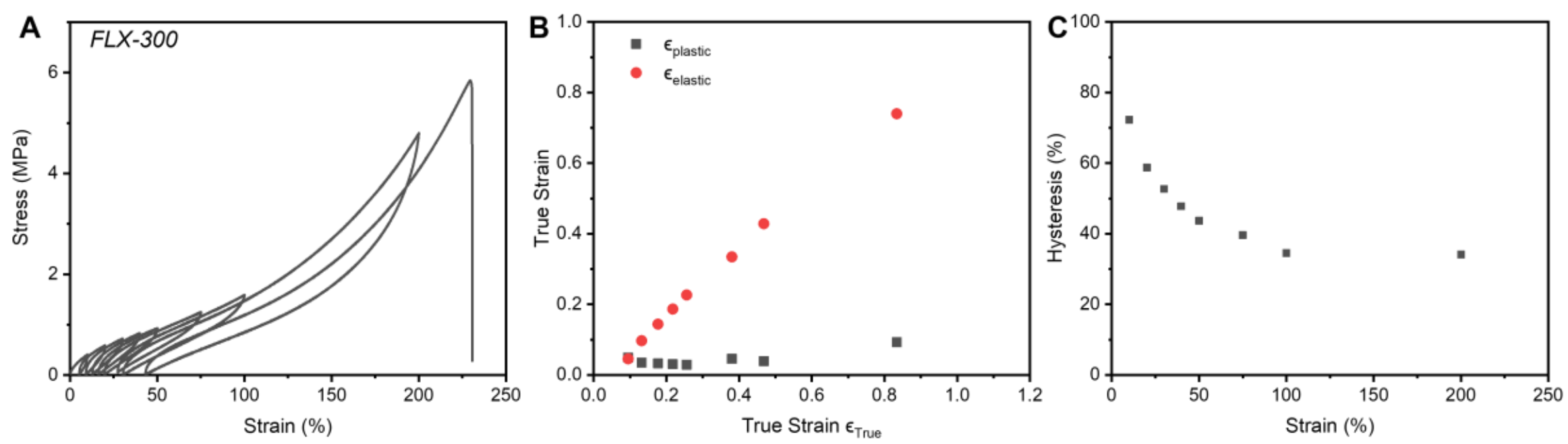

Figure S81. stepwise cyclic tensile testing analysis of printed dog-bone samples with Flex-300 resin. (A) Stress-strain curve during the stepwise cyclic tensile testing. (B) Corresponding true strain, showing elastic and plastic behavior from stepwise cyclic tensile testing. (C) Corresponding hysteresis from stepwise cyclic tensile testing.

S2.22.2 Silicone 40A (Formlabs)

Printing conditions: Form 3 SLA printer with the installed printing condition.

Post-Curing procedure: The printed samples were rinsed in 80% isopropanol (IPA) and 20% *n*-butyl acetate for 20 min, air-dried, and post-cured using a Form Cure system for 45 min at 60 °C in a water bath. The samples were then dried under vacuum at 40 °C for 12 h before testing.

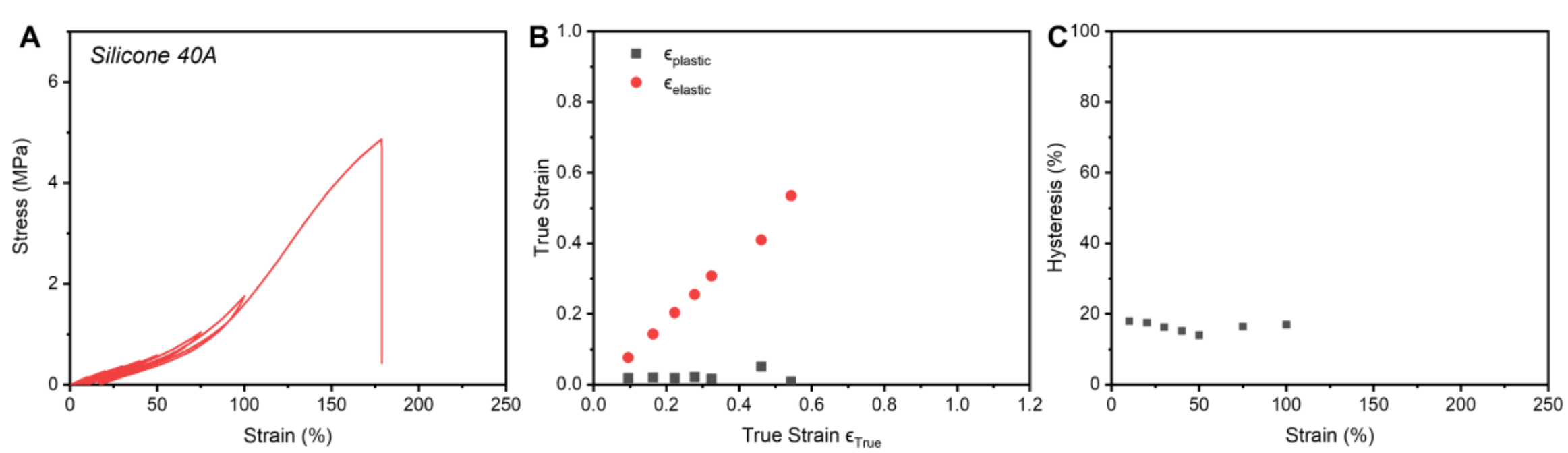

Figure S82. Stepwise cyclic tensile testing of printed dog-bone samples with Silicone 40A resin. (A) Stress-strain curve during the stepwise cyclic tensile testing. (B) Corresponding true strain, showing elastic and plastic behavior from stepwise cyclic tensile testing. (C) Corresponding hysteresis from stepwise cyclic tensile testing.

S2.22.3 Elastic 50A (Formlabs)

Printing conditions: Form 3 SLA printer with the installed printing condition.

Post-Curing procedure: The printed samples were rinsed in isopropanol (IPA) for 20 min, air-dried, and post-cured using a Form Cure system for 30 min at 70 °C in water bath. The samples were then dried under vacuum at 40 °C for 12 h before testing.

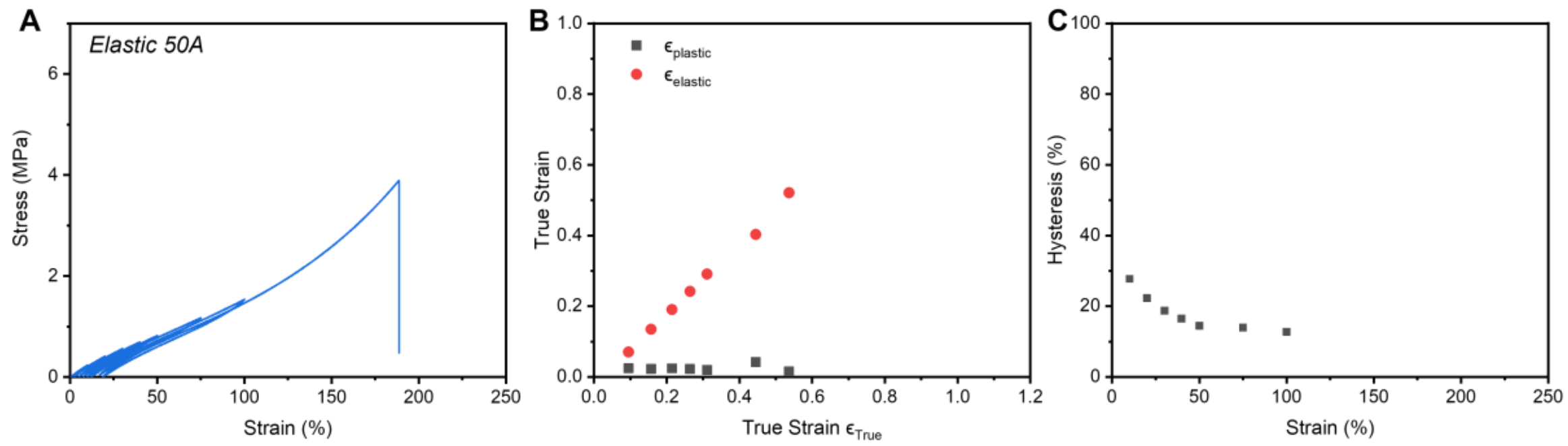

Figure S83. Stepwise cyclic tensile testing of printed dog-bone samples with Elastic 50A resin. (A) Stress-strain curve during the stepwise cyclic tensile testing. (B) Corresponding true strain, showing elastic and plastic behavior from stepwise cyclic tensile testing. (C) Corresponding hysteresis from stepwise cyclic tensile testing.

S2.22.4 Rebound 55A (Siraya Tech)

Printing conditions: Monoprinter (MONO3-VZ4, monoprinter), 5 mW/cm^2 365 nm LED, 6 s per 100 μm layer.

Post-Curing procedure: The printed samples were rinsed in isopropanol (IPA) for 20 min and air-dried, followed by oven drying at 50 °C for 90 min. The samples were then post-cured using an AnyCubic curing system for 25 min and then dried under vacuum at 40 °C for 12 h prior to testing.

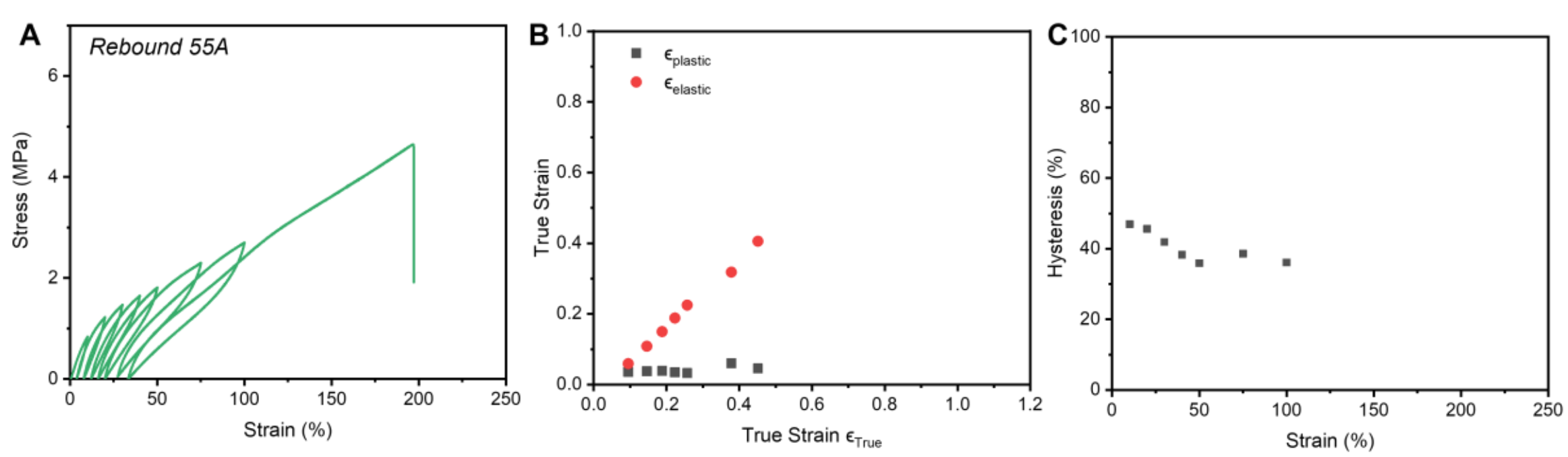

Figure S84. Stepwise cyclic tensile testing of printed dog-bone samples with Rebound 55A resin. (A) Stress-strain curve during the stepwise cyclic tensile testing. (B) Corresponding true strain, showing elastic and plastic behavior from stepwise cyclic tensile testing. (C) Corresponding hysteresis from stepwise cyclic tensile testing.

S2.22.5 Tenacious Flex Black 70A (Siraya Tech)

Printing conditions: Monoprinter (MONO3-VZ4, monoprinter), 5 mW/cm^2 365 nm LED, 6 s per 100 µm layer.

Post-Curing procedure: The printed samples were rinsed in ethanol for 3 min and air-dried. This procedure was repeated five times until the surface was no longer sticky. The samples were then post-cured using an AnyCubic curing system for 25 min and then dried under vacuum at 40 °C for 12 h prior to testing.

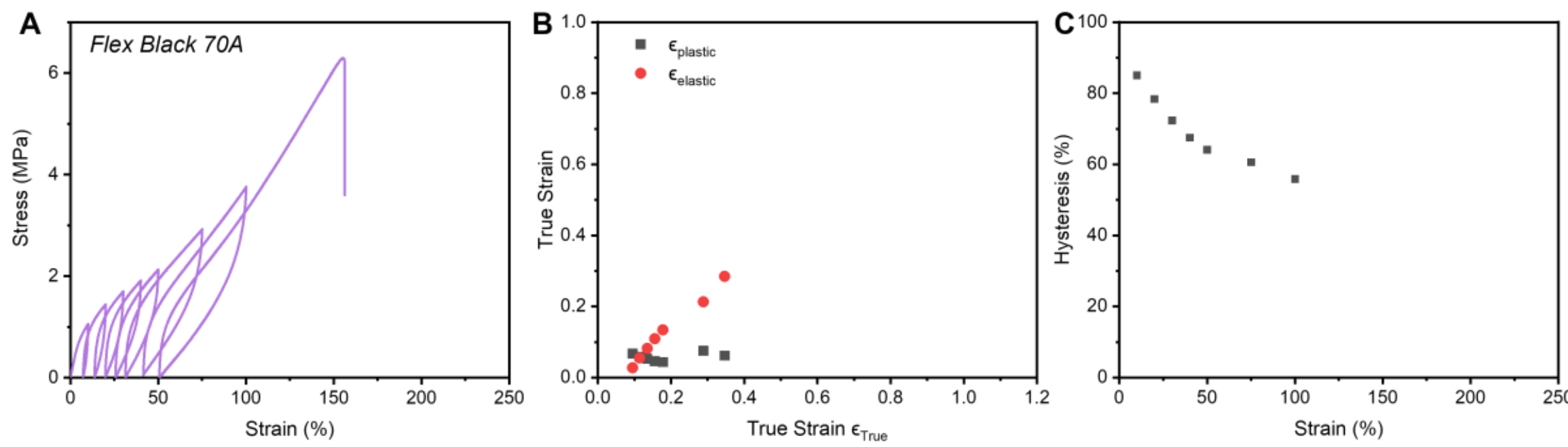

Figure S85. Stepwise cyclic tensile testing of printed dog-bone samples with Tenacious Flex Black 70A resin. (A) Stress-strain curve during the stepwise cyclic tensile testing. (B) Corresponding true strain, showing elastic and plastic behavior from stepwise cyclic tensile testing. (C) Corresponding hysteresis from stepwise cyclic tensile testing.

Table S49. Tabulated results from **Figure 5C**. Comparison of 3D printed elastomers prepared with commercial resins. Values are averages ($n \geq 3$) with ±1 standard deviation from the mean provided.

Resin	Printing conditions	Young's Modulus E_T (MPa)	Strain at break ε_f (%)	Tensile Strength σ_m (MPa)	Modulus of Toughness U_t (MJ/m^3)	Hyst-eresis (%)	Hardness (Shore A)	Fracture Toughness G_c (kJ/m^2)	Fracto-cohesive length L_f=G_c / W_F (mm)
FLX-300 (AmeraLabs)	6s/100 µm (5 mW/cm^2) 405 nm	4.6 ± 0.4	286 ± 25	7.9 ± 0.4	8 ± 1	34.6	59 ± 2	1.26 ± 0.08	0.15 ± 0.02
Silicone 40A (Formlabs)	Form 3 SLA	1.9 ± 0.1	203 ± 10	6.1 ± 0.7	5 ± 1	17.0	40.0 ± 0.4	0.57 ± 0.05	0.10 ± 0.02
Elastic 40A (Formlabs)	Form 3 SLA	2.77 ± 0.09	134 ± 18	2.7 ± 0.7	1.74 ± 0.4	12.8	51.2 ± 0.8	0.56 ± 0.03	0.32 ± 0.07
Rebound 55A (Siraya Tech)	6s/100 µm (5 mW/cm^2) 405 nm	12 ± 1	197 ± 34	6 ± 2	7 ± 2	36.1	68.5 ± 0.7	1.3 ± 0.1	0.19 ± 0.05
Flex Black 70A (Siraya Tech)	6s/100 µm (5 mW/cm^2) 405 nm	20.9 ± 0.8	141 ± 5	8.8 ± 0.7	6.3 ± 0.6	55.8	74.6 ± 0.8	1.47 ± 0.08	0.23 ± 0.03
PolyEA-30s *(post-cure)*	30s/100 µm (5 mW/cm^2) 365 nm	0.79 ± 0.01	586 ± 35	1.9 ± 0.1	4.6 ± 0.4	10.7	26.3 ± 0.3	0.8 ± 0.1	0.18 ± 0.03

S2.23 Weathering tests of 3D prints

The weathering test represents harsh conditions to accelerate potential aging of materials as they are exposed to sunlight and moisture during real-world usage. To assess the stability of the formulations, they were aged in a Binder KBF-LQC climate chamber for 1 and 5 cycles. The exposure cycle consists of 8 h UVA at 60°C/50 % RH followed by 4 h at 50°C/50 % RH.

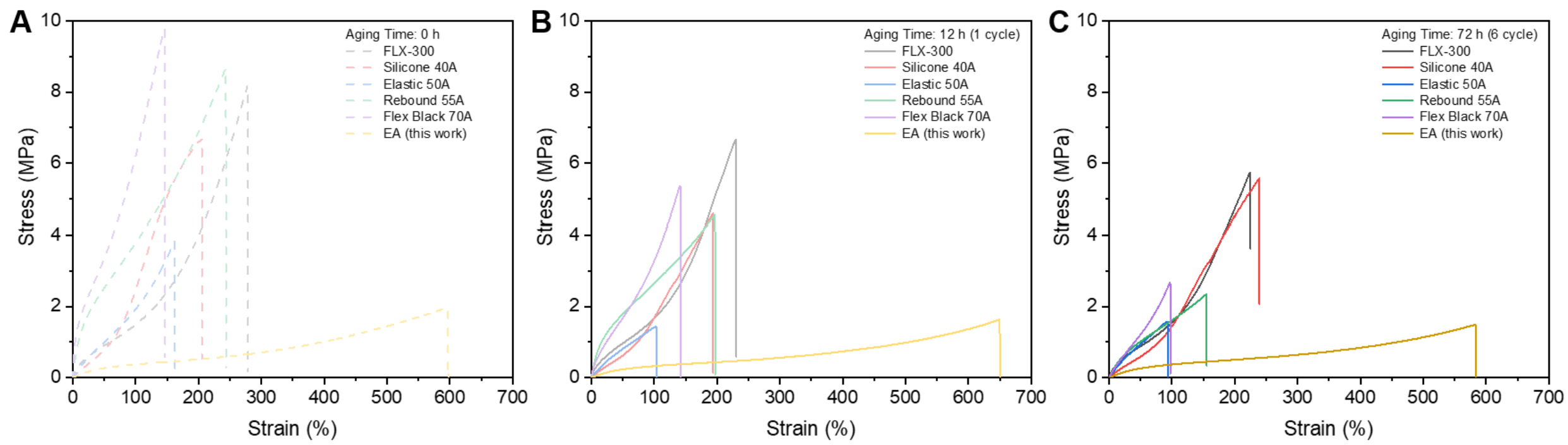

Figure S86. Representative uniaxial tensile stress-strain curves of commercial resin 3D printed dog-bone samples and 3D printed entangled poly(ethyl acrylate) elastomers upon accelerated aging. (A) no aging; (B) samples placed in the aging chamber for 12 hours (1 cycle); (C) samples place in the aging chamber for 72 hours (6 cycles).

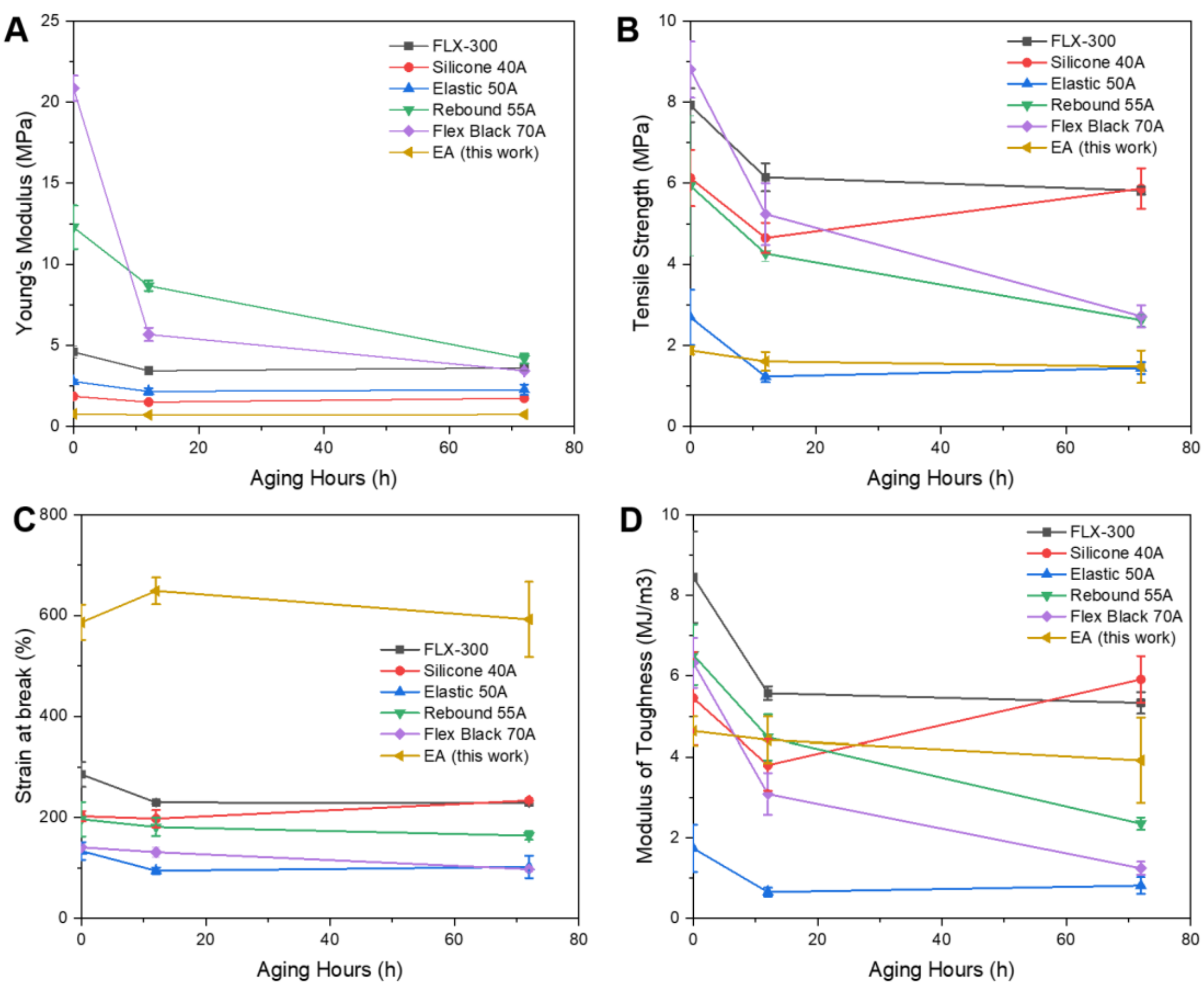

Figure S87. Mechanical properties of commercial resin 3D printed dog-bone samples and 3D printed entangled poly(ethyl acrylate) elastomers after different aging cycles. (A) Young's modulus; (B) Strain at break; (C) Tensile strength; (D) Modulus of toughness. Symbols are averages (n ≥3) with error bars representing ±1 standard deviation from the mean.

Table S50. Tabulated results from **Figure S87**. The change in mechanical properties for 3D printed elastomers prepared with commercial resins and highly entangled elastomer resin. Values are averages (n ≥3) with ±1 standard deviation from the mean provided.

Aging Time (h)	Resin	Young's Modulus E_T (MPa)	Strain at break ε_f (%)	Tensile Strength σ_m (MPa)	Modulus of Toughness U_t (MJ/m^3)
12 (1 cycle)	FLX-300 (AmeraLabs)	3.43 ± 0.06	230 ± 3	6.2 ± 0.3	5.6 ± 0.2
	Silicone 40A (Formlabs)	1.50 ± 0.09	198 ± 17	4.7 ± 0.4	3.8 ± 0.6
	Elastic 40A (Formlabs)	2.2 ± 0.2	94 ± 6	1.2 ± 0.1	0.7 ± 0.1
	Rebound 55A (Siraya Tech)	8.7 ± 0.3	181 ± 18	4.3 ± 0.2	4.5 ± 0.6
	Flex Black 70A (Siraya Tech)	5.7 ± 0.4	131 ± 10	5.2 ± 0.8	3.1 ± 0.5
	PolyEA-30s *(post-cure)*	0.71 ± 0.04	649 ± 27	1.6 ± 0.2	4.4 ± 0.6
72 (5 cycle)	FLX-300 (AmeraLabs)	3.6 ± 0.1	230 ± 5	5.8 ± 0.1	5.3 ± 0.3
	Silicone 40A (Formlabs)	1.73 ± 0.09	233.8 ± 0.4	5.9 ± 0.5	5.9 ± 0.6
	Elastic 40A (Formlabs)	2.3 ± 0.3	102 ± 22	1.4 ± 0.2	0.8 ± 0.2
	Rebound 55A (Siraya Tech)	4.2 ± 0.3	164 ± 8	2.6 ± 0.1	2.4 ± 0.2
	Flex Black 70A (Siraya Tech)	3.43 ± 0.09	98 ± 6	2.7 ± 0.3	1.2 ± 0.2
	PolyEA-30s *(post-cure)*	0.74 ± 0.04	592 ± 75	1.5 ± 0.4	4 ± 1

S2.24 Additional photos of 3D prints

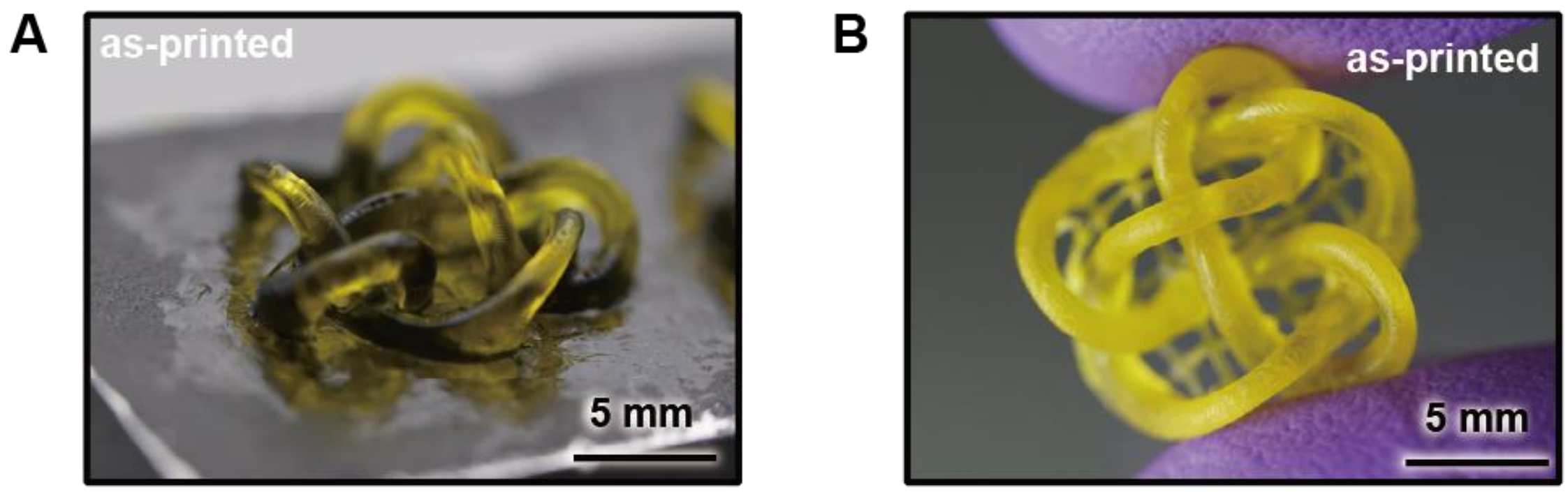

Figure S88. (A/B) Photographs of an as-printed (no UV post-curing) 3D 7_7 knot, prepared using the hydrogel system. Conditions: [LAP]/[AAm] = 0.001 mol%, [XL]/[AAm] = 0.01 mol%, [Tartrazine] = 0.05 wt%, [DI H_2O] = 33 wt%, 5 mW/cm^2 365 nm LED, 60 s per 100 µm layer, 35 °C.

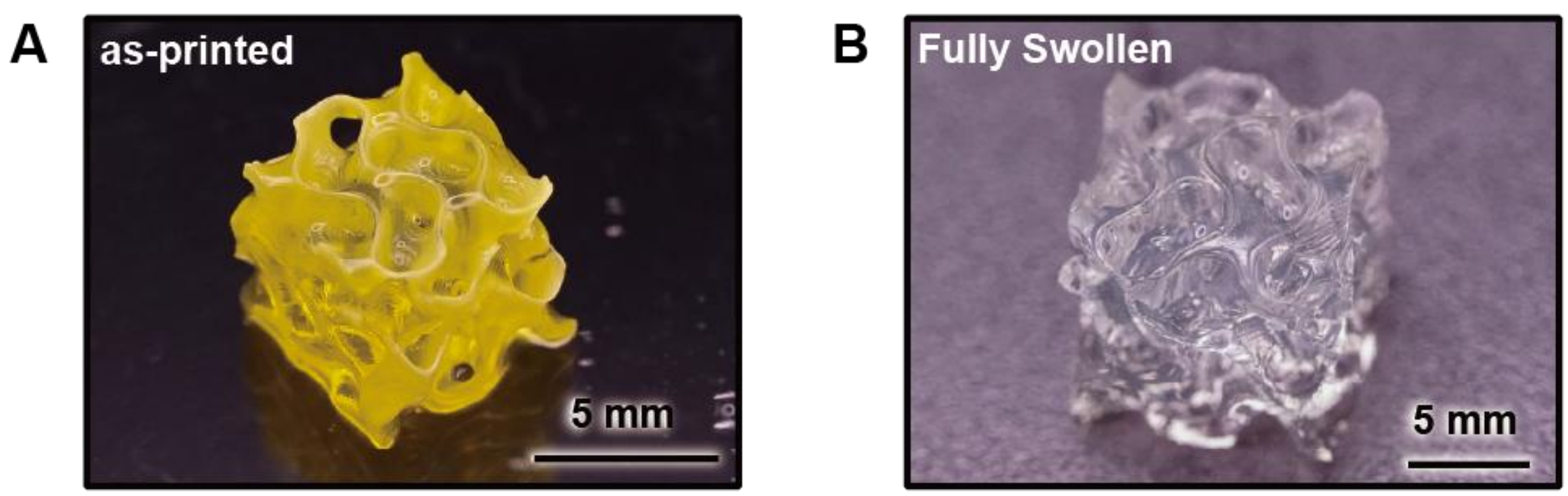

Figure S89. (A) Photograph of an as-printed (no UV post-curing) "Gyroid"[S16], prepared using the hydrogel system. Conditions: [LAP]/[AAm] = 0.001 mol%, [XL]/[AAm] = 0.01 mol%, [Tartrazine] = 0.05 wt%, [DI H_2O] = 33 wt%, 5 mW/cm^2 365 nm LED, 60 s per 100 µm layer. (A) Photograph of the fully swollen 3D-printed "Gyroid" after the sample shown in (A) was submerged in DI H_2O for 24 h.

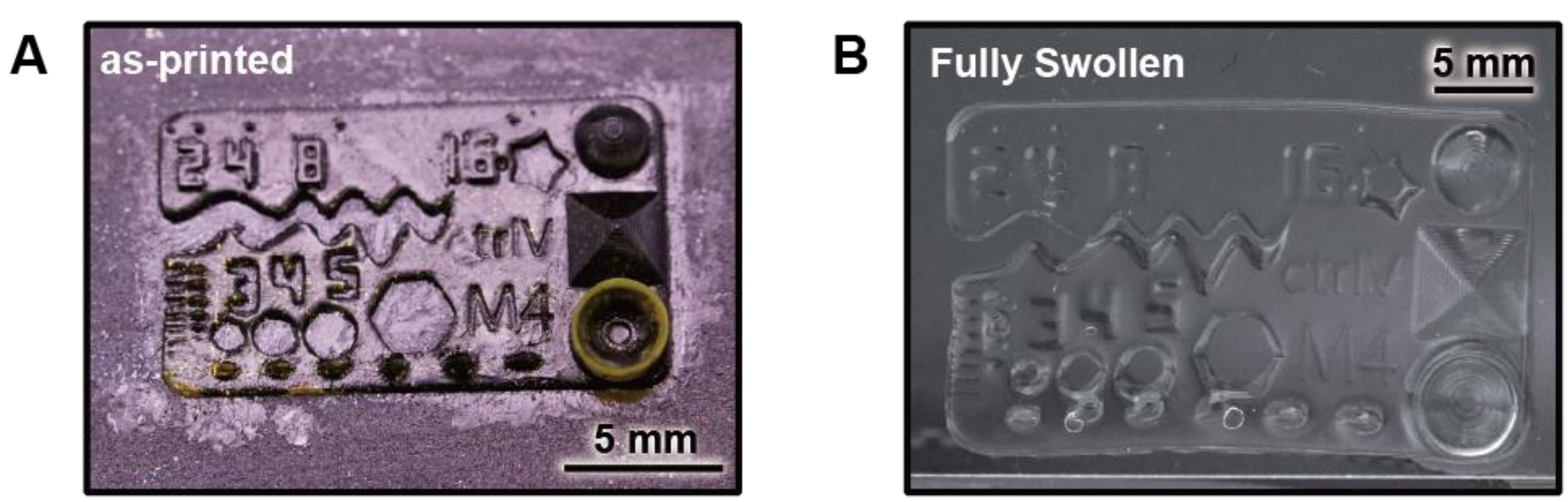

Figure S90. (A) Photograph of an as-printed (no UV post-curing) "Test your printer" print, prepared with the hydrogel system. Conditions: [LAP]/[AAm] = 0.001 mol%, [XL]/[AAm] = 0.01 mol%, [Tartrazine] = 0.05 wt%, [DI H_2O] = 33 wt%, 5 mW/cm^2 365 nm LED, 60 s per 100 µm layer, 35 °C. (B) Photograph of the fully swollen 3D-printed "Test your printer" after the sample shown in (A) was submerged in DI H_2O for 24 h.

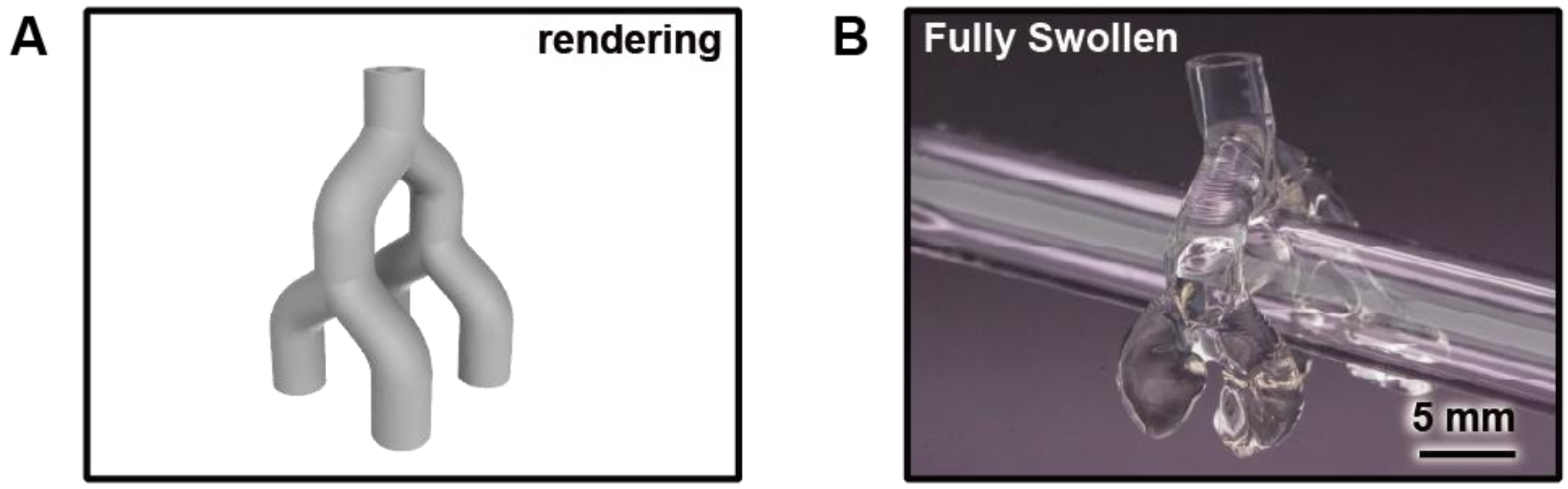

Figure S91. Blood vessel print. (A) Digital rendering of the print file prepared in-house and (B) photographs of the fully swollen 3D-printed (no UV post-curing) hydrogel blood vessel after the sample was submerged in DI H_2O for 24 h. Conditions: [LAP]/[AAm] = 0.001 mol%, [XL]/[AAm] = 0.01 mol%, [Tartrazine] = 0.05 wt%, [DI H_2O] = 33 wt%, 5 mW/cm^2 365 nm LED, 60 s per 100 µm layer, 35 °C.

S2.25 Mechanical properties of 3D printed highly entangled hydrogels

Table S51. Summary of mechanical properties for 3D printed highly entangled hydrogels. Conditions: [LAP]/[AAm] = 0.001 mol%, [XL]/[AAm] = 0.01 mol%, [Tartrazine] = 0.05 wt%. Printing conditions are listed as LED Intensity (exposure time per 100 µm layer). Values are averages ($n \geq 3$) with ±1 standard deviation from the mean provided.

Printing Condition (mW/cm^2)/Time	Swollen ratio	Water Content (wt%)	Young's Modulus E_T (kPa)	Strain at break ε_f (%)	Tensile Strength σ_m (kPa)	Modulus of Toughness U_t (kJ/m^3)	Fracture Toughness G_c (kJ/m^2)
5(60s)	4.9 ± 0.0	88.2 ± 0.1	54 ± 1	606 ± 25	316 ± 18	798 ± 40	0.8 ± 0.1
5(60s) PostCure	4.8 ± 0.1	86.8 ± 0.4	72 ± 2	602 ± 19	380 ± 16	940 ± 87	1.2 ± 0.1
10(30s)	5.6 ± 0.4	91.7 ± 0.2	41 ± 5	679 ± 81	100 ± 12	285 ± 14	
10(30s) PostCure	5.0 ± 0.1	87.2 ± 0.6	71 ± 9	644 ± 38	318 ± 43	825 ± 86	
10(60s)	4.9 ± < 0.1	87.6 ± 0.2	75 ± 5	595 ± 20	253 ± 20	657 ± 44	
10(60s) PostCure	4.7 ± < 0.1	86.1 ± 0.7	88 ± 6	535 ± 55	279 ± 58	794 ± 26	
50(20s)	5.8 ± 0.1	91.9 ± 0.2	33 ± 2	804 ± 20	198 ± 17	607 ± 33	
50(20s) PostCure	5.2 ± 0.1	89.4 ± 0.7	57 ± 9	500 ± 43	149 ± 25	352 ± 75	
50(40s)	5.3 ± 0.1	89.0 ± 0.8	55 ± 2	514 ± 57	135 ± 23	286 ± 63	N/A
50(40s) PostCure	4.8 ± 0.1	87.6 ± 0.4	65 ± 8	472 ± 53	160 ± 24	345 ± 62	
100(10s)	6.0 ± 0.1	95.7 ± 0.1	17 ± 2	Strain Reached Testing Limit			
100(10s) PostCure	5.6 ± < 0.1	92.8 ± 0.8	28 ± 3	558 ± 83	72 ± 13	205 ± 50	
100(20s)	7.7 ± 0.2	94.8 ± 0.4	15.5 ± 0.8	Strain Reached Testing Limit			
100(20s) PostCure	6.6 ± 0.5	93 ± 1	31 ± 6	456 ± 74	68 ± 13	140 ± 43	

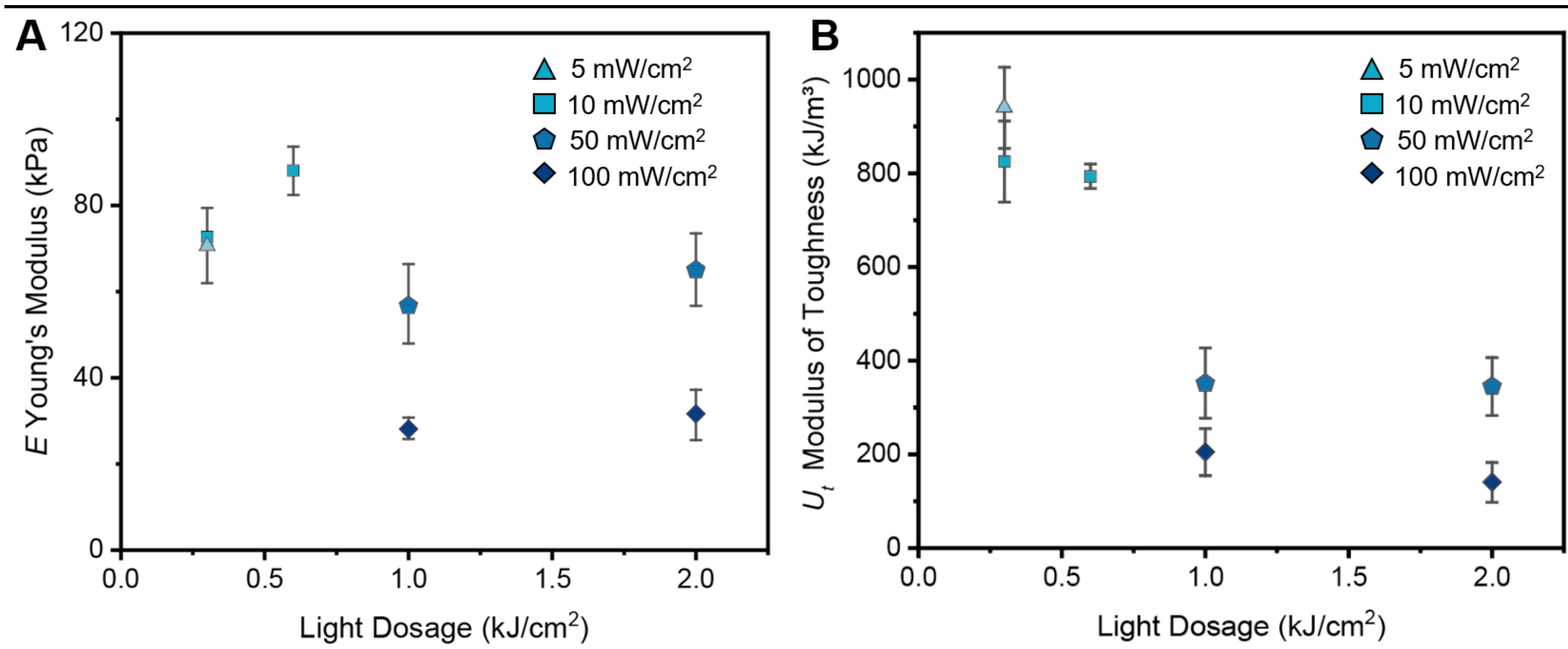

Figure S92. Examining the influence of light dosage on mechanical performance via tuning exposure intensity for hydrogel resins used to print dog-bone samples that were then post-cured under a 365 nm LED (5 mW/cm^2) for 10 min. Resin formulation: [LAP]/[AAm] = 0.001 mol%, [XL]/[AAm] = 0.01 mol%, [Tartrazine] = 0.05 wt%, [DI H_2O] = 33 wt%. Printing conditions: 365 nm LED, 100 µm layer, 35 °C. (A) Light dosage vs Young's modulus. (B) Light dosage vs modulus of toughness (U_T). Results summarized in **Table S51**. Values are averages ($n \geq 3$) with ±1 standard deviation from the mean provided.

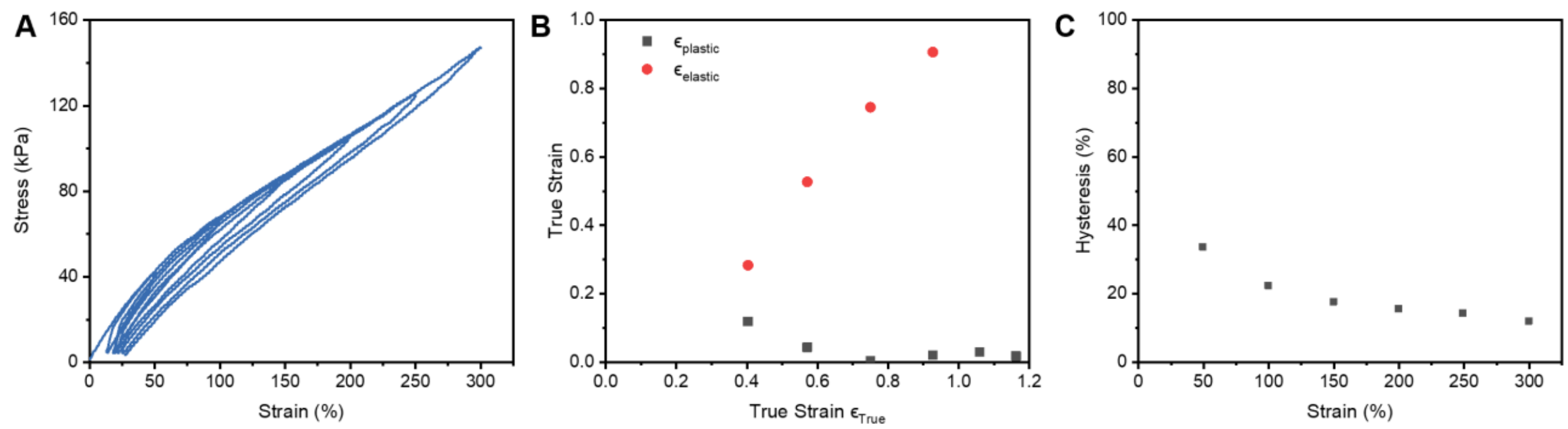

Figure S93. Stepwise cyclic tensile testing of 3D printed highly entangled hydrogel dogbones that were post-cured under a 365 nm LED (5 mW/cm^2) for 10 min. Resin formulation: [LAP]/[AAm] = 0.001 mol%, [XL]/[AAm] = 0.01 mol%, [Tartrazine] = 0.05 wt%, [DI H_2O] = 33 wt%. Printing condition: 5 mW/cm^2 365 nm LED, 60 s per 100 µm layer, 35 °C. (A) Stress-strain curve during the stepwise cyclic tensile testing. (B) Corresponding true strain, showing elastic and plastic behavior from stepwise cyclic tensile testing. (C) Corresponding hysteresis from stepwise cyclic tensile testing. Cyclic strains of 50, 100, 150, 200, 250, and 300% were set, relaxing to 0.02 N between cycles. All loading and unloading cycles were run using a 0.02 s^{-1} strain rate.

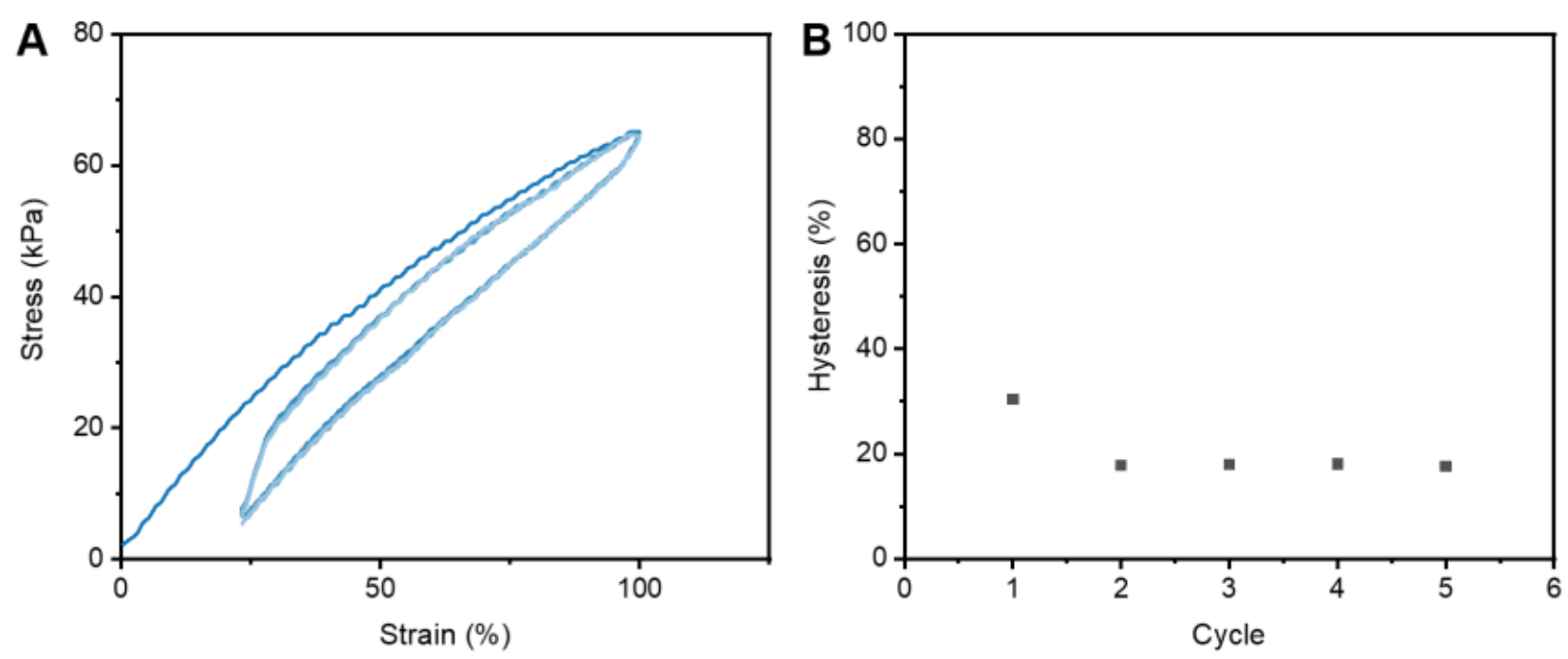

Figure S94. Cyclic tensile testing of 3D printed highly entangled hydrogel dogbones that were post-cured under a 365 nm LED (5 mW/cm^2) for 10 min. Resin formulation: [LAP]/[AAm] = 0.001 mol%, [XL]/[AAm] = 0.01 mol%, [Tartrazine] = 0.05 wt%, [DI H_2O] = 33 wt%. Printing condition: 5 mW/cm^2 365 nm LED, 60 s per 100 µm layer, 35 °C. (A) Stress-strain curve during the cyclic tensile tests. (B) Corresponding hysteresis from stepwise cyclic tensile testing. Cyclic strains of 100% were set, relaxing to 0.02 N between cycles for a total of five cycles. All loading and unloading cycles were run using a 0.02 s^{-1} strain rate.

Table S52. Tabulated results from **Figure 5F**. Comparison of 3D printed highly entangled hydrogels with previously reported 3D printed hydrogels. Values with error (±) provided represent averages (*n* ≥3) with ±1 standard deviation from the mean provided.

Resin	Young's Modulus E_T (kPa)	Modulus of Toughness U_t (kJ/m^3)	Strain at break ε_f (%)	Tensile Strength σ_m (kPa)	Reference
CLEAR	69.6	884.3	151.3	186.1	ref[S17] (Figure S29A-C)*
	154.4	1655.4	375.4	231.1	
	199.8	681.6	1468.5	187.3	
	261.7	485.1	1215.4	128.0	
CLEAR	50 – 290	150-1650	/	/	ref[S17]
Highly Entangled AAm	100-200	20-600	/	/	ref[S17,18]
IPN	19.6	21.6	114.3	45.6	ref[S19] (Figure 4)*
5mW/cm^2 60s	54 ± 1	798 ± 40	606 ± 25	316 ± 18	This Work (DLP 3D Printing)
5mW/cm^2 60s (*post-cure*)	72 ± 2	940 ± 87	602 ± 19	380 ± 16	
10mW/cm^2 60s (*post-cure*)	88 ± 6	794 ± 26	535 ± 55	279 ± 58	
50mW/cm^2 20s (*post-cure*)	57 ± 9	352 ± 75	500 ± 43	149 ± 25	
100mW/cm^2 10s (*post-cure*)	28 ± 3	205 ± 50	558 ± 83	72 ± 13	
[LAP]/[AAm] 0.01 (mol%)	143 ± 7	131 ± 3	175 ± 3	141 ± 5	This Work (Casted)
[LAP]/[AAm] 0.001 (mol%)	122 ± 4	8983 ± 99	515 ± 19	370 ± 42	
[LAP]/[AAm] 0.0001 (mol%)	66 ± 3	1807 ± 196	876 ± 16	628 ± 68	

*Data from reference was obtained via extracting data from figures via Webplotdigitizer

S3 References

[S1] M. H. Stenzel, L. Cummins, G. E. Roberts, T. P. Davis, P. Vana, C. Barner-Kowollik, *Macromolecular Chemistry and Physics* **2003**, *204*, 1160.

[S2] O. Grinevich, D. L. Snavely, *Chemical Physics Letters* **1999**, *304*, 202.

[S3] K. Liang, Z. Wang, W. Quan, Y. Shi, H. Zhou, L. Bi, Z. Yin, N. Romero, M. Young, S. Li, *Science* **2026**, *391*, 807.

[S4] K. Hong, A. Rastogi, G. Strobl, *Macromolecules* **2004**, *37*, 10165.

[S5] Y. Men, G. Strobl, *Macromolecules* **2003**, *36*, 1889.

[S6] H. W. Greensmith, *Journal of Applied Polymer Science* **1963**, *7*, 993.

[S7] K. S. Mason, J.-W. Kim, E. A. Recker, J. M. Nymick, M. Shi, F. A. Stolpen, J. Ju, Z. A. Page, *ACS Cent. Sci.* **2025**, *11*, 975.

[S8] Z. Wu, Z. He, Y. Zhou, T. Kou, K. Gong, F. Nan, T. T. Bezuneh, S. Han, C. Boyer, W. W. Yu, *Angewandte Chemie International Edition* **2025**, *64*, e202422975.

[S9] J. Li, C. Ding, Z. Zhang, X. Pan, N. Li, J. Zhu, X. Zhu, *Macromolecular Rapid Communications* **2017**, *38*, 1600482.

[S10] Z. Zhang, D. Su, M. Zhang, Z. An, C. Boyer, C. Wu, *Angewandte Chemie International Edition* **2026**, *65*, e19602.

[S11] S. Jockusch, N. J. Turro, *J. Am. Chem. Soc.* **1998**, *120*, 11773.

[S12] S. Jockusch, I. V. Koptyug, P. F. McGarry, G. W. Sluggett, N. J. Turro, D. M. Watkins, *J. Am. Chem. Soc.* **1997**, *119*, 11495.

[S13] J. H. Lee, R. K. Prud'homme, I. A. Aksay, *Journal of Materials Research* **2001**, *16*, 3536.

[S14] Thingiverse.com, "Tricolored Projection of Knot 7_7 by mathgrrl," can be found under https://www.thingiverse.com/thing:1195364, **n.d.**

[S15] Thingiverse.com, "Test your 3D printer! v2 by ctrlV," can be found under https://www.thingiverse.com/thing:1019228, **n.d.**

[S16] Thingiverse.com, "Gyroid Cube by moczys," can be found under https://www.thingiverse.com/thing:757884, **n.d.**

[S17] A. P. Dhand, M. D. Davidson, H. M. Zlotnick, T. J. Kolibaba, J. P. Killgore, J. A. Burdick, *Science* **2024**, *385*, 566.

[S18] J. Kim, G. Zhang, M. Shi, Z. Suo, *Science* **2021**, *374*, 212.

[S19] A. P. Dhand, M. D. Davidson, J. H. Galarraga, T. H. Qazi, R. C. Locke, R. L. Mauck, J. A. Burdick, *Advanced Materials* **2022**, *34*, 2202261.
